# A cross-national analysis of demographic variation in belief in God, gods, or spiritual forces in 22 countries

**DOI:** 10.1038/s41598-024-79103-w

**Published:** 2025-04-30

**Authors:** Eric Y. Aglozo, Kathryn A. Johnson, Brendan Case, R. Noah Padgett, Byron R. Johnson, Tyler J. VanderWeele

**Affiliations:** 1 Psychology Department, Arizona State University, Tempe, AZ USA; 2 Human Flourishing Program, Institute for Quantitative Social Science, Harvard University, Cambridge, MA USA; 3 Department of Economics, Baylor University, Waco, TX USA; 4 Department of Biostatistics, Harvard T.H. Chan School of Public Health, Boston, MA USA

**Keywords:** Global flourishing study, Belief, Belief in God, Cross-cultural, Psychology, Human behaviour

## Abstract

Although prior research documents the importance of belief in God (e.g., for health and well-being), most of the research has focused on Western samples. Much less is known about how belief in one God, multiple gods, or spiritual forces (“Belief in God”) differs across cultures and demographic groups within those cultures. Using a diverse and international dataset of over 200,000 individuals from 22 countries, we examined the proportions of Belief in God across key demographics, focusing on country, age, gender, marital status, employment status, religious service attendance, education, and immigration status. Being mindful of interpretative challenges due to varying cultural contexts and the nature of the response items used, we offer insight into country-specific variations in Belief in God and lay a foundation for future investigations into sociocultural influences that might shape—or be shaped by—belief (or non-belief) in God, gods, and spiritual forces.

**Supplementary Information:**

The online version contains supplementary material available at 10.1038/s41598-024-79103-w.

## Introduction

Religion has basic components which vary in content and salience across cultures^1^. One component is belief in the existence of God, gods, or spiritual forces^2^. However, there is limited research on how such beliefs might differ across cultures and demographic groups within those cultures. In the present research, we used data from the Global Flourishing Study to investigate the proportion of people believing in God, gods, or spiritual forces in 22 countries. We explored how this belief (and non-belief) varies across different demographic categories.

## Ancient to contemporary belief in God, gods, or spiritual forces

Belief in non-human or non-living intelligences goes back deep into our human history. Archaeological evidence reveals paleolithic burial practices and the ritual use of red ochre, which are frequently associated with belief in an afterlife, even among hunter-gatherer societies to the present day^3^. Peoples et al^4^. found that every one of the 33 contemporary hunter-gatherer societies they examined across five continents endorsed some version of “animism,” such as the belief that all things in nature have intentionality and at least some influence on human lives. These nature spirits still throng the imaginations of many societies worldwide—including the kami or nature spirits of Japanese Shinto, a rare animist tradition that has survived the transition to urban civilization relatively intact^5^.

From a universal foundation of animism, human societies gradually (and, in some cases, independently) revised their conceptions of non-human, non-living spirits to be more powerful, knowledgeable, and moral. Societies with “bigger,” more powerful, and morally admirable gods may have had substantial advantages. In contrast to local spirits, who are often thought to be as capricious and immoral as any human, the belief in a powerful God or gods who see their followers’ every act and who promise post-mortem punishments for misdeeds and rewards for virtue are more likely to foster honesty, cooperation, and non-malevolence in their worshippers^6,7^.

Thus, today the world’s religious landscape is dominated by theists from multiple world religions, including Christianity (31.7% of the global population), Islam (25%), Hinduism (15%), Buddhism (6.6%), and the smaller communions of Sikhism (0.3%) and Judaism (0.18%). Adherents of primary – or “folk” – religions continue to make up about 5.6% of the world^8^.

However, empirical studies on religious beliefs, including belief in God and demographic variations in religious beliefs, have predominantly been conducted in Western societies, including Europe, Canada, and the United States^9,10^. Even less is known about the global distribution of non-belief (i.e., agnosticism or atheism).

## Society, belief, and non-belief

The prevailing social and ecological conditions within a society can influence the prevalence or absence of religious belief^11^. Religion holds increased importance in nations characterized by existential insecurities, including mortality, poverty, and safety concerns^12^. In such nations, religiousness can be a useful coping resource that contributes to well-being. Atheism is probably less likely in these nations, as credibility-enhancing displays of religious commitments are more likely to be observed^13,14^.

However, the number of individuals indicating no belief in God, gods, or spiritual forces, or the proportion of individuals identifying as atheists, is rising in some regions of the world, including the United States, United Kingdom, Sweden, France, and the Netherlands^15^. Given the pervasive prejudice and discrimination against atheists, the number of atheists may be underestimated, even in surveys conducted anonymously^16^. Negative attitudes toward atheists may differ across various cultures and nations. In certain African and Muslim-majority societies where religion is essential to civic as well as cultural identity, lack of belief is met with disapproval^17^. Moreover, there are severe structural and legal constraints on leaving religion in Muslim-majority societies (such as Afghanistan, Brunei, and Malaysia), where apostasy and blasphemy are considered crimes, in some cases punishable by death^18,19^.

The prevalence of religious belief and non-belief also might differ across demographic groups, including age, gender, education, and immigration status. Past studies have observed that older people tend to be more religious than younger people^20^. However, it is debatable whether there is indeed an age effect or if it is instead a cohort effect. For instance, Argue et al^21^. found that religiosity was more prevalent among individuals between 18 and 30 years old compared with older age groups. Shulgin et al^9^. addressed the age versus cohort effect using World Values Survey data (1981–2014), finding that in developed nations, age has a stronger effect than cohort. Older people reported more belief in God and identified more as religious (as opposed to atheist) compared to younger people. However, the increase in religiousness may not be due to age per se but to the challenges associated with growing older^22^.

Gender difference in religiousness is well-documented, with females being more religious than males^23^. Mind perception and preference for risk may be contributory factors to the difference. The ability to represent supernatural agents is a byproduct of the more general cognitive process of “mentalizing,” or perceiving other minds, an ability in which women, on average, excel more than men^24^. Furthermore, non-belief is considered risky within the framework of “Pascal’s wager” (which proposes that the potential post-mortem risks of atheism are higher than the risks of theism), and men are higher on risk preference, reflecting their lower religious commitment compared to women^25^.

Education’s impact on religiosity is mixed. Glaeser and Sacerdote^26^note that secular education can undermine religious beliefs (e.g., in miracles and the Devil) due to conflicts with secular views. Conversely, they find that education positively affects religious attendance by enhancing social skills and increasing participation in social activities like church attendance. In contrast, Arias-Vazquez finds that education is negatively associated with both religious attendance and the importance placed on religion^27^.

Immigrants tend to exhibit higher levels of religiousness compared to the population in the receiving countries, including the US and European countries^28^. The religiosity of immigrants is often believed to stem from a desire to assimilate into the local population, in addition to serving as a coping mechanism against challenges encountered in the receiving country. In line with these motives, religiousness tends to be more pronounced among unemployed immigrants who possess lower education and have recently arrived in the host country^29^.

Considering the potential diversity in belief in the transcendent (such as God, multiple gods, and spiritual forces) as well as non-belief, this study seeks to investigate the proportion of theists and atheists across a global sample of 22 countries. We expect meaningful variation across different countries in the proportion endorsing belief versus non-belief—whether that belief be in God, gods, or impersonal spiritual forces. In addition, this proportion is expected to vary across different demographic categories.

## Method

The description of the methods below has been adapted from VanderWeele et al^30^. Further methodological detail is available elsewhere^31–36^.

## Data

The Global Flourishing Study (GFS) is a study of 202,898 participants from 22 geographically and culturally diverse countries, with nationally representative sampling within each country, primarily focused on the distribution of potential determinants of well-being. Wave 1 of the data included the following countries and territories: Argentina, Australia, Brazil, Egypt, Germany, Hong Kong, India, Indonesia, Israel, Japan, Kenya, Mexico, Nigeria, the Philippines, Poland, South Africa, Spain, Sweden, Tanzania, Turkey, United Kingdom, and the United States. The countries were selected to (a) maximize coverage of the world’s population, (b) ensure geographic, cultural, and religious diversity, and (c) prioritize feasibility and existing data collection infrastructure. Data collection was carried out by Gallup Inc. Data for Wave 1 were collected principally during 2023, with some countries beginning data collection in 2022 and exact dates varying by country^35^. Four additional waves of panel data on the participants will be collected annually from 2024 to 2027. The precise sampling design ensures that nationally representative samples vary by country, and further details are available in Ritter et al^35^..

Survey items included aspects of well-being such as happiness, health, meaning, character, relationships, and financial stability^37^ along with other demographic, social, economic, political, personality, childhood, community, health, and religious variables. The data are publicly available through the Center for Open Science (https://www.cos.io/gfs). During the translation process, Gallup adhered to the TRAPD model (translation, review, adjudication, pretesting, and documentation) for cross-cultural survey research (ccsg.isr.umich.edu/chapters/translation/overview).

## Measures

### Demographics variables

Continuous age was classified as 18–24, 25–29, 30–39, 40–49, 50–59, 60–69, 70–79, and 80 or older. Gender was assessed as male, female, or other. Marital status was assessed as single/never married, married, separated, divorced, widowed, and domestic partner. Employment was assessed as employed, self-employed, retired, student, homemaker, unemployed and searching, and other. Education was assessed as up to 8, 9–15, and 16 + years. Religious service attendance was assessed less than once/week, once/week, one to three times/month, a few times/year, or never. Immigration status was dichotomously assessed: “Were you born in this country or not?”

Religious tradition/affiliation with categories of Christianity, Islam, Hinduism, Buddhism, Judaism, Sikhism, Baha’i, Jainism, Shinto, Taoism, Confucianism, Primal/Animist/Folk religion, Spiritism, African-Derived, some other religion, or no religion/atheist/agnostic; precise response categories varied by country^38^. Racial/ethnic identity was assessed in some, but not all, countries, with response categories varying by country. For additional details on the assessments, see the COS GFS codebook or Crabtree et al^31^..

## Outcome variable

Belief in God (the primary outcome variable) was assessed with the question: “Do you believe in one God, more than one god, an impersonal spiritual force, or none of these?” Respondents chose one response, “I believe in one God”; “I believe in more than one god”; I believe in an impersonal spiritual force”; “None of the above”; or “Unsure.”

In the present research, the variable was dichotomized as either Belief in God (i.e., belief in one God, gods, or spiritual forces) or Non-belief (i.e., non-belief or unsure) for theoretical and statistical reasons. First, people imagine the divine—the transcendent, the supernatural, the spiritual—in myriad ways. A Christian would most likely report belief in one God, but adherents of other monotheistic religions might counter that belief in a Trinitarian God is equivalent to belief in more than one god. Similarly, for many Hindus, all the “gods” are one (i.e., Brahman)^1,2^. Some theists represent the divine as a personified being, whereas others think of the divine as a cosmic force, a “higher power,” or “the universe”^3^. Moreover, belief in other spiritual forces (e.g., jinn, angels, curses) often coexists with belief in a supreme being^4–6^. In the present study, we are interested in belief in any and all of these forms of metaphysical belief—belief in God, gods, or spiritual forces—and we have combined these as the positive “Belief in God” variable. Furthermore, there are many forms of atheism, such as those who firmly believe the metaphysical does not exist, unbelievers who have concluded one cannot know, or those who simply do not care. We have combined these types and refer to them as non-belief in the “Belief in God” variable.

Pragmatically speaking, a descriptive report of each of the five response categories across 22 countries for all the levels of our eight key demographic variables would require a massive number of tables, even exceeding the 94 pages of tables in the Supplemental Materials. However, the data is open and available to any interested researcher in an online repository^34^.

### Data analysis

Descriptive statistics for the full sample, weighted to be nationally representative within each country, were estimated for each demographic variable. Nationally representative means/proportions for Belief in God were estimated separately for each country and ordered from highest to lowest, along with 95% confidence intervals. Variations in proportions in Belief in God across demographic categories were estimated, with all analyses initially conducted by country (online supplement).

Primary results consisted of random effects meta-analyses of country-specific proportions of belief in God in each specific demographic category^39,40^along with 95% confidence intervals, standard errors, upper and lower limits of a 95% prediction interval across countries, heterogeneity (τ), and I^2^for evidence concerning variation within a particular demographic variable across countries^41^. Forest plots of estimates are available in the online supplement. All meta-analyses were conducted in R^42^using the metafor package^43^.

Within each country, a global test of variation of outcome across levels of each particular demographic variable was conducted, and a pooled p-value^44^across countries was reported concerning evidence for variation within any country. Bonferroni corrected p-value thresholds are provided based on the number of demographic variables^45,46^. Religious affiliation/tradition and race/ethnicity were used, when available, as control variables within the country but were not included in the meta-analyses since the availability of these response categories varied by country. Population-weighted meta-analyses were also conducted as a supplementary analysis. All analyses were pre-registered with COS prior to data access (*osf.io/97a4h/registrations*); all code to reproduce analyses is openly available in an online repository^34^.

### Missing data

Missing data on all variables was imputed using multivariate imputation by chained equations, and five imputed datasets were used^47^. To account for variation in the assessment of certain variables across countries (e.g., religious affiliation/tradition and race/ethnicity), the imputation process was conducted separately in each country. This within-country imputation approach ensured that the imputation models accurately reflected country-specific contexts and assessment methods. Sampling weights were included in the imputation models to account for specific variable missingness that may have been related to the probability of inclusion in the study.

### Accounting for complex sampling design

The GFS used different sampling schemes across countries based on availability of existing panels and recruitment needs^35^. All analyses accounted for the complex survey design components by including weights, primary sampling units, and strata. Additional methodological detail, including accounting for the complex sampling design, is provided elsewhere.^133^

## Results

The descriptive statistics for the demographic variables for all 22 countries are provided in Table 1. Note that Table 1 does not use multiple imputations for missing data; it simply reports the proportions/numbers missing. In the total sample, the proportions of different age groups were generally similar, except that there were fewer participants aged 70 years or older (Table 1). The gender distribution was 51% female, 48% male, and less than 1% other gender. Most participants were married (52%), employed by an employer (39%), had 9–15 years of education (57%), never attended religious services (37%), and were native-born (94%). Sample sizes in each country ranged from 1,473 in Turkey to 38,312 in the United States. Participant characteristics for each country are presented in Supplementary Tables S1a to S22a, with the countries ordered alphabetically.

**Table 1:** Nationally representative descriptive statistics of the observed sample.

Characteristic	*N* = 202,898^1^
**Age group**	
18–24	27,007 (13%)
25–29	20,700 (10%)
30–39	40,256 (20%)
40–49	34,464 (17%)
50–59	31,793 (16%)
60–69	27,763 (14%)
70–79	16,776 (8.3%)
80 or older	4,119 (2.0%)
(Missing)	20 (< 0.1%)
**Gender**	
Male	98,411 (49%)
Female	103,488 (51%)
Other	602 (0.3%)
(Missing)	397 (0.2%)
**Marital status**	
Married	107,354 (53%)
Separated	5,195 (2.6%)
Divorced	11,654 (5.7%)
Widowed	9,823 (4.8%)
Never	52,115 (26%)
Domestic Partner	14,931 (7.4%)
(Missing)	1,826 (0.9%)
**Employment**	
Employed for an employer	78,815 (39%)
Self-employed	36,362 (18%)
Retired	29,303 (14%)
Student	10,726 (5.3%)
Homemaker	21,677 (11%)
Unemployed and looking for a job	16,790 (8.3%)
None of these/Other	8,431 (4.2%)
(Missing)	793 (0.4%)
**Education**	
Up to 8 years	45,078 (22%)
9–15 years	115,097 (57%)
16 + years	42,578 (21%)
(Missing)	146 (< 0.1%)
**Religious service attendance**	
>1/week	26,537 (13%)
1/week	39,157 (19%)
1–3/month	19,749 (9.7%)
A few times a year	41,436 (20%)
Never	75,297 (37%)
(Missing)	722 (0.4%)
**Immigration**	
Born in this country	190,998 (94%)
Born in another country	9,791 (4.8%)
(Missing)	2,110 (1.0%)
Argentina	6,724 (3.3%)
Australia	3,844 (1.9%)
Brazil	13,204 (6.5%)
Egypt	4,729 (2.3%)
Germany	9,506 (4.7%)
Hong Kong	3,012 (1.5%)
India	12,765 (6.3%)
Indonesia	6,992 (3.4%)
Israel	3,669 (1.8%)
Japan	20,543 (10%)
Kenya	11,389 (5.6%)
Mexico	5,776 (2.8%)
Nigeria	6,827 (3.4%)
Philippines	5,292 (2.6%)
Poland	10,389 (5.1%)
South Africa	2,651 (1.3%)
Spain	6,290 (3.1%)
Sweden	15,068 (7.4%)
Tanzania	9,075 (4.5%)
Turkey	1,473 (0.7%)
United Kingdom	5,368 (2.6%)
United States	38,312 (19%)

^1^n (%).

Table 2 provides the ordered proportions for Belief in God, 95% confidence intervals, and standard errors in each of the 22 countries. We note that care should be taken in interpreting differences in the ordered proportions because differences across countries can arise from different beliefs; however, in cross-cultural studies (the present research), results may also be a function of translation or participants’ interpretation of an item.

**Table 2:** Ordered proportions of belief in God.

Country	Proportion	95% CI	SE
Egypt	1.00	(1.00, 1.00)	< 0.001
Nigeria	0.99	(0.99, 1.00)	< 0.001
Kenya	0.98	(0.98, 0.99)	< 0.001
Tanzania	0.98	(0.98, 0.99)	< 0.001
Indonesia	0.97	(0.97, 0.98)	< 0.001
Brazil	0.96	(0.96, 0.96)	< 0.001
South Africa	0.96	(0.95, 0.97)	0.010
India	0.95	(0.95, 0.96)	< 0.001
Philippines	0.93	(0.92, 0.94)	0.010
Mexico	0.91	(0.90, 0.92)	< 0.001
Turkey	0.85	(0.83, 0.87)	0.010
Argentina	0.84	(0.83, 0.86)	0.010
Poland	0.83	(0.81, 0.84)	0.010
United States	0.73	(0.71, 0.74)	0.010
Israel	0.72	(0.69, 0.75)	0.020
Spain	0.64	(0.62, 0.65)	0.010
Hong Kong	0.59	(0.57, 0.62)	0.010
Germany	0.55	(0.54, 0.57)	0.010
Australia	0.54	(0.52, 0.56)	0.010
United Kingdom	0.54	(0.52, 0.56)	0.010
Sweden	0.44	(0.43, 0.45)	< 0.001
Japan	0.20	(0.19, 0.21)	< 0.001

Note. *N* = 202,898; Belief in God was assessed by the item “Do you believe in one God, more than one god, an impersonal spiritual force, or none of these?”; and categories collapsed as (Belief in God (i.e., belief in one God, gods, or spiritual forces [1]) or Non-belief (i.e., non-belief or unsure [0]); CI = confidence interval; SE = standard error; the combination of small SE and rounding can lead to nonsymmetric CI for some countries.

Table 3 is the primary table and provides the meta-analytic proportions for each of the demographic groups across the 22 countries, using a random effects meta-analysis (essentially, assuming that the proportion in each country is drawn from an underlying distribution of countries, corresponding to the 22 countries in the study). Supplementary Tables S1b-S22b provide the proportions of Belief in God for each demographic category, for each country. The estimated overall proportion and the 95% CI and lower and upper limits are provided in the meta-analysis for each demographic category, along with a standard error for the estimated overall proportion for each demographic group.

An estimate of “tau” is also provided in Table 3, which is the estimated standard deviation of the distribution of proportions of Belief in God across countries (i.e., an estimate of how much the proportion in a demographic category varies across countries). There may not be much difference in the proportion for a particular demographic, for example, between men and women across the 22 countries. However, a large tau might suggest that although the proportions are similar, there are some countries where women report considerably higher mean beliefs than the overall meta-analytic mean (Brazil, Egypt, India, Indonesia, Kenya, Mexico, Nigeria, Philippines, South Africa, Tanzania). Thus, a large tau suggests a need for a more nuanced examination of country-specific estimates in Supplementary Tables S1b-S22b described below. I^2 is reported to provide an additional estimate regarding variability due to heterogeneity across countries vs. sampling variability. Note, however, that I^2 may sometimes be quite large in this study due to the large sample size.

The final column in Table 3 provides a “global p-value.” The global p-value corresponds to a test of the null hypothesis that no significant differences exist in Belief in God vs. non-belief for that demographic category in any of the 22 countries. The p-values are all significant, providing evidence that, in at least one country, the proportion of belief in God differs across demographic categories within each variable. Supplementary Table S23 provides an alternative meta-analysis wherein instead of treating each country with equal weight (as in a random effects meta-analysis), each country’s results are weighted by the 2023 country population size. Further quantitative details on variation across countries are provided in the Online Supplement, and additional comments on these results are provided in the Discussion section below.

**Table 3:** Random effects meta-analysis of belief in God proportions by demographic category.

Prediction Interval									
Variable	Category	Proportion	95% CI of Proportion	SE Analogue (CI Width/4)	LL	UL	Heterogeneity (τ)	I^2^	Global *p*-value
Age group									< 0.001**
	18–24	0.85	(0.72,0.93)	0.05	0.23	1.00	0.24	99.1	
	25–29	0.88	(0.75,0.95)	0.05	0.23	1.00	0.23	99.2	
	30–39	0.88	(0.75,0.95)	0.05	0.22	1.00	0.22	99.3	
	40–49	0.89	(0.77,0.95)	0.05	0.21	1.00	0.20	99.2	
	50–59	0.91	(0.80,0.97)	0.04	0.20	1.00	0.18	99.3	
	60–69	0.92	(0.79,0.97)	0.05	0.19	1.00	0.19	99.4	
	70–79	0.91	(0.79,0.97)	0.04	0.17	1.00	0.20	99.4	
	80 or olderǂ	0.98	(0.91,1.00)	0.02	0.23	1.00	0.07	99.6	
Gender									< 0.001**
	Male	0.87	(0.73,0.94)	0.05	0.20	1.00	0.24	99.2	
	Female	0.90	(0.80,0.96)	0.04	0.20	1.00	0.18	99.1	
	Otherǂ	0.81	(0.41,0.96)	0.14	0.00	1.00	0.60	99.8	
Marital status									< 0.001**
	Married	0.91	(0.80,0.96)	0.04	0.19	1.00	0.18	99.2	
	Separated	0.86	(0.74,0.93)	0.05	0.35	1.00	0.22	99.0	
	Divorced	0.90	(0.76,0.96)	0.05	0.20	1.00	0.22	99.4	
	Widowed	0.92	(0.81,0.97)	0.04	0.19	1.00	0.17	99.3	
	Domestic partner	0.86	(0.65,0.95)	0.08	0.27	1.00	0.33	99.6	
	Single, never married	0.85	(0.72,0.93)	0.05	0.22	1.00	0.24	99.1	
Employment status									< 0.001**
	Employed for an employer	0.89	(0.76,0.95)	0.05	0.21	1.00	0.22	99.3	
	Self-employed	0.88	(0.77,0.94)	0.04	0.25	1.00	0.20	99.0	
	Retired	0.91	(0.79,0.96)	0.04	0.18	1.00	0.19	99.3	
	Student	0.87	(0.70,0.95)	0.06	0.24	1.00	0.27	99.4	
	Homemaker	0.92	(0.82,0.96)	0.04	0.19	1.00	0.16	99.1	
	Unemployed and looking for a job	0.88	(0.75,0.94)	0.05	0.19	1.00	0.22	99.2	
	None of these/other	0.86	(0.73,0.94)	0.05	0.16	1.00	0.24	99.2	
Education									< 0.001**
	Up to 8 years	0.91	(0.82,0.96)	0.04	0.19	1.00	0.16	98.9	
	9–15 years	0.89	(0.76,0.95)	0.05	0.19	1.00	0.21	99.2	
	16 + years	0.89	(0.75,0.95)	0.05	0.25	1.00	0.23	99.3	
Religious service attendance									< 0.001**
	> 1/week	0.98	(0.97,0.99)	0.01	0.57	1.00	0.03	97.5	
	1/week	0.97	(0.95,0.98)	0.01	0.69	1.00	0.04	96.5	
	1–3/month	0.95	(0.91,0.97)	0.01	0.51	1.00	0.07	97.7	
	A few times a year	0.90	(0.82,0.95)	0.03	0.30	1.00	0.15	98.8	
	Never	0.70	(0.51,0.84)	0.08	0.15	1.00	0.39	99.2	
Immigration status									< 0.001**
	Born in this country	0.88	(0.76,0.95)	0.05	0.20	1.00	0.21	99.2	
	Born in another country	0.92	(0.78,0.97)	0.05	0.28	1.00	0.20	99.5	

*Note. N* = 202,898. *p < .05; **p< .007 (Bonferroni corrected threshold); ǂGroup is very small (< 0.1% of the observed sample) within several countries, leading to large uncertainty in this estimate—be cautious about interpreting this estimate^1^; SE Analogue = CI Width/4; CI = confidence interval; SE = standard error; LL = lower limits of prediction interval; UL = upper limit of prediction interval; prediction interval is the range of likely values of the effect for a randomly selected country; τ is the standard deviation of the distribution of proportions across countries, which is an indicator of cross-national heterogeneity and is based on the back-transformed$\:\tau\:$for estimated on the logit scale;$\:{I}^{2}$is an estimate of the proportion of variability in means due to heterogeneity across countries vs. sampling variability; the Global *p*-value corresponds to a test of the null hypothesis that there are no differences between the groups for that sociodemographic characteristic in all 22 countries or territories. Additional details of the heterogeneity of estimates are available in the forest plots in our online supplement.

## Discussion

This study investigated the proportion of belief in God, gods, and spiritual forces vs. non-belief and uncertainty using a sample of 202,898 individuals from 22 countries and explored how this belief (or non-belief) varies across different demographic categories. The proportion of belief ranged from 20% in Japan to 100% in Egypt. Nearly half of the countries (10 of 22) included in wave 1 of the GFS have a reported proportion of the population with a belief in God exceeding 90%. Five of those ten countries are in Africa (Egypt, Kenya, Nigeria, South Africa, Tanzania). This continent’s high proportion of belief in God, gods, and spiritual forces might reflect social and ecological influences. In these countries, the cost associated with non-belief can be significant, ranging from social disapproval to severe structural and legal consequences. Religious commitment is generally high in Muslim-majority countries. The study’s results reflected this for Egypt (100%; variability was non-existent in the demographic groups) and Indonesia (97%). However, in Turkey (85%), another Muslim-majority country, belief was relatively lower.

On the lower end of the spectrum for Belief in God are countries such as Australia (54%), the United Kingdom (54%), Sweden (44%), and Japan (20%). The decline in religious commitment in countries with prolonged economic and social prosperity represents one of the most significant developments in recent human history and has been the subject of intense scholarly debate. One leading explanation is that the rise of modern science beginning in the seventeenth century—and, still more, the ‘mechanistic’ metaphysics by which those developments have frequently been interpreted—have made theism seem less plausible to many^48^. More ‘materialist’ factors might also have played a role. Notably, the rise in material prosperity undermines individuals’ felt need for the social support and spiritual consolation offered by religious belief^12^.

### Demographic variation in belief in God

Across all countries, Belief in God was highest among individuals who are 70 years or older (98%), female (90%), widowed or married (92%), homemakers or retired (92%), attending religious services less than once a week (98%), with lower levels of education (91%), and who are born in a country other than where they are presently living (i.e., immigrants; 92%). These findings are consistent with prior work that showed higher levels of religiousness for older people^20^, women^23^, individuals with low education^27^, and immigrants^28^.

### Country-specific variation in belief in God across demographic groups

Despite the global trends, demographic variations in belief in God were inconsistent across all 22 countries. We summarize key differences (see details in the data-rich online supplement).

**Age**. Although the proportion of Belief in God was generally highest among individuals 80 years or older globally, this belief was not consistently higher for older individuals compared to younger ones. For example, in Brazil, Kenya, and Tanzania, young adults were similarly likely to report belief in God, gods, or spiritual forces compared with older adults; in Hong Kong and Indonesia, young adults were significantly more likely than older adults to report Belief in God; in Sweden, we observed a U-shaped curve with young and older adults both having significantly higher proportions of believers compared with middle-aged adults; and, in Japan, non-belief was consistent across all age groups.

**Gender.** Women (and, notably, homemakers) had a higher proportion of Belief in God relative to males in most countries. Notably, gender differences were pronounced in countries such as Argentina, Poland, and the United States, where atheists presumably face less disapproval or mistreatment. The measure used in the Global Flourishing Study might also have contributed to the observed similarities or marginal differences in highly religious countries.

**Marital status.** The proportion of believers was almost consistently higher among married individuals. This is understandable from a functionalist perspective. All religious groups stress the importance of marriage and direct marital choice and relationships. Further, religious dictates typically support continued parental investment and mate retention once offspring are produced. Thus, never-married (or not yet-married) individuals may have rejected these religious norms, perhaps seeking fewer restrictions in a free society, or may have felt less drawn towards or need of the religious norms themselves. Although the number of widows and widowers in each country was typically less than 10% of the total sample, there were interesting differences across countries. Widows in some countries had a higher proportion of belief than married individuals (e.g., Poland, South Africa, Spain, U.S.). In contrast, widows and widowers had a significantly smaller proportion of believers than marrieds in other countries (e.g., India). More research seems warranted regarding the religious experiences of marital survivors.

**Employment and education. **In the pooled meta-analysis, there was relatively little variation in belief by education (e.g., 89% for those with 16 + years of education vs. 91% for those up to 8 years). Individuals with fewer than nine years of education reported the highest proportion of belief in God in most countries, with a few exceptions (e.g., Indonesia, Japan, Philippines, and the UK). However, in countries that had the highest proportions of belief in God, there were either no differences in educational attainment (e.g., Egypt and Tanzania) or differences that were not statistically significant (e.g., Nigeria and Kenya). One interpretation is that socialization in highly religious cultures might increase the value of theism^49^ thereby mitigating education differences. Additionally, the cost of non-belief in some countries is severe, making it more likely for individuals to report believing in God even if they do not. Reports of legally sanctioned mistreatment of atheists and individuals accused of blasphemy are not uncommon in Egypt and Nigeria, for example.

**Religious service attendance. **Belief in God is more basic—and even personal—compared with other measures of religiosity used in prior studies, such as religious importance or religious service attendance, for which significant differences have been observed^25^. However, in the present study, we found that the proportion of belief was just as high among frequent religious service attenders and individuals who rarely attend services in countries with a high proportion of believers (e.g., Brazil, Indonesia, Kenya, and Nigeria). This finding suggests that the frequency of service attendance may not adequately capture the level of religious convictions in countries where theism is the norm. More generally, religious belief and practice are not always congruent. For example, it is not uncommon for individuals who identify as atheists who lack belief in God to attend religious service for various reasons, including an appreciation for the music, accompanying a spouse, engaging with the community, or attending religious ceremonies^10,50^. Even for religious individuals, reports of low religious service attendance are common^10^.

**Immigration status.** There were other considerable country-specific variations in belief in God based on immigration status. Individuals born outside the receiving country reported higher levels of belief in God in mostly European countries (Germany, Spain, Sweden, and the United Kingdom). These findings align with those from past research but diverge for countries with a substantial immigrant population (i.e., greater than 2%), such as Israel, Mexico, South Africa, and the U.S., where a significantly higher proportion of natives reported Belief in God. Data collected in the upcoming years as part of the Global Flourishing Study will help further our understanding of these patterns. One noteworthy observation is that in European countries where immigrants had a higher proportion of belief in God, the proportion of theists was generally lower compared to countries where natives had the highest belief in God. Countries with a considerable sample size of immigrants yet showed non-significant differences include Argentina and Hong Kong.

### Demographic variation by religious affiliation

In many countries, especially those in the top half of the list (e.g., Nigeria, Kenya, and Indonesia), there were no significant differences in Belief in God based on religious affiliation. In contrast, differences were observed in many countries in the bottom half of the list (e.g., Spain, Germany, Hong Kong, and the United Kingdom). In most of these countries, Christians constituted the majority and Muslims the minority. Belief in God was higher among Muslims compared to Christians, based on the observed proportions. For example, in Spain, 79% of Christians and 85% of Muslims expressed Belief in God; in Germany, 67% of Christians and 83% of Muslims; and in the United Kingdom, 72% of Christians and 98% of Muslims. These findings align with previous studies showing that Muslims are religiously distinctive in secular countries and report more conservative social and moral attitudes^51^. Generally, Islam is perceived as being less prone to secularization, strictly maintaining its religious practices and values, compared to Christianity^52^. This strictness may contribute to relatively higher levels of religious commitment among Muslims in these countries^53^.

In Japan, 37% of the sample reported being affiliated either with Buddhism (33%), Shinto (2.3%), or Christianity (1.9%). Belief in God was higher among individuals affiliated with Christianity (77%) compared to those affiliated with Shinto (47%) and Buddhism (25%). These differences may be attributed to the varying emphasis on Belief in God within these religions. Buddhism typically does not focus on the belief in a god or gods to the same extent as Christianity or Shinto. Additionally, historical events may have influenced these beliefs. Japan’s Buddhist and Shinto communities experienced significant trauma during the nineteenth and twentieth centuries. This period saw the implementation of the *shinbutsu bunri rei*, an edict that mandated the separation of Shinto from Buddhism, and the *haibutsu kishaku*movement, which aimed to abolish Buddhism and destroy Buddhist temples and images, particularly during the early Meiji period (1868–1912). Following this separation, the establishment of ‘State Shinto’ severely impacted traditional Japanese Buddhism and closely associated Shinto with imperial expansion and the deified emperor. Japan’s defeat in World War II further discredited its imperial ambitions, affecting Shinto’s public perception. The 1945 Shinto Directive by the American occupiers quickly removed public support and financial backing for Shinto. Hardacre^54^ notes that citizens rapidly adopted the directive to resist ongoing pressures to support Shinto shrines financially.

### Limitations and future directions

This study has several limitations. Different categories of responses to the question about belief in God were combined and then dichotomized. While this decision made the analyses easier to conduct, it may have obscured some important nuances. For instance, individuals who indicated belief in an impersonal spiritual force may differ in key ways from those who believe in one or more gods. Belief in an impersonal spiritual force likely reflects a more abstract view, whereas belief in one or more gods may imply a more personal and anthropomorphic concept. These differences in levels of abstraction could have implications for demographic, between-country, and country-specific variations in these beliefs. Relatedly, the non-belief category combined responses from individuals who chose “none of the above” and “unsure.” The aggregation ignores potential differences among the categories, which may include positive atheists (strong atheists), negative atheists (weak atheists), agnostic atheists, and agnostic theists.

The measurement of Belief in God as a categorical distinction may have influenced the observed results. Assessing belief in God as a continuum of belief strength rather than a categorical distinction might have led to different conclusions, potentially introducing more variations in responses. For example, Egypt’s lack of demographic variation might have been less pronounced if belief had been assessed as a continuum. Additionally, although belief and non-belief are inherently messy categories, some important nuance is lost by aggregating and dichotomizing the five possible responses to the Belief in God item.

Finally, we caution against overinterpretation of the data presented here. For example, care should be taken when interpreting the ordered means. Differences across countries can certainly arise from different life experiences but may also be a function of translation, a participant’s interpretation of the items, or how participants in different countries respond to scaled items. For example, participants may tend to choose the middle, rather than the extremes, on a Likert. The comparisons across demographic groups (Table 3) or comparisons within countries (Tables S1b-S22b) may thus be more reasonable resources for drawing conclusions. Additionally, it is important to remember that these are merely descriptive statistics of which groups score higher or lower on a relevant construct. The results do not imply causality.

The Global Flourishing Study assesses key demographic variables often associated with religion and spirituality. However, future researchers might want to evaluate cross-cultural differences for factors such as quality of childhood education, family relationships, social networks, or being in the country’s majority vs. minority religious group. Other research might consider how these and other childhood demographics might influence belief in God, gods, and spiritual forces in later life. The results of this study might also shed light on the collective beliefs that contribute to a nation’s cultural values or political priorities.

## Conclusion

Overall, assessing belief in God, gods, and spiritual forces vs. non-belief and uncertainty in the Global Flourishing Study (GFS) supports previous research and can enhance our understanding of global religious trends, including the importance of demographic characteristics related to theism and non-belief, both around the world and within countries. We look forward to tracking changes in these variables using later waves of the GFS.

## Electronic supplementary material

Below is the link to the electronic supplementary material.

Supplementary Material 1

## Data Availability

The data are publicly available through the Center for Open Science (https://www.cos.io/gfs).
