# Supplementary material for: A cross-national analysis of demographic variation in belief in God, gods, or spiritual forces in 22 countries
Source: Sci Rep. 2025 Apr 30;15:13302. doi: 10.1038/s41598-024-79103-w (PMC12043916; doi:10.1038/s41598-024-79103-w)
Supplement: Supplementary file 1 — Supplementary Material 1 [file 41598_2024_79103_MOESM1_ESM.pdf]

A Cross-National Analysis of Demographic Variation in Belief in God, Gods, or Spiritual Forces in 22 Countries

Eric Y. Aglozo[a]
eaglozo@asu.edu

Kathryn A. Johnson [a]*
Kathryn.a.johnson@asu.edu

Brendan Case [b]
brendan_case@fas.harvard.edu

R. Noah Padgett [b]
npadgett@hsph.harvard.edu

Byron R. Johnson [c] **
Byron_Johnson@baylor.edu

Tyler J. VanderWeele [d] **
tvanderw@hsph.harvard.edu

***Corresponding Author: Kathryn A. Johnson; Kathryn.a.johnson@asu.edu

**Authors contributed equally as senior authors

[a] Psychology Department, Arizona State University, Tempe, AZ, USA

[b] Human Flourishing Program, Institute for Quantitative Social Science, Harvard University, Cambridge, MA, USA

[c] Department of Economics, Baylor University, Waco, TX, USA

[d] Department of Biostatistics, Harvard T.H. Chan School of Public Health, Boston, MA, USA

This submission is part of the Global Flourishing Study collection. We were invited to submit this manuscript following a peer review offer by the Chief Editor.

A Cross-National Analysis of Demographic Variation in Belief in God, Gods, or Spiritual Forces in 22 Countries

Supplemental Materials

Supplementary Tables S1a-S22a provide the nationally representative descriptive statistics for each demographic category for each country separately. The countries are ordered alphabetically.

Supplementary Tables S1b-S22b provide the proportions of Belief in God, gods, and spiritual forces for each of the demographic categories for each country separately. The countries are ordered alphabetically, with proportion tables following the demographics tables. Global p-values are provided. The tables use multiple imputations to handle missing data.

Supplementary Table S23 provides an alternative meta-analysis wherein instead of treating each country similarly (as in a random effects meta-analysis), each country's results are weighted by the 2023 population size. Note this is the country's actual population (not the study sample size). The random effects meta-analysis effectively treats each of the 22 countries equally. The population-weighted meta-analysis effectively treats each person in the 22 countries equally.

Forest Plots S1-S115 show the proportion of Belief in God, gods, or spiritual forces by country for each demographic variable.

Table S1a. Nationally representative descriptive statistics for Argentina

Characteristic	N = 6,724[1]
**Age group**	
18-24	1,108 (16%)
25-29	719 (11%)
30-39	1,432 (21%)
40-49	1,254 (19%)
50-59	1,014 (15%)
60-69	730 (11%)
70-79	356 (5.3%)
80 or older	112 (1.7%)
(Missing)	0 (0%)
**Gender**	
Male	3,143 (47%)
Female	3,542 (53%)
Other	21 (0.3%)
(Missing)	18 (0.3%)
**Marital status**	
Married	1,565 (23%)
Separated	455 (6.8%)
Divorced	321 (4.8%)
Widowed	401 (6.0%)
Never	2,381 (35%)
Domestic Partner	1,514 (23%)
(Missing)	88 (1.3%)
**Employment**	
Employed for an employer	2,440 (36%)
Self-employed	1,748 (26%)
Retired	773 (11%)
Student	354 (5.3%)
Homemaker	639 (9.5%)
Unemployed and looking for a job	569 (8.5%)
None of these/Other	179 (2.7%)
(Missing)	22 (0.3%)
**Religious service attendance**	
At least 1/week	532 (7.9%)
1/week	773 (12%)
1-3/month	461 (6.8%)
A few times a year	1,949 (29%)
Never	2,982 (44%)
(Missing)	27 (0.4%)
**Education**	
Up to 8 years	2,263 (34%)

**Characteristic**	**N = 6,724**[1]
9-15 years	3,823 (57%)
16+ years	635 (9.4%)
(Missing)	3 (<0.1%)
**Immigration**	
Born in this country	6,346 (94%)
Born in another country	348 (5.2%)
(Missing)	29 (0.4%)
**Religious affiliation**	
Christianity	4,992 (74%)
Islam	9 (0.1%)
Hinduism	6 (<0.1%)
Buddhism	35 (0.5%)
Judaism	40 (0.6%)
Sikhism	0 (<0.1%)
Baha'i	0 (0%)
Jainism	0 (0%)
Shinto	0 (0%)
Taoism	2 (<0.1%)
Confucianism	0 (<0.1%)
Primal, Animist, or Folk religion	19 (0.3%)
Spiritism	0 (0%)
Umbanda, Candomble, and other African-derived religions	0 (0%)
Chinese folk/traditional religion	0 (0%)
Some other religion	156 (2.3%)
No religion/Atheist/Agnostic	1,352 (20%)
(Missing)	111 (1.7%)
**Race/Ethnicity**	
Asian	43 (0.6%)
Black	95 (1.4%)
Indigenous	129 (1.9%)
Mestizo(a)	1,801 (27%)
Mullato(a)	75 (1.1%)
Other	104 (1.5%)
White	3,406 (51%)
(Missing)	1,070 (16%)

[1]n (%)

Table S1b. Proportions by demographic category for Argentina

Variable	Category	Mean	95% CI	SE	Global p-value
Age group	18-24	0.73	(0.70, 0.77)	0.02	< .001
	25-29	0.81	(0.77, 0.85)	0.02	
	30-39	0.86	(0.83, 0.88)	0.01	
	40-49	0.88	(0.85, 0.90)	0.01	
	50-59	0.88	(0.86, 0.91)	0.01	
	60-69	0.89	(0.86, 0.92)	0.01	
	70-79	0.85	(0.79, 0.91)	0.03	
	80 or older	0.96	(0.92, 0.99)	0.02	
Gender	Male	0.80	(0.78, 0.82)	0.01	< .001
	Female	0.88	(0.87, 0.90)	0.01	
	Other	0.65	(0.37, 0.93)	0.13	
Marital status	Married	0.90	(0.88, 0.92)	0.01	< .001
	Separated	0.86	(0.82, 0.91)	0.02	
	Divorced	0.86	(0.81, 0.91)	0.02	
	Widowed	0.93	(0.89, 0.97)	0.02	
	Never	0.80	(0.77, 0.82)	0.01	
	Domestic Partner	0.84	(0.81, 0.86)	0.01	
Employment	Employed for an employer	0.82	(0.80, 0.84)	0.01	< .001
	Self-employed	0.83	(0.81, 0.86)	0.01	
	Retired	0.89	(0.86, 0.92)	0.02	
	Student	0.73	(0.67, 0.79)	0.03	
	Homemaker	0.94	(0.91, 0.97)	0.01	
	Unemployed and looking for a job	0.88	(0.85, 0.92)	0.02	
	None of these/Other	0.83	(0.76, 0.90)	0.03	
Religious service attendance	At least 1/week	0.98	(0.96, 1.00)	0.01	< .001
	1/week	0.96	(0.94, 0.98)	0.01	
	1-3/month	0.95	(0.92, 0.98)	0.02	
	A few times a year	0.94	(0.92, 0.95)	0.01	
	Never	0.71	(0.69, 0.74)	0.01	
Education	Up to 8 years	0.90	(0.87, 0.92)	0.01	< .001
	9-15 years	0.83	(0.82, 0.84)	0.01	
	16+ years	0.75	(0.71, 0.79)	0.02	
Immigration status	Born in this country	0.84	(0.83, 0.86)	0.01	0.915

Variable	Category	Mean	95% CI	SE	Global p-value
	Born in another country	0.85	(0.79, 0.90)	0.03	
Religious affiliation	Christianity	0.93	(0.92, 0.94)	0.01	< .001
	Islam	0.93	(0.74, 1.00)	0.07	
	Hinduism	0.70	*	*	
	Buddhism	0.87	(0.75, 0.99)	0.06	
	Judaism	0.79	(0.60, 0.98)	0.09	
	Sikhism	1.00	*	*	
	Taoism	0.40	*	*	
	Confucianism	0.00	*	*	
	Primal, Animist, or Folk religion	1.00	(1.00, 1.00)	0.00	
	Some other religion	0.91	(0.84, 0.98)	0.03	
	No religion/Atheist/Agnostic	0.53	(0.49, 0.57)	0.02	
Race/ethnicity	Asian	0.90	(0.75, 1.00)	0.07	0.475
	Black	0.78	(0.64, 0.91)	0.07	
	Indigenous	0.88	(0.80, 0.97)	0.04	
	Mestizo(a)	0.85	(0.83, 0.88)	0.01	
	Mullato(a)	0.81	(0.70, 0.93)	0.06	
	White	0.84	(0.83, 0.86)	0.01	
	Other	0.81	(0.70, 0.93)	0.06	

Table S2a. Nationally representative descriptive statistics for Australia

Characteristic	N = 3,844[1]
**Age group**	
18-24	345 (9.0%)
25-29	282 (7.3%)
30-39	641 (17%)
40-49	618 (16%)
50-59	691 (18%)
60-69	589 (15%)
70-79	498 (13%)
80 or older	178 (4.6%)
(Missing)	2 (<0.1%)
**Gender**	
Male	1,861 (48%)
Female	1,941 (50%)
Other	36 (0.9%)
(Missing)	6 (0.2%)
**Marital status**	
Married	1,797 (47%)
Separated	158 (4.1%)
Divorced	332 (8.6%)
Widowed	215 (5.6%)
Never	855 (22%)
Domestic Partner	450 (12%)
(Missing)	38 (1.0%)
**Employment**	
Employed for an employer	1,881 (49%)
Self-employed	380 (9.9%)
Retired	912 (24%)
Student	190 (5.0%)
Homemaker	137 (3.6%)
Unemployed and looking for a job	134 (3.5%)
None of these/Other	206 (5.4%)
(Missing)	4 (0.1%)
**Religious service attendance**	
At least 1/week	162 (4.2%)
1/week	299 (7.8%)
1-3/month	135 (3.5%)
A few times a year	656 (17%)
Never	2,584 (67%)
(Missing)	7 (0.2%)
**Education**	
Up to 8 years	70 (1.8%)

Characteristic	N = 3,844[1]
9-15 years	2,434 (63%)
16+ years	1,330 (35%)
(Missing)	10 (0.3%)
**Immigration**	
Born in this country	2,953 (77%)
Born in another country	885 (23%)
(Missing)	6 (0.2%)
**Religious affiliation**	
Christianity	1,592 (41%)
Islam	45 (1.2%)
Hinduism	31 (0.8%)
Buddhism	36 (0.9%)
Judaism	26 (0.7%)
Sikhism	8 (0.2%)
Baha'i	7 (0.2%)
Jainism	0 (0%)
Shinto	0 (0%)
Taoism	5 (0.1%)
Confucianism	0 (0%)
Primal, Animist, or Folk religion	23 (0.6%)
Spiritism	0 (0%)
Umbanda, Candomble, and other African-derived religions	0 (0%)
Chinese folk/traditional religion	0 (0%)
Some other religion	39 (1.0%)
No religion/Atheist/Agnostic	2,020 (53%)
(Missing)	15 (0.4%)
**Race/Ethnicity**	
Aboriginal	53 (1.4%)
Australian	1,946 (51%)
Australian, British/European	1,047 (27%)
Chinese	75 (1.9%)
Indian	58 (1.5%)
Japanese	1 (<0.1%)
Malay	11 (0.3%)
New Zealander	91 (2.4%)
Other	163 (4.2%)
Other European	357 (9.3%)
Russian	7 (0.2%)
Samoan	4 (0.1%)
Sinhalese	1 (<0.1%)
Spanish	2 (<0.1%)
Sri Lankan Moor	1 (<0.1%)
Sri Lankan Tamil	7 (0.2%)

**Characteristic**	**N = 3,844**[1]
Vietnamese	7 (0.2%)
(Missing)	14 (0.4%)

[1]n (%)

Table S2b. Proportions by demographic category for Australia

Variable	Category	Mean	95% CI	SE	Global p-value
Age group	18-24	0.46	(0.38, 0.54)	0.04	< .001
	25-29	0.43	(0.34, 0.51)	0.04	
	30-39	0.45	(0.40, 0.51)	0.03	
	40-49	0.55	(0.50, 0.60)	0.03	
	50-59	0.55	(0.51, 0.59)	0.02	
	60-69	0.57	(0.53, 0.61)	0.02	
	70-79	0.63	(0.58, 0.67)	0.02	
	80 or older	0.72	(0.65, 0.79)	0.04	
Gender	Male	0.46	(0.43, 0.49)	0.01	< .001
	Female	0.61	(0.58, 0.64)	0.01	
	Other	0.58	(0.34, 0.82)	0.12	
Marital status	Married	0.56	(0.54, 0.59)	0.01	< .001
	Separated	0.56	(0.46, 0.66)	0.05	
	Divorced	0.64	(0.58, 0.70)	0.03	
	Widowed	0.70	(0.63, 0.77)	0.04	
	Never	0.48	(0.44, 0.53)	0.02	
	Domestic Partner	0.38	(0.32, 0.44)	0.03	
Employment	Employed for an employer	0.49	(0.46, 0.52)	0.01	< .001
	Self-employed	0.53	(0.47, 0.59)	0.03	
	Retired	0.61	(0.58, 0.65)	0.02	
	Student	0.40	(0.29, 0.50)	0.05	
	Homemaker	0.65	(0.54, 0.76)	0.06	
	Unemployed and looking for a job	0.58	(0.46, 0.70)	0.06	
	None of these/Other	0.64	(0.55, 0.73)	0.05	
Religious service attendance	At least 1/week	1.00	(0.99, 1.00)	0.00	< .001
	1/week	0.97	(0.95, 0.99)	0.01	
	1-3/month	0.89	(0.82, 0.96)	0.04	
	A few times a year	0.76	(0.73, 0.80)	0.02	
	Never	0.38	(0.36, 0.41)	0.01	
Education	Up to 8 years	0.63	(0.45, 0.80)	0.09	0.018
	9-15 years	0.55	(0.53, 0.58)	0.01	
	16+ years	0.50	(0.47, 0.53)	0.01	
Immigration status	Born in this country	0.52	(0.50, 0.54)	0.01	< .001

Variable	Category	Mean	95% CI	SE	Global p-value
	Born in another country	0.60	(0.56, 0.63)	0.02	
Religious affiliation	Christianity	0.81	(0.79, 0.84)	0.01	< .001
	Islam	0.95	(0.84, 1.00)	0.05	
	Hinduism	0.87	(0.70, 1.00)	0.08	
	Buddhism	0.66	(0.49, 0.84)	0.08	
	Judaism	0.67	(0.44, 0.89)	0.10	
	Sikhism	0.87	*	*	
	Baha'i	0.98	*	*	
	Taoism	1.00	*	*	
	Primal, Animist, or Folk religion	0.97	(0.90, 1.00)	0.03	
	Some other religion	0.62	(0.40, 0.85)	0.11	
	No religion/Atheist/Agnostic	0.29	(0.26, 0.32)	0.01	
Race/ethnicity	Aboriginal	0.74	(0.56, 0.92)	0.09	< .001
	Australian	0.54	(0.51, 0.57)	0.01	
	Australian British/European	0.48	(0.44, 0.52)	0.02	
	Chinese	0.40	(0.25, 0.56)	0.08	
	Indian	0.91	(0.84, 0.98)	0.03	
	Japanese	0.54	*	*	
	Malay	0.73	*	*	
	Sinhalese	0.36	*	*	
	Spanish	0.54	*	*	
	Sri Lankan Moor	0.61	*	*	
	Sri Lankan Tamil	0.64	*	*	
	Vietnamese	0.44	*	*	
	Russian	0.88	*	*	
	Samoan	1.00	*	*	
	New Zealander	0.56	(0.42, 0.70)	0.07	
	Other European	0.56	(0.50, 0.62)	0.03	
	Other	0.64	(0.54, 0.74)	0.05	

Table S3a. Nationally representative descriptive statistics for Brazil

Characteristic	N = 13,204[1]
**Age group**	
18-24	1,986 (15%)
25-29	1,468 (11%)
30-39	2,908 (22%)
40-49	2,638 (20%)
50-59	2,131 (16%)
60-69	1,435 (11%)
70-79	510 (3.9%)
80 or older	126 (1.0%)
(Missing)	0 (0%)
**Gender**	
Male	6,320 (48%)
Female	6,820 (52%)
Other	35 (0.3%)
(Missing)	30 (0.2%)
**Marital status**	
Married	4,646 (35%)
Separated	594 (4.5%)
Divorced	865 (6.5%)
Widowed	408 (3.1%)
Never	4,347 (33%)
Domestic Partner	2,081 (16%)
(Missing)	263 (2.0%)
**Employment**	
Employed for an employer	3,756 (28%)
Self-employed	2,918 (22%)
Retired	1,536 (12%)
Student	624 (4.7%)
Homemaker	1,305 (9.9%)
Unemployed and looking for a job	2,419 (18%)
None of these/Other	448 (3.4%)
(Missing)	199 (1.5%)
**Religious service attendance**	
At least 1/week	2,386 (18%)
1/week	2,272 (17%)
1-3/month	1,398 (11%)
A few times a year	3,978 (30%)
Never	3,110 (24%)
(Missing)	61 (0.5%)
**Education**	
Up to 8 years	3,139 (24%)

Characteristic	N = 13,204[1]
9-15 years	7,665 (58%)
16+ years	2,390 (18%)
(Missing)	10 (<0.1%)
**Immigration**	
Born in this country	12,688 (96%)
Born in another country	153 (1.2%)
(Missing)	363 (2.7%)
**Religious affiliation**	
Christianity	9,911 (75%)
Islam	6 (<0.1%)
Hinduism	1 (<0.1%)
Buddhism	37 (0.3%)
Judaism	31 (0.2%)
Sikhism	0 (0%)
Baha'i	2 (<0.1%)
Jainism	2 (<0.1%)
Shinto	1 (<0.1%)
Taoism	1 (<0.1%)
Confucianism	6 (<0.1%)
Primal, Animist, or Folk religion	15 (0.1%)
Spiritism	696 (5.3%)
Umbanda, Candomble, and other African-derived religions	525 (4.0%)
Chinese folk/traditional religion	0 (0%)
Some other religion	144 (1.1%)
No religion/Atheist/Agnostic	1,712 (13%)
(Missing)	113 (0.9%)
**Race/Ethnicity**	
Amarela	238 (1.8%)
Branca	5,169 (39%)
Indígena	131 (1.0%)
Other	61 (0.5%)
Parda	5,125 (39%)
Preta	1,615 (12%)
(Missing)	865 (6.6%)

[1]n (%)

Table S3b. Proportions by demographic category for Brazil

Variable	Category	Mean	95% CI	SE	Global p-value
Age group	18-24	0.92	(0.91, 0.94)	0.01	< .001
	25-29	0.96	(0.94, 0.97)	0.01	
	30-39	0.96	(0.95, 0.97)	0.00	
	40-49	0.97	(0.96, 0.98)	0.00	
	50-59	0.98	(0.97, 0.99)	0.00	
	60-69	0.96	(0.95, 0.98)	0.01	
	70-79	0.95	(0.92, 0.99)	0.02	
	80 or older	0.99	(0.97, 1.00)	0.01	
Gender	Male	0.94	(0.93, 0.95)	0.00	< .001
	Female	0.98	(0.97, 0.98)	0.00	
	Other	0.96	(0.90, 1.00)	0.03	
Marital status	Married	0.97	(0.97, 0.98)	0.00	< .001
	Separated	0.97	(0.94, 0.99)	0.01	
	Divorced	0.96	(0.94, 0.98)	0.01	
	Widowed	0.98	(0.96, 0.99)	0.01	
	Never	0.94	(0.93, 0.95)	0.00	
	Domestic Partner	0.96	(0.95, 0.97)	0.01	
Employment	Employed for an employer	0.96	(0.96, 0.97)	0.00	0.001
	Self-employed	0.96	(0.95, 0.97)	0.01	
	Retired	0.96	(0.95, 0.98)	0.01	
	Student	0.91	(0.89, 0.94)	0.01	
	Homemaker	0.98	(0.96, 0.99)	0.01	
	Unemployed and looking for a job	0.96	(0.95, 0.97)	0.01	
	None of these/Other	0.94	(0.91, 0.97)	0.01	
Religious service attendance	At least 1/week	0.99	(0.98, 1.00)	0.00	< .001
	1/week	0.98	(0.98, 0.99)	0.00	
	1-3/month	0.99	(0.98, 0.99)	0.00	
	A few times a year	0.98	(0.98, 0.99)	0.00	
	Never	0.88	(0.86, 0.89)	0.01	
Education	Up to 8 years	0.97	(0.96, 0.98)	0.00	0.009
	9-15 years	0.96	(0.95, 0.97)	0.00	
	16+ years	0.95	(0.94, 0.96)	0.01	
Immigration status	Born in this country	0.96	(0.96, 0.97)	0.00	0.027

Variable	Category	Mean	95% CI	SE	Global p-value
	Born in another country	0.87	(0.79, 0.95)	0.04	
Religious affiliation	Christianity	0.98	(0.98, 0.99)	0.00	1.000
	Islam	0.78	*	*	
	Hinduism	1.00	*	*	
	Buddhism	0.93	(0.84, 1.00)	0.04	
	Judaism	0.98	(0.94, 1.00)	0.02	
	Baha'i	1.00	*	*	
	Jainism	1.00	*	*	
	Shinto	1.00	*	*	
	Taoism	1.00	*	*	
	Confucianism	1.00	*	*	
	Primal, Animist, or Folk religion	0.78	*	*	
	Spiritism	1.00	(0.99, 1.00)	0.00	
	Umbanda, Candomble, and other African-derived religions	0.97	(0.95, 0.99)	0.01	
	Some other religion	0.96	(0.93, 0.99)	0.02	
	No religion/Atheist/Agnostic	0.81	(0.78, 0.83)	0.01	
Race/ethnicity	Branca	0.95	(0.94, 0.96)	0.00	< .001
	Preta	0.97	(0.95, 0.98)	0.01	
	Parda	0.97	(0.97, 0.98)	0.00	
	Amarela	0.95	(0.92, 0.98)	0.02	
	Indígena	0.93	(0.88, 0.99)	0.03	
	Other	0.93	(0.87, 1.00)	0.03	

Table S4a. Nationally representative descriptive statistics for Egypt

Characteristic	N = 4,729[1]
**Age group**	
18-24	960 (20%)
25-29	607 (13%)
30-39	1,204 (25%)
40-49	897 (19%)
50-59	613 (13%)
60-69	387 (8.2%)
70-79	54 (1.1%)
80 or older	7 (0.2%)
(Missing)	0 (0%)
**Gender**	
Male	2,394 (51%)
Female	2,334 (49%)
Other	0 (0%)
(Missing)	0 (<0.1%)
**Marital status**	
Married	3,387 (72%)
Separated	39 (0.8%)
Divorced	101 (2.1%)
Widowed	238 (5.0%)
Never	947 (20%)
Domestic Partner	0 (0%)
(Missing)	17 (0.4%)
**Employment**	
Employed for an employer	1,267 (27%)
Self-employed	892 (19%)
Retired	253 (5.4%)
Student	297 (6.3%)
Homemaker	1,772 (37%)
Unemployed and looking for a job	224 (4.7%)
None of these/Other	21 (0.4%)
(Missing)	3 (<0.1%)
**Religious service attendance**	
At least 1/week	839 (18%)
1/week	960 (20%)
1-3/month	368 (7.8%)
A few times a year	458 (9.7%)
Never	2,091 (44%)
(Missing)	12 (0.3%)
**Education**	
Up to 8 years	2,486 (53%)

**Characteristic**	**N = 4,729**[1]
9-15 years	1,599 (34%)
16+ years	643 (14%)
(Missing)	1 (<0.1%)
**Immigration**	
Born in this country	4,713 (100%)
Born in another country	16 (0.3%)
(Missing)	1 (<0.1%)
**Religious affiliation**	
Christianity	120 (2.5%)
Islam	4,607 (97%)
Hinduism	0 (0%)
Buddhism	0 (0%)
Judaism	0 (0%)
Sikhism	0 (0%)
Baha'i	0 (0%)
Jainism	0 (0%)
Shinto	0 (0%)
Taoism	0 (<0.1%)
Confucianism	0 (0%)
Primal, Animist, or Folk religion	0 (0%)
Spiritism	0 (0%)
Umbanda, Candomble, and other African-derived religions	0 (0%)
Chinese folk/traditional religion	0 (0%)
Some other religion	0 (0%)
No religion/Atheist/Agnostic	0 (0%)
(Missing)	1 (<0.1%)
**Race/Ethnicity**	
Arab	4,585 (97%)
Bedouin Arab	4 (<0.1%)
Greek	1 (<0.1%)
Nubian	27 (0.6%)
Turkish	9 (0.2%)
(Missing)	102 (2.2%)

[1]n (%)

Table S4b. Proportions by demographic category for Egypt

Variable	Category	Mean	95% CI	SE	Global p-value
Age group	18-24	1.00	(0.99, 1.00)	0.00	1.000
	25-29	1.00	*	*	
	30-39	1.00	*	*	
	40-49	1.00	*	*	
	50-59	1.00	*	*	
	60-69	1.00	(1.00, 1.00)	0.00	
	70-79	1.00	*	*	
	80 or older	1.00	*	*	
Gender	Male	1.00	*	*	0.319
	Female	1.00	(1.00, 1.00)	0.00	
Marital status	Married	1.00	*	*	1.000
	Separated	1.00	(1.00, 1.00)	0.00	
	Divorced	1.00	*	*	
	Widowed	1.00	*	*	
	Never	1.00	(0.99, 1.00)	0.00	
Employment	Employed for an employer	1.00	*	*	1.000
	Self-employed	1.00	(0.99, 1.00)	0.00	
	Retired	1.00	*	*	
	Student	1.00	*	*	
	Homemaker	1.00	*	*	
	Unemployed and looking for a job	1.00	*	*	
	None of these/Other	1.00	*	*	
Religious service attendance	At least 1/week	1.00	*	*	1.000
	1/week	1.00	*	*	
	1-3/month	1.00	*	*	
	A few times a year	1.00	*	*	
	Never	1.00	(1.00, 1.00)	0.00	
Education	Up to 8 years	1.00	(1.00, 1.00)	0.00	0.999
	9-15 years	1.00	*	*	
	16+ years	1.00	(1.00, 1.00)	0.00	
Immigration status	Born in this country	1.00	(1.00, 1.00)	0.00	0.321
	Born in another country	1.00	*	*	
Religious affiliation	Christianity	1.00	*	*	1.000

Variable	Category	Mean	95% CI	SE	Global p-value
	Islam	1.00	(1.00, 1.00)	0.00	
	Taoism	1.00	*	*	
Race/ethnicity	Arab	1.00	(1.00, 1.00)	0.00	1.000
	Turkish	1.00	*	*	
	Greek	1.00	*	*	
	Bedouin Arab	1.00	*	*	
	Nubian	1.00	*	*	

Table S5a. Nationally representative descriptive statistics for Germany

Characteristic	N = 9,506[1]
**Age group**	
18-24	829 (8.7%)
25-29	774 (8.1%)
30-39	1,438 (15%)
40-49	1,494 (16%)
50-59	1,729 (18%)
60-69	1,915 (20%)
70-79	1,137 (12%)
80 or older	190 (2.0%)
(Missing)	0 (0%)
**Gender**	
Male	4,641 (49%)
Female	4,843 (51%)
Other	11 (0.1%)
(Missing)	11 (0.1%)
**Marital status**	
Married	4,784 (50%)
Separated	219 (2.3%)
Divorced	767 (8.1%)
Widowed	409 (4.3%)
Never	2,627 (28%)
Domestic Partner	619 (6.5%)
(Missing)	81 (0.9%)
**Employment**	
Employed for an employer	4,950 (52%)
Self-employed	712 (7.5%)
Retired	2,480 (26%)
Student	605 (6.4%)
Homemaker	251 (2.6%)
Unemployed and looking for a job	288 (3.0%)
None of these/Other	204 (2.1%)
(Missing)	14 (0.2%)
**Religious service attendance**	
At least 1/week	285 (3.0%)
1/week	424 (4.5%)
1-3/month	550 (5.8%)
A few times a year	2,362 (25%)
Never	5,876 (62%)
(Missing)	9 (<0.1%)
**Education**	
Up to 8 years	235 (2.5%)

Characteristic	N = 9,506[1]
9-15 years	6,094 (64%)
16+ years	3,164 (33%)
(Missing)	13 (0.1%)
**Immigration**	
Born in this country	8,722 (92%)
Born in another country	744 (7.8%)
(Missing)	40 (0.4%)
**Religious affiliation**	
Christianity	5,052 (53%)
Islam	351 (3.7%)
Hinduism	12 (0.1%)
Buddhism	51 (0.5%)
Judaism	19 (0.2%)
Sikhism	5 (<0.1%)
Baha'i	3 (<0.1%)
Jainism	0 (0%)
Shinto	2 (<0.1%)
Taoism	0 (<0.1%)
Confucianism	4 (<0.1%)
Primal, Animist, or Folk religion	34 (0.4%)
Spiritism	0 (0%)
Umbanda, Candomble, and other African-derived religions	0 (0%)
Chinese folk/traditional religion	0 (0%)
Some other religion	60 (0.6%)
No religion/Atheist/Agnostic	3,815 (40%)
(Missing)	99 (1.0%)

[1]n (%)

Table S5b. Proportions by demographic category for Germany

Variable	Category	Mean	95% CI	SE	Global p-value
Age group	18-24	0.50	(0.45, 0.55)	0.02	0.013
	25-29	0.56	(0.52, 0.60)	0.02	
	30-39	0.57	(0.54, 0.60)	0.02	
	40-49	0.57	(0.54, 0.60)	0.02	
	50-59	0.55	(0.52, 0.58)	0.02	
	60-69	0.53	(0.50, 0.56)	0.01	
	70-79	0.58	(0.54, 0.62)	0.02	
	80 or older	0.67	(0.59, 0.76)	0.04	
Gender	Male	0.55	(0.53, 0.57)	0.01	0.069
	Female	0.56	(0.54, 0.58)	0.01	
	Other	0.26	(0.00, 0.61)	0.14	
Marital status	Married	0.57	(0.55, 0.59)	0.01	0.143
	Separated	0.58	(0.50, 0.66)	0.04	
	Divorced	0.56	(0.51, 0.60)	0.02	
	Widowed	0.55	(0.49, 0.61)	0.03	
	Never	0.53	(0.51, 0.56)	0.01	
	Domestic Partner	0.52	(0.47, 0.57)	0.03	
Employment	Employed for an employer	0.55	(0.53, 0.57)	0.01	0.009
	Self-employed	0.58	(0.53, 0.63)	0.02	
	Retired	0.57	(0.55, 0.60)	0.01	
	Student	0.48	(0.42, 0.53)	0.03	
	Homemaker	0.60	(0.53, 0.67)	0.04	
	Unemployed and looking for a job	0.48	(0.41, 0.55)	0.04	
	None of these/Other	0.54	(0.46, 0.63)	0.04	
Religious service attendance	At least 1/week	0.89	(0.85, 0.93)	0.02	< .001
	1/week	0.92	(0.90, 0.95)	0.01	
	1-3/month	0.89	(0.85, 0.92)	0.02	
	A few times a year	0.68	(0.66, 0.71)	0.01	
	Never	0.43	(0.41, 0.44)	0.01	
Education	Up to 8 years	0.56	(0.49, 0.64)	0.04	0.563
	9-15 years	0.55	(0.53, 0.56)	0.01	
	16+ years	0.56	(0.54, 0.59)	0.01	
Immigration status	Born in this country	0.55	(0.54, 0.56)	0.01	0.008

Variable	Category	Mean	95% CI	SE	Global p-value
	Born in another country	0.61	(0.57, 0.66)	0.02	
Religious affiliation	Christianity	0.67	(0.65, 0.69)	0.01	< .001
	Islam	0.83	(0.78, 0.88)	0.02	
	Hinduism	0.96	*	*	
	Buddhism	0.82	(0.69, 0.94)	0.06	
	Judaism	0.58	(0.23, 0.93)	0.14	
	Sikhism	0.74	*	*	
	Baha'i	0.71	*	*	
	Shinto	1.00	*	*	
	Taoism	0.00	*	*	
	Confucianism	0.00	*	*	
	Primal, Animist, or Folk religion	0.82	(0.61, 1.00)	0.10	
	Some other religion	0.92	(0.84, 1.00)	0.04	
	No religion/Atheist/Agnostic	0.36	(0.34, 0.38)	0.01	

Table S6a. Nationally representative descriptive statistics for Hong Kong

Characteristic	N = 3,012[1]
**Age group**	
18-24	217 (7.2%)
25-29	198 (6.6%)
30-39	507 (17%)
40-49	580 (19%)
50-59	711 (24%)
60-69	620 (21%)
70-79	164 (5.5%)
80 or older	15 (0.5%)
(Missing)	0 (0%)
**Gender**	
Male	1,390 (46%)
Female	1,620 (54%)
Other	2 (<0.1%)
(Missing)	0 (0%)
**Marital status**	
Married	2,080 (69%)
Separated	21 (0.7%)
Divorced	105 (3.5%)
Widowed	45 (1.5%)
Never	723 (24%)
Domestic Partner	37 (1.2%)
(Missing)	1 (<0.1%)
**Employment**	
Employed for an employer	2,056 (68%)
Self-employed	245 (8.1%)
Retired	423 (14%)
Student	55 (1.8%)
Homemaker	114 (3.8%)
Unemployed and looking for a job	62 (2.0%)
None of these/Other	39 (1.3%)
(Missing)	18 (0.6%)
**Religious service attendance**	
At least 1/week	237 (7.9%)
1/week	567 (19%)
1-3/month	332 (11%)
A few times a year	543 (18%)
Never	1,332 (44%)
(Missing)	1 (<0.1%)
**Education**	
Up to 8 years	433 (14%)

**Characteristic**	**N = 3,012**[1]
9-15 years	2,031 (67%)
16+ years	547 (18%)
(Missing)	0 (0%)
**Immigration**	
Born in this country	2,637 (88%)
Born in another country	321 (11%)
(Missing)	53 (1.8%)
**Religious affiliation**	
Christianity	757 (25%)
Islam	86 (2.8%)
Hinduism	20 (0.7%)
Buddhism	349 (12%)
Judaism	10 (0.3%)
Sikhism	2 (<0.1%)
Baha'i	3 (<0.1%)
Jainism	1 (<0.1%)
Shinto	19 (0.6%)
Taoism	97 (3.2%)
Confucianism	11 (0.4%)
Primal, Animist, or Folk religion	27 (0.9%)
Spiritism	0 (0%)
Umbanda, Candomble, and other African-derived religions	0 (0%)
Chinese folk/traditional religion	106 (3.5%)
Some other religion	4 (0.1%)
No religion/Atheist/Agnostic	1,518 (50%)
(Missing)	5 (0.2%)
**Race/Ethnicity**	
Chinese (Cantonese)	1,930 (64%)
Chinese (Chaoshan)	201 (6.7%)
Chinese (Fujianese)	117 (3.9%)
Chinese (Hakka)	121 (4.0%)
Chinese (Other ethnicity)	264 (8.8%)
Chinese (Shanghainese)	89 (2.9%)
East Asian (Korean, Japanese)	10 (0.3%)
Other	4 (0.1%)
South Asian (Indian, Nepalese, Pakistani)	17 (0.6%)
Southeast Asian (Filipino, Indonesian, Thailand)	46 (1.5%)
Taiwanese	14 (0.4%)
White	15 (0.5%)
(Missing)	184 (6.1%)

[1]n (%)

Table S6b. Proportions by demographic category for Hong Kong

Variable	Category	Mean	95% CI	SE	Global p-value
Age group	18-24	0.63	(0.56, 0.69)	0.03	< .001
	25-29	0.65	(0.57, 0.73)	0.04	
	30-39	0.64	(0.60, 0.69)	0.02	
	40-49	0.61	(0.56, 0.65)	0.02	
	50-59	0.62	(0.58, 0.66)	0.02	
	60-69	0.51	(0.44, 0.59)	0.04	
	70-79	0.44	(0.27, 0.60)	0.09	
	80 or older	1.00	*	*	
Gender	Male	0.58	(0.54, 0.62)	0.02	0.663
	Female	0.60	(0.57, 0.64)	0.02	
	Other	0.55	*	*	
Marital status	Married	0.62	(0.59, 0.65)	0.02	< .001
	Separated	0.42	(0.00, 0.89)	0.22	
	Divorced	0.67	(0.53, 0.81)	0.07	
	Widowed	0.22	(0.00, 0.44)	0.11	
	Never	0.54	(0.49, 0.58)	0.02	
	Domestic Partner	0.38	(0.18, 0.58)	0.10	
Employment	Employed for an employer	0.61	(0.59, 0.64)	0.01	< .001
	Self-employed	0.75	(0.68, 0.82)	0.03	
	Retired	0.46	(0.37, 0.56)	0.05	
	Student	0.49	(0.35, 0.62)	0.07	
	Homemaker	0.46	(0.31, 0.61)	0.07	
	Unemployed and looking for a job	0.55	(0.36, 0.73)	0.09	
	None of these/Other	0.56	(0.30, 0.82)	0.13	
Religious service attendance	At least 1/week	0.96	(0.93, 1.00)	0.02	< .001
	1/week	0.95	(0.92, 0.97)	0.01	
	1-3/month	0.85	(0.79, 0.91)	0.03	
	A few times a year	0.76	(0.71, 0.80)	0.02	
	Never	0.25	(0.22, 0.28)	0.02	
Education	Up to 8 years	0.70	(0.62, 0.78)	0.04	0.009
	9-15 years	0.57	(0.54, 0.60)	0.01	
	16+ years	0.60	(0.55, 0.66)	0.03	
Immigration status	Born in this country	0.59	(0.56, 0.61)	0.01	0.425

Variable	Category	Mean	95% CI	SE	Global p-value
	Born in another country	0.63	(0.54, 0.72)	0.05	
Religious affiliation	Christianity	0.94	(0.92, 0.96)	0.01	< .001
	Islam	1.00	*	*	
	Hinduism	0.93	(0.69, 1.00)	0.07	
	Buddhism	0.88	(0.84, 0.93)	0.02	
	Judaism	1.00	*	*	
	Sikhism	1.00	*	*	
	Baha'i	1.00	*	*	
	Jainism	1.00	*	*	
	Shinto	0.91	(0.54, 1.00)	0.09	
	Taoism	0.80	(0.68, 0.93)	0.06	
	Confucianism	0.88	*	*	
	Primal, Animist, or Folk religion	0.74	(0.55, 0.93)	0.08	
	Chinese folk/traditional religion	0.68	(0.54, 0.82)	0.07	
	Some other religion	1.00	*	*	
	No religion/Atheist/Agnostic	0.29	(0.26, 0.32)	0.01	
Race/ethnicity	White	0.76	(0.21, 1.00)	0.13	< .001
	Other	0.75	*	*	
	Chinese (Cantonese)	0.56	(0.53, 0.59)	0.02	
	Chinese (Chaoshan)	0.63	(0.54, 0.72)	0.05	
	Chinese (Fujianese)	0.57	(0.46, 0.67)	0.05	
	Chinese (Hakka)	0.60	(0.49, 0.72)	0.06	
	Chinese (Shanghainese)	0.79	(0.67, 0.91)	0.06	
	Chinese (Other ethnicity)	0.65	(0.57, 0.73)	0.04	
	East Asian (Korean, Japanese)	0.77	(0.00, 1.00)	0.18	
	Southeast Asian (Filipino, Indonesian, Thailand)	1.00	*	*	
	South Asian (Indian, Nepalese, Pakistani)	0.99	(0.73, 1.00)	0.05	

Variable	Category	Mean	95% CI	SE	Global p-value
	Taiwanese	0.52	*	*	

Table S7a. Nationally representative descriptive statistics for India

Characteristic	N = 12,765[1]
**Age group**	
18-24	2,543 (20%)
25-29	1,640 (13%)
30-39	3,109 (24%)
40-49	2,275 (18%)
50-59	1,574 (12%)
60-69	1,188 (9.3%)
70-79	370 (2.9%)
80 or older	67 (0.5%)
(Missing)	0 (0%)
**Gender**	
Male	6,473 (51%)
Female	6,292 (49%)
Other	0 (0%)
(Missing)	0 (0%)
**Marital status**	
Married	9,848 (77%)
Separated	45 (0.4%)
Divorced	25 (0.2%)
Widowed	445 (3.5%)
Never	2,065 (16%)
Domestic Partner	269 (2.1%)
(Missing)	69 (0.5%)
**Employment**	
Employed for an employer	2,660 (21%)
Self-employed	3,401 (27%)
Retired	286 (2.2%)
Student	532 (4.2%)
Homemaker	4,221 (33%)
Unemployed and looking for a job	902 (7.1%)
None of these/Other	715 (5.6%)
(Missing)	48 (0.4%)
**Religious service attendance**	
At least 1/week	2,875 (23%)
1/week	3,166 (25%)
1-3/month	2,740 (21%)
A few times a year	2,090 (16%)
Never	1,823 (14%)
(Missing)	71 (0.6%)
**Education**	
Up to 8 years	11,422 (89%)

**Characteristic**	**N = 12,765**[1]
9-15 years	1,194 (9.4%)
16+ years	145 (1.1%)
(Missing)	4 (<0.1%)
**Immigration**	
Born in this country	12,629 (99%)
Born in another country	110 (0.9%)
(Missing)	26 (0.2%)
**Religious affiliation**	
Christianity	306 (2.4%)
Islam	1,555 (12%)
Hinduism	10,362 (81%)
Buddhism	230 (1.8%)
Judaism	0 (0%)
Sikhism	127 (1.0%)
Baha'i	0 (0%)
Jainism	10 (<0.1%)
Shinto	1 (<0.1%)
Taoism	0 (0%)
Confucianism	0 (0%)
Primal, Animist, or Folk religion	30 (0.2%)
Spiritism	0 (0%)
Umbanda, Candomble, and other African-derived religions	0 (0%)
Chinese folk/traditional religion	0 (0%)
Some other religion	67 (0.5%)
No religion/Atheist/Agnostic	13 (0.1%)
(Missing)	62 (0.5%)
**Race/Ethnicity**	
General	3,538 (28%)
Other backward caste	4,177 (33%)
Schedule caste	3,599 (28%)
Schedule tribe	1,185 (9.3%)
(Missing)	267 (2.1%)

[1]n (%)

Table S7b. Proportions by demographic category for India

Variable	Category	Mean	95% CI	SE	Global p-value
Age group	18-24	0.95	(0.93, 0.96)	0.01	< .001
	25-29	0.95	(0.94, 0.96)	0.01	
	30-39	0.95	(0.94, 0.96)	0.00	
	40-49	0.96	(0.95, 0.97)	0.01	
	50-59	0.97	(0.96, 0.98)	0.01	
	60-69	0.96	(0.95, 0.98)	0.01	
	70-79	0.95	(0.92, 0.99)	0.02	
	80 or older	1.00	*	*	
Gender	Male	0.94	(0.93, 0.95)	0.00	< .001
	Female	0.97	(0.96, 0.97)	0.00	
Marital status	Married	0.96	(0.96, 0.97)	0.00	< .001
	Separated	0.89	(0.77, 1.00)	0.06	
	Divorced	1.00	*	*	
	Widowed	0.97	(0.94, 0.99)	0.01	
	Never	0.92	(0.91, 0.94)	0.01	
	Domestic Partner	0.92	(0.88, 0.96)	0.02	
Employment	Employed for an employer	0.93	(0.92, 0.95)	0.01	< .001
	Self-employed	0.95	(0.95, 0.96)	0.00	
	Retired	0.94	(0.91, 0.98)	0.02	
	Student	0.92	(0.89, 0.94)	0.01	
	Homemaker	0.97	(0.97, 0.98)	0.00	
	Unemployed and looking for a job	0.97	(0.95, 0.98)	0.01	
	None of these/Other	0.96	(0.94, 0.98)	0.01	
Religious service attendance	At least 1/week	0.98	(0.98, 0.99)	0.00	< .001
	1/week	0.98	(0.97, 0.98)	0.00	
	1-3/month	0.97	(0.96, 0.98)	0.00	
	A few times a year	0.95	(0.94, 0.96)	0.01	
	Never	0.86	(0.84, 0.88)	0.01	
Education	Up to 8 years	0.96	(0.95, 0.96)	0.00	0.002
	9-15 years	0.93	(0.91, 0.94)	0.01	
	16+ years	0.93	(0.88, 0.99)	0.03	
Immigration status	Born in this country	0.95	(0.95, 0.96)	0.00	0.202
	Born in another country	0.98	(0.94, 1.00)	0.02	

Variable	Category	Mean	95% CI	SE	Global p-value
Religious affiliation	Christianity	0.95	(0.92, 0.98)	0.02	< .001
	Islam	0.97	(0.96, 0.98)	0.01	
	Hinduism	0.96	(0.95, 0.96)	0.00	
	Buddhism	0.85	(0.79, 0.90)	0.03	
	Sikhism	0.94	(0.89, 1.00)	0.03	
	Jainism	1.00	*	*	
	Shinto	1.00	*	*	
	Primal, Animist, or Folk religion	1.00	*	*	
	Some other religion	0.95	(0.88, 1.00)	0.03	
	No religion/Atheist/Agnostic	0.44	(0.00, 1.00)	0.18	
Race/ethnicity	General	0.96	(0.95, 0.97)	0.00	0.069
	Other backward caste	0.96	(0.95, 0.97)	0.00	
	Schedule caste	0.94	(0.93, 0.95)	0.01	
	Schedule tribe	0.96	(0.94, 0.98)	0.01	

Table S8a. Nationally representative descriptive statistics for Indonesia

Characteristic	N = 6,992[1]
**Age group**	
18-24	1,216 (17%)
25-29	849 (12%)
30-39	1,591 (23%)
40-49	1,576 (23%)
50-59	1,169 (17%)
60-69	490 (7.0%)
70-79	83 (1.2%)
80 or older	17 (0.2%)
(Missing)	0 (0%)
**Gender**	
Male	3,461 (50%)
Female	3,513 (50%)
Other	7 (<0.1%)
(Missing)	11 (0.2%)
**Marital status**	
Married	4,846 (69%)
Separated	81 (1.2%)
Divorced	196 (2.8%)
Widowed	425 (6.1%)
Never	1,381 (20%)
Domestic Partner	18 (0.3%)
(Missing)	45 (0.6%)
**Employment**	
Employed for an employer	1,323 (19%)
Self-employed	2,187 (31%)
Retired	78 (1.1%)
Student	272 (3.9%)
Homemaker	2,138 (31%)
Unemployed and looking for a job	529 (7.6%)
None of these/Other	448 (6.4%)
(Missing)	18 (0.3%)
**Religious service attendance**	
At least 1/week	2,667 (38%)
1/week	2,529 (36%)
1-3/month	786 (11%)
A few times a year	659 (9.4%)
Never	332 (4.8%)
(Missing)	18 (0.3%)
**Education**	
Up to 8 years	3,079 (44%)

Characteristic	N = 6,992[1]
9-15 years	3,491 (50%)
16+ years	419 (6.0%)
(Missing)	2 (<0.1%)
**Immigration**	
Born in this country	6,958 (100%)
Born in another country	34 (0.5%)
(Missing)	0 (0%)
**Religious affiliation**	
Christianity	504 (7.2%)
Islam	6,406 (92%)
Hinduism	73 (1.0%)
Buddhism	3 (<0.1%)
Judaism	0 (0%)
Sikhism	0 (0%)
Baha'i	0 (0%)
Jainism	0 (0%)
Shinto	0 (0%)
Taoism	1 (<0.1%)
Confucianism	0 (0%)
Primal, Animist, or Folk religion	0 (0%)
Spiritism	0 (0%)
Umbanda, Candomble, and other African-derived religions	0 (0%)
Chinese folk/traditional religion	0 (0%)
Some other religion	1 (<0.1%)
No religion/Atheist/Agnostic	0 (0%)
(Missing)	4 (<0.1%)
**Race/Ethnicity**	
Bali	69 (1.0%)
Banjar/Melayu Banjar	320 (4.6%)
Batak	165 (2.4%)
Betawi	251 (3.6%)
Bugis	243 (3.5%)
Jawa	2,846 (41%)
Madura	262 (3.7%)
Makasar	91 (1.3%)
Minangkabau	273 (3.9%)
Other	1,262 (18%)
Sunda/Parahyangan	1,172 (17%)
(Missing)	38 (0.5%)

[1]n (%)

Table S8b. Proportions by demographic category for Indonesia

Variable	Category	Mean	95% CI	SE	Global p-value
Age group	18-24	0.96	(0.95, 0.98)	0.01	< .001
	25-29	0.98	(0.97, 0.99)	0.00	
	30-39	0.98	(0.97, 0.99)	0.00	
	40-49	0.97	(0.96, 0.98)	0.01	
	50-59	0.97	(0.95, 0.98)	0.01	
	60-69	0.96	(0.93, 0.99)	0.01	
	70-79	0.96	(0.89, 1.00)	0.04	
	80 or older	1.00	(1.00, 1.00)	0.00	
Gender	Male	0.97	(0.96, 0.98)	0.00	0.837
	Female	0.97	(0.96, 0.98)	0.00	
	Other	0.92	(0.61, 1.00)	0.08	
Marital status	Married	0.97	(0.97, 0.98)	0.00	< .001
	Separated	0.96	(0.89, 1.00)	0.04	
	Divorced	0.95	(0.92, 0.99)	0.02	
	Widowed	0.94	(0.91, 0.98)	0.02	
	Never	0.97	(0.96, 0.98)	0.01	
	Domestic Partner	1.00	*	*	
Employment	Employed for an employer	0.98	(0.96, 0.99)	0.01	< .001
	Self-employed	0.97	(0.95, 0.98)	0.01	
	Retired	0.97	(0.93, 1.00)	0.02	
	Student	1.00	(0.99, 1.00)	0.00	
	Homemaker	0.97	(0.96, 0.98)	0.00	
	Unemployed and looking for a job	0.95	(0.92, 0.98)	0.01	
	None of these/Other	0.98	(0.96, 0.99)	0.01	
Religious service attendance	At least 1/week	0.98	(0.97, 0.99)	0.00	0.008
	1/week	0.97	(0.96, 0.98)	0.00	
	1-3/month	0.98	(0.97, 0.99)	0.00	
	A few times a year	0.97	(0.96, 0.99)	0.01	
	Never	0.90	(0.85, 0.94)	0.02	
Education	Up to 8 years	0.96	(0.95, 0.98)	0.01	< .001
	9-15 years	0.97	(0.97, 0.98)	0.00	
	16+ years	1.00	(0.99, 1.00)	0.00	
Immigration status	Born in this country	0.97	(0.97, 0.98)	0.00	< .001

Variable	Category	Mean	95% CI	SE	Global p-value
	Born in another country	1.00	*	*	
Religious affiliation	Christianity	0.98	(0.97, 1.00)	0.01	0.993
	Islam	0.97	(0.96, 0.98)	0.00	
	Hinduism	0.98	(0.95, 1.00)	0.02	
	Buddhism	1.00	*	*	
	Taoism	1.00	*	*	
	Some other religion	1.00	*	*	
Race/ethnicity	Banjar/Melayu Banjar	0.98	(0.96, 0.99)	0.01	0.741
	Betawi	0.95	(0.92, 0.99)	0.02	
	Bugis	0.98	(0.96, 1.00)	0.01	
	Jawa	0.97	(0.96, 0.98)	0.00	
	Madura	0.95	(0.92, 0.99)	0.02	
	Minangkabau	0.98	(0.97, 1.00)	0.01	
	Sunda/Parahyangan	0.97	(0.95, 0.98)	0.01	
	Bali	0.98	(0.94, 1.00)	0.02	
	Batak	0.95	(0.90, 1.00)	0.03	
	Makasar	0.98	(0.96, 1.00)	0.01	
	Other	0.98	(0.97, 0.99)	0.00	

Table S9a. Nationally representative descriptive statistics for Israel

Characteristic	N = 3,669[1]
**Age group**	
18-24	553 (15%)
25-29	407 (11%)
30-39	666 (18%)
40-49	616 (17%)
50-59	542 (15%)
60-69	469 (13%)
70-79	336 (9.2%)
80 or older	79 (2.2%)
(Missing)	0 (0%)
**Gender**	
Male	1,791 (49%)
Female	1,872 (51%)
Other	0 (<0.1%)
(Missing)	6 (0.2%)
**Marital status**	
Married	2,056 (56%)
Separated	48 (1.3%)
Divorced	258 (7.0%)
Widowed	212 (5.8%)
Never	834 (23%)
Domestic Partner	193 (5.3%)
(Missing)	69 (1.9%)
**Employment**	
Employed for an employer	1,793 (49%)
Self-employed	424 (12%)
Retired	576 (16%)
Student	388 (11%)
Homemaker	211 (5.7%)
Unemployed and looking for a job	148 (4.0%)
None of these/Other	118 (3.2%)
(Missing)	10 (0.3%)
**Religious service attendance**	
At least 1/week	649 (18%)
1/week	495 (14%)
1-3/month	374 (10%)
A few times a year	1,014 (28%)
Never	1,122 (31%)
(Missing)	14 (0.4%)
**Education**	
Up to 8 years	224 (6.1%)

**Characteristic**	**N = 3,669**[1]
9-15 years	1,517 (41%)
16+ years	1,926 (52%)
(Missing)	2 (<0.1%)
**Immigration**	
Born in this country	2,796 (76%)
Born in another country	868 (24%)
(Missing)	5 (0.1%)
**Religious affiliation**	
Christianity	39 (1.1%)
Islam	656 (18%)
Hinduism	0 (0%)
Buddhism	0 (0%)
Judaism	2,897 (79%)
Sikhism	0 (0%)
Baha'i	2 (<0.1%)
Jainism	0 (0%)
Shinto	0 (0%)
Taoism	1 (<0.1%)
Confucianism	0 (0%)
Primal, Animist, or Folk religion	1 (<0.1%)
Spiritism	0 (0%)
Umbanda, Candomble, and other African-derived religions	0 (0%)
Chinese folk/traditional religion	0 (0%)
Some other religion	5 (0.1%)
No religion/Atheist/Agnostic	64 (1.7%)
(Missing)	4 (0.1%)
**Race/Ethnicity**	
Arab	674 (18%)
Jewish	2,926 (80%)
Other	39 (1.1%)
(Missing)	30 (0.8%)

[1]n (%)

Table S9b. Proportions by demographic category for Israel

Variable	Category	Mean	95% CI	SE	Global p-value
Age group	18-24	0.77	(0.72, 0.82)	0.03	0.023
	25-29	0.69	(0.61, 0.76)	0.04	
	30-39	0.74	(0.70, 0.78)	0.02	
	40-49	0.72	(0.67, 0.77)	0.03	
	50-59	0.72	(0.67, 0.77)	0.03	
	60-69	0.73	(0.67, 0.79)	0.03	
	70-79	0.64	(0.55, 0.72)	0.04	
	80 or older	0.63	(0.49, 0.76)	0.07	
Gender	Male	0.71	(0.67, 0.74)	0.02	< .001
	Female	0.73	(0.69, 0.77)	0.02	
	Other	0.00	*	*	
Marital status	Married	0.75	(0.72, 0.79)	0.02	< .001
	Separated	0.66	(0.54, 0.79)	0.06	
	Divorced	0.65	(0.57, 0.73)	0.04	
	Widowed	0.76	(0.68, 0.84)	0.04	
	Never	0.70	(0.66, 0.75)	0.03	
	Domestic Partner	0.50	(0.40, 0.60)	0.05	
Employment	Employed for an employer	0.69	(0.65, 0.73)	0.02	< .001
	Self-employed	0.68	(0.62, 0.74)	0.03	
	Retired	0.70	(0.63, 0.77)	0.04	
	Student	0.76	(0.70, 0.83)	0.03	
	Homemaker	0.94	(0.90, 0.98)	0.02	
	Unemployed and looking for a job	0.86	(0.79, 0.93)	0.04	
	None of these/Other	0.71	(0.59, 0.82)	0.06	
Religious service attendance	At least 1/week	0.99	(0.98, 1.00)	0.01	< .001
	1/week	0.97	(0.95, 0.99)	0.01	
	1-3/month	0.91	(0.88, 0.95)	0.02	
	A few times a year	0.75	(0.69, 0.81)	0.03	
	Never	0.36	(0.31, 0.41)	0.03	
Education	Up to 8 years	0.94	(0.90, 0.99)	0.02	< .001
	9-15 years	0.81	(0.78, 0.84)	0.02	
	16+ years	0.63	(0.58, 0.67)	0.02	
Immigration status	Born in this country	0.77	(0.73, 0.80)	0.02	< .001

Variable	Category	Mean	95% CI	SE	Global p-value
	Born in another country	0.57	(0.51, 0.63)	0.03	
Religious affiliation	Christianity	0.85	(0.74, 0.96)	0.05	< .001
	Islam	0.96	(0.94, 0.98)	0.01	
	Judaism	0.68	(0.64, 0.72)	0.02	
	Baha'i	1.00	*	*	
	Taoism	1.00	*	*	
	Primal, Animist, or Folk religion	1.00	*	*	
	Some other religion	1.00	*	*	
	No religion/Atheist/Agnostic	0.13	(0.02, 0.24)	0.06	
Race/ethnicity	Arab	0.96	(0.94, 0.98)	0.01	< .001
	Jewish	0.67	(0.63, 0.71)	0.02	
	Other	0.32	(0.12, 0.53)	0.10	

Table S10a. Nationally representative descriptive statistics for Japan

Characteristic	N = 20,543[1]
**Age group**	
18-24	1,589 (7.7%)
25-29	806 (3.9%)
30-39	2,851 (14%)
40-49	3,363 (16%)
50-59	3,770 (18%)
60-69	4,118 (20%)
70-79	3,554 (17%)
80 or older	493 (2.4%)
(Missing)	0 (0%)
**Gender**	
Male	9,847 (48%)
Female	10,602 (52%)
Other	28 (0.1%)
(Missing)	66 (0.3%)
**Marital status**	
Married	11,837 (58%)
Separated	190 (0.9%)
Divorced	2,126 (10%)
Widowed	1,179 (5.7%)
Never	5,004 (24%)
Domestic Partner	144 (0.7%)
(Missing)	64 (0.3%)
**Employment**	
Employed for an employer	10,853 (53%)
Self-employed	1,748 (8.5%)
Retired	2,535 (12%)
Student	491 (2.4%)
Homemaker	1,276 (6.2%)
Unemployed and looking for a job	622 (3.0%)
None of these/Other	2,983 (15%)
(Missing)	36 (0.2%)
**Religious service attendance**	
At least 1/week	316 (1.5%)
1/week	348 (1.7%)
1-3/month	862 (4.2%)
A few times a year	3,112 (15%)
Never	15,788 (77%)
(Missing)	117 (0.6%)
**Education**	
Up to 8 years	567 (2.8%)

**Characteristic**	**N = 20,543**[1]
9-15 years	14,893 (72%)
16+ years	5,083 (25%)
(Missing)	0 (0%)
**Immigration**	
Born in this country	19,548 (95%)
Born in another country	158 (0.8%)
(Missing)	837 (4.1%)
**Religious affiliation**	
Christianity	381 (1.9%)
Islam	10 (<0.1%)
Hinduism	5 (<0.1%)
Buddhism	6,709 (33%)
Judaism	10 (<0.1%)
Sikhism	6 (<0.1%)
Baha'i	2 (<0.1%)
Jainism	11 (<0.1%)
Shinto	469 (2.3%)
Taoism	7 (<0.1%)
Confucianism	17 (<0.1%)
Primal, Animist, or Folk religion	19 (<0.1%)
Spiritism	0 (0%)
Umbanda, Candomble, and other African-derived religions	0 (0%)
Chinese folk/traditional religion	0 (0%)
Some other religion	46 (0.2%)
No religion/Atheist/Agnostic	12,497 (61%)
(Missing)	355 (1.7%)

[1]n (%)

Table S10b. Proportions by demographic category for Japan

Variable	Category	Mean	95% CI	SE	Global p-value
Age group	18-24	0.23	(0.20, 0.26)	0.01	< .001
	25-29	0.23	(0.19, 0.26)	0.02	
	30-39	0.22	(0.20, 0.24)	0.01	
	40-49	0.20	(0.19, 0.22)	0.01	
	50-59	0.20	(0.18, 0.21)	0.01	
	60-69	0.19	(0.18, 0.20)	0.01	
	70-79	0.17	(0.16, 0.19)	0.01	
	80 or older	0.23	(0.19, 0.28)	0.02	
Gender	Male	0.20	(0.19, 0.21)	0.00	0.057
	Female	0.20	(0.19, 0.21)	0.00	
	Other	0.42	(0.23, 0.62)	0.09	
Marital status	Married	0.19	(0.18, 0.20)	0.00	< .001
	Separated	0.35	(0.26, 0.44)	0.05	
	Divorced	0.20	(0.17, 0.22)	0.01	
	Widowed	0.19	(0.16, 0.23)	0.02	
	Never	0.22	(0.20, 0.23)	0.01	
	Domestic Partner	0.27	(0.17, 0.36)	0.05	
Employment	Employed for an employer	0.21	(0.20, 0.22)	0.00	< .001
	Self-employed	0.25	(0.23, 0.28)	0.01	
	Retired	0.18	(0.17, 0.20)	0.01	
	Student	0.23	(0.20, 0.27)	0.02	
	Homemaker	0.19	(0.17, 0.21)	0.01	
	Unemployed and looking for a job	0.19	(0.16, 0.22)	0.01	
	None of these/Other	0.16	(0.15, 0.18)	0.01	
Religious service attendance	At least 1/week	0.57	(0.50, 0.63)	0.03	< .001
	1/week	0.69	(0.63, 0.75)	0.03	
	1-3/month	0.51	(0.46, 0.55)	0.02	
	A few times a year	0.30	(0.28, 0.32)	0.01	
	Never	0.15	(0.14, 0.15)	0.00	
Education	Up to 8 years	0.19	(0.15, 0.23)	0.02	< .001
	9-15 years	0.19	(0.18, 0.19)	0.00	
	16+ years	0.25	(0.23, 0.26)	0.01	
Immigration status	Born in this country	0.20	(0.19, 0.21)	0.00	0.124

Variable	Category	Mean	95% CI	SE	Global p-value
	Born in another country	0.27	(0.18, 0.36)	0.05	
Religious affiliation	Christianity	0.77	(0.72, 0.82)	0.02	< .001
	Islam	0.65	*	*	
	Hinduism	0.67	*	*	
	Buddhism	0.25	(0.24, 0.26)	0.01	
	Judaism	0.66	*	*	
	Sikhism	0.86	*	*	
	Baha'i	1.00	*	*	
	Jainism	0.78	(0.38, 1.00)	0.20	
	Shinto	0.47	(0.42, 0.52)	0.03	
	Taoism	0.37	*	*	
	Confucianism	0.46	(0.00, 1.00)	0.12	
	Primal, Animist, or Folk religion	0.58	(0.12, 1.00)	0.15	
	Some other religion	0.52	(0.33, 0.70)	0.09	
	No religion/Atheist/Agnostic	0.14	(0.13, 0.15)	0.00	

Table S11a. Nationally representative descriptive statistics for Kenya

**Characteristic**	**N = 11,389**[1]
**Age group**	
18-24	2,868 (25%)
25-29	2,035 (18%)
30-39	2,564 (23%)
40-49	1,708 (15%)
50-59	1,072 (9.4%)
60-69	710 (6.2%)
70-79	360 (3.2%)
80 or older	67 (0.6%)
(Missing)	5 (<0.1%)
**Gender**	
Male	5,567 (49%)
Female	5,813 (51%)
Other	2 (<0.1%)
(Missing)	7 (<0.1%)
**Marital status**	
Married	6,626 (58%)
Separated	467 (4.1%)
Divorced	111 (1.0%)
Widowed	464 (4.1%)
Never	3,531 (31%)
Domestic Partner	146 (1.3%)
(Missing)	43 (0.4%)
**Employment**	
Employed for an employer	1,467 (13%)
Self-employed	3,630 (32%)
Retired	319 (2.8%)
Student	1,136 (10.0%)
Homemaker	1,537 (13%)
Unemployed and looking for a job	3,153 (28%)
None of these/Other	138 (1.2%)
(Missing)	9 (<0.1%)
**Religious service attendance**	
At least 1/week	2,774 (24%)
1/week	6,063 (53%)
1-3/month	1,219 (11%)
A few times a year	855 (7.5%)
Never	465 (4.1%)
(Missing)	13 (0.1%)
**Education**	
Up to 8 years	4,485 (39%)

**Characteristic**	**N = 11,389**[1]
9-15 years	6,115 (54%)
16+ years	783 (6.9%)
(Missing)	6 (<0.1%)
**Immigration**	
Born in this country	11,270 (99%)
Born in another country	117 (1.0%)
(Missing)	2 (<0.1%)
**Religious affiliation**	
Christianity	10,334 (91%)
Islam	918 (8.1%)
Hinduism	0 (0%)
Buddhism	1 (<0.1%)
Judaism	3 (<0.1%)
Sikhism	0 (0%)
Baha'i	1 (<0.1%)
Jainism	1 (<0.1%)
Shinto	0 (0%)
Taoism	0 (0%)
Confucianism	3 (<0.1%)
Primal, Animist, or Folk religion	7 (<0.1%)
Spiritism	0 (0%)
Umbanda, Candomble, and other African-derived religions	0 (0%)
Chinese folk/traditional religion	0 (0%)
Some other religion	5 (<0.1%)
No religion/Atheist/Agnostic	108 (0.9%)
(Missing)	9 (<0.1%)
**Race/Ethnicity**	
Embu	197 (1.7%)
Kalenjin	1,377 (12%)
Kamba	1,299 (11%)
Kenyan Somali/Somali	396 (3.5%)
Kikuyu	2,119 (19%)
Kisii	789 (6.9%)
Luhya	1,943 (17%)
Luo	1,120 (9.8%)
Maasai	237 (2.1%)
Meru	630 (5.5%)
Miji Kenda tribes	708 (6.2%)
Other	548 (4.8%)
(Missing)	27 (0.2%)

[1] n (%)

Table S11b. Proportions by demographic category for Kenya

Variable	Category	Mean	95% CI	SE	Global p-value
Age group	18-24	0.98	(0.98, 0.99)	0.00	< .001
	25-29	0.98	(0.97, 0.98)	0.00	
	30-39	0.98	(0.98, 0.99)	0.00	
	40-49	0.99	(0.98, 1.00)	0.00	
	50-59	1.00	(0.99, 1.00)	0.00	
	60-69	0.99	(0.98, 1.00)	0.01	
	70-79	0.98	(0.96, 1.00)	0.01	
	80 or older	1.00	*	*	
Gender	Male	0.98	(0.98, 0.99)	0.00	< .001
	Female	0.99	(0.98, 0.99)	0.00	
	Other	1.00	*	*	
Marital status	Married	0.99	(0.99, 0.99)	0.00	0.027
	Separated	0.97	(0.95, 0.99)	0.01	
	Divorced	0.97	(0.94, 1.00)	0.02	
	Widowed	0.99	(0.97, 1.00)	0.01	
	Never	0.98	(0.97, 0.99)	0.00	
	Domestic Partner	0.97	(0.93, 1.00)	0.02	
Employment	Employed for an employer	0.99	(0.98, 1.00)	0.00	0.008
	Self-employed	0.99	(0.99, 0.99)	0.00	
	Retired	0.98	(0.96, 1.00)	0.01	
	Student	0.98	(0.98, 0.99)	0.00	
	Homemaker	0.99	(0.98, 1.00)	0.00	
	Unemployed and looking for a job	0.98	(0.97, 0.98)	0.00	
	None of these/Other	0.97	(0.93, 1.00)	0.02	
Religious service attendance	At least 1/week	0.99	(0.99, 0.99)	0.00	< .001
	1/week	0.99	(0.99, 0.99)	0.00	
	1-3/month	0.98	(0.98, 0.99)	0.00	
	A few times a year	0.97	(0.96, 0.99)	0.01	
	Never	0.91	(0.88, 0.94)	0.02	
Education	Up to 8 years	0.99	(0.98, 0.99)	0.00	0.144
	9-15 years	0.98	(0.98, 0.99)	0.00	
	16+ years	0.98	(0.97, 1.00)	0.01	
Immigration status	Born in this country	0.98	(0.98, 0.99)	0.00	0.581

Variable	Category	Mean	95% CI	SE	Global p-value
	Born in another country	0.99	(0.97, 1.00)	0.01	
Religious affiliation	Christianity	0.98	(0.98, 0.99)	0.00	1.000
	Islam	0.99	(0.98, 1.00)	0.00	
	Buddhism	1.00	*	*	
	Judaism	1.00	*	*	
	Baha'i	1.00	*	*	
	Jainism	1.00	*	*	
	Confucianism	1.00	*	*	
	Primal, Animist, or Folk religion	1.00	*	*	
	Some other religion	1.00	*	*	
	No religion/Atheist/Agnostic	0.93	(0.87, 0.99)	0.03	
Race/ethnicity	Luhya	0.99	(0.98, 0.99)	0.00	0.812
	Luo	0.98	(0.97, 0.99)	0.00	
	Kalenjin	0.99	(0.98, 1.00)	0.00	
	Kamba	0.98	(0.97, 0.99)	0.00	
	Kikuyu	0.98	(0.97, 0.99)	0.00	
	Kisii	0.99	(0.99, 1.00)	0.00	
	Maasai	0.99	(0.98, 1.00)	0.01	
	Meru	0.97	(0.95, 1.00)	0.01	
	Kenyan Somali/Somali	1.00	*	*	
	Miji Kenda tribes	0.99	(0.98, 1.00)	0.01	
	Embu	1.00	*	*	
	Other	1.00	(0.99, 1.00)	0.00	

Table S12a. Nationally representative descriptive statistics for Mexico

Characteristic	N = 5,776[1]
**Age group**	
18-24	986 (17%)
25-29	623 (11%)
30-39	1,312 (23%)
40-49	1,027 (18%)
50-59	873 (15%)
60-69	611 (11%)
70-79	277 (4.8%)
80 or older	68 (1.2%)
(Missing)	0 (0%)
**Gender**	
Male	2,755 (48%)
Female	2,997 (52%)
Other	3 (<0.1%)
(Missing)	21 (0.4%)
**Marital status**	
Married	2,089 (36%)
Separated	403 (7.0%)
Divorced	230 (4.0%)
Widowed	347 (6.0%)
Never	1,432 (25%)
Domestic Partner	1,109 (19%)
(Missing)	166 (2.9%)
**Employment**	
Employed for an employer	1,921 (33%)
Self-employed	1,091 (19%)
Retired	386 (6.7%)
Student	247 (4.3%)
Homemaker	1,257 (22%)
Unemployed and looking for a job	564 (9.8%)
None of these/Other	169 (2.9%)
(Missing)	141 (2.4%)
**Religious service attendance**	
At least 1/week	609 (11%)
1/week	1,261 (22%)
1-3/month	676 (12%)
A few times a year	2,054 (36%)
Never	1,134 (20%)
(Missing)	43 (0.7%)
**Education**	
Up to 8 years	1,291 (22%)

**Characteristic**	**N = 5,776**[1]
9-15 years	3,180 (55%)
16+ years	1,304 (23%)
(Missing)	1 (<0.1%)
**Immigration**	
Born in this country	5,517 (96%)
Born in another country	108 (1.9%)
(Missing)	151 (2.6%)
**Religious affiliation**	
Christianity	4,844 (84%)
Islam	2 (<0.1%)
Hinduism	3 (<0.1%)
Buddhism	6 (0.1%)
Judaism	7 (0.1%)
Sikhism	0 (0%)
Baha'i	1 (<0.1%)
Jainism	1 (<0.1%)
Shinto	2 (<0.1%)
Taoism	4 (<0.1%)
Confucianism	1 (<0.1%)
Primal, Animist, or Folk religion	20 (0.3%)
Spiritism	0 (0%)
Umbanda, Candomble, and other African-derived religions	0 (0%)
Chinese folk/traditional religion	0 (0%)
Some other religion	41 (0.7%)
No religion/Atheist/Agnostic	770 (13%)
(Missing)	75 (1.3%)
**Race/Ethnicity**	
Black	108 (1.9%)
Indigenous	594 (10%)
Mestizo	2,762 (48%)
Mulatto	63 (1.1%)
Other	339 (5.9%)
White	1,116 (19%)
(Missing)	794 (14%)

[1]n (%)

Table S12b. Proportions by demographic category for Mexico

Variable	Category	Mean	95% CI	SE	Global p-value
Age group	18-24	0.84	(0.82, 0.87)	0.01	< .001
	25-29	0.89	(0.86, 0.92)	0.01	
	30-39	0.90	(0.88, 0.92)	0.01	
	40-49	0.95	(0.93, 0.96)	0.01	
	50-59	0.96	(0.94, 0.97)	0.01	
	60-69	0.93	(0.91, 0.96)	0.01	
	70-79	0.95	(0.91, 0.99)	0.02	
	80 or older	0.95	(0.85, 1.00)	0.05	
Gender	Male	0.89	(0.88, 0.91)	0.01	< .001
	Female	0.93	(0.92, 0.94)	0.01	
	Other	0.63	*	*	
Marital status	Married	0.94	(0.92, 0.95)	0.01	< .001
	Separated	0.94	(0.92, 0.97)	0.01	
	Divorced	0.92	(0.88, 0.96)	0.02	
	Widowed	0.96	(0.93, 0.99)	0.01	
	Never	0.86	(0.84, 0.89)	0.01	
	Domestic Partner	0.91	(0.89, 0.93)	0.01	
Employment	Employed for an employer	0.91	(0.89, 0.92)	0.01	< .001
	Self-employed	0.92	(0.91, 0.94)	0.01	
	Retired	0.92	(0.88, 0.96)	0.02	
	Student	0.80	(0.74, 0.86)	0.03	
	Homemaker	0.95	(0.93, 0.96)	0.01	
	Unemployed and looking for a job	0.91	(0.88, 0.93)	0.01	
	None of these/Other	0.86	(0.79, 0.93)	0.04	
Religious service attendance	At least 1/week	0.99	(0.98, 1.00)	0.00	< .001
	1/week	0.97	(0.96, 0.98)	0.01	
	1-3/month	0.96	(0.94, 0.98)	0.01	
	A few times a year	0.93	(0.92, 0.94)	0.01	
	Never	0.75	(0.72, 0.78)	0.02	
Education	Up to 8 years	0.94	(0.92, 0.96)	0.01	< .001
	9-15 years	0.91	(0.90, 0.93)	0.01	
	16+ years	0.89	(0.86, 0.91)	0.01	
Immigration status	Born in this country	0.92	(0.91, 0.92)	0.00	0.013

Variable	Category	Mean	95% CI	SE	Global p-value
	Born in another country	0.80	(0.70, 0.89)	0.05	
Religious affiliation	Christianity	0.95	(0.95, 0.96)	0.00	1.000
	Islam	1.00	*	*	
	Hinduism	0.80	*	*	
	Buddhism	0.83	*	*	
	Judaism	0.86	*	*	
	Baha'i	1.00	*	*	
	Jainism	1.00	*	*	
	Shinto	1.00	*	*	
	Taoism	1.00	*	*	
	Confucianism	1.00	*	*	
	Primal, Animist, or Folk religion	1.00	*	*	
	Some other religion	0.99	(0.97, 1.00)	0.01	
	No religion/Atheist/Agnostic	0.66	(0.62, 0.70)	0.02	
Race/ethnicity	Black	0.92	(0.86, 0.98)	0.03	0.093
	Indigenous	0.91	(0.88, 0.94)	0.02	
	White	0.92	(0.91, 0.94)	0.01	
	Mestizo	0.91	(0.89, 0.92)	0.01	
	Mulatto	0.87	(0.76, 0.98)	0.05	
	Other	0.95	(0.92, 0.97)	0.01	

Table S13a. Nationally representative descriptive statistics for Nigeria

Characteristic	N = 6,827[1]
**Age group**	
18-24	1,533 (22%)
25-29	1,193 (17%)
30-39	1,943 (28%)
40-49	1,059 (16%)
50-59	619 (9.1%)
60-69	296 (4.3%)
70-79	133 (2.0%)
80 or older	50 (0.7%)
(Missing)	0 (0%)
**Gender**	
Male	3,371 (49%)
Female	3,456 (51%)
Other	0 (<0.1%)
(Missing)	0 (0%)
**Marital status**	
Married	4,065 (60%)
Separated	117 (1.7%)
Divorced	71 (1.0%)
Widowed	231 (3.4%)
Never	2,289 (34%)
Domestic Partner	12 (0.2%)
(Missing)	42 (0.6%)
**Employment**	
Employed for an employer	699 (10%)
Self-employed	3,898 (57%)
Retired	178 (2.6%)
Student	650 (9.5%)
Homemaker	499 (7.3%)
Unemployed and looking for a job	684 (10%)
None of these/Other	211 (3.1%)
(Missing)	8 (0.1%)
**Religious service attendance**	
At least 1/week	4,049 (59%)
1/week	1,895 (28%)
1-3/month	531 (7.8%)
A few times a year	254 (3.7%)
Never	77 (1.1%)
(Missing)	20 (0.3%)
**Education**	
Up to 8 years	2,575 (38%)

Characteristic	N = 6,827[1]
9-15 years	4,120 (60%)
16+ years	130 (1.9%)
(Missing)	2 (<0.1%)
**Immigration**	
Born in this country	6,779 (99%)
Born in another country	47 (0.7%)
(Missing)	1 (<0.1%)
**Religious affiliation**	
Christianity	3,476 (51%)
Islam	3,302 (48%)
Hinduism	0 (0%)
Buddhism	0 (0%)
Judaism	0 (0%)
Sikhism	0 (0%)
Baha'i	0 (0%)
Jainism	0 (0%)
Shinto	1 (<0.1%)
Taoism	0 (0%)
Confucianism	0 (<0.1%)
Primal, Animist, or Folk religion	24 (0.3%)
Spiritism	0 (0%)
Umbanda, Candomble, and other African-derived religions	0 (0%)
Chinese folk/traditional religion	0 (0%)
Some other religion	1 (<0.1%)
No religion/Atheist/Agnostic	15 (0.2%)
(Missing)	9 (0.1%)
**Race/Ethnicity**	
Edo	116 (1.7%)
Efik	48 (0.7%)
Fulani	266 (3.9%)
Hausa	2,342 (34%)
Ibibio	180 (2.6%)
Idoma	61 (0.9%)
Igala	77 (1.1%)
Igbo (Ibo)	1,111 (16%)
Ijaw	110 (1.6%)
Kanuri	31 (0.5%)
Other	1,014 (15%)
Tiv	198 (2.9%)
Urhobo	38 (0.6%)
Yoruba	1,230 (18%)
(Missing)	4 (<0.1%)

[1]n (%)

Table S13b. Proportions by demographic category for Nigeria

Variable	Category	Mean	95% CI	SE	Global p-value
Age group	18-24	1.00	(0.99, 1.00)	0.00	0.995
	25-29	0.99	(0.99, 1.00)	0.00	
	30-39	1.00	(0.99, 1.00)	0.00	
	40-49	0.99	(0.98, 1.00)	0.01	
	50-59	1.00	(1.00, 1.00)	0.00	
	60-69	1.00	(1.00, 1.00)	0.00	
	70-79	0.97	(0.91, 1.00)	0.03	
	80 or older	1.00	*	*	
Gender	Male	0.99	(0.99, 1.00)	0.00	0.001
	Female	1.00	(1.00, 1.00)	0.00	
	Other	1.00	*	*	
Marital status	Married	1.00	(0.99, 1.00)	0.00	< .001
	Separated	0.92	(0.83, 1.00)	0.04	
	Divorced	0.98	(0.95, 1.00)	0.01	
	Widowed	1.00	(1.00, 1.00)	0.00	
	Never	1.00	(0.99, 1.00)	0.00	
	Domestic Partner	1.00	*	*	
Employment	Employed for an employer	1.00	(0.99, 1.00)	0.00	0.276
	Self-employed	1.00	(0.99, 1.00)	0.00	
	Retired	0.98	(0.93, 1.00)	0.02	
	Student	1.00	(1.00, 1.00)	0.00	
	Homemaker	0.99	(0.98, 1.00)	0.01	
	Unemployed and looking for a job	0.99	(0.98, 1.00)	0.00	
	None of these/Other	0.98	(0.94, 1.00)	0.02	
Religious service attendance	At least 1/week	1.00	(1.00, 1.00)	0.00	0.066
	1/week	0.99	(0.99, 1.00)	0.00	
	1-3/month	0.99	(0.97, 1.00)	0.01	
	A few times a year	0.96	(0.93, 1.00)	0.02	
	Never	0.96	(0.92, 1.00)	0.02	
Education	Up to 8 years	0.99	(0.98, 1.00)	0.00	0.528
	9-15 years	1.00	(0.99, 1.00)	0.00	
	16+ years	1.00	(0.99, 1.00)	0.00	
Immigration status	Born in this country	0.99	(0.99, 1.00)	0.00	< .001

Variable	Category	Mean	95% CI	SE	Global p-value
	Born in another country	1.00	*	*	
Religious affiliation	Christianity	1.00	(0.99, 1.00)	0.00	1.000
	Islam	0.99	(0.99, 1.00)	0.00	
	Shinto	1.00	*	*	
	Confucianism	1.00	*	*	
	Primal, Animist, or Folk religion	1.00	*	*	
	Some other religion	1.00	*	*	
	No religion/Atheist/Agnostic	1.00	*	*	
Race/ethnicity	Hausa	0.99	(0.98, 1.00)	0.00	1.000
	Yoruba	0.99	(0.98, 1.00)	0.00	
	Igbo (Ibo)	1.00	(1.00, 1.00)	0.00	
	Edo	1.00	*	*	
	Urhobo	0.99	(0.97, 1.00)	0.01	
	Fulani	1.00	(1.00, 1.00)	0.00	
	Kanuri	1.00	*	*	
	Tiv	0.99	(0.98, 1.00)	0.00	
	Efik	0.98	(0.94, 1.00)	0.02	
	Ijaw	1.00	(1.00, 1.00)	0.00	
	Igala	0.98	(0.94, 1.00)	0.02	
	Ibibio	1.00	*	*	
	Idoma	1.00	*	*	
	Other	1.00	(0.99, 1.00)	0.00	

Table S14a. Nationally representative descriptive statistics for Philippines

Characteristic	N = 5,292[1]
**Age group**	
18-24	1,073 (20%)
25-29	695 (13%)
30-39	1,160 (22%)
40-49	972 (18%)
50-59	732 (14%)
60-69	495 (9.4%)
70-79	143 (2.7%)
80 or older	23 (0.4%)
(Missing)	0 (0%)
**Gender**	
Male	2,625 (50%)
Female	2,643 (50%)
Other	13 (0.2%)
(Missing)	11 (0.2%)
**Marital status**	
Married	2,385 (45%)
Separated	249 (4.7%)
Divorced	9 (0.2%)
Widowed	274 (5.2%)
Never	1,206 (23%)
Domestic Partner	1,152 (22%)
(Missing)	16 (0.3%)
**Employment**	
Employed for an employer	1,350 (26%)
Self-employed	1,379 (26%)
Retired	158 (3.0%)
Student	585 (11%)
Homemaker	1,049 (20%)
Unemployed and looking for a job	658 (12%)
None of these/Other	113 (2.1%)
(Missing)	0 (0%)
**Religious service attendance**	
At least 1/week	844 (16%)
1/week	1,929 (36%)
1-3/month	1,374 (26%)
A few times a year	929 (18%)
Never	210 (4.0%)
(Missing)	6 (0.1%)
**Education**	
Up to 8 years	1,188 (22%)

Characteristic	N = 5,292[1]
9-15 years	3,722 (70%)
16+ years	381 (7.2%)
(Missing)	1 (<0.1%)
**Immigration**	
Born in this country	5,284 (100%)
Born in another country	8 (0.1%)
(Missing)	0 (0%)
**Religious affiliation**	
Christianity	4,914 (93%)
Islam	297 (5.6%)
Hinduism	0 (0%)
Buddhism	4 (<0.1%)
Judaism	4 (<0.1%)
Sikhism	0 (0%)
Baha'i	1 (<0.1%)
Jainism	0 (0%)
Shinto	0 (0%)
Taoism	0 (0%)
Confucianism	0 (0%)
Primal, Animist, or Folk religion	5 (<0.1%)
Spiritism	0 (0%)
Umbanda, Candomble, and other African-derived religions	0 (0%)
Chinese folk/traditional religion	0 (0%)
Some other religion	35 (0.7%)
No religion/Atheist/Agnostic	23 (0.4%)
(Missing)	9 (0.2%)
**Race/Ethnicity**	
Aeta	1 (<0.1%)
Badjao	2 (<0.1%)
Bicolano/Bikolano	300 (5.7%)
Cebuano	656 (12%)
Chinese-Filipino	3 (<0.1%)
Igorot	42 (0.8%)
Ilocano/Ilokano	429 (8.1%)
Ilonggo/Hiligaynon	428 (8.1%)
Kapampangan	107 (2.0%)
Maguindanaoan	84 (1.6%)
Mangyan	2 (<0.1%)
Maranao	39 (0.7%)
Masbateno	54 (1.0%)
Other	244 (4.6%)
Pangasinense	107 (2.0%)
Tagalog	1,691 (32%)

Characteristic	N = 5,292[1]
Tausug	94 (1.8%)
Visayan/Bisaya	739 (14%)
Waray	216 (4.1%)
Zamboangueno	51 (1.0%)
(Missing)	3 (<0.1%)

[1]n (%)

Table S14b. Proportions by demographic category for Philippines

Variable	Category	Mean	95% CI	SE	Global p-value
Age group	18-24	0.91	(0.88, 0.93)	0.01	0.031
	25-29	0.92	(0.89, 0.95)	0.01	
	30-39	0.93	(0.92, 0.95)	0.01	
	40-49	0.92	(0.90, 0.94)	0.01	
	50-59	0.94	(0.91, 0.96)	0.01	
	60-69	0.94	(0.92, 0.97)	0.01	
	70-79	0.97	(0.95, 1.00)	0.01	
	80 or older	0.90	(0.71, 1.00)	0.09	
Gender	Male	0.92	(0.90, 0.93)	0.01	0.061
	Female	0.94	(0.93, 0.95)	0.00	
	Other	0.85	(0.62, 1.00)	0.10	
Marital status	Married	0.94	(0.93, 0.95)	0.01	0.111
	Separated	0.93	(0.89, 0.98)	0.02	
	Divorced	0.90	(0.44, 1.00)	0.10	
	Widowed	0.95	(0.92, 0.98)	0.02	
	Never	0.90	(0.87, 0.93)	0.01	
	Domestic Partner	0.93	(0.91, 0.94)	0.01	
Employment	Employed for an employer	0.93	(0.91, 0.95)	0.01	0.452
	Self-employed	0.93	(0.91, 0.95)	0.01	
	Retired	0.96	(0.91, 1.00)	0.02	
	Student	0.91	(0.87, 0.94)	0.02	
	Homemaker	0.93	(0.92, 0.95)	0.01	
	Unemployed and looking for a job	0.92	(0.89, 0.94)	0.01	
	None of these/Other	0.91	(0.85, 0.97)	0.03	
Religious service attendance	At least 1/week	0.96	(0.95, 0.98)	0.01	< .001
	1/week	0.94	(0.92, 0.95)	0.01	
	1-3/month	0.93	(0.92, 0.95)	0.01	
	A few times a year	0.91	(0.89, 0.94)	0.01	
	Never	0.70	(0.61, 0.78)	0.04	
Education	Up to 8 years	0.91	(0.88, 0.93)	0.01	0.027
	9-15 years	0.93	(0.92, 0.94)	0.01	
	16+ years	0.96	(0.93, 0.99)	0.01	
Immigration status	Born in this country	0.93	(0.92, 0.94)	0.01	0.798

Variable	Category	Mean	95% CI	SE	Global p-value
	Born in another country	0.94	(0.77, 1.00)	0.06	
Religious affiliation	Christianity	0.93	(0.92, 0.94)	0.01	0.055
	Islam	0.92	(0.88, 0.97)	0.02	
	Buddhism	0.51	*	*	
	Judaism	1.00	*	*	
	Baha'i	1.00	*	*	
	Primal, Animist, or Folk religion	1.00	*	*	
	Some other religion	0.94	(0.87, 1.00)	0.03	
	No religion/Atheist/Agnostic	0.70	(0.43, 0.97)	0.12	
Race/ethnicity	Tagalog	0.92	(0.91, 0.94)	0.01	0.040
	Cebuano	0.90	(0.87, 0.93)	0.02	
	Ilocano/Ilokano	0.93	(0.89, 0.96)	0.02	
	Visayan/Bisaya	0.94	(0.92, 0.96)	0.01	
	Ilonggo/Hiligaynon	0.95	(0.93, 0.98)	0.01	
	Bicolano/Bikolano	0.95	(0.91, 0.98)	0.02	
	Waray	0.94	(0.91, 0.98)	0.02	
	Tausug	0.94	(0.89, 0.99)	0.03	
	Maranao	0.95	(0.88, 1.00)	0.03	
	Maguindanaoan	0.93	(0.82, 1.00)	0.05	
	Chinese-Filipino	0.87	*	*	
	Kapampangan	0.90	(0.83, 0.97)	0.04	
	Pangasinense	0.90	(0.80, 0.99)	0.05	
	Zamboangueno	0.88	(0.73, 1.00)	0.07	
	Masbateno	0.93	(0.86, 1.00)	0.03	
	Aeta	1.00	*	*	
	Igorot	0.97	(0.94, 1.00)	0.02	
	Mangyan	1.00	*	*	
	Badjao	1.00	*	*	
	Other	0.90	(0.87, 0.93)	0.02	

Table S15a. Nationally representative descriptive statistics for Poland

**Characteristic**	**N = 10,389**[1]
**Age group**	
18-24	955 (9.2%)
25-29	761 (7.3%)
30-39	2,159 (21%)
40-49	1,956 (19%)
50-59	1,670 (16%)
60-69	1,909 (18%)
70-79	833 (8.0%)
80 or older	145 (1.4%)
(Missing)	1 (<0.1%)
**Gender**	
Male	4,974 (48%)
Female	5,387 (52%)
Other	3 (<0.1%)
(Missing)	26 (0.2%)
**Marital status**	
Married	6,065 (58%)
Separated	111 (1.1%)
Divorced	529 (5.1%)
Widowed	990 (9.5%)
Never	1,811 (17%)
Domestic Partner	504 (4.8%)
(Missing)	379 (3.6%)
**Employment**	
Employed for an employer	5,837 (56%)
Self-employed	686 (6.6%)
Retired	2,434 (23%)
Student	515 (5.0%)
Homemaker	338 (3.3%)
Unemployed and looking for a job	284 (2.7%)
None of these/Other	169 (1.6%)
(Missing)	126 (1.2%)
**Religious service attendance**	
At least 1/week	305 (2.9%)
1/week	3,263 (31%)
1-3/month	2,081 (20%)
A few times a year	3,064 (29%)
Never	1,597 (15%)
(Missing)	78 (0.8%)
**Education**	
Up to 8 years	1,238 (12%)

Characteristic	N = 10,389[1]
9-15 years	6,130 (59%)
16+ years	3,020 (29%)
(Missing)	1 (<0.1%)
**Immigration**	
Born in this country	10,258 (99%)
Born in another country	108 (1.0%)
(Missing)	23 (0.2%)
**Religious affiliation**	
Christianity	9,378 (90%)
Islam	2 (<0.1%)
Hinduism	0 (0%)
Buddhism	2 (<0.1%)
Judaism	0 (0%)
Sikhism	1 (<0.1%)
Baha'i	0 (0%)
Jainism	3 (<0.1%)
Shinto	1 (<0.1%)
Taoism	0 (0%)
Confucianism	0 (0%)
Primal, Animist, or Folk religion	11 (0.1%)
Spiritism	0 (0%)
Umbanda, Candomble, and other African-derived religions	0 (0%)
Chinese folk/traditional religion	0 (0%)
Some other religion	0 (0%)
No religion/Atheist/Agnostic	942 (9.1%)
(Missing)	50 (0.5%)
**Race/Ethnicity**	
Belarussian	2 (<0.1%)
German	4 (<0.1%)
Kashubians	3 (<0.1%)
Other	4 (<0.1%)
Polish	10,309 (99%)
Silesia	14 (0.1%)
Ukrainian	38 (0.4%)
(Missing)	14 (0.1%)

[1]n (%)

Table S15b. Proportions by demographic category for Poland

Variable	Category	Mean	95% CI	SE	Global p-value
Age group	18-24	0.70	(0.65, 0.76)	0.03	< .001
	25-29	0.73	(0.69, 0.77)	0.02	
	30-39	0.80	(0.77, 0.83)	0.01	
	40-49	0.83	(0.80, 0.86)	0.01	
	50-59	0.87	(0.85, 0.90)	0.01	
	60-69	0.88	(0.85, 0.90)	0.01	
	70-79	0.90	(0.85, 0.95)	0.02	
	80 or older	0.89	(0.81, 0.97)	0.04	
Gender	Male	0.78	(0.76, 0.81)	0.01	< .001
	Female	0.86	(0.85, 0.88)	0.01	
	Other	0.60	*	*	
Marital status	Married	0.87	(0.85, 0.89)	0.01	< .001
	Separated	0.76	(0.58, 0.93)	0.09	
	Divorced	0.81	(0.77, 0.85)	0.02	
	Widowed	0.89	(0.85, 0.93)	0.02	
	Never	0.70	(0.66, 0.74)	0.02	
	Domestic Partner	0.64	(0.58, 0.69)	0.03	
Employment	Employed for an employer	0.83	(0.81, 0.85)	0.01	< .001
	Self-employed	0.71	(0.65, 0.77)	0.03	
	Retired	0.89	(0.86, 0.91)	0.01	
	Student	0.71	(0.63, 0.78)	0.04	
	Homemaker	0.80	(0.70, 0.91)	0.05	
	Unemployed and looking for a job	0.75	(0.66, 0.84)	0.05	
	None of these/Other	0.75	(0.64, 0.86)	0.06	
Religious service attendance	At least 1/week	0.98	(0.96, 1.00)	0.01	< .001
	1/week	0.99	(0.98, 0.99)	0.00	
	1-3/month	0.96	(0.95, 0.97)	0.01	
	A few times a year	0.81	(0.78, 0.84)	0.02	
	Never	0.33	(0.28, 0.37)	0.02	
Education	Up to 8 years	0.85	(0.78, 0.91)	0.03	< .001
	9-15 years	0.84	(0.83, 0.86)	0.01	
	16+ years	0.78	(0.76, 0.80)	0.01	
Immigration status	Born in this country	0.83	(0.81, 0.84)	0.01	0.897

Variable	Category	Mean	95% CI	SE	Global p-value
	Born in another country	0.82	(0.72, 0.92)	0.05	
Religious affiliation	Christianity	0.87	(0.85, 0.89)	0.01	< .001
	Islam	1.00	*	*	
	Buddhism	0.42	*	*	
	Sikhism	1.00	*	*	
	Jainism	1.00	*	*	
	Shinto	1.00	*	*	
	Primal, Animist, or Folk religion	0.56	(0.00, 1.00)	0.23	
	No religion/Atheist/Agnostic	0.41	(0.35, 0.47)	0.03	
Race/ethnicity	Polish	0.83	(0.81, 0.84)	0.01	0.186
	German	0.85	*	*	
	Belarussian	0.79	*	*	
	Ukrainian	0.91	(0.83, 0.99)	0.04	
	Silesia	0.88	(0.55, 1.00)	0.13	
	Kashubians	0.50	*	*	
	Other	0.77	(0.33, 1.00)	0.21	

Table S16a. Nationally representative descriptive statistics for South Africa

Characteristic	N = 2,651[1]
**Age group**	
18-24	461 (17%)
25-29	364 (14%)
30-39	655 (25%)
40-49	522 (20%)
50-59	309 (12%)
60-69	195 (7.4%)
70-79	120 (4.5%)
80 or older	17 (0.6%)
(Missing)	9 (0.3%)
**Gender**	
Male	1,288 (49%)
Female	1,356 (51%)
Other	2 (<0.1%)
(Missing)	4 (0.2%)
**Marital status**	
Married	539 (20%)
Separated	76 (2.9%)
Divorced	51 (1.9%)
Widowed	133 (5.0%)
Never	1,561 (59%)
Domestic Partner	264 (10.0%)
(Missing)	28 (1.0%)
**Employment**	
Employed for an employer	569 (21%)
Self-employed	412 (16%)
Retired	243 (9.2%)
Student	204 (7.7%)
Homemaker	137 (5.2%)
Unemployed and looking for a job	1,008 (38%)
None of these/Other	74 (2.8%)
(Missing)	3 (0.1%)
**Religious service attendance**	
At least 1/week	414 (16%)
1/week	891 (34%)
1-3/month	574 (22%)
A few times a year	431 (16%)
Never	334 (13%)
(Missing)	7 (0.3%)
**Education**	
Up to 8 years	668 (25%)

Characteristic	N = 2,651[1]
9-15 years	1,796 (68%)
16+ years	183 (6.9%)
(Missing)	4 (0.2%)
**Immigration**	
Born in this country	2,511 (95%)
Born in another country	139 (5.2%)
(Missing)	1 (<0.1%)
**Religious affiliation**	
Christianity	2,163 (82%)
Islam	62 (2.3%)
Hinduism	1 (<0.1%)
Buddhism	12 (0.5%)
Judaism	0 (0%)
Sikhism	0 (0%)
Baha'i	0 (0%)
Jainism	2 (<0.1%)
Shinto	2 (<0.1%)
Taoism	1 (<0.1%)
Confucianism	0 (0%)
Primal, Animist, or Folk religion	127 (4.8%)
Spiritism	0 (0%)
Umbanda, Candomble, and other African-derived religions	0 (0%)
Chinese folk/traditional religion	0 (0%)
Some other religion	5 (0.2%)
No religion/Atheist/Agnostic	253 (9.6%)
(Missing)	23 (0.9%)
**Race/Ethnicity**	
Asian/Indian	6 (0.2%)
Black	2,381 (90%)
Colored	252 (9.5%)
Other	1 (<0.1%)
White	8 (0.3%)
(Missing)	3 (0.1%)

[1]n (%)

Table S16b. Proportions by demographic category for South Africa

Variable	Category	Mean	95% CI	SE	Global p-value
Age group	18-24	0.93	(0.90, 0.96)	0.01	0.350
	25-29	0.96	(0.93, 0.98)	0.01	
	30-39	0.94	(0.92, 0.96)	0.01	
	40-49	0.96	(0.94, 0.98)	0.01	
	50-59	0.98	(0.94, 1.00)	0.02	
	60-69	1.00	(0.99, 1.00)	0.00	
	70-79	1.00	*	*	
	80 or older	1.00	(1.00, 1.00)	0.00	
Gender	Male	0.94	(0.92, 0.96)	0.01	< .001
	Female	0.97	(0.96, 0.98)	0.00	
	Other	1.00	*	*	
Marital status	Married	0.98	(0.97, 1.00)	0.01	< .001
	Separated	0.90	(0.78, 1.00)	0.06	
	Divorced	0.92	(0.84, 1.00)	0.04	
	Widowed	1.00	(0.99, 1.00)	0.00	
	Never	0.95	(0.94, 0.96)	0.01	
	Domestic Partner	0.94	(0.90, 0.97)	0.02	
Employment	Employed for an employer	0.94	(0.92, 0.96)	0.01	< .001
	Self-employed	0.94	(0.90, 0.97)	0.02	
	Retired	1.00	*	*	
	Student	0.96	(0.92, 0.99)	0.02	
	Homemaker	0.98	(0.95, 1.00)	0.01	
	Unemployed and looking for a job	0.96	(0.95, 0.97)	0.01	
	None of these/Other	0.98	(0.96, 1.00)	0.01	
Religious service attendance	At least 1/week	0.99	(0.98, 1.00)	0.01	< .001
	1/week	0.98	(0.97, 0.99)	0.00	
	1-3/month	0.97	(0.95, 0.99)	0.01	
	A few times a year	0.94	(0.91, 0.96)	0.01	
	Never	0.86	(0.82, 0.91)	0.02	
Education	Up to 8 years	0.98	(0.96, 1.00)	0.01	0.047
	9-15 years	0.95	(0.94, 0.96)	0.01	
	16+ years	0.94	(0.91, 0.98)	0.02	
Immigration status	Born in this country	0.96	(0.95, 0.97)	0.01	0.847

Variable	Category	Mean	95% CI	SE	Global p-value
	Born in another country	0.95	(0.90, 1.00)	0.03	
Religious affiliation	Christianity	0.96	(0.95, 0.97)	0.01	0.002
	Islam	0.99	(0.97, 1.00)	0.01	
	Hinduism	1.00	*	*	
	Buddhism	0.94	(0.39, 1.00)	0.05	
	Jainism	1.00	*	*	
	Shinto	1.00	*	*	
	Taoism	0.00	*	*	
	Primal, Animist, or Folk religion	0.92	(0.86, 0.97)	0.03	
	Some other religion	0.84	*	*	
	No religion/Atheist/Agnostic	0.92	(0.87, 0.96)	0.02	
Race/ethnicity	Black	0.96	(0.95, 0.97)	0.01	0.998
	White	1.00	*	*	
	Asian/Indian	1.00	*	*	
	Colored	0.96	(0.92, 0.99)	0.02	
	Other	1.00	*	*	

Table S17a. Nationally representative descriptive statistics for Spain

**Characteristic**	**N = 6,290**[1]
**Age group**	
18-24	594 (9.4%)
25-29	450 (7.2%)
30-39	1,111 (18%)
40-49	1,396 (22%)
50-59	1,252 (20%)
60-69	977 (16%)
70-79	467 (7.4%)
80 or older	43 (0.7%)
(Missing)	0 (0%)
**Gender**	
Male	3,142 (50%)
Female	3,119 (50%)
Other	6 (0.1%)
(Missing)	22 (0.4%)
**Marital status**	
Married	2,947 (47%)
Separated	237 (3.8%)
Divorced	518 (8.2%)
Widowed	189 (3.0%)
Never	1,742 (28%)
Domestic Partner	589 (9.4%)
(Missing)	67 (1.1%)
**Employment**	
Employed for an employer	2,862 (45%)
Self-employed	576 (9.2%)
Retired	1,278 (20%)
Student	448 (7.1%)
Homemaker	345 (5.5%)
Unemployed and looking for a job	646 (10%)
None of these/Other	123 (2.0%)
(Missing)	11 (0.2%)
**Religious service attendance**	
At least 1/week	317 (5.0%)
1/week	662 (11%)
1-3/month	437 (6.9%)
A few times a year	1,972 (31%)
Never	2,875 (46%)
(Missing)	27 (0.4%)
**Education**	
Up to 8 years	802 (13%)

**Characteristic**	**N = 6,290**[1]
9-15 years	4,145 (66%)
16+ years	1,341 (21%)
(Missing)	2 (<0.1%)
**Immigration**	
Born in this country	5,479 (87%)
Born in another country	788 (13%)
(Missing)	23 (0.4%)
**Religious affiliation**	
Christianity	4,074 (65%)
Islam	135 (2.1%)
Hinduism	7 (0.1%)
Buddhism	36 (0.6%)
Judaism	4 (<0.1%)
Sikhism	3 (<0.1%)
Baha'i	2 (<0.1%)
Jainism	1 (<0.1%)
Shinto	0 (0%)
Taoism	5 (<0.1%)
Confucianism	3 (<0.1%)
Primal, Animist, or Folk religion	7 (0.1%)
Spiritism	0 (0%)
Umbanda, Candomble, and other African-derived religions	0 (0%)
Chinese folk/traditional religion	0 (0%)
Some other religion	27 (0.4%)
No religion/Atheist/Agnostic	1,932 (31%)
(Missing)	55 (0.9%)

[1]n (%)

Table S17b. Proportions by demographic category for Spain

Variable	Category	Mean	95% CI	SE	Global p-value
Age group	18-24	0.64	(0.59, 0.69)	0.03	< .001
	25-29	0.62	(0.57, 0.67)	0.03	
	30-39	0.62	(0.59, 0.65)	0.02	
	40-49	0.63	(0.60, 0.66)	0.01	
	50-59	0.65	(0.61, 0.68)	0.02	
	60-69	0.61	(0.56, 0.66)	0.03	
	70-79	0.71	(0.64, 0.79)	0.04	
	80 or older	0.89	(0.78, 0.99)	0.05	
Gender	Male	0.62	(0.60, 0.65)	0.01	0.007
	Female	0.65	(0.63, 0.67)	0.01	
	Other	0.87	(0.42, 1.00)	0.09	
Marital status	Married	0.66	(0.63, 0.68)	0.01	0.007
	Separated	0.66	(0.58, 0.74)	0.04	
	Divorced	0.63	(0.57, 0.69)	0.03	
	Widowed	0.74	(0.64, 0.84)	0.05	
	Never	0.59	(0.57, 0.62)	0.01	
	Domestic Partner	0.63	(0.58, 0.67)	0.02	
Employment	Employed for an employer	0.60	(0.58, 0.62)	0.01	< .001
	Self-employed	0.67	(0.62, 0.71)	0.02	
	Retired	0.68	(0.64, 0.72)	0.02	
	Student	0.60	(0.54, 0.66)	0.03	
	Homemaker	0.75	(0.69, 0.82)	0.03	
	Unemployed and looking for a job	0.66	(0.61, 0.70)	0.02	
	None of these/Other	0.57	(0.48, 0.67)	0.05	
Religious service attendance	At least 1/week	0.89	(0.84, 0.93)	0.02	< .001
	1/week	0.91	(0.88, 0.93)	0.01	
	1-3/month	0.86	(0.83, 0.90)	0.02	
	A few times a year	0.76	(0.74, 0.79)	0.01	
	Never	0.43	(0.40, 0.45)	0.01	
Education	Up to 8 years	0.72	(0.67, 0.78)	0.03	< .001
	9-15 years	0.64	(0.62, 0.65)	0.01	
	16+ years	0.59	(0.56, 0.62)	0.02	
Immigration status	Born in this country	0.61	(0.60, 0.63)	0.01	< .001

Variable	Category	Mean	95% CI	SE	Global p-value
	Born in another country	0.79	(0.76, 0.82)	0.02	
Religious affiliation	Christianity	0.79	(0.77, 0.81)	0.01	< .001
	Islam	0.85	(0.77, 0.92)	0.04	
	Hinduism	0.81	*	*	
	Buddhism	0.79	(0.62, 0.97)	0.08	
	Judaism	0.81	*	*	
	Sikhism	0.71	*	*	
	Baha'i	0.40	*	*	
	Jainism	1.00	*	*	
	Taoism	0.42	*	*	
	Confucianism	0.72	*	*	
	Primal, Animist, or Folk religion	0.94	*	*	
	Some other religion	0.69	(0.41, 0.97)	0.13	
	No religion/Atheist/Agnostic	0.30	(0.27, 0.32)	0.01	

Table S18a. Nationally representative descriptive statistics for Sweden

Characteristic	N = 15,068[1]
**Age group**	
18-24	1,515 (10%)
25-29	1,399 (9.3%)
30-39	2,398 (16%)
40-49	2,221 (15%)
50-59	2,493 (17%)
60-69	2,168 (14%)
70-79	2,253 (15%)
80 or older	621 (4.1%)
(Missing)	0 (0%)
**Gender**	
Male	7,536 (50%)
Female	7,493 (50%)
Other	27 (0.2%)
(Missing)	12 (<0.1%)
**Marital status**	
Married	6,408 (43%)
Separated	426 (2.8%)
Divorced	801 (5.3%)
Widowed	433 (2.9%)
Never	3,854 (26%)
Domestic Partner	3,073 (20%)
(Missing)	72 (0.5%)
**Employment**	
Employed for an employer	7,907 (52%)
Self-employed	1,243 (8.3%)
Retired	3,832 (25%)
Student	1,332 (8.8%)
Homemaker	75 (0.5%)
Unemployed and looking for a job	324 (2.2%)
None of these/Other	337 (2.2%)
(Missing)	18 (0.1%)
**Religious service attendance**	
At least 1/week	236 (1.6%)
1/week	434 (2.9%)
1-3/month	486 (3.2%)
A few times a year	3,950 (26%)
Never	9,918 (66%)
(Missing)	45 (0.3%)
**Education**	
Up to 8 years	252 (1.7%)

Characteristic	N = 15,068[1]
9-15 years	10,790 (72%)
16+ years	4,026 (27%)
(Missing)	0 (0%)
**Immigration**	
Born in this country	13,922 (92%)
Born in another country	1,052 (7.0%)
(Missing)	94 (0.6%)
**Religious affiliation**	
Christianity	8,346 (55%)
Islam	470 (3.1%)
Hinduism	22 (0.1%)
Buddhism	110 (0.7%)
Judaism	54 (0.4%)
Sikhism	4 (<0.1%)
Baha'i	6 (<0.1%)
Jainism	0 (0%)
Shinto	0 (<0.1%)
Taoism	4 (<0.1%)
Confucianism	0 (0%)
Primal, Animist, or Folk religion	83 (0.5%)
Spiritism	0 (0%)
Umbanda, Candomble, and other African-derived religions	0 (0%)
Chinese folk/traditional religion	0 (0%)
Some other religion	198 (1.3%)
No religion/Atheist/Agnostic	5,697 (38%)
(Missing)	74 (0.5%)

[1]n (%)

Table S18b. Proportions by demographic category for Sweden

Variable	Category	Mean	95% CI	SE	Global p-value
Age group	18-24	0.46	(0.43, 0.49)	0.01	< .001
	25-29	0.43	(0.40, 0.46)	0.02	
	30-39	0.37	(0.35, 0.40)	0.01	
	40-49	0.41	(0.38, 0.44)	0.01	
	50-59	0.45	(0.43, 0.48)	0.01	
	60-69	0.46	(0.44, 0.49)	0.01	
	70-79	0.48	(0.46, 0.51)	0.01	
	80 or older	0.47	(0.42, 0.52)	0.02	
Gender	Male	0.38	(0.37, 0.40)	0.01	< .001
	Female	0.50	(0.48, 0.51)	0.01	
	Other	0.65	(0.44, 0.85)	0.10	
Marital status	Married	0.46	(0.44, 0.47)	0.01	< .001
	Separated	0.52	(0.46, 0.57)	0.03	
	Divorced	0.50	(0.46, 0.54)	0.02	
	Widowed	0.49	(0.43, 0.54)	0.03	
	Never	0.42	(0.40, 0.44)	0.01	
	Domestic Partner	0.39	(0.37, 0.41)	0.01	
Employment	Employed for an employer	0.40	(0.39, 0.42)	0.01	< .001
	Self-employed	0.45	(0.41, 0.49)	0.02	
	Retired	0.48	(0.46, 0.50)	0.01	
	Student	0.47	(0.44, 0.50)	0.02	
	Homemaker	0.66	(0.53, 0.78)	0.06	
	Unemployed and looking for a job	0.51	(0.45, 0.57)	0.03	
	None of these/Other	0.53	(0.47, 0.59)	0.03	
Religious service attendance	At least 1/week	0.99	(0.98, 1.00)	0.00	< .001
	1/week	0.96	(0.94, 0.98)	0.01	
	1-3/month	0.90	(0.87, 0.93)	0.02	
	A few times a year	0.63	(0.61, 0.65)	0.01	
	Never	0.31	(0.29, 0.32)	0.01	
Education	Up to 8 years	0.46	(0.39, 0.54)	0.04	0.279
	9-15 years	0.43	(0.42, 0.45)	0.01	
	16+ years	0.45	(0.43, 0.47)	0.01	
Immigration status	Born in this country	0.43	(0.42, 0.44)	0.01	< .001

Variable	Category	Mean	95% CI	SE	Global p-value
	Born in another country	0.63	(0.59, 0.66)	0.02	
Religious affiliation	Christianity	0.58	(0.57, 0.59)	0.01	1.000
	Islam	0.93	(0.89, 0.96)	0.02	
	Hinduism	0.65	(0.27, 1.00)	0.16	
	Buddhism	0.75	(0.63, 0.88)	0.06	
	Judaism	0.57	(0.36, 0.78)	0.10	
	Sikhism	1.00	*	*	
	Baha'i	1.00	*	*	
	Shinto	1.00	*	*	
	Taoism	1.00	*	*	
	Primal, Animist, or Folk religion	0.77	(0.63, 0.91)	0.07	
	Some other religion	0.69	(0.59, 0.79)	0.05	
	No religion/Atheist/Agnostic	0.17	(0.16, 0.18)	0.01	

Table S19a. Nationally representative descriptive statistics for Tanzania

Characteristic	N = 9,075[1]
**Age group**	
18-24	2,284 (25%)
25-29	1,349 (15%)
30-39	2,060 (23%)
40-49	1,503 (17%)
50-59	912 (10%)
60-69	575 (6.3%)
70-79	297 (3.3%)
80 or older	93 (1.0%)
(Missing)	2 (<0.1%)
**Gender**	
Male	4,299 (47%)
Female	4,776 (53%)
Other	0 (0%)
(Missing)	0 (0%)
**Marital status**	
Married	5,577 (61%)
Separated	404 (4.5%)
Divorced	103 (1.1%)
Widowed	450 (5.0%)
Never	2,260 (25%)
Domestic Partner	275 (3.0%)
(Missing)	7 (<0.1%)
**Employment**	
Employed for an employer	513 (5.6%)
Self-employed	4,625 (51%)
Retired	139 (1.5%)
Student	319 (3.5%)
Homemaker	1,796 (20%)
Unemployed and looking for a job	1,491 (16%)
None of these/Other	186 (2.1%)
(Missing)	6 (<0.1%)
**Religious service attendance**	
At least 1/week	2,622 (29%)
1/week	4,268 (47%)
1-3/month	1,082 (12%)
A few times a year	814 (9.0%)
Never	288 (3.2%)
(Missing)	1 (<0.1%)
**Education**	
Up to 8 years	6,699 (74%)

Characteristic	N = 9,075[1]
9-15 years	2,252 (25%)
16+ years	122 (1.3%)
(Missing)	2 (<0.1%)
**Immigration**	
Born in this country	9,048 (100%)
Born in another country	25 (0.3%)
(Missing)	1 (<0.1%)
**Religious affiliation**	
Christianity	5,647 (62%)
Islam	3,189 (35%)
Hinduism	0 (0%)
Buddhism	0 (0%)
Judaism	0 (0%)
Sikhism	0 (0%)
Baha'i	0 (0%)
Jainism	0 (0%)
Shinto	0 (0%)
Taoism	1 (<0.1%)
Confucianism	0 (0%)
Primal, Animist, or Folk religion	12 (0.1%)
Spiritism	0 (0%)
Umbanda, Candomble, and other African-derived religions	0 (0%)
Chinese folk/traditional religion	0 (0%)
Some other religion	0 (0%)
No religion/Atheist/Agnostic	216 (2.4%)
(Missing)	10 (0.1%)
**Race/Ethnicity**	
African	9,060 (100%)
Arab	11 (0.1%)
Indian	3 (<0.1%)
(Missing)	2 (<0.1%)

[1]n (%)

Table S19b. Proportions by demographic category for Tanzania

Variable	Category	Mean	95% CI	SE	Global p-value
Age group	18-24	0.97	(0.96, 0.98)	0.01	0.008
	25-29	0.98	(0.98, 0.99)	0.00	
	30-39	0.98	(0.98, 0.99)	0.00	
	40-49	0.98	(0.97, 0.99)	0.01	
	50-59	0.99	(0.98, 1.00)	0.00	
	60-69	0.99	(0.98, 1.00)	0.01	
	70-79	1.00	(0.99, 1.00)	0.00	
	80 or older	0.98	(0.95, 1.00)	0.02	
Gender	Male	0.98	(0.98, 0.99)	0.00	0.378
	Female	0.98	(0.97, 0.99)	0.00	
Marital status	Married	0.99	(0.98, 0.99)	0.00	0.001
	Separated	0.98	(0.96, 1.00)	0.01	
	Divorced	0.97	(0.93, 1.00)	0.02	
	Widowed	0.99	(0.99, 1.00)	0.00	
	Never	0.97	(0.96, 0.98)	0.00	
	Domestic Partner	0.95	(0.91, 0.98)	0.02	
Employment	Employed for an employer	0.99	(0.98, 1.00)	0.00	0.002
	Self-employed	0.99	(0.98, 0.99)	0.00	
	Retired	0.99	(0.98, 1.00)	0.01	
	Student	0.96	(0.94, 0.99)	0.01	
	Homemaker	0.99	(0.98, 0.99)	0.00	
	Unemployed and looking for a job	0.97	(0.96, 0.98)	0.01	
	None of these/Other	0.95	(0.90, 1.00)	0.03	
Religious service attendance	At least 1/week	0.99	(0.99, 1.00)	0.00	< .001
	1/week	0.98	(0.98, 0.99)	0.00	
	1-3/month	0.98	(0.97, 0.99)	0.01	
	A few times a year	0.97	(0.96, 0.99)	0.01	
	Never	0.92	(0.87, 0.96)	0.02	
Education	Up to 8 years	0.98	(0.98, 0.99)	0.00	0.971
	9-15 years	0.98	(0.97, 0.99)	0.00	
	16+ years	0.98	(0.95, 1.00)	0.01	
Immigration status	Born in this country	0.98	(0.98, 0.99)	0.00	0.465
	Born in another country	0.94	(0.81, 1.00)	0.06	

Variable	Category	Mean	95% CI	SE	Global p-value
Religious affiliation	Christianity	0.99	(0.98, 0.99)	0.00	0.787
	Islam	0.98	(0.97, 0.99)	0.00	
	Taoism	1.00	*	*	
	Primal, Animist, or Folk religion	1.00	*	*	
	No religion/Atheist/Agnostic	0.96	(0.92, 0.99)	0.02	
Race/ethnicity	Indian	1.00	*	*	0.996
	Arab	1.00	*	*	
	African	0.98	(0.98, 0.99)	0.00	

Table S20a. Nationally representative descriptive statistics for Turkey

Characteristic	N = 1,473[1]
**Age group**	
18-24	222 (15%)
25-29	152 (10%)
30-39	315 (21%)
40-49	312 (21%)
50-59	225 (15%)
60-69	164 (11%)
70-79	65 (4.4%)
80 or older	18 (1.2%)
(Missing)	0 (0%)
**Gender**	
Male	754 (51%)
Female	719 (49%)
Other	0 (0%)
(Missing)	0 (0%)
**Marital status**	
Married	936 (64%)
Separated	13 (0.9%)
Divorced	64 (4.3%)
Widowed	64 (4.3%)
Never	379 (26%)
Domestic Partner	0 (0%)
(Missing)	17 (1.1%)
**Employment**	
Employed for an employer	413 (28%)
Self-employed	255 (17%)
Retired	205 (14%)
Student	107 (7.3%)
Homemaker	347 (24%)
Unemployed and looking for a job	87 (5.9%)
None of these/Other	59 (4.0%)
(Missing)	0 (0%)
**Religious service attendance**	
At least 1/week	493 (33%)
1/week	271 (18%)
1-3/month	174 (12%)
A few times a year	255 (17%)
Never	274 (19%)
(Missing)	6 (0.4%)
**Education**	
Up to 8 years	436 (30%)

**Characteristic**	**N = 1,473**[1]
9-15 years	711 (48%)
16+ years	326 (22%)
(Missing)	0 (0%)
**Immigration**	
Born in this country	1,415 (96%)
Born in another country	58 (4.0%)
(Missing)	0 (0%)
**Religious affiliation**	
Christianity	2 (0.1%)
Islam	1,381 (94%)
Hinduism	0 (0%)
Buddhism	1 (<0.1%)
Judaism	1 (<0.1%)
Sikhism	1 (<0.1%)
Baha'i	0 (0%)
Jainism	0 (0%)
Shinto	0 (0%)
Taoism	0 (0%)
Confucianism	0 (0%)
Primal, Animist, or Folk religion	1 (<0.1%)
Spiritism	0 (0%)
Umbanda, Candomble, and other African-derived religions	0 (0%)
Chinese folk/traditional religion	0 (0%)
Some other religion	1 (<0.1%)
No religion/Atheist/Agnostic	66 (4.5%)
(Missing)	19 (1.3%)
**Race/Ethnicity**	
Albanian	8 (0.5%)
Arab	51 (3.5%)
Armenian	1 (<0.1%)
Azeri	9 (0.6%)
Bosnian	5 (0.3%)
Circassian	19 (1.3%)
Georgian	4 (0.3%)
Greek	1 (<0.1%)
Kurdish/Zaza	252 (17%)
Laz	25 (1.7%)
Other	58 (3.9%)
Turkish	1,030 (70%)
Uyghur	1 (<0.1%)
(Missing)	9 (0.6%)

[1]n (%)

Table S20b. Proportions by demographic category for Turkey

Variable	Category	Mean	95% CI	SE	Global p-value
Age group	18-24	0.78	(0.73, 0.83)	0.03	0.061
	25-29	0.89	(0.84, 0.94)	0.03	
	30-39	0.85	(0.80, 0.89)	0.02	
	40-49	0.89	(0.85, 0.92)	0.02	
	50-59	0.88	(0.83, 0.94)	0.03	
	60-69	0.84	(0.76, 0.93)	0.04	
	70-79	0.84	(0.69, 0.99)	0.07	
	80 or older	0.80	(0.45, 1.00)	0.15	
Gender	Male	0.83	(0.80, 0.86)	0.02	0.007
	Female	0.88	(0.85, 0.91)	0.01	
Marital status	Married	0.87	(0.84, 0.90)	0.01	0.006
	Separated	0.85	(0.64, 1.00)	0.09	
	Divorced	0.83	(0.72, 0.95)	0.06	
	Widowed	0.95	(0.88, 1.00)	0.04	
	Never	0.81	(0.77, 0.84)	0.02	
Employment	Employed for an employer	0.86	(0.83, 0.90)	0.02	0.015
	Self-employed	0.82	(0.77, 0.88)	0.03	
	Retired	0.84	(0.78, 0.91)	0.03	
	Student	0.77	(0.70, 0.84)	0.04	
	Homemaker	0.91	(0.87, 0.95)	0.02	
	Unemployed and looking for a job	0.81	(0.73, 0.90)	0.04	
	None of these/Other	0.80	(0.66, 0.95)	0.07	
Religious service attendance	At least 1/week	0.93	(0.91, 0.96)	0.01	< .001
	1/week	0.88	(0.83, 0.94)	0.03	
	1-3/month	0.93	(0.88, 0.98)	0.03	
	A few times a year	0.89	(0.85, 0.93)	0.02	
	Never	0.60	(0.53, 0.66)	0.03	
Education	Up to 8 years	0.88	(0.83, 0.93)	0.02	0.016
	9-15 years	0.86	(0.83, 0.89)	0.01	
	16+ years	0.80	(0.77, 0.84)	0.02	
Immigration status	Born in this country	0.85	(0.83, 0.87)	0.01	0.987
	Born in another country	0.85	(0.74, 0.96)	0.05	
Religious affiliation	Christianity	0.60	*	*	< .001

Variable	Category	Mean	95% CI	SE	Global p-value
	Islam	0.88	(0.86, 0.90)	0.01	
	Buddhism	0.00	*	*	
	Judaism	1.00	*	*	
	Sikhism	1.00	*	*	
	Primal, Animist, or Folk religion	0.43	*	*	
	Some other religion	0.00	*	*	
	No religion/Atheist/Agnostic	0.32	(0.22, 0.43)	0.05	
Race/ethnicity	Arab	0.89	(0.80, 0.99)	0.05	0.008
	Turkish	0.85	(0.83, 0.88)	0.01	
	Greek	0.57	*	*	
	Kurdish/Zaza	0.89	(0.84, 0.94)	0.02	
	Laz	0.72	(0.44, 0.99)	0.12	
	Circassian	0.77	(0.49, 1.00)	0.10	
	Bosnian	0.69	*	*	
	Armenian	1.00	*	*	
	Georgian	0.68	*	*	
	Uyghur	1.00	*	*	
	Albanian	1.00	*	*	
	Azeri	0.65	*	*	
	Other	0.78	(0.63, 0.92)	0.07	

Table S21a. Nationally representative descriptive statistics for United Kingdom

**Characteristic**	**N = 5,368**[1]
**Age group**	
18-24	490 (9.1%)
25-29	391 (7.3%)
30-39	946 (18%)
40-49	827 (15%)
50-59	949 (18%)
60-69	889 (17%)
70-79	711 (13%)
80 or older	163 (3.0%)
(Missing)	1 (<0.1%)
**Gender**	
Male	2,557 (48%)
Female	2,789 (52%)
Other	14 (0.3%)
(Missing)	9 (0.2%)
**Marital status**	
Married	2,510 (47%)
Separated	114 (2.1%)
Divorced	435 (8.1%)
Widowed	294 (5.5%)
Never	1,456 (27%)
Domestic Partner	512 (9.5%)
(Missing)	48 (0.9%)
**Employment**	
Employed for an employer	2,798 (52%)
Self-employed	469 (8.7%)
Retired	1,262 (24%)
Student	229 (4.3%)
Homemaker	184 (3.4%)
Unemployed and looking for a job	215 (4.0%)
None of these/Other	201 (3.7%)
(Missing)	11 (0.2%)
**Religious service attendance**	
At least 1/week	291 (5.4%)
1/week	499 (9.3%)
1-3/month	293 (5.5%)
A few times a year	1,165 (22%)
Never	3,110 (58%)
(Missing)	10 (0.2%)
**Education**	
Up to 8 years	1,314 (24%)

Characteristic	N = 5,368[1]
9-15 years	2,072 (39%)
16+ years	1,974 (37%)
(Missing)	8 (0.2%)
**Immigration**	
Born in this country	4,659 (87%)
Born in another country	682 (13%)
(Missing)	27 (0.5%)
**Religious affiliation**	
Christianity	2,750 (51%)
Islam	218 (4.1%)
Hinduism	61 (1.1%)
Buddhism	30 (0.6%)
Judaism	44 (0.8%)
Sikhism	29 (0.5%)
Baha'i	6 (0.1%)
Jainism	4 (<0.1%)
Shinto	0 (0%)
Taoism	4 (<0.1%)
Confucianism	2 (<0.1%)
Primal, Animist, or Folk religion	36 (0.7%)
Spiritism	0 (0%)
Umbanda, Candomble, and other African-derived religions	0 (0%)
Chinese folk/traditional religion	0 (0%)
Some other religion	61 (1.1%)
No religion/Atheist/Agnostic	2,099 (39%)
(Missing)	25 (0.5%)
**Race/Ethnicity**	
Asian	426 (7.9%)
Black	152 (2.8%)
Other	96 (1.8%)
White	4,647 (87%)
(Missing)	47 (0.9%)

[1]n (%)

Table S21b. Proportions by demographic category for United Kingdom

Variable	Category	Mean	95% CI	SE	Global p-value
Age group	18-24	0.52	(0.45, 0.60)	0.04	0.062
	25-29	0.59	(0.53, 0.66)	0.03	
	30-39	0.55	(0.51, 0.60)	0.02	
	40-49	0.56	(0.51, 0.60)	0.02	
	50-59	0.51	(0.46, 0.55)	0.02	
	60-69	0.51	(0.46, 0.55)	0.02	
	70-79	0.58	(0.52, 0.63)	0.03	
	80 or older	0.64	(0.53, 0.74)	0.05	
Gender	Male	0.52	(0.50, 0.55)	0.01	0.004
	Female	0.56	(0.54, 0.59)	0.01	
	Other	0.22	(0.00, 0.50)	0.12	
Marital status	Married	0.58	(0.56, 0.61)	0.01	< .001
	Separated	0.52	(0.40, 0.63)	0.06	
	Divorced	0.57	(0.50, 0.63)	0.03	
	Widowed	0.57	(0.49, 0.64)	0.04	
	Never	0.51	(0.47, 0.55)	0.02	
	Domestic Partner	0.41	(0.35, 0.47)	0.03	
Employment	Employed for an employer	0.55	(0.53, 0.58)	0.01	0.120
	Self-employed	0.54	(0.48, 0.60)	0.03	
	Retired	0.55	(0.51, 0.59)	0.02	
	Student	0.52	(0.42, 0.61)	0.05	
	Homemaker	0.60	(0.50, 0.71)	0.05	
	Unemployed and looking for a job	0.49	(0.39, 0.60)	0.05	
	None of these/Other	0.42	(0.32, 0.51)	0.05	
Religious service attendance	At least 1/week	0.97	(0.94, 1.00)	0.02	< .001
	1/week	0.93	(0.89, 0.96)	0.02	
	1-3/month	0.90	(0.85, 0.95)	0.02	
	A few times a year	0.71	(0.68, 0.75)	0.02	
	Never	0.34	(0.32, 0.37)	0.01	
Education	Up to 8 years	0.54	(0.49, 0.59)	0.03	< .001
	9-15 years	0.50	(0.48, 0.53)	0.01	
	16+ years	0.59	(0.56, 0.61)	0.01	
Immigration status	Born in this country	0.52	(0.50, 0.54)	0.01	< .001

Variable	Category	Mean	95% CI	SE	Global p-value
	Born in another country	0.67	(0.63, 0.72)	0.02	
Religious affiliation	Christianity	0.72	(0.70, 0.75)	0.01	< .001
	Islam	0.98	(0.96, 1.00)	0.01	
	Hinduism	0.92	(0.85, 1.00)	0.04	
	Buddhism	0.60	(0.39, 0.82)	0.10	
	Judaism	0.74	(0.57, 0.91)	0.08	
	Sikhism	0.84	(0.66, 1.00)	0.08	
	Baha'i	0.15	*	*	
	Jainism	1.00	*	*	
	Taoism	0.52	*	*	
	Confucianism	1.00	*	*	
	Primal, Animist, or Folk religion	0.74	(0.49, 0.99)	0.12	
	Some other religion	0.80	(0.69, 0.91)	0.05	
	No religion/Atheist/Agnostic	0.23	(0.21, 0.26)	0.01	
Race/ethnicity	Asian	0.82	(0.77, 0.87)	0.03	< .001
	Black	0.86	(0.79, 0.93)	0.03	
	White	0.50	(0.48, 0.52)	0.01	
	Other	0.68	(0.55, 0.82)	0.07	

Table S22a. Nationally representative descriptive statistics for United States

**Characteristic**	**N = 38,312**[1]
**Age group**	
18-24	2,682 (7.0%)
25-29	3,540 (9.2%)
30-39	7,284 (19%)
40-49	5,649 (15%)
50-59	6,745 (18%)
60-69	6,832 (18%)
70-79	4,054 (11%)
80 or older	1,525 (4.0%)
(Missing)	0 (0%)
**Gender**	
Male	18,222 (48%)
Female	19,562 (51%)
Other	392 (1.0%)
(Missing)	136 (0.4%)
**Marital status**	
Married	20,360 (53%)
Separated	727 (1.9%)
Divorced	3,636 (9.5%)
Widowed	1,978 (5.2%)
Never	9,431 (25%)
Domestic Partner	1,971 (5.1%)
(Missing)	207 (0.5%)
**Employment**	
Employed for an employer	19,502 (51%)
Self-employed	3,445 (9.0%)
Retired	9,016 (24%)
Student	1,145 (3.0%)
Homemaker	2,049 (5.3%)
Unemployed and looking for a job	1,777 (4.6%)
None of these/Other	1,292 (3.4%)
(Missing)	87 (0.2%)
**Religious service attendance**	
At least 1/week	2,633 (6.9%)
1/week	5,887 (15%)
1-3/month	2,819 (7.4%)
A few times a year	8,870 (23%)
Never	17,975 (47%)
(Missing)	128 (0.3%)
**Education**	
Up to 8 years	210 (0.5%)

Characteristic	N = 38,312[1]
9-15 years	25,322 (66%)
16+ years	12,705 (33%)
(Missing)	75 (0.2%)
**Immigration**	
Born in this country	34,865 (91%)
Born in another country	3,020 (7.9%)
(Missing)	427 (1.1%)
**Religious affiliation**	
Christianity	22,954 (60%)
Islam	205 (0.5%)
Hinduism	167 (0.4%)
Buddhism	336 (0.9%)
Judaism	638 (1.7%)
Sikhism	24 (<0.1%)
Baha'i	13 (<0.1%)
Jainism	18 (<0.1%)
Shinto	12 (<0.1%)
Taoism	93 (0.2%)
Confucianism	8 (<0.1%)
Primal, Animist, or Folk religion	240 (0.6%)
Spiritism	0 (0%)
Umbanda, Candomble, and other African-derived religions	0 (0%)
Chinese folk/traditional religion	0 (0%)
Some other religion	1,267 (3.3%)
No religion/Atheist/Agnostic	11,870 (31%)
(Missing)	467 (1.2%)
**Race/Ethnicity**	
Asian	2,466 (6.4%)
Black	4,501 (12%)
Hispanic	6,724 (18%)
Other	997 (2.6%)
White	23,605 (62%)
(Missing)	20 (<0.1%)

[1]n (%)

Table S22b. Proportions by demographic category for United States

Variable	Category	Mean	95% CI	SE	Global p-value
Age group	18-24	0.56	(0.48, 0.64)	0.04	< .001
	25-29	0.60	(0.55, 0.65)	0.03	
	30-39	0.65	(0.62, 0.68)	0.02	
	40-49	0.70	(0.67, 0.72)	0.01	
	50-59	0.80	(0.78, 0.81)	0.01	
	60-69	0.82	(0.81, 0.83)	0.01	
	70-79	0.80	(0.79, 0.82)	0.01	
	80 or older	0.83	(0.79, 0.87)	0.02	
Gender	Male	0.68	(0.66, 0.70)	0.01	< .001
	Female	0.78	(0.76, 0.79)	0.01	
	Other	0.42	(0.26, 0.59)	0.08	
Marital status	Married	0.76	(0.75, 0.77)	0.01	< .001
	Separated	0.80	(0.72, 0.88)	0.04	
	Divorced	0.77	(0.75, 0.79)	0.01	
	Widowed	0.87	(0.84, 0.90)	0.01	
	Never	0.63	(0.60, 0.66)	0.02	
	Domestic Partner	0.60	(0.54, 0.66)	0.03	
Employment	Employed for an employer	0.69	(0.68, 0.71)	0.01	< .001
	Self-employed	0.75	(0.71, 0.79)	0.02	
	Retired	0.81	(0.80, 0.82)	0.01	
	Student	0.51	(0.41, 0.62)	0.05	
	Homemaker	0.78	(0.74, 0.82)	0.02	
	Unemployed and looking for a job	0.65	(0.56, 0.74)	0.05	
	None of these/Other	0.75	(0.68, 0.82)	0.04	
Religious service attendance	At least 1/week	0.99	(0.98, 1.00)	0.00	< .001
	1/week	0.98	(0.97, 0.99)	0.00	
	1-3/month	0.92	(0.87, 0.96)	0.02	
	A few times a year	0.84	(0.82, 0.86)	0.01	
	Never	0.52	(0.50, 0.53)	0.01	
Education	Up to 8 years	0.92	(0.77, 1.00)	0.07	< .001
	9-15 years	0.75	(0.73, 0.76)	0.01	
	16+ years	0.68	(0.67, 0.69)	0.00	
Immigration status	Born in this country	0.73	(0.72, 0.74)	0.01	0.033

Variable	Category	Mean	95% CI	SE	Global p-value
	Born in another country	0.68	(0.63, 0.72)	0.02	
Religious affiliation	Christianity	0.94	(0.93, 0.94)	0.00	< .001
	Islam	0.94	(0.84, 1.00)	0.05	
	Hinduism	0.89	(0.80, 0.97)	0.04	
	Buddhism	0.79	(0.72, 0.86)	0.03	
	Judaism	0.66	(0.61, 0.72)	0.03	
	Sikhism	0.99	(0.97, 1.00)	0.01	
	Baha'i	1.00	(1.00, 1.00)	0.00	
	Jainism	0.87	(0.44, 1.00)	0.11	
	Shinto	0.93	(0.78, 1.00)	0.07	
	Taoism	0.32	(0.04, 0.60)	0.14	
	Confucianism	1.00	*	*	
	Primal, Animist, or Folk religion	0.92	(0.85, 0.99)	0.04	
	Some other religion	0.77	(0.69, 0.84)	0.04	
	No religion/Atheist/Agnostic	0.31	(0.28, 0.33)	0.01	
Race/ethnicity	Asian	0.61	(0.56, 0.65)	0.02	< .001
	Black	0.84	(0.80, 0.87)	0.02	
	White	0.72	(0.71, 0.73)	0.01	
	Other	0.82	(0.78, 0.86)	0.02	
	Hispanic	0.71	(0.67, 0.75)	0.02	

Population Weighted Meta-Analysis

Table S23. Population weighted meta-analysis of results demographic group means.

Variable	Category	Proportion	95% CI of Proportion	SE Analogue (CI Width/4)
Age group				
	18-24	0.90	(0.89,0.92)	0.01
	25-29	0.93	(0.91,0.94)	0.01
	30-39	0.93	(0.91,0.94)	0.01
	40-49	0.93	(0.92,0.94)	0.01
	50-59	0.95	(0.94,0.96)	0.00
	60-69	0.95	(0.94,0.96)	0.01
	70-79	0.94	(0.92,0.95)	0.01
	80 or older	1.00	(1.00,1.00)	0.00
Gender				
	Male	0.91	(0.90,0.93)	0.01
	Female	0.94	(0.93,0.95)	0.01
	Other	0.87	(0.84,0.89)	0.01
Marital status				
	Married	0.94	(0.93,0.95)	0.01
	Separated	0.90	(0.88,0.91)	0.01
	Divorced	0.99	(0.99,1.00)	0.00
	Widowed	0.95	(0.94,0.96)	0.00
	Domestic partner	0.94	(0.92,0.95)	0.01
	Single, never married	0.90	(0.88,0.91)	0.01
Employment status				
	Employed for an employer	0.92	(0.91,0.93)	0.01
	Self-employed	0.93	(0.91,0.94)	0.01
	Retired	0.93	(0.92,0.94)	0.01
	Student	0.91	(0.89,0.92)	0.01
	Homemaker	0.95	(0.94,0.96)	0.00
	Unemployed and looking for a job	0.93	(0.91,0.94)	0.01
	None of these/other	0.92	(0.91,0.93)	0.01
Education				
	Up to 8 years	0.94	(0.93,0.95)	0.01
	9-15 years	0.92	(0.90,0.93)	0.01
	16+ years	0.93	(0.91,0.94)	0.01
Religious service attendance				
	>1/week	0.98	(0.98,0.99)	0.00
	1/week	0.97	(0.97,0.98)	0.00
	1-3/month	0.96	(0.95,0.97)	0.00
	A few times a year	0.94	(0.92,0.95)	0.01
	Never	0.80	(0.77,0.82)	0.01

Variable	Category	Proportion	95% CI of Proportion	SE Analogue (CI Width/4)
Immigration status				
	Born in this country	0.93	(0.92,0.94)	0.01
	Born in another country	0.97	(0.96,0.98)	0.00

1. Forest Plots

Figure S1. Forest plot for `Age group`-`18-24`

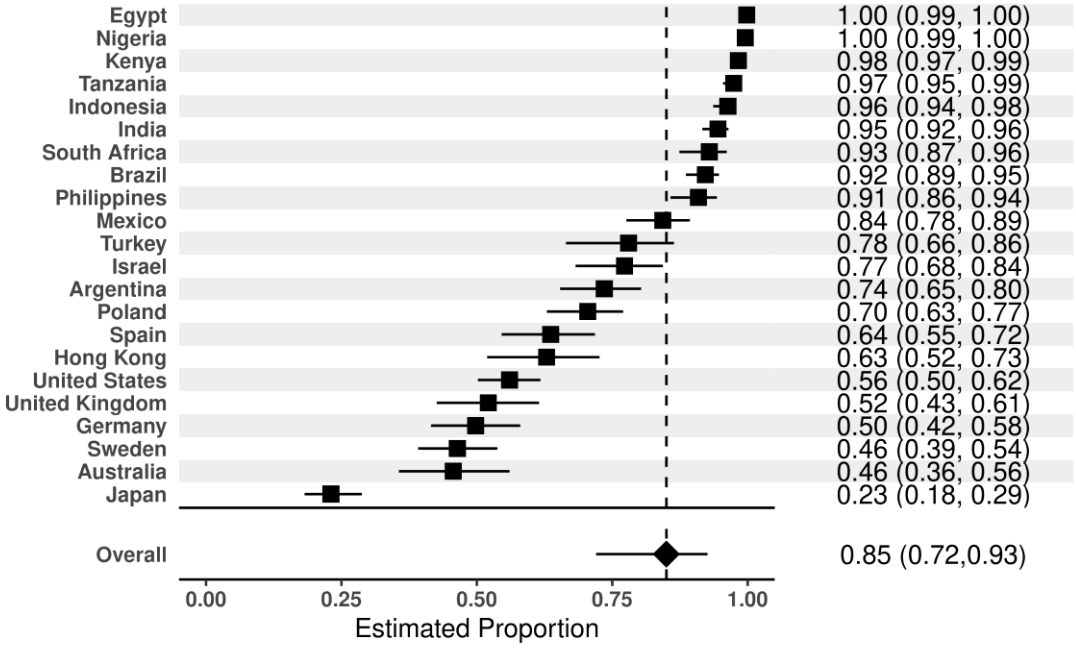

Probability-scale: τ=0.239;
Logit-scale: τ=1.874; Q(df=21)=1293.37, p<.001;Q-profile 95% CI [1.360, 2.539]; I^2=99.09;
Plot is based on back transformed bounds after using approximate logit SE that aren't guarenteed to match the robust SE of a proportion.

Figure S2. Forest plot for `Age group`-`25-29`

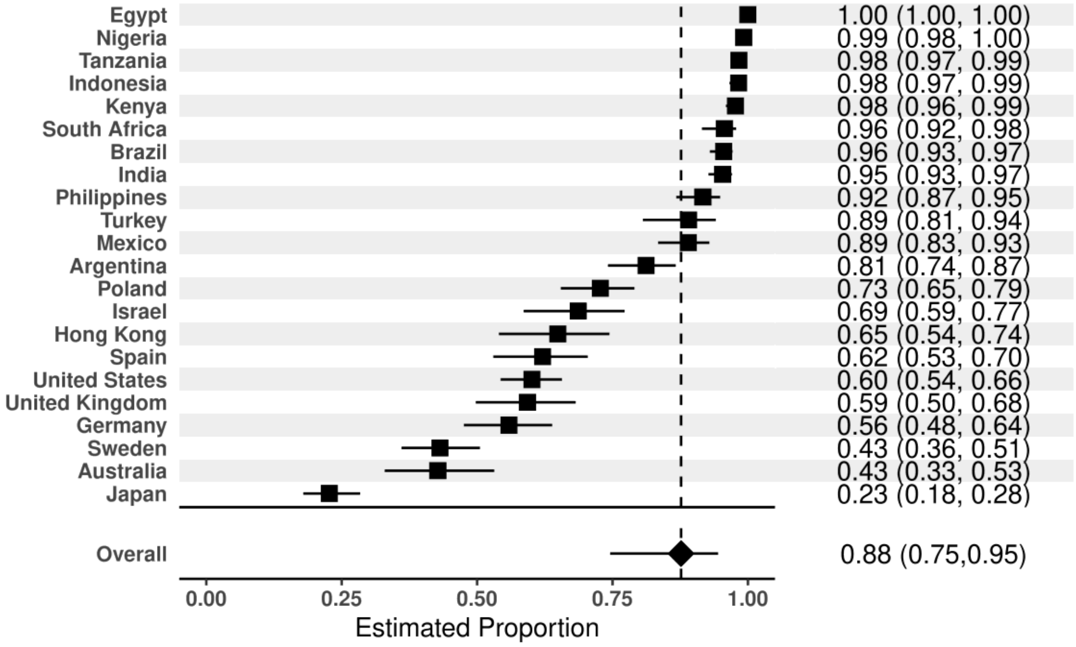

Probability-scale: τ=0.227;
Logit-scale: τ=2.098; Q(df=21)=1366.62, p<.001;Q-profile 95% CI [1.459, 2.815]; I^2=99.23;
Plot is based on back transformed bounds after using approximate logit SE that aren't guarenteed to match the robust SE of a proportion.

Figure S3. Forest plot for `Age group`-`30-39`

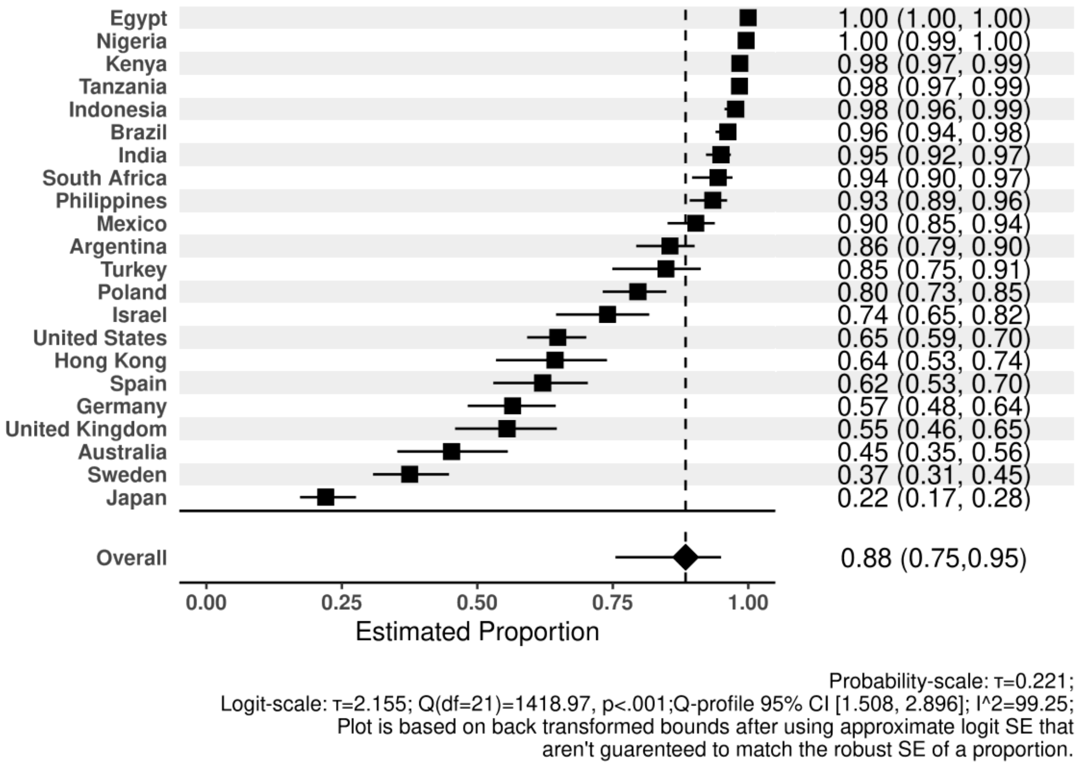

Figure S4. Forest plot for `Age group`-`40-49`

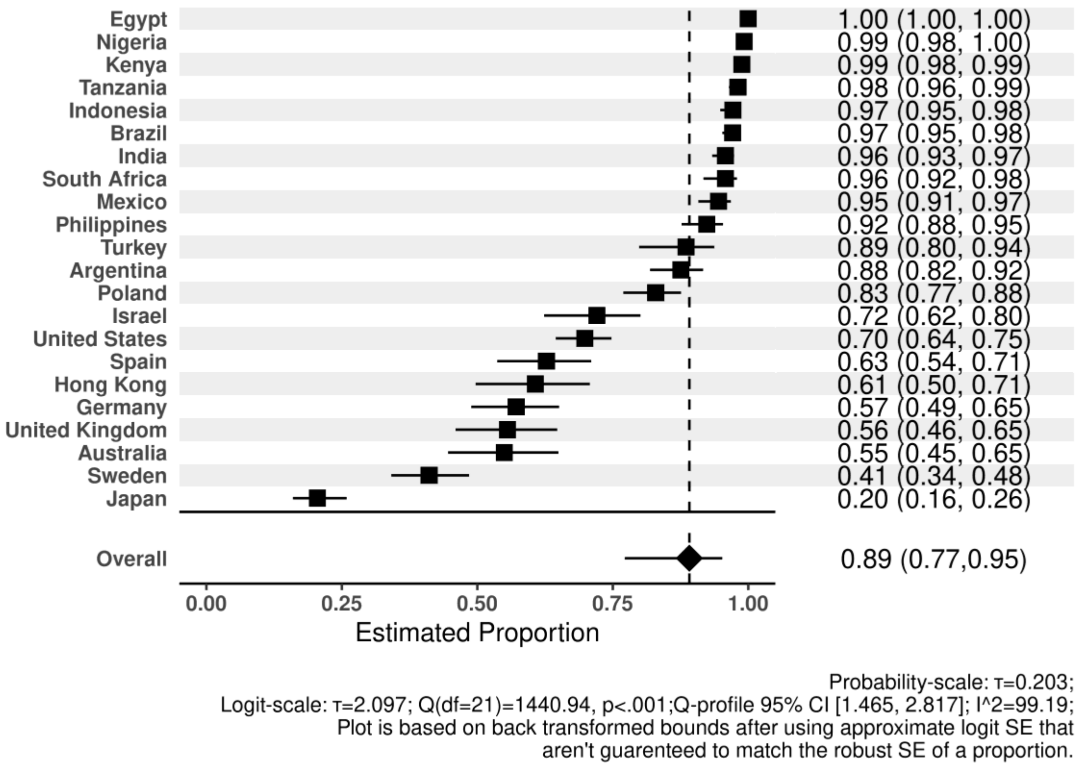

Figure S5. Forest plot for `Age group`-`50-59`

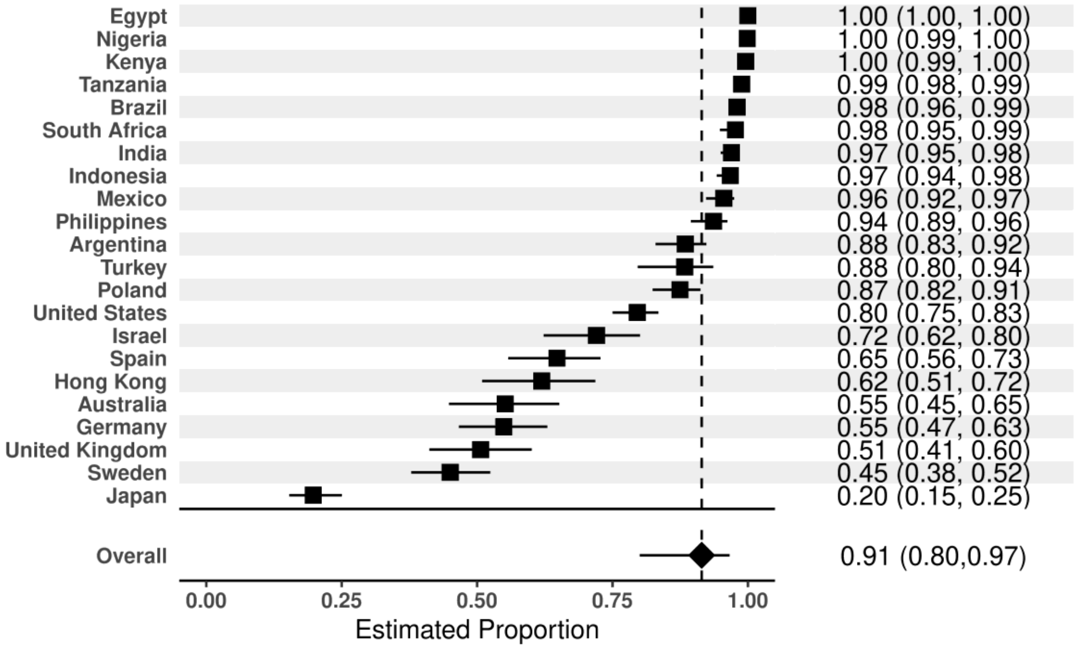

Probability-scale: τ=0.182;
Logit-scale: τ=2.335; Q(df=21)=1512.23, p<.001;Q-profile 95% CI [1.661, 3.151]; I^2=99.30;
Plot is based on back transformed bounds after using approximate logit SE that aren't guarenteed to match the robust SE of a proportion.

Figure S6. Forest plot for `Age group`-`60-69`

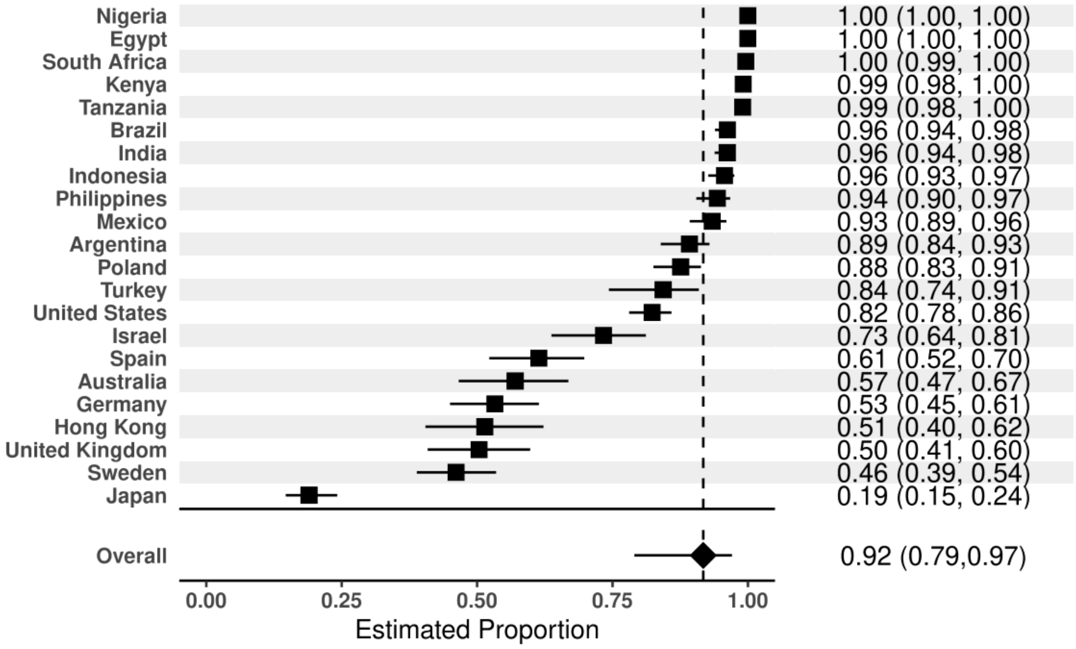

Probability-scale: τ=0.194;
Logit-scale: τ=2.567; Q(df=21)=1471.83, p<.001;Q-profile 95% CI [1.795, 3.455]; I^2=99.43;
Plot is based on back transformed bounds after using approximate logit SE that aren't guarenteed to match the robust SE of a proportion.

Figure S7. Forest plot for `Age group`-`70-79`

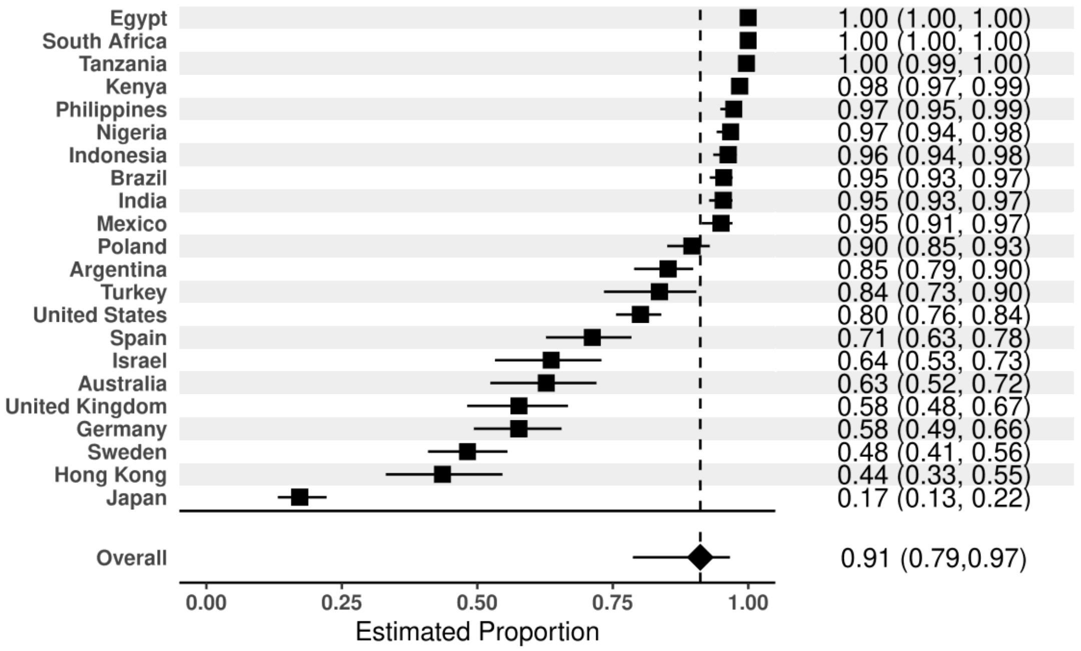

Probability-scale: τ=0.196;
Logit-scale: τ=2.426; Q(df=21)=1446.29, p<.001;Q-profile 95% CI [1.690, 3.262]; I^2=99.37;
Plot is based on back transformed bounds after using approximate logit SE that
aren't guarenteed to match the robust SE of a proportion.

Figure S8. Forest plot for `Age group`-`80 or older`

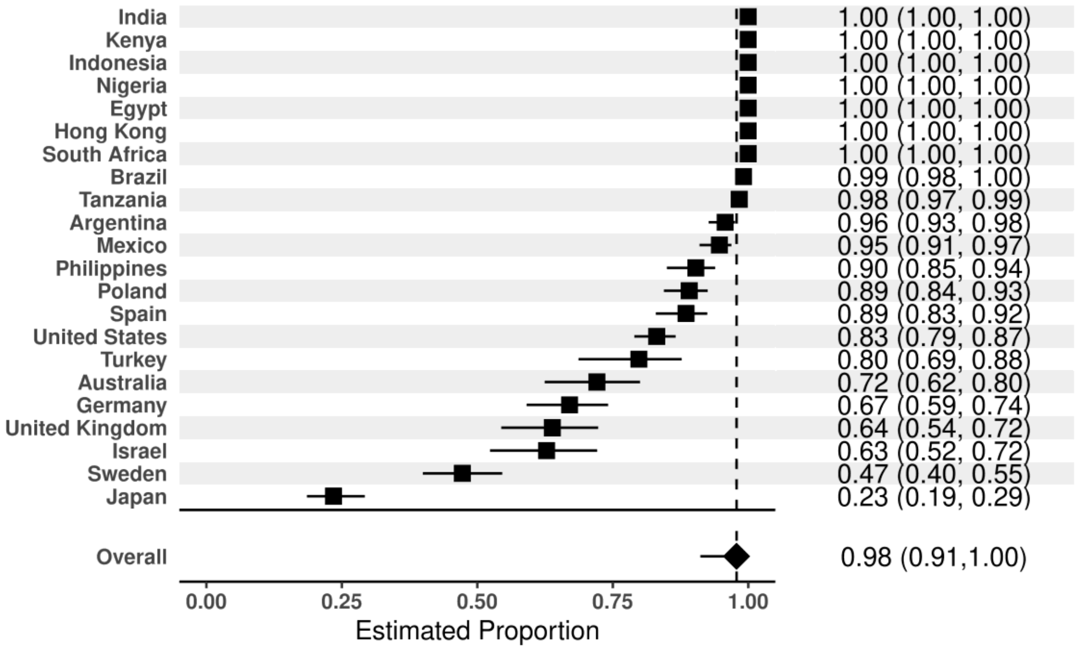

Probability-scale: τ=0.074;
Logit-scale: τ=3.498; Q(df=21)=1306.45, p<.001;Q-profile 95% CI [2.524, 4.753]; I^2=99.62;
Plot is based on back transformed bounds after using approximate logit SE that
aren't guarenteed to match the robust SE of a proportion.

Figure S9. Forest plot for `Gender`-`Male`

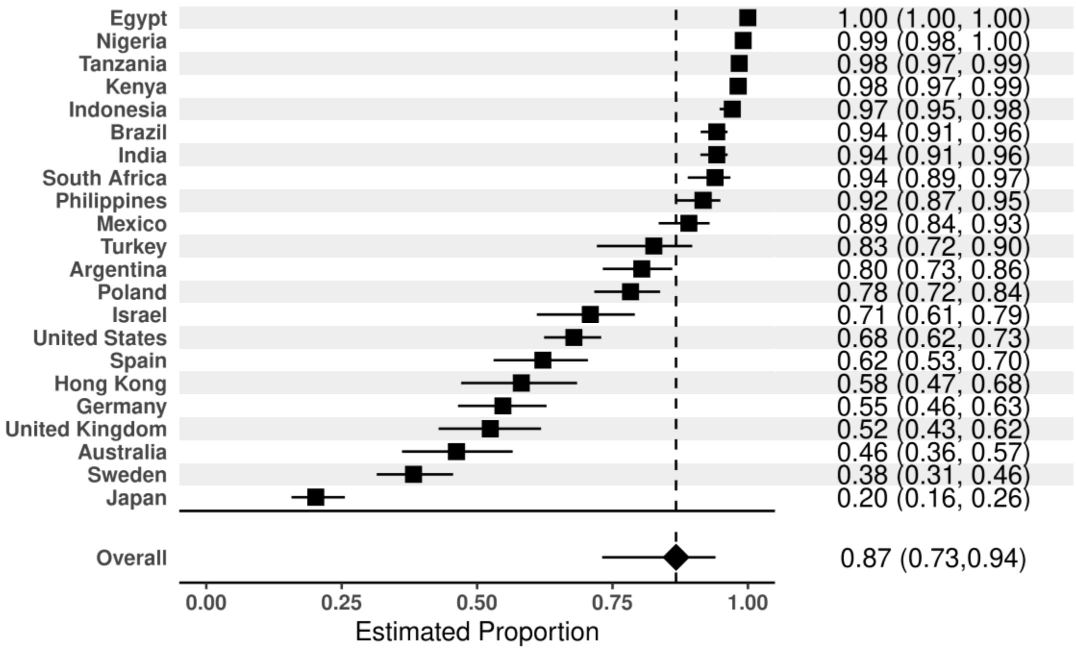

Probability-scale: τ=0.240;
Logit-scale: τ=2.083; Q(df=21)=1368.09, p<.001;Q-profile 95% CI [1.442, 2.793]; I^2=99.23;
Plot is based on back transformed bounds after using approximate logit SE that
aren't guarenteed to match the robust SE of a proportion.

Figure S10. Forest plot for `Gender`-`Female`

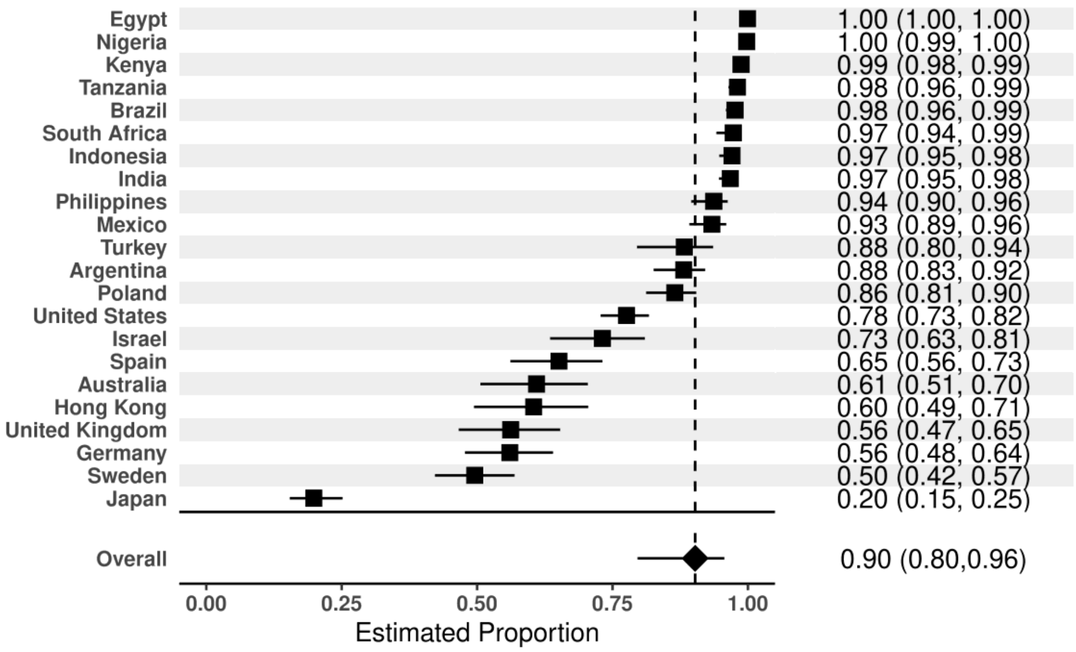

Probability-scale: τ=0.180;
Logit-scale: τ=2.048; Q(df=21)=1427.20, p<.001;Q-profile 95% CI [1.480, 2.774]; I^2=99.12;
Plot is based on back transformed bounds after using approximate logit SE that
aren't guarenteed to match the robust SE of a proportion.

Figure S11. Forest plot for `Gender`-`Other`

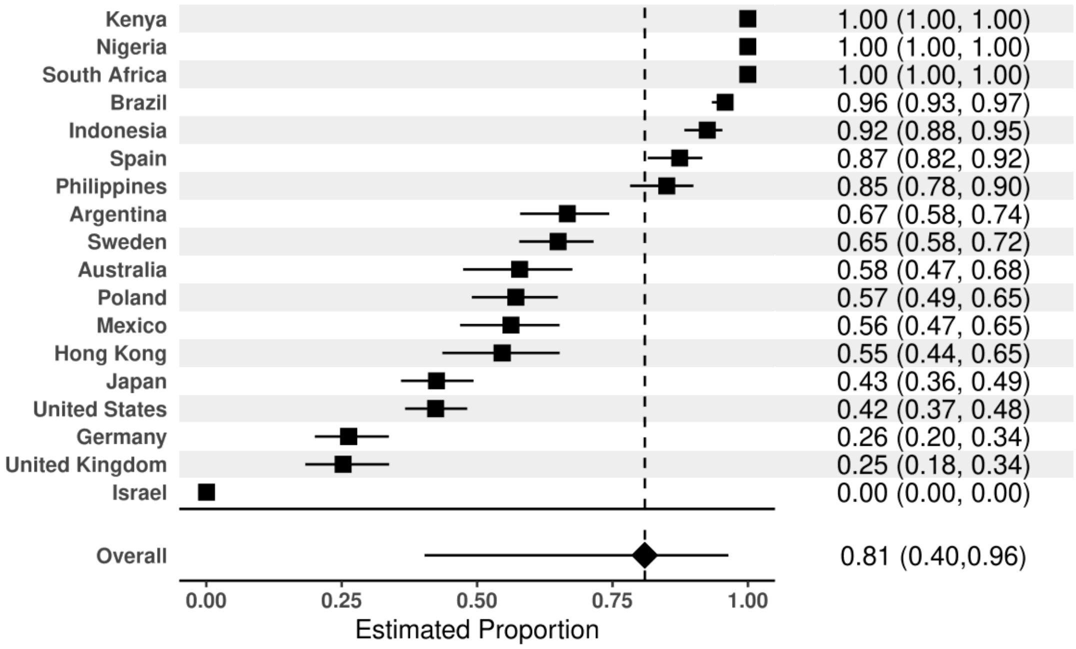

Probability-scale: τ=0.610;
Logit-scale: τ=3.957; Q(df=17)=908.70, p<.001;Q-profile 95% CI [2.710, 5.525]; I^2=99.80;
Plot is based on back transformed bounds after using approximate logit SE that aren't guarenteed to match the robust SE of a proportion.
Excluded countries: India, Egypt, Tanzania, Turkiye

Figure S12. Forest plot for `Marital status`-`Married`

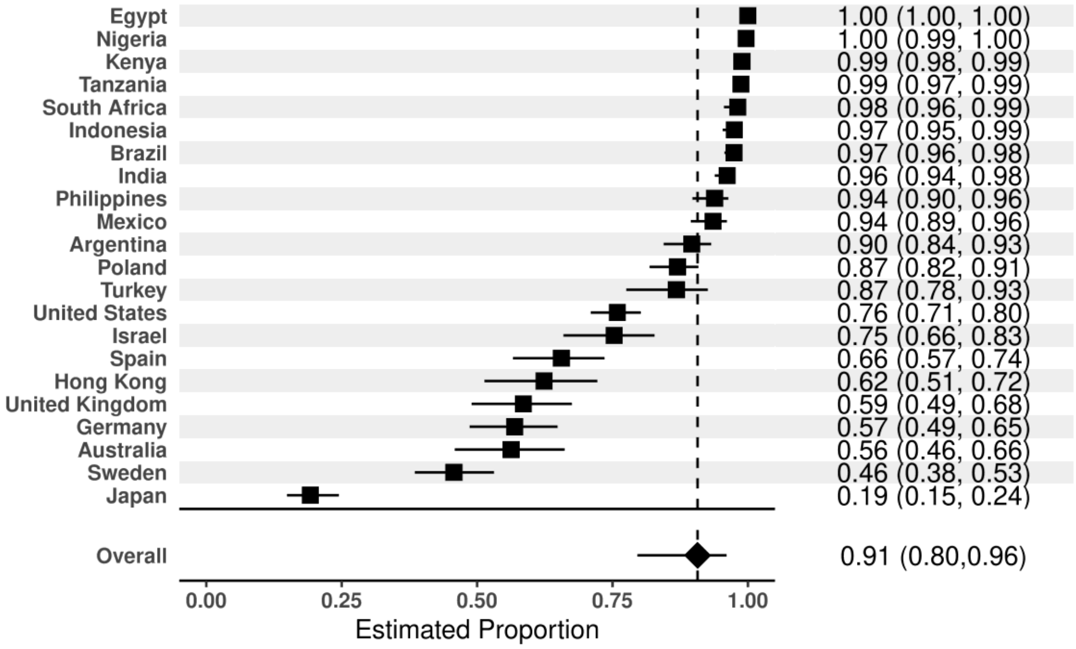

Probability-scale: τ=0.183;
Logit-scale: τ=2.173; Q(df=21)=1457.68, p<.001;Q-profile 95% CI [1.532, 2.926]; I^2=99.21;
Plot is based on back transformed bounds after using approximate logit SE that aren't guarenteed to match the robust SE of a proportion.

Figure S13. Forest plot for `Marital status`-`Separated`

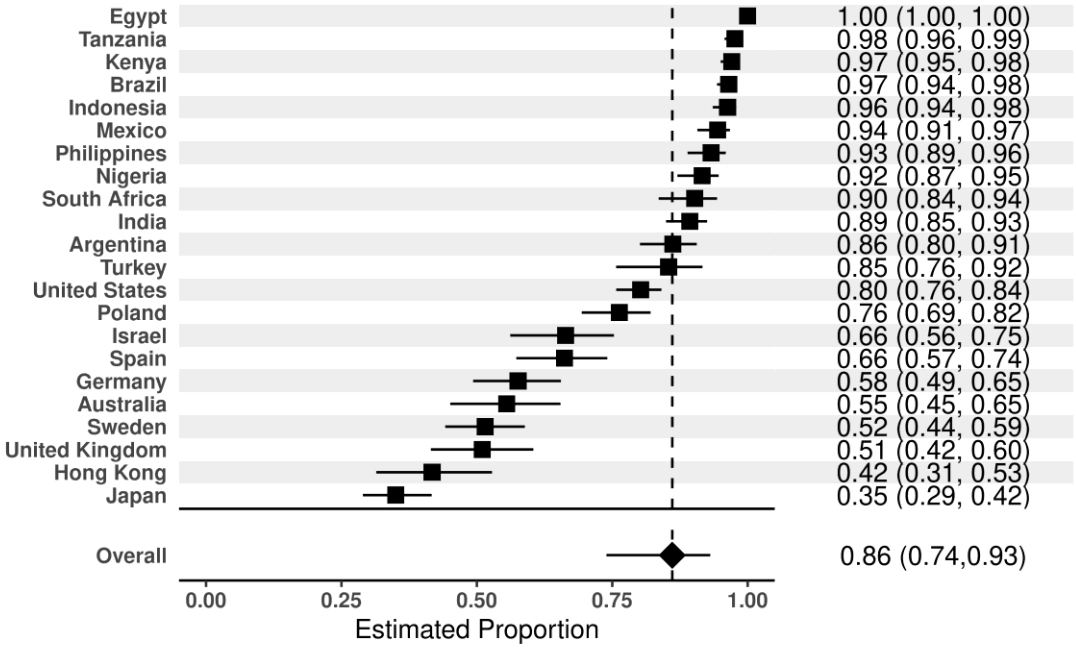

Probability-scale: τ=0.221;
Logit-scale: τ=1.846; Q(df=21)=1081.60, p<.001;Q-profile 95% CI [1.221, 2.454]; I^2=99.04;
Plot is based on back transformed bounds after using approximate logit SE that aren't guarenteed to match the robust SE of a proportion.

Figure S14. Forest plot for `Marital status`-`Divorced`

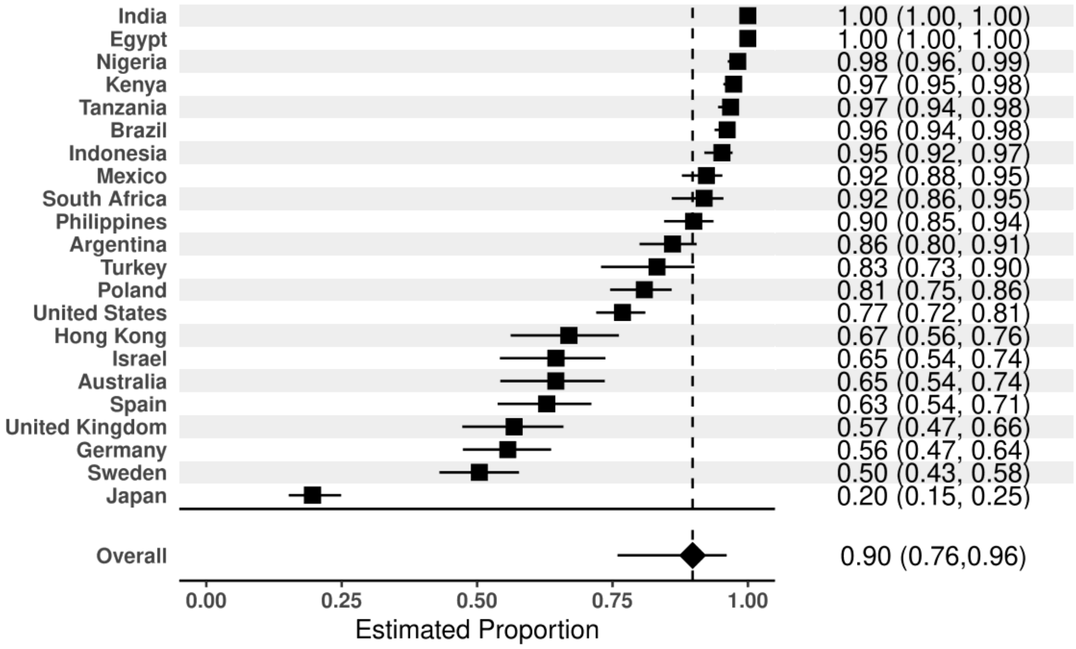

Probability-scale: τ=0.223;
Logit-scale: τ=2.432; Q(df=21)=1212.33, p<.001;Q-profile 95% CI [1.639, 3.250]; I^2=99.40;
Plot is based on back transformed bounds after using approximate logit SE that aren't guarenteed to match the robust SE of a proportion.

Figure S15. Forest plot for `Marital status`-`Widowed`

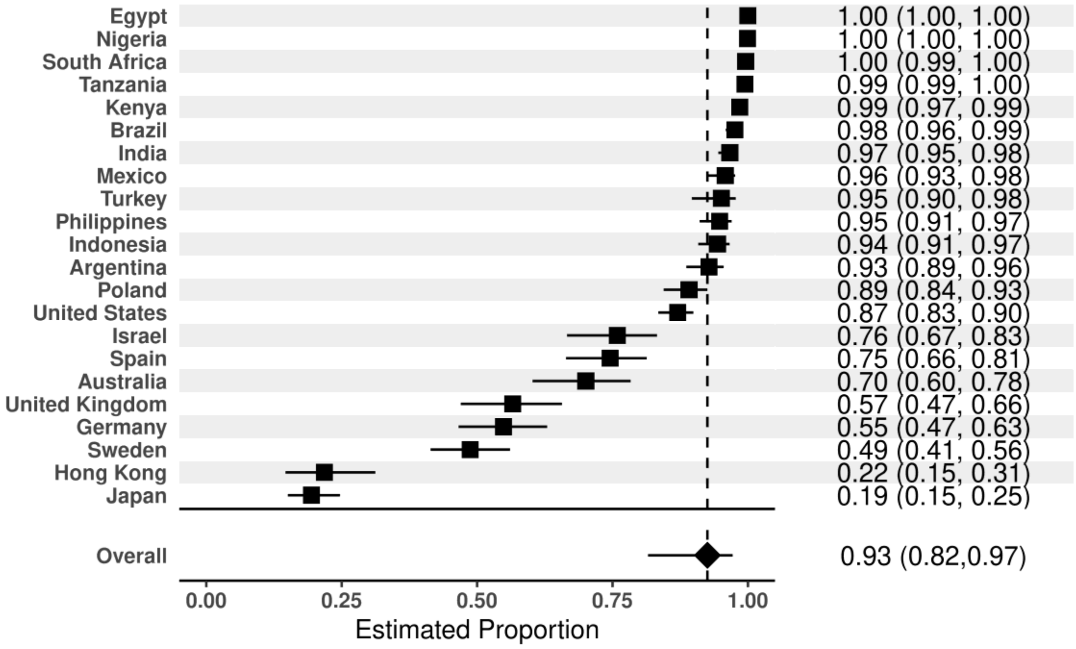

Probability-scale: τ=0.169;
Logit-scale: τ=2.439; Q(df=21)=1616.83, p<.001;Q-profile 95% CI [1.743, 3.295]; I^2=99.32;
Plot is based on back transformed bounds after using approximate logit SE that
aren't guarenteed to match the robust SE of a proportion.

Figure S16. Forest plot for `Marital status`-`Single, never married`

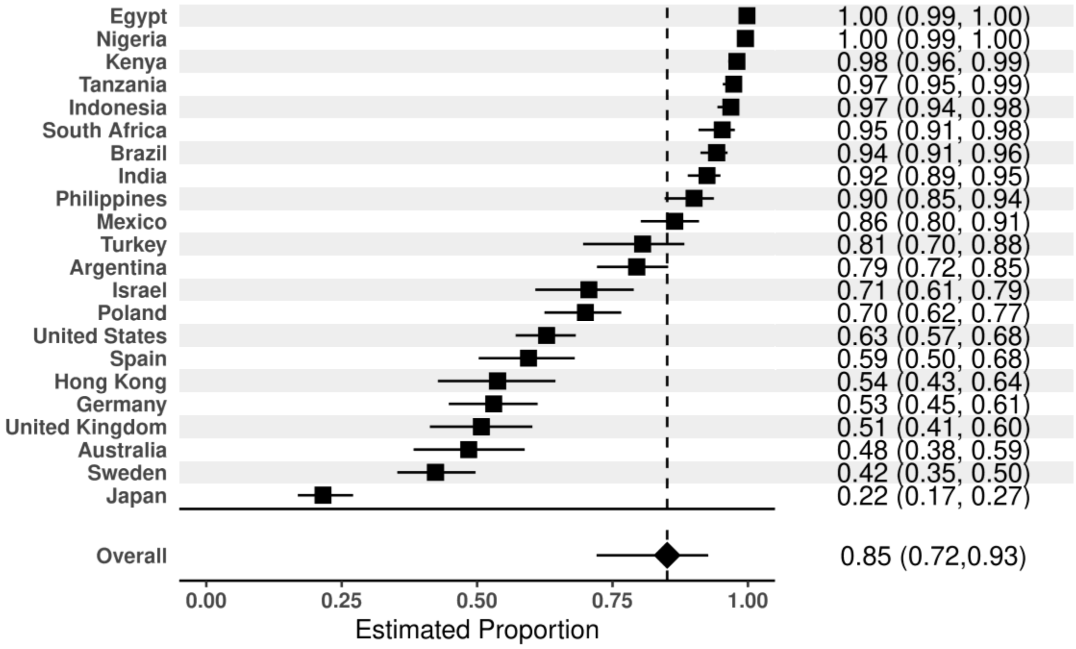

Probability-scale: τ=0.239;
Logit-scale: τ=1.888; Q(df=21)=1326.13, p<.001;Q-profile 95% CI [1.372, 2.559]; I^2=99.09;
Plot is based on back transformed bounds after using approximate logit SE that
aren't guarenteed to match the robust SE of a proportion.

Figure S17. Forest plot for `Marital status`-`Domestic partner`

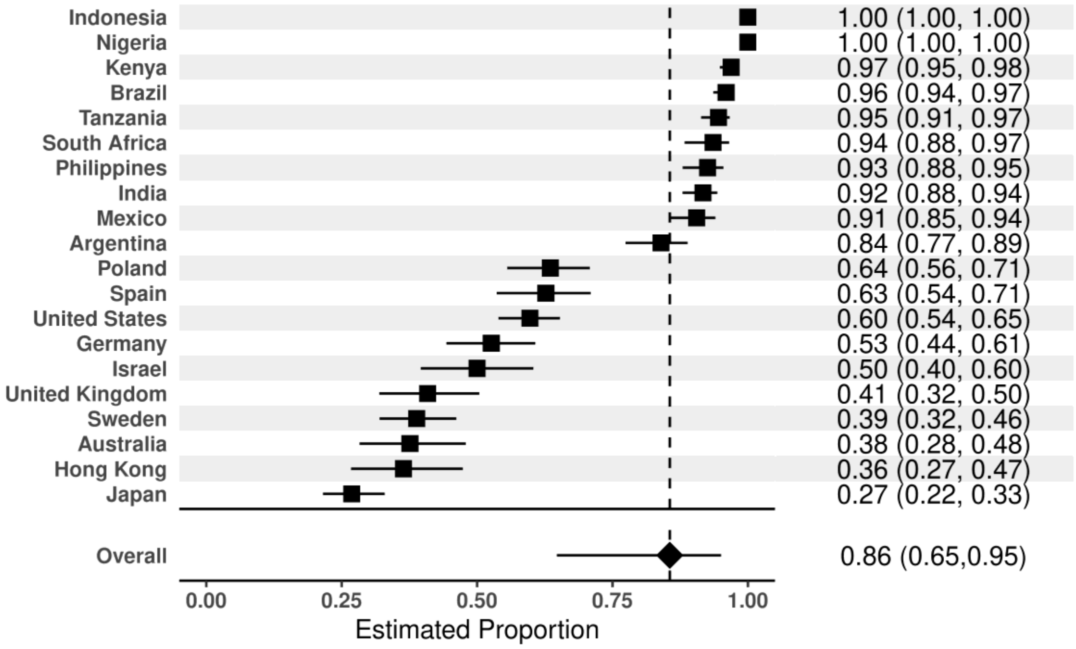

Probability-scale: τ=0.328;
Logit-scale: τ=2.656; Q(df=19)=1226.52, p<.001;Q-profile 95% CI [1.786, 3.615]; I^2=99.56;
Plot is based on back transformed bounds after using approximate logit SE that aren't guarenteed to match the robust SE of a proportion.
Excluded countries: Egypt, Turkiye

Figure S18. Forest plot for `Employment status`-`Employed for an employer`

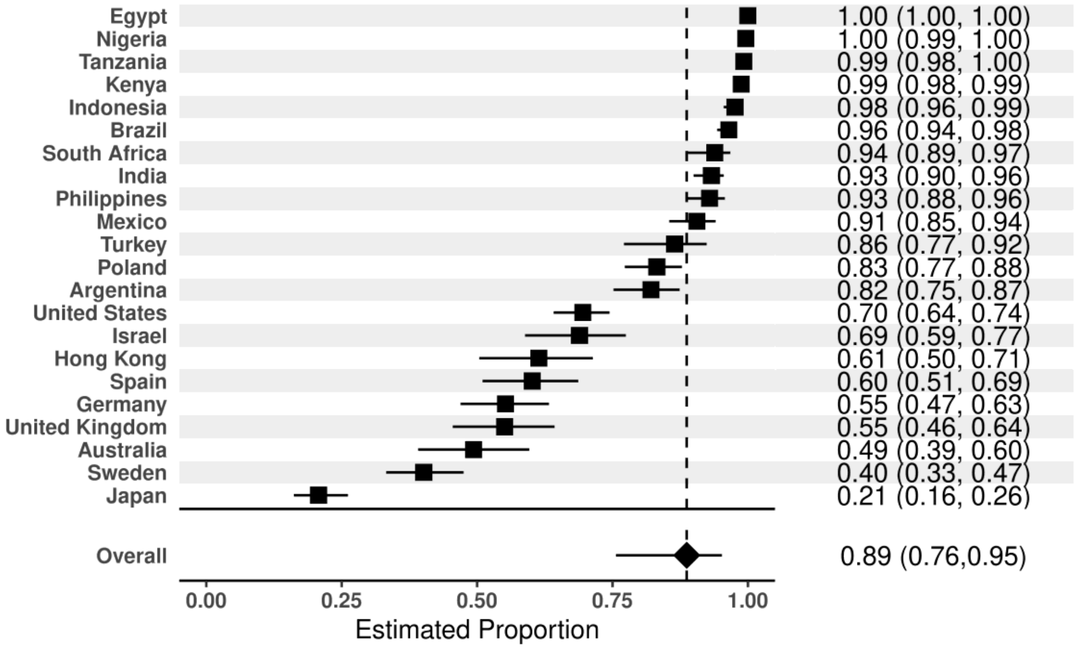

Probability-scale: τ=0.221;
Logit-scale: τ=2.201; Q(df=21)=1400.55, p<.001;Q-profile 95% CI [1.546, 2.960]; I^2=99.28;
Plot is based on back transformed bounds after using approximate logit SE that aren't guarenteed to match the robust SE of a proportion.

Figure S19. Forest plot for `Employment status`-`Self-employed`

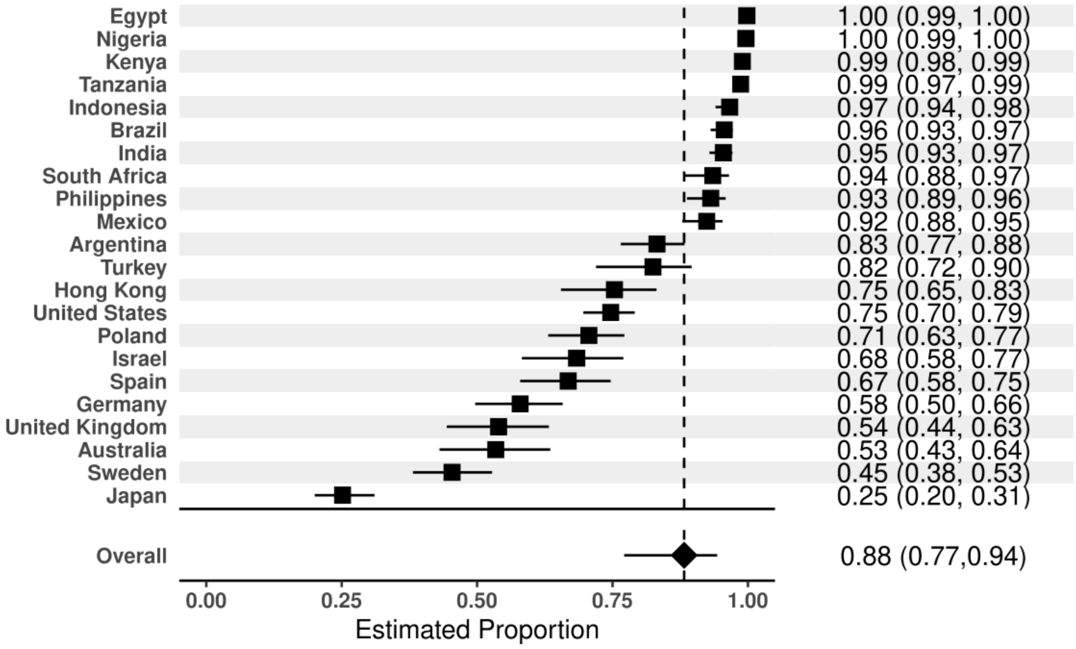

Probability-scale: τ=0.197;
Logit-scale: τ=1.891; Q(df=21)=1304.98, p<.001;Q-profile 95% CI [1.378, 2.566]; I^2=99.04;
Plot is based on back transformed bounds after using approximate logit SE that
aren't guarenteed to match the robust SE of a proportion.

Figure S20. Forest plot for `Employment status`-`Retired`

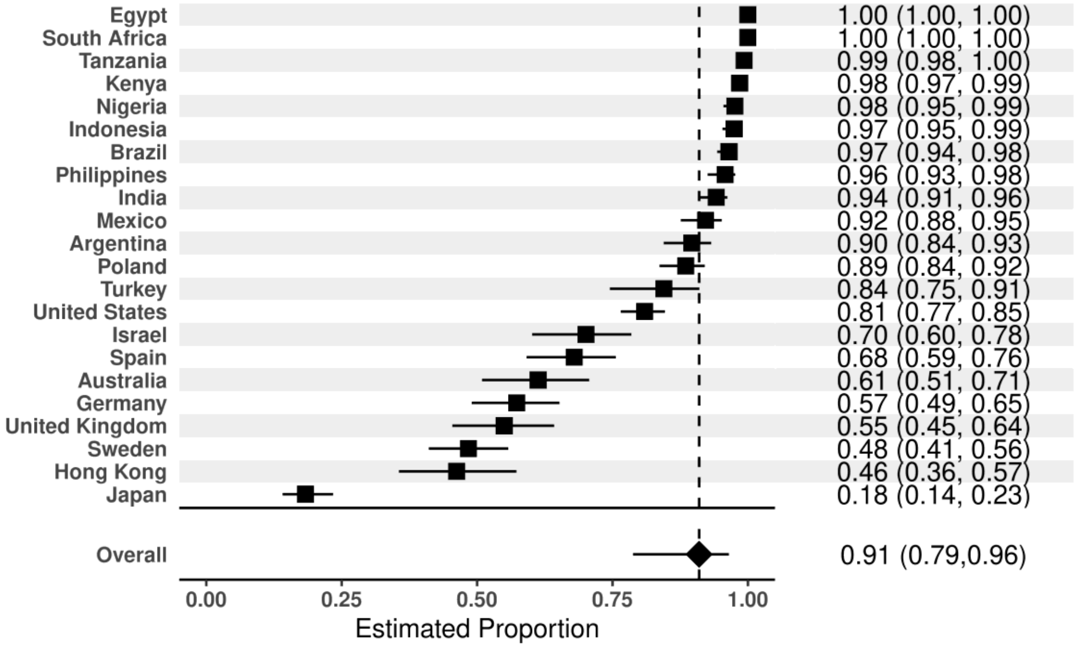

Probability-scale: τ=0.194;
Logit-scale: τ=2.372; Q(df=21)=1437.52, p<.001;Q-profile 95% CI [1.642, 3.185]; I^2=99.34;
Plot is based on back transformed bounds after using approximate logit SE that
aren't guarenteed to match the robust SE of a proportion.

Figure S21. Forest plot for `Employment status`-`Student`

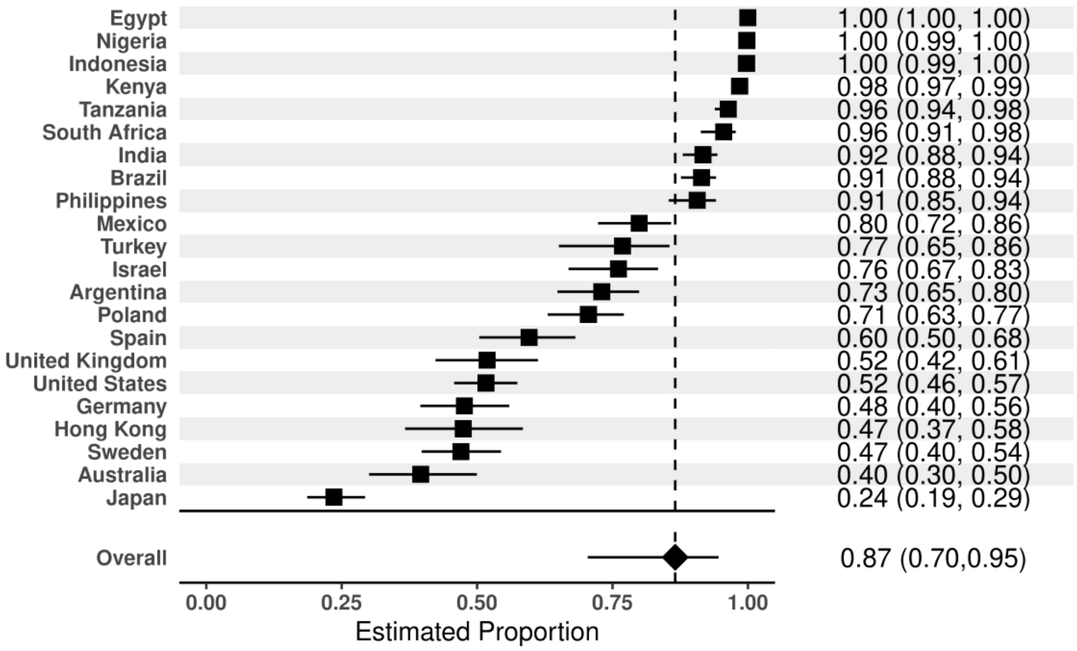

Probability-scale: τ=0.275;
Logit-scale: τ=2.365; Q(df=21)=1285.23, p<.001;Q-profile 95% CI [1.667, 3.183]; I^2=99.42;
Plot is based on back transformed bounds after using approximate logit SE that aren't guarenteed to match the robust SE of a proportion.

Figure S22. Forest plot for `Employment status`-`Homemaker`

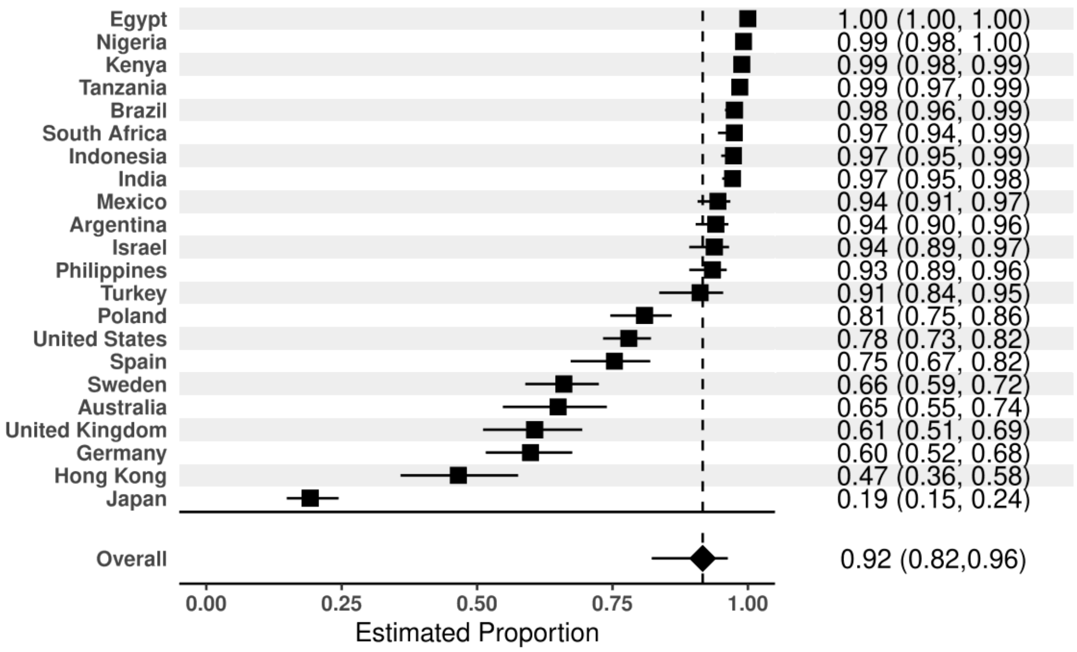

Probability-scale: τ=0.156;
Logit-scale: τ=2.043; Q(df=21)=1402.68, p<.001;Q-profile 95% CI [1.431, 2.748]; I^2=99.08;
Plot is based on back transformed bounds after using approximate logit SE that aren't guarenteed to match the robust SE of a proportion.

Figure S23. Forest plot for `Employment status`-`Unemployed and looking for a job`

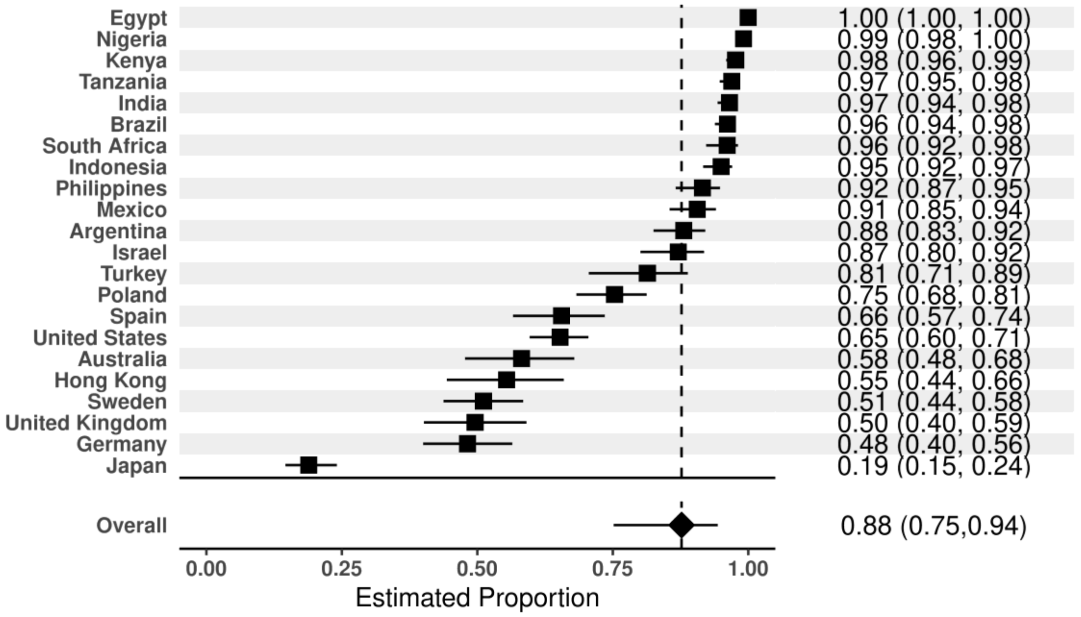

Probability-scale: τ=0.219;
Logit-scale: τ=2.028; Q(df=21)=1378.35, p<.001;Q-profile 95% CI [1.398, 2.717]; I^2=99.17;
Plot is based on back transformed bounds after using approximate logit SE that aren't guarenteed to match the robust SE of a proportion.

Figure S24. Forest plot for `Employment status`-`None of these/other`

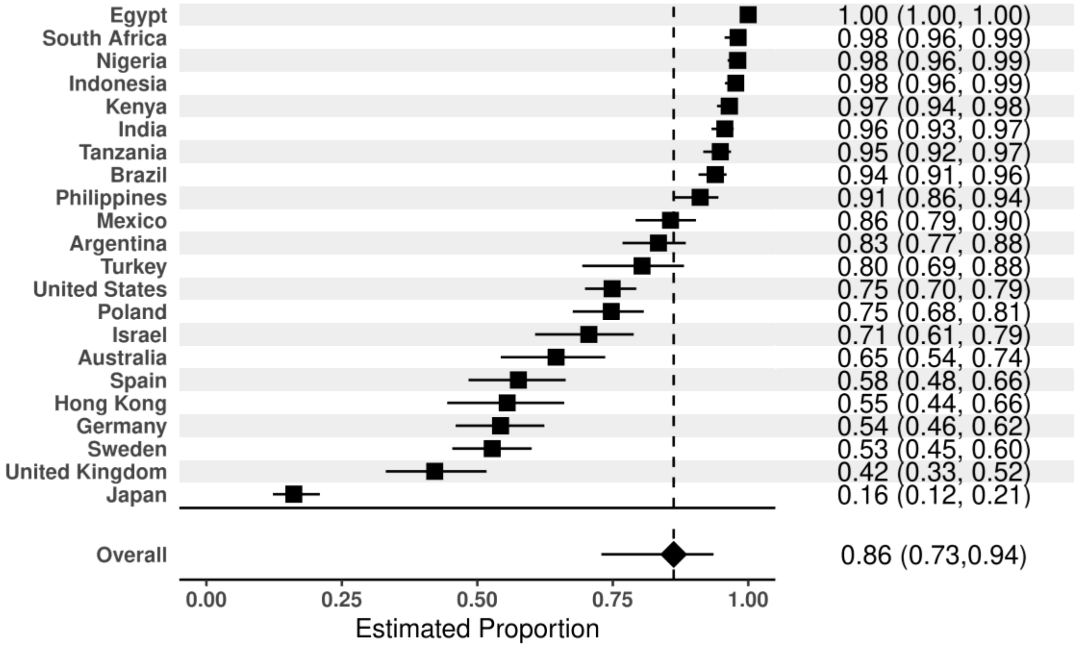

Probability-scale: τ=0.238;
Logit-scale: τ=2.005; Q(df=21)=1297.96, p<.001;Q-profile 95% CI [1.370, 2.681]; I^2=99.17;
Plot is based on back transformed bounds after using approximate logit SE that aren't guarenteed to match the robust SE of a proportion.

Figure S25. Forest plot for `Religious service attendance`-`>1/week`

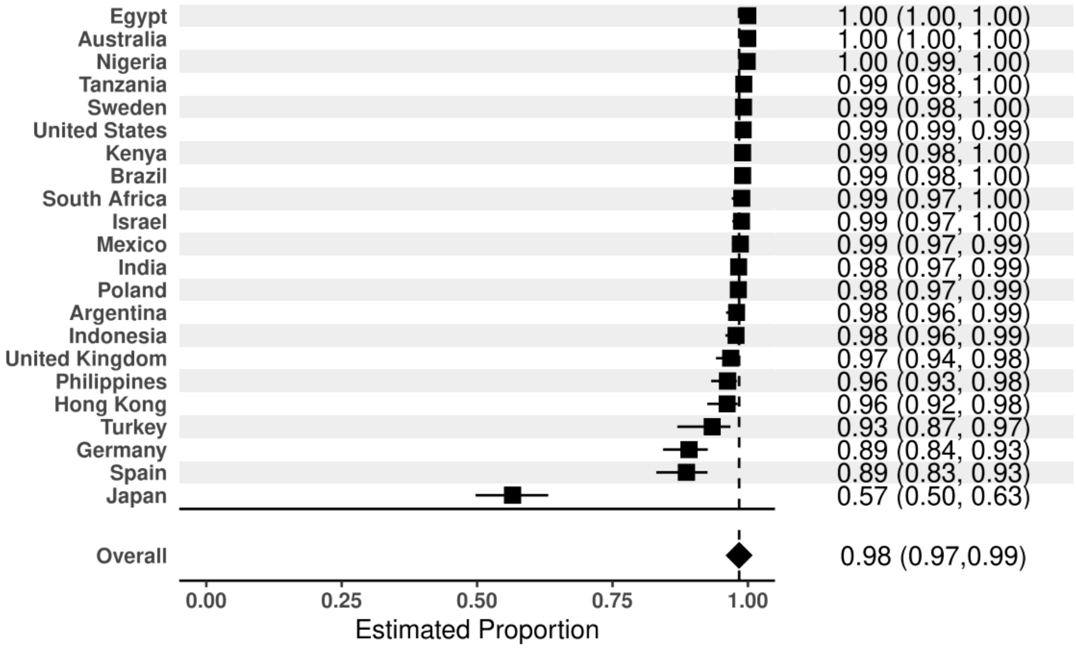

Probability-scale: τ=0.027;
Logit-scale: τ=1.706; Q(df=21)=937.94, p<.001;Q-profile 95% CI [1.137, 2.294]; I^2=97.51;
Plot is based on back transformed bounds after using approximate logit SE that aren't guarenteed to match the robust SE of a proportion.

Figure S26. Forest plot for `Religious service attendance`-`1/week`

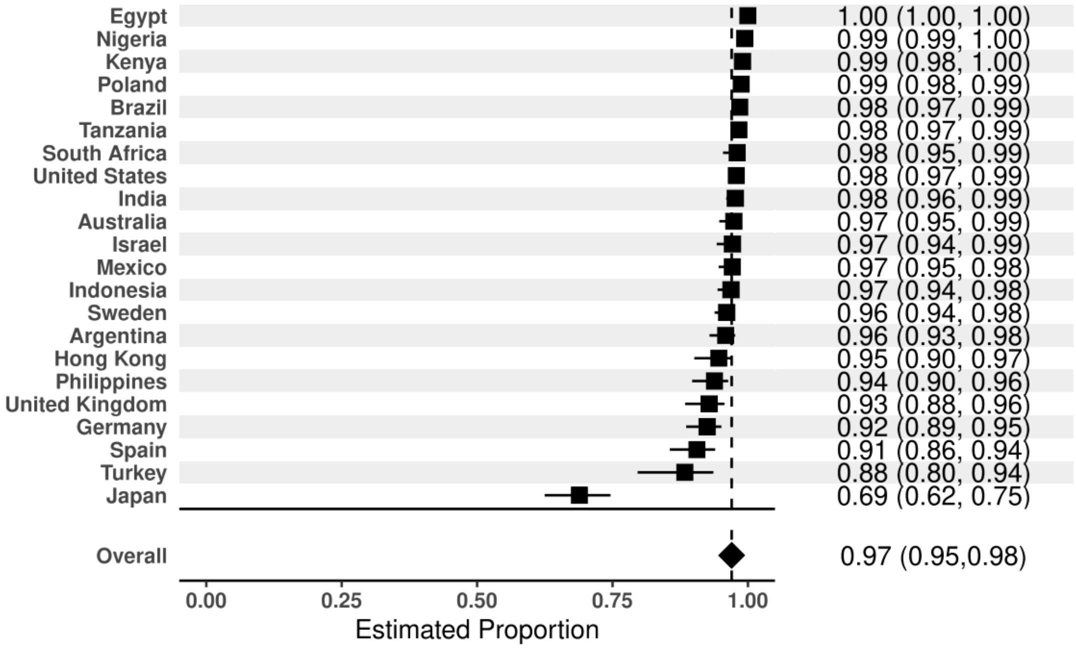

Probability-scale: τ=0.037;
Logit-scale: τ=1.282; Q(df=21)=541.84, p<.001;Q-profile 95% CI [0.785, 1.693]; I^2=96.43;
Plot is based on back transformed bounds after using approximate logit SE that aren't guarenteed to match the robust SE of a proportion.

Figure S27. Forest plot for `Religious service attendance`-`1-3/month`

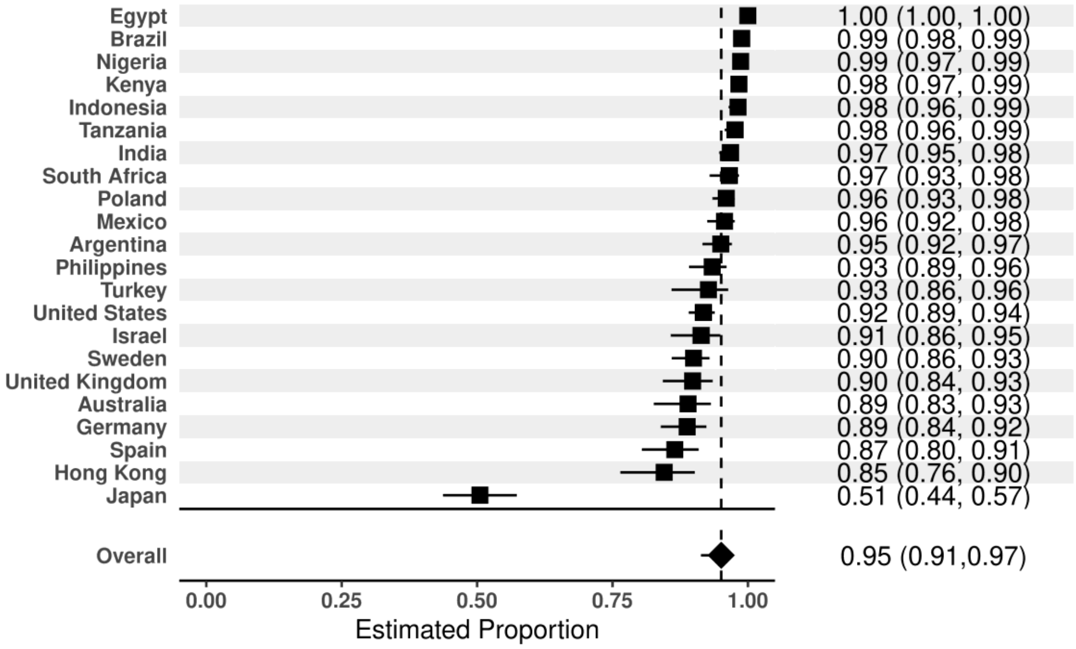

Probability-scale: τ=0.066;
Logit-scale: τ=1.417; Q(df=21)=695.43, p<.001;Q-profile 95% CI [0.876, 1.868]; I^2=97.67;
Plot is based on back transformed bounds after using approximate logit SE that aren't guarenteed to match the robust SE of a proportion.

Figure S28. Forest plot for `Religious service attendance`-`A few times a year`

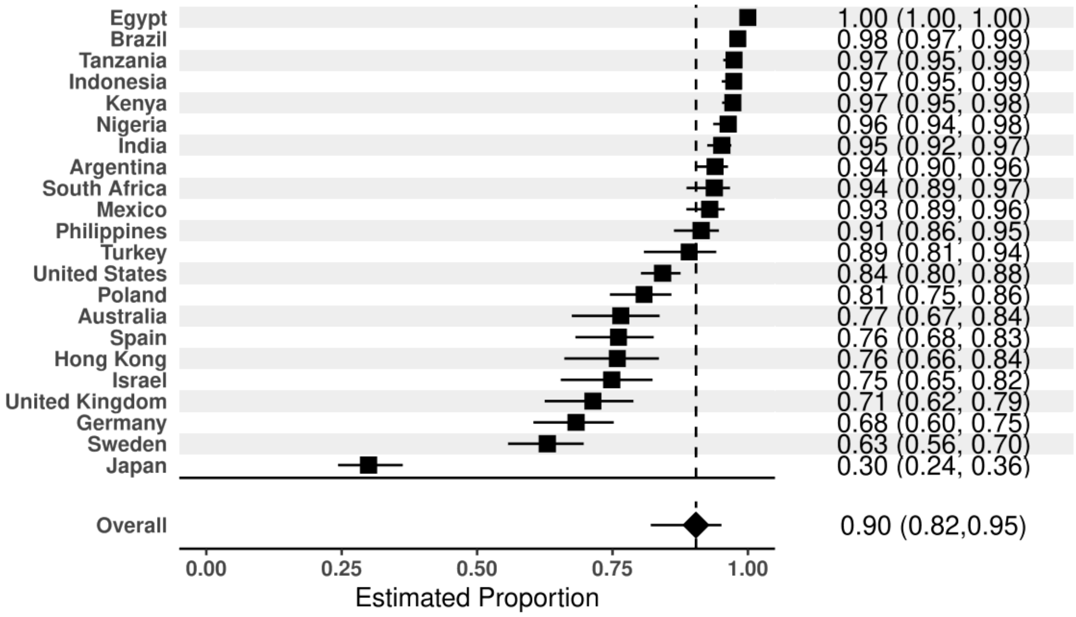

Probability-scale: τ=0.148;
Logit-scale: τ=1.709; Q(df=21)=1007.81, p<.001;Q-profile 95% CI [1.116, 2.268]; I^2=98.76;
Plot is based on back transformed bounds after using approximate logit SE that aren't guarenteed to match the robust SE of a proportion.

Figure S29. Forest plot for `Religious service attendance`-`Never`

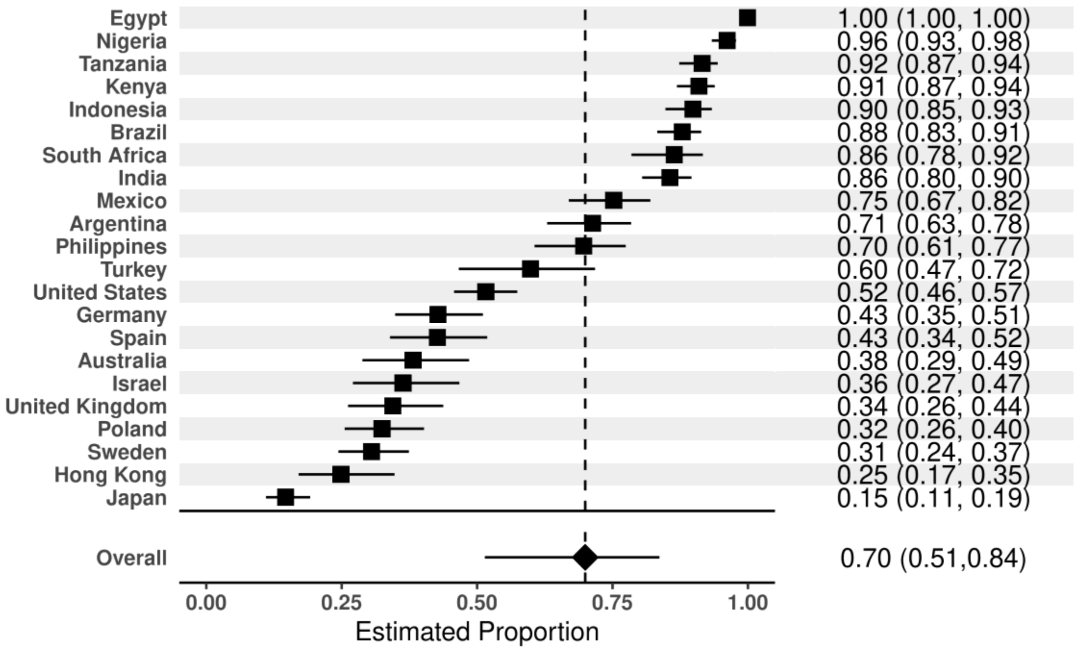

Probability-scale: τ=0.394;
Logit-scale: τ=1.873; Q(df=21)=1326.67, p<.001;Q-profile 95% CI [1.320, 2.520]; I^2=99.19;
Plot is based on back transformed bounds after using approximate logit SE that aren't guarenteed to match the robust SE of a proportion.

Figure S30. Forest plot for `Education`-`Up to 8 years`

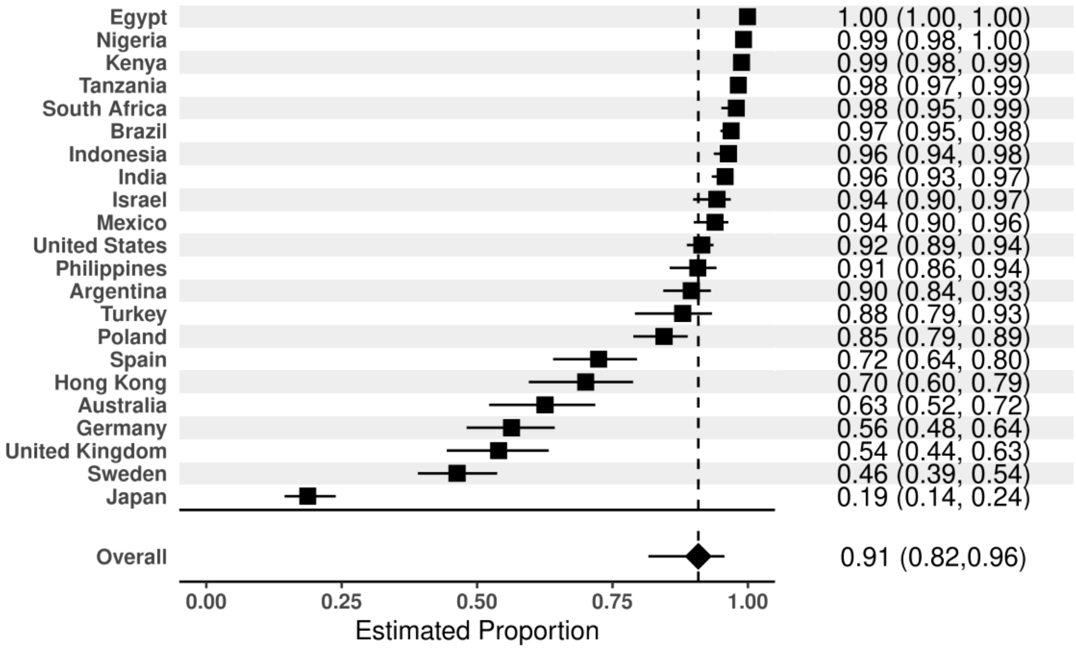

Probability-scale: τ=0.158;
Logit-scale: τ=1.905; Q(df=21)=1503.82, p<.001;Q-profile 95% CI [1.374, 2.580]; I^2=98.93;
Plot is based on back transformed bounds after using approximate logit SE that aren't guarenteed to match the robust SE of a proportion.

Figure S31. Forest plot for `Education`-`9-15 years`

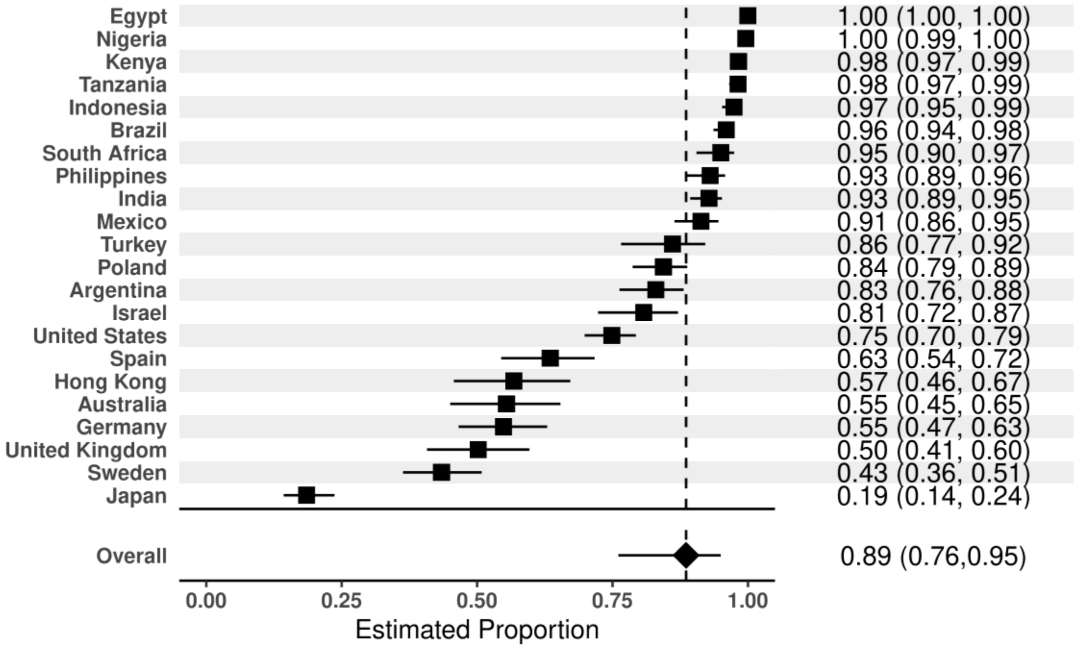

Probability-scale: τ=0.214;
Logit-scale: τ=2.114; Q(df=21)=1389.27, p<.001;Q-profile 95% CI [1.472, 2.839]; I^2=99.22;
Plot is based on back transformed bounds after using approximate logit SE that aren't guarenteed to match the robust SE of a proportion.

Figure S32. Forest plot for `Education`-`16+ years`

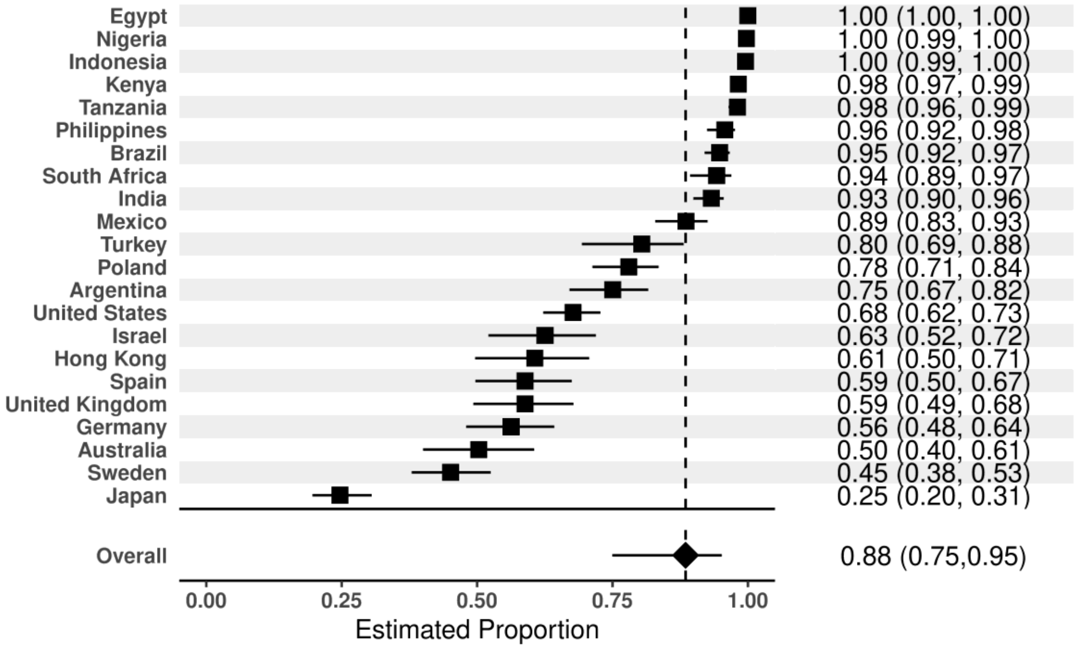

Probability-scale: τ=0.228;
Logit-scale: τ=2.239; Q(df=21)=1284.18, p<.001;Q-profile 95% CI [1.573, 3.011]; I^2=99.32;
Plot is based on back transformed bounds after using approximate logit SE that aren't guarenteed to match the robust SE of a proportion.

Figure S33. Forest plot for `Immigration status`-`Born in this country`

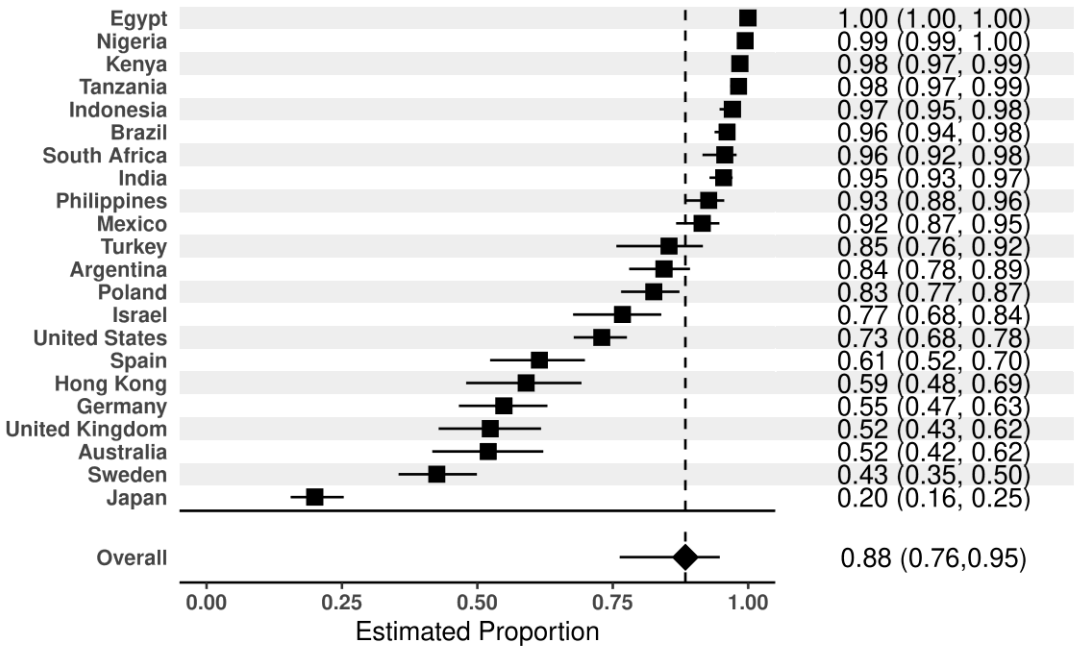

Probability-scale: τ=0.210;
Logit-scale: τ=2.049; Q(df=21)=1415.62, p<.001;Q-profile 95% CI [1.455, 2.763]; I^2=99.17;
Plot is based on back transformed bounds after using approximate logit SE that aren't guarenteed to match the robust SE of a proportion.

Figure S34. Forest plot for `Immigration status`-`Born in another country`

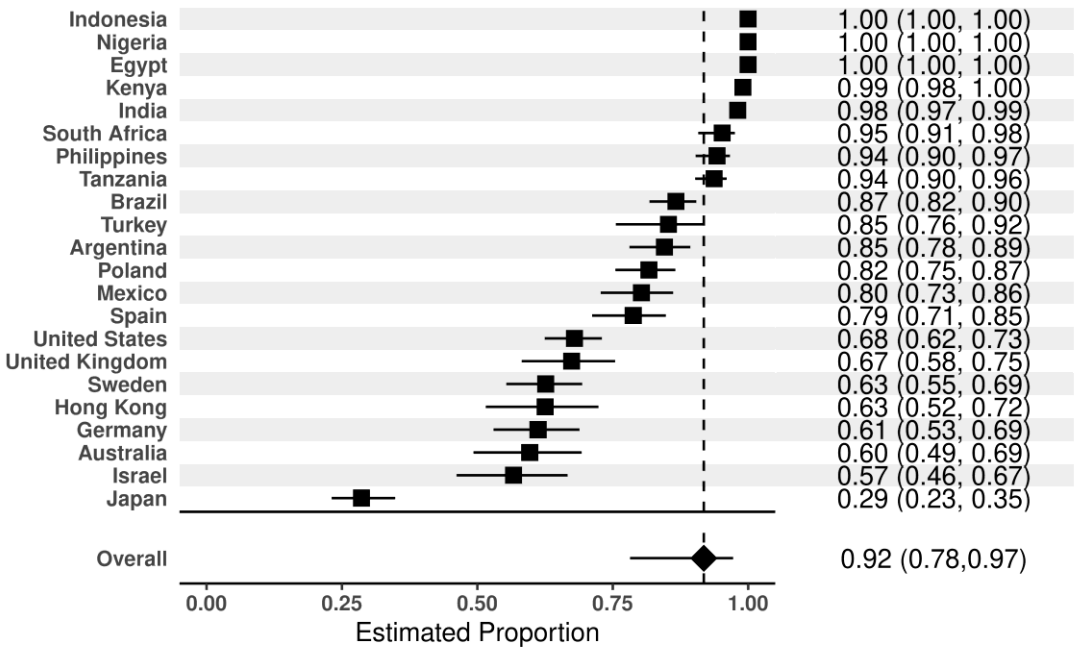

Probability-scale: τ=0.203;
Logit-scale: τ=2.699; Q(df=21)=988.84, p<.001;Q-profile 95% CI [1.847, 3.622]; I^2=99.52;
Plot is based on back transformed bounds after using approximate logit SE that aren't guarenteed to match the robust SE of a proportion.

Figure S35. Forest plot for `Age group`-`(Ref: 18-24) 25-29`

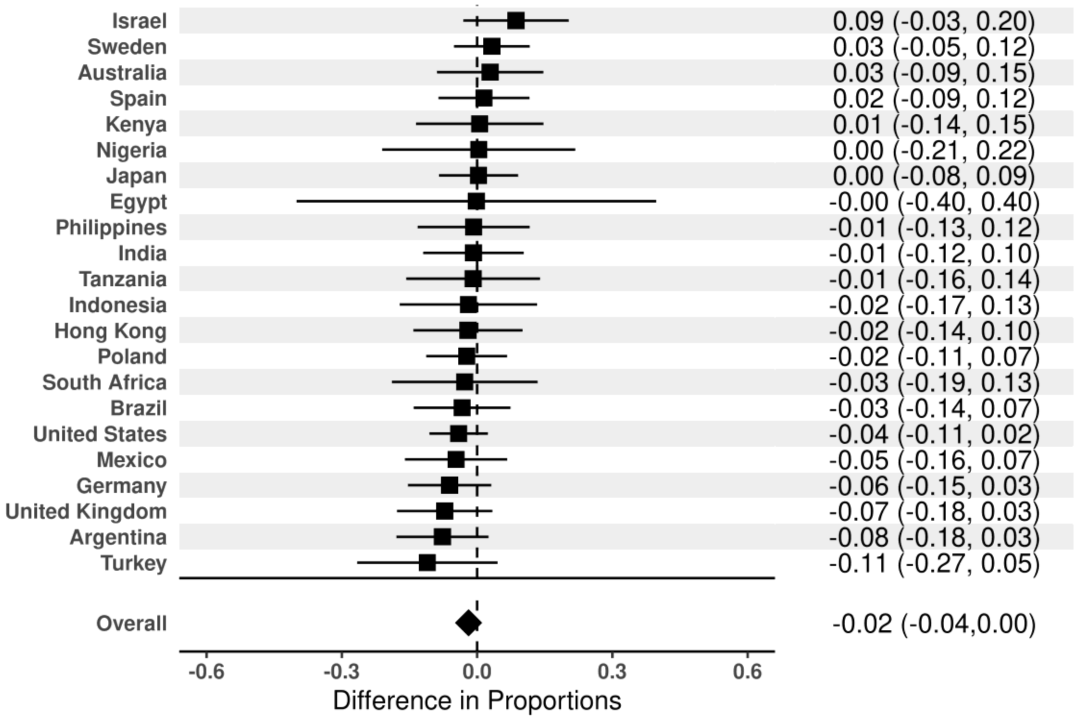

τ=0.000; Q(df=21)=11.22, p0.958;Q-profile 95% CI [0.000, 0.036]; I^2=0.00;

Figure S36. Forest plot for `Age group`-`(Ref: 18-24) 30-39`

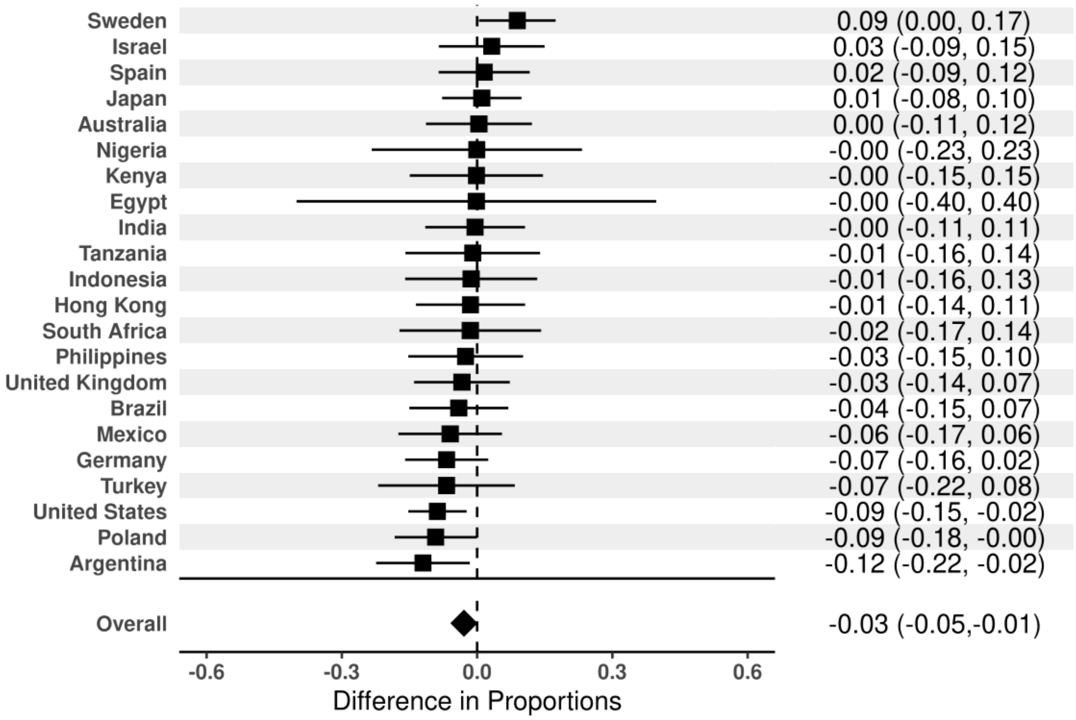

τ=0.000; Q(df=21)=20.10, p0.515;Q-profile 95% CI [0.000, 0.064]; I^2=0.00;

Figure S37. Forest plot for `Age group`-`(Ref: 18-24) 40-49`

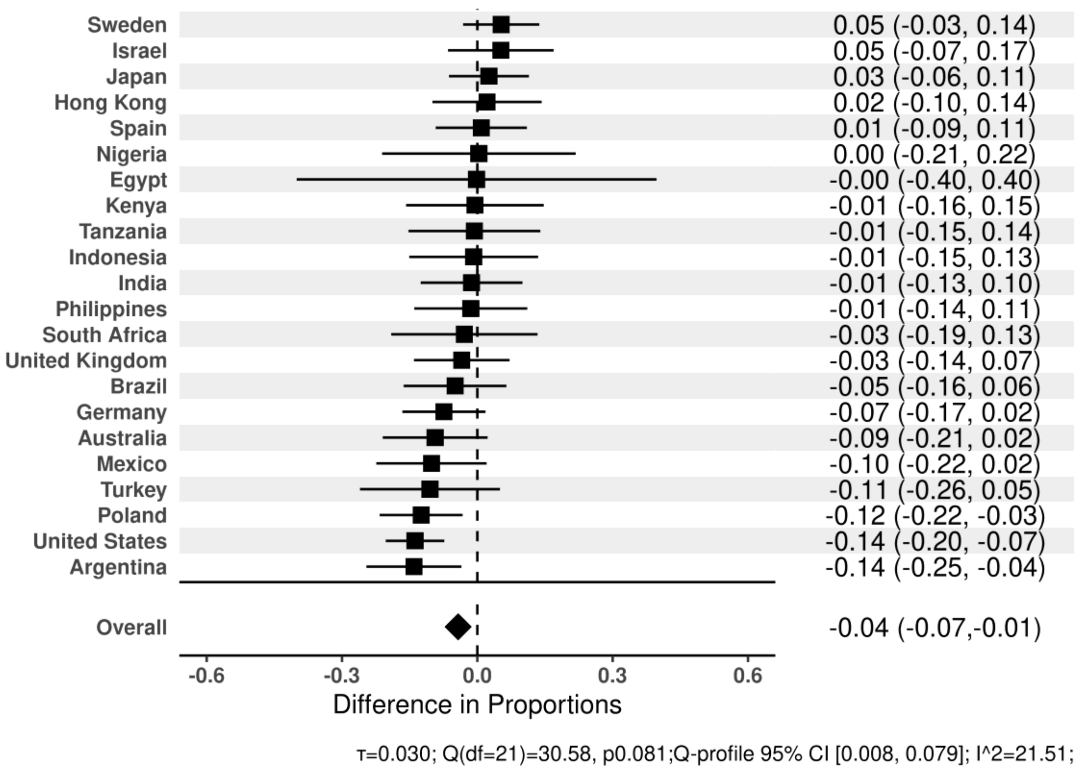

Figure S38. Forest plot for `Age group`-`(Ref: 18-24) 50-59`

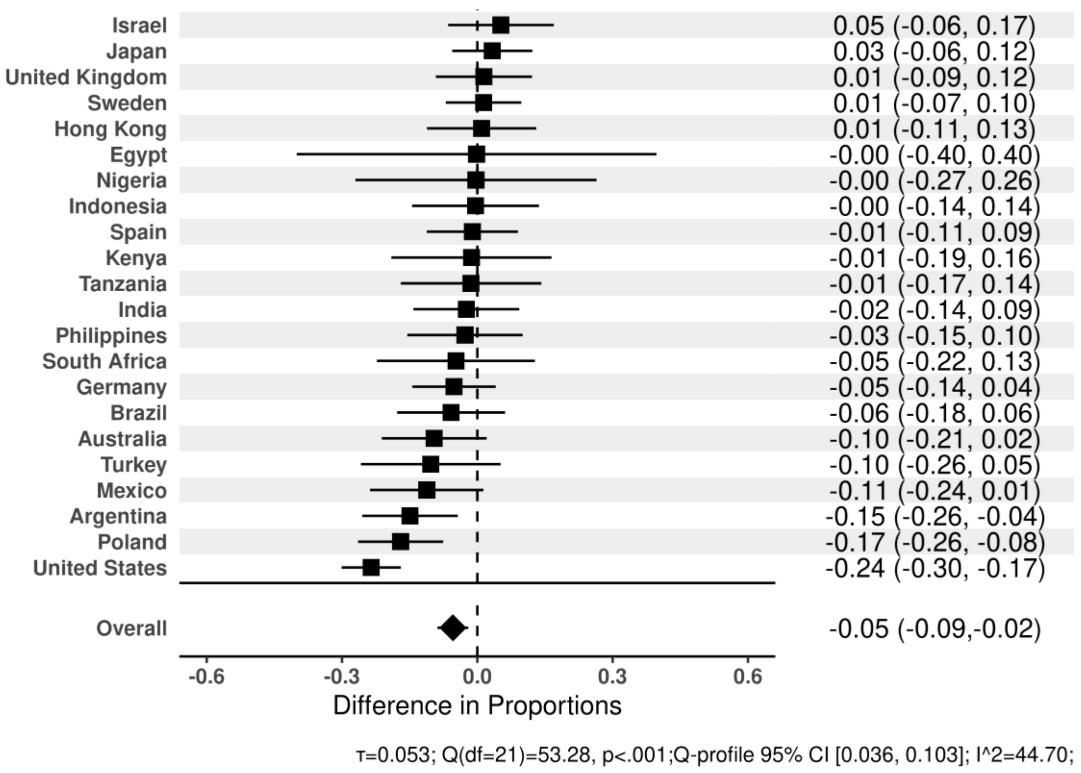

Figure S39. Forest plot for `Age group`-`(Ref: 18-24) 60-69`

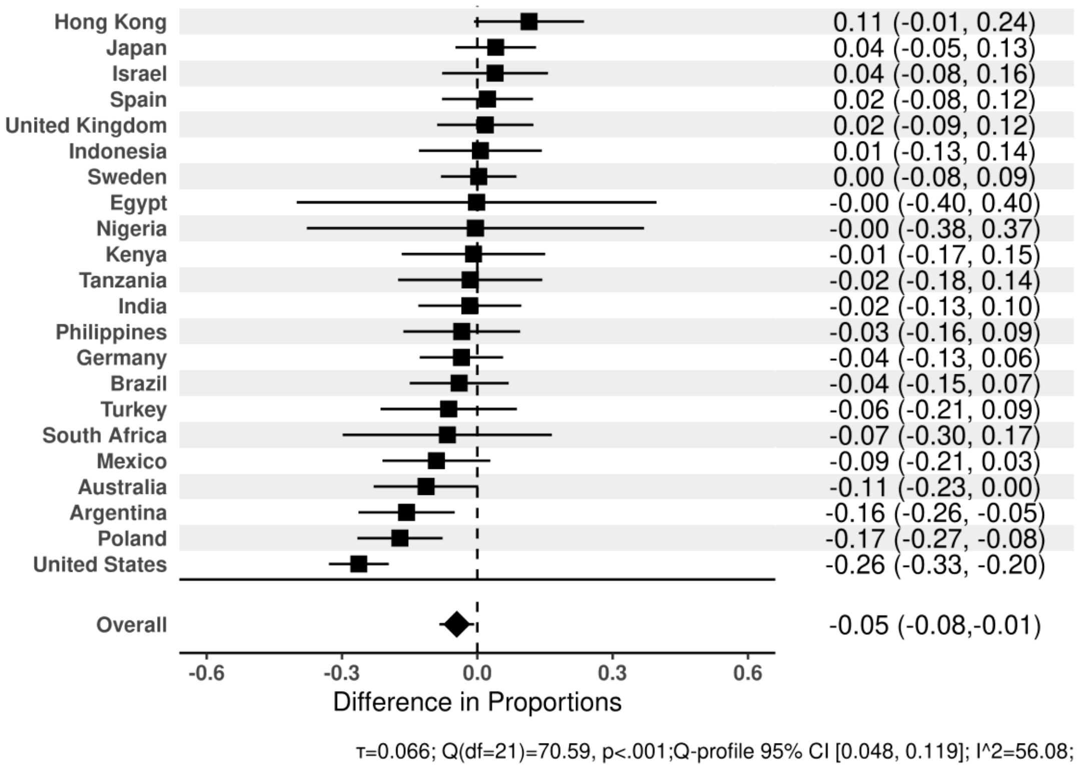

Figure S40. Forest plot for `Age group`-`(Ref: 18-24) 70-79`

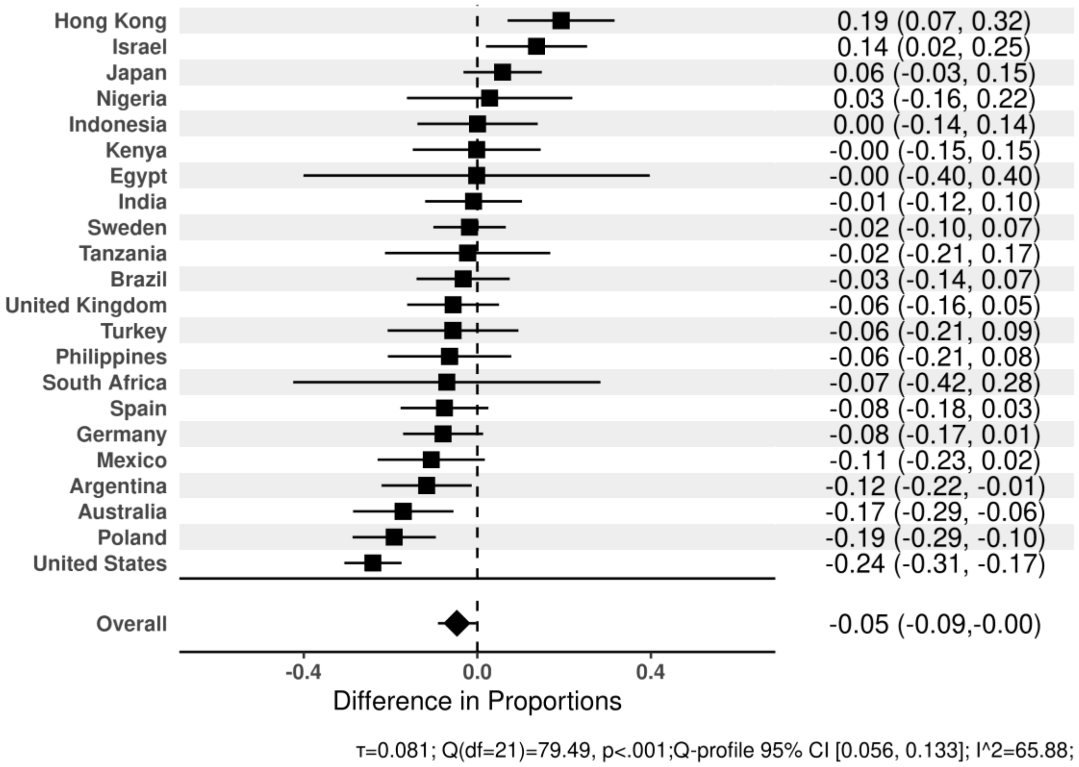

Figure S41. Forest plot for `Age group`-`(Ref: 18-24) 80 or older`

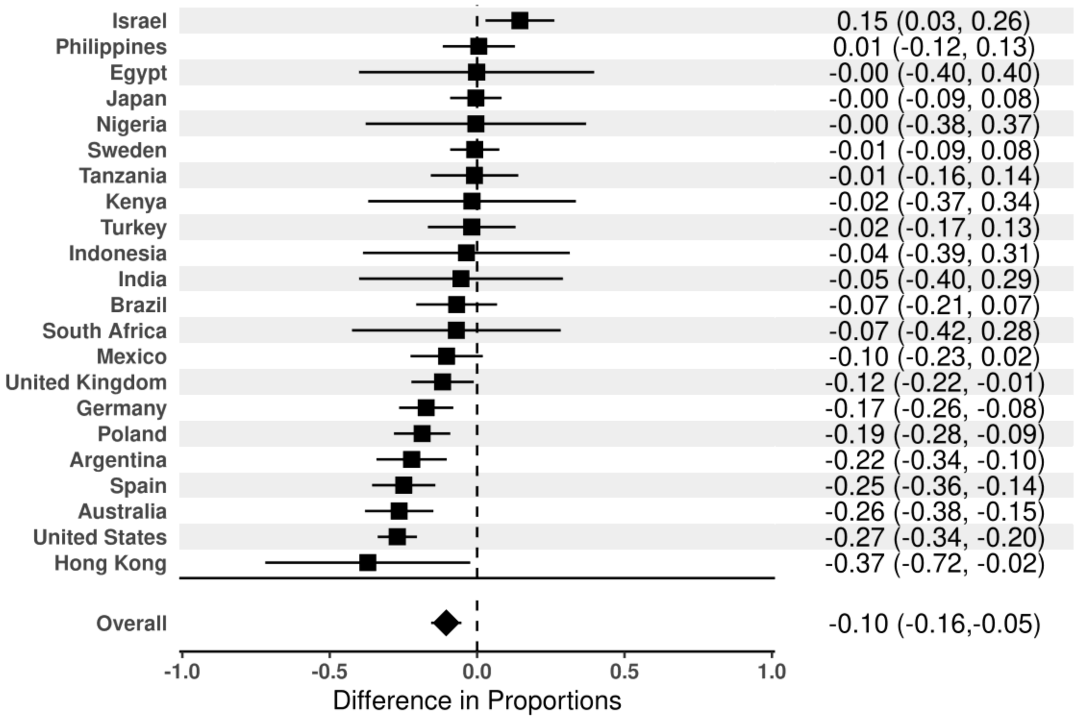

τ=0.092; Q(df=21)=82.68, p<.001;Q-profile 95% CI [0.065, 0.157]; I^2=67.49;

Figure S42. Forest plot for `Age group`-`(Ref: 25-29) 30-39`

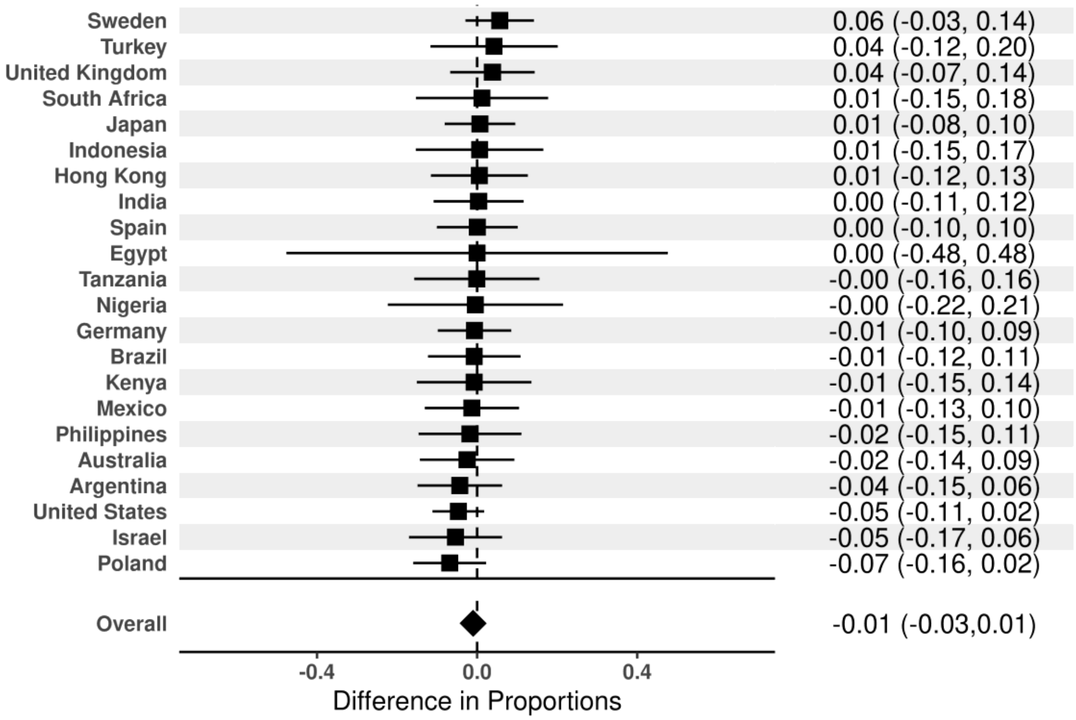

τ=0.000; Q(df=21)=7.84, p0.996;Q-profile 95% CI [0.000, 0.035]; I^2=0.00;

Figure S43. Forest plot for `Age group`-`(Ref: 25-29) 40-49`

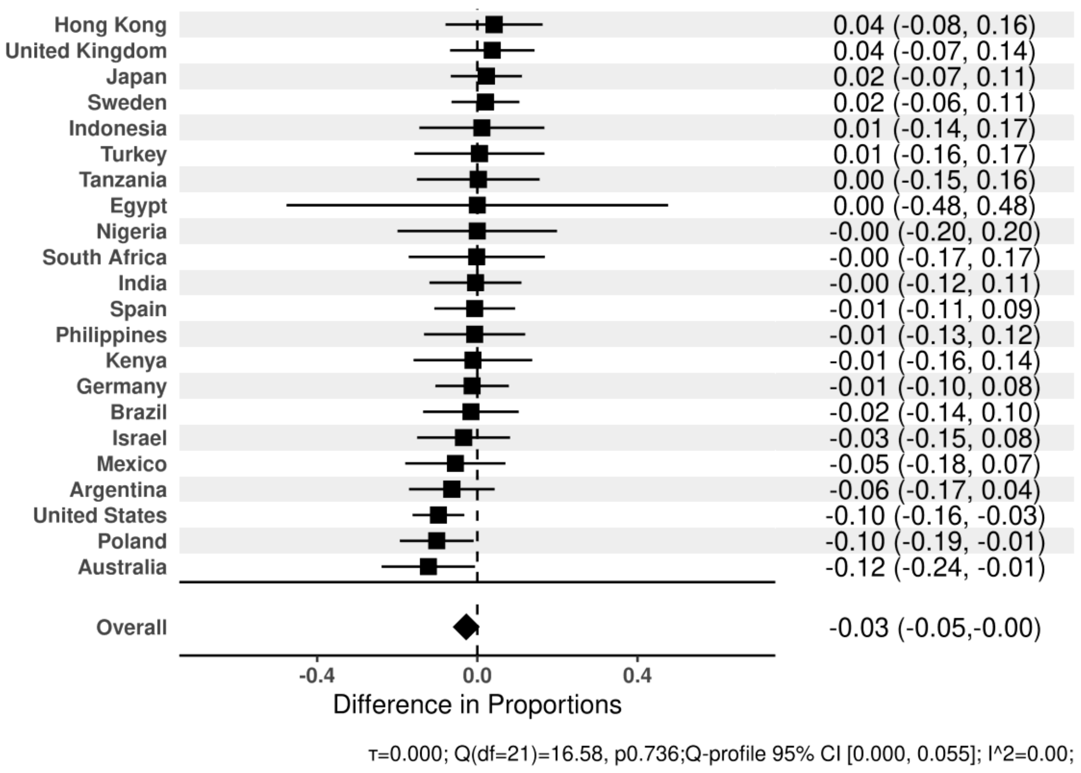

Figure S44. Forest plot for `Age group`-`(Ref: 25-29) 50-59`

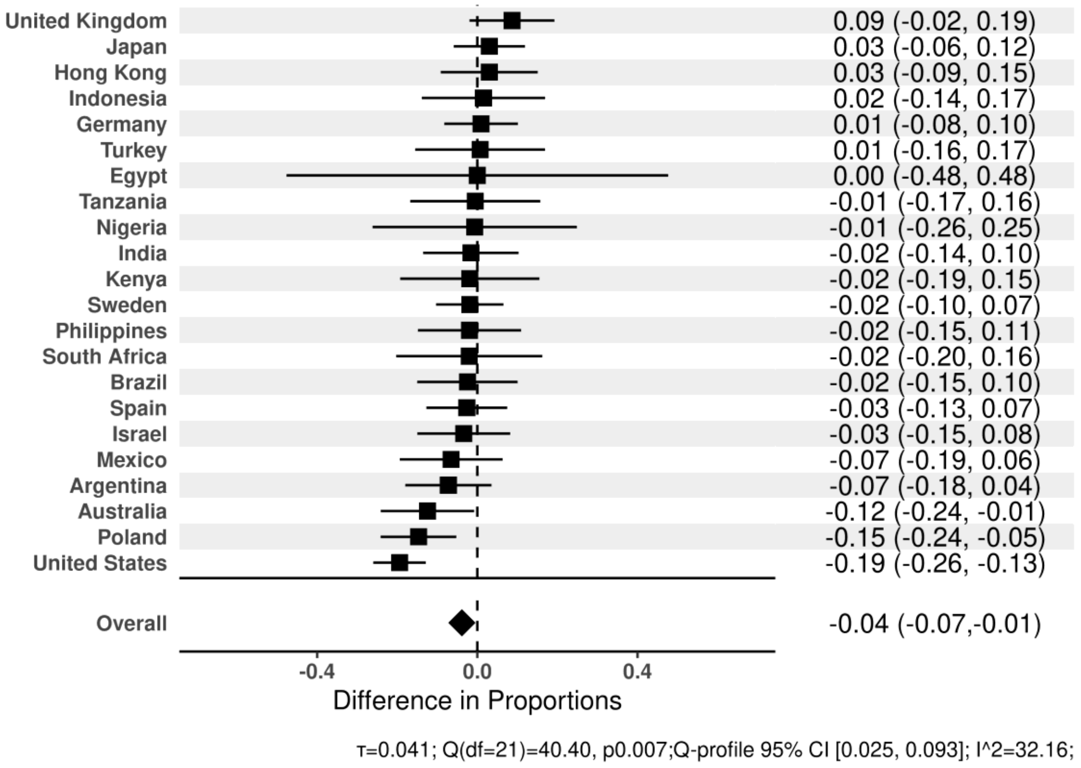

Figure S45. Forest plot for `Age group`-`(Ref: 25-29) 60-69`

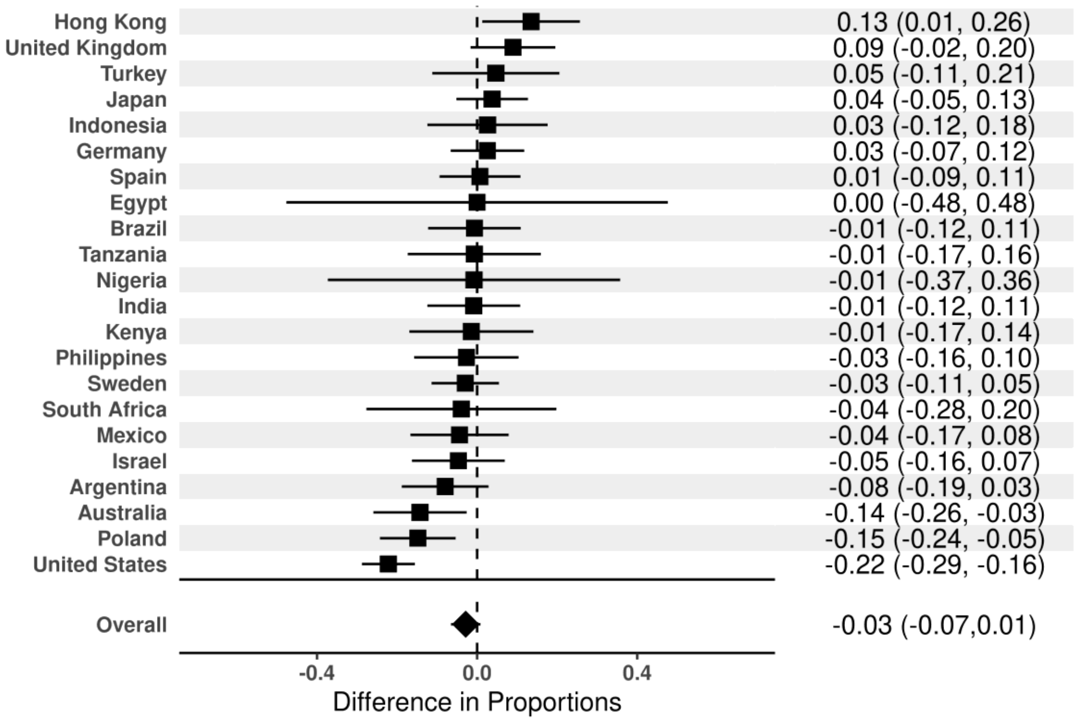

τ=0.060; Q(df=21)=59.67, p<.001;Q-profile 95% CI [0.042, 0.112]; I^2=50.69;

Figure S46. Forest plot for `Age group`-`(Ref: 25-29) 70-79`

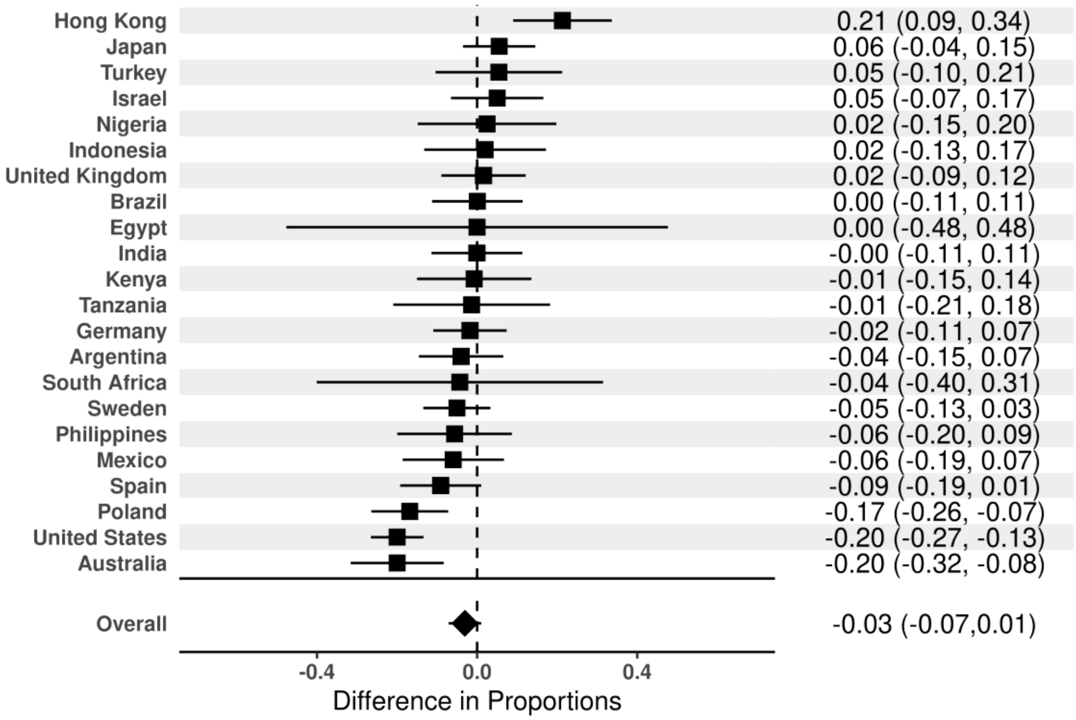

τ=0.072; Q(df=21)=65.51, p<.001;Q-profile 95% CI [0.048, 0.123]; I^2=59.82;

Figure S47. Forest plot for `Age group`-`(Ref: 25-29) 80 or older`

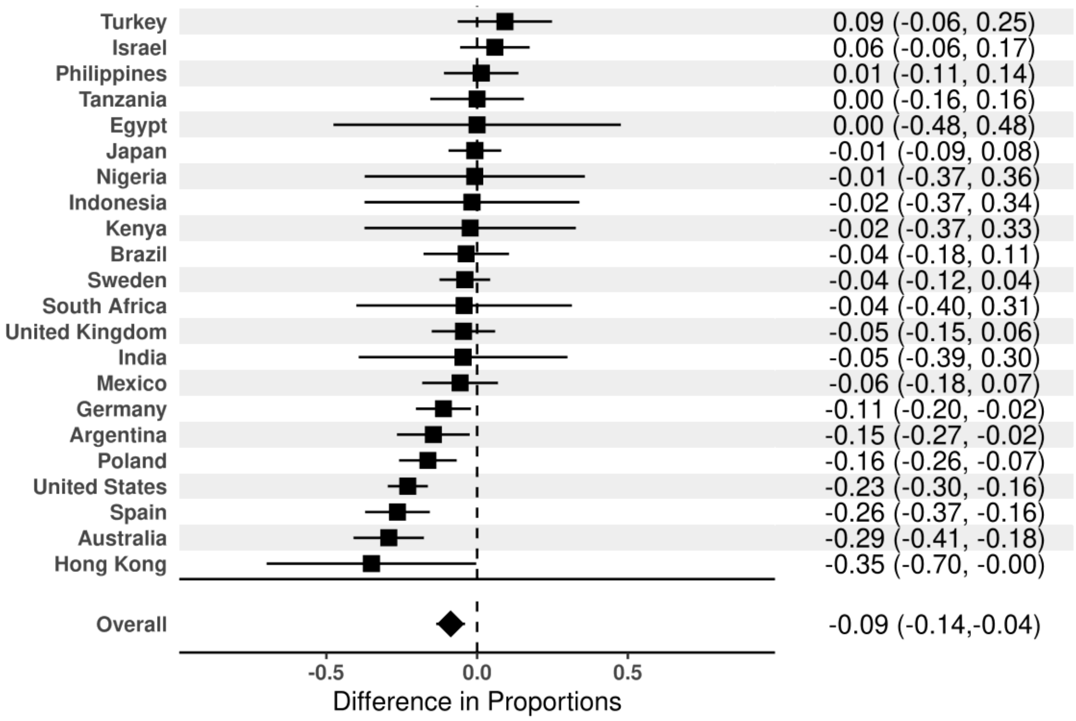

τ=0.082; Q(df=21)=65.81, p<.001;Q-profile 95% CI [0.054, 0.145]; I^2=61.88;

Figure S48. Forest plot for `Age group`-`(Ref: 30-39) 40-49`

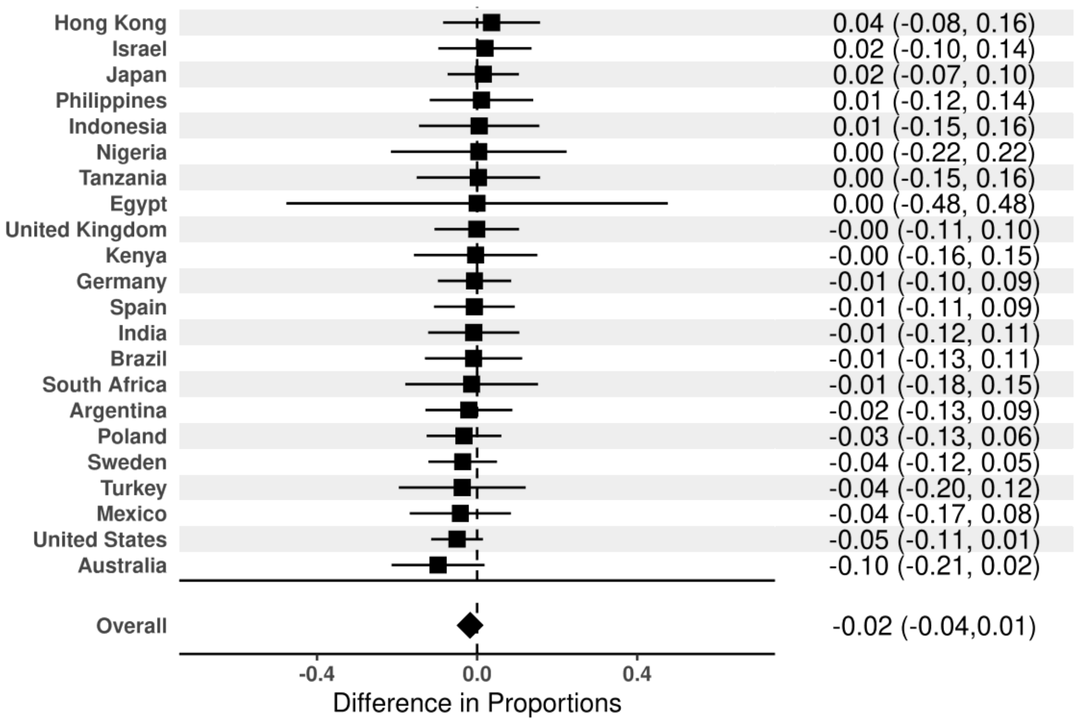

τ=0.000; Q(df=21)=5.62, p1.000;Q-profile 95% CI [0.000, 0.031]; I^2=0.00;

Figure S49. Forest plot for `Age group`-`(Ref: 30-39) 50-59`

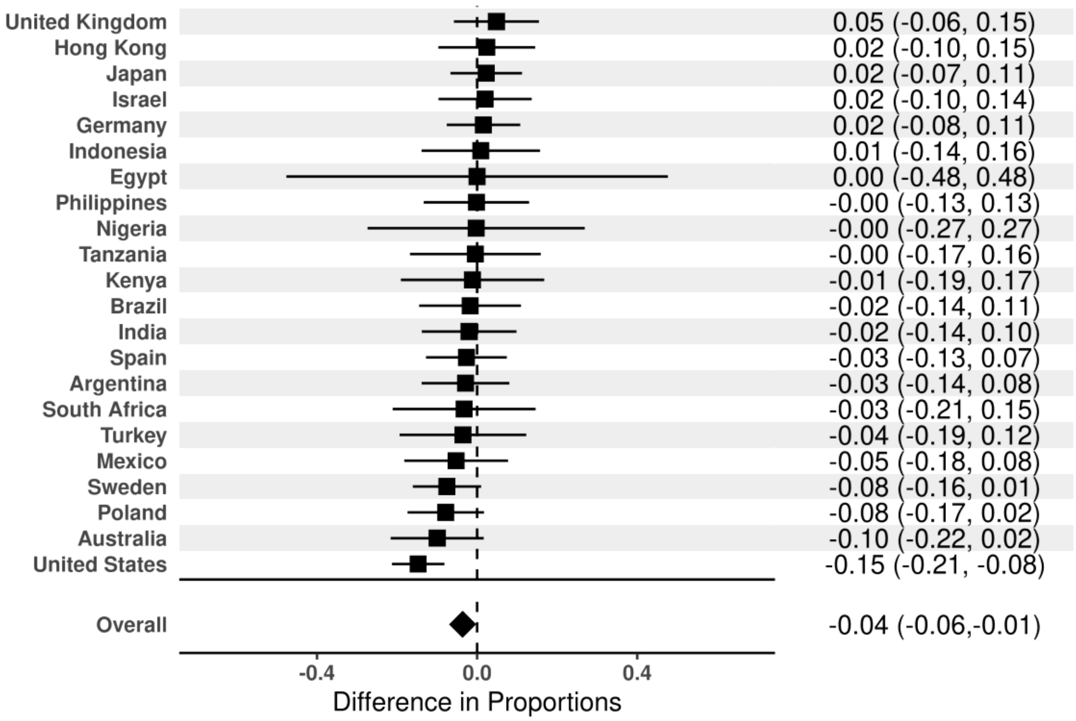

τ=0.011; Q(df=21)=22.10, p0.394;Q-profile 95% CI [0.000, 0.075]; I^2=3.27;

Figure S50. Forest plot for `Age group`-`(Ref: 30-39) 60-69`

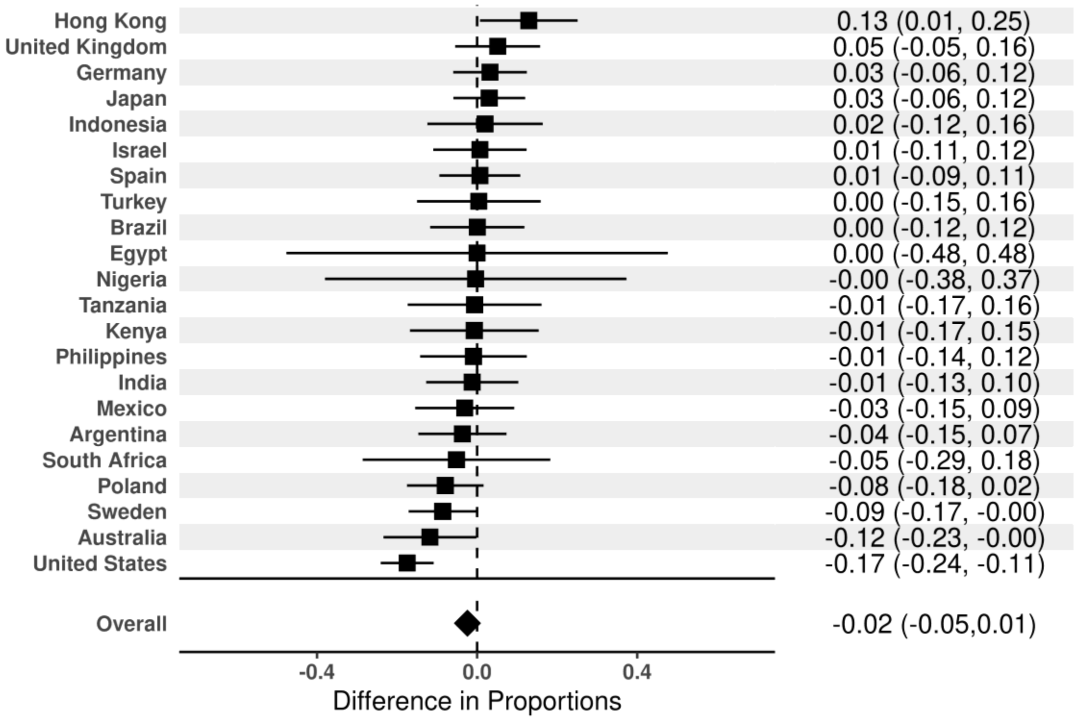

τ=0.038; Q(df=21)=37.76, p0.014;Q-profile 95% CI [0.023, 0.092]; I^2=29.69;

Figure S51. Forest plot for `Age group`-`(Ref: 30-39) 70-79`

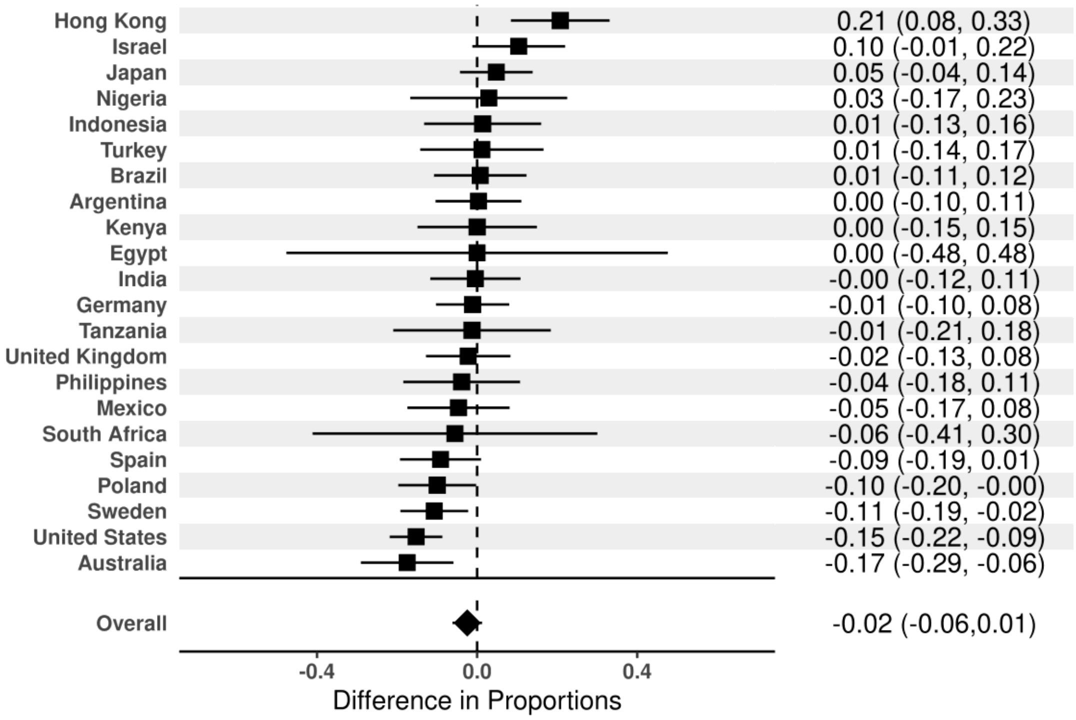

τ=0.060; Q(df=21)=50.30, p<.001;Q-profile 95% CI [0.037, 0.109]; I^2=50.78;

Figure S52. Forest plot for `Age group`-`(Ref: 30-39) 80 or older`

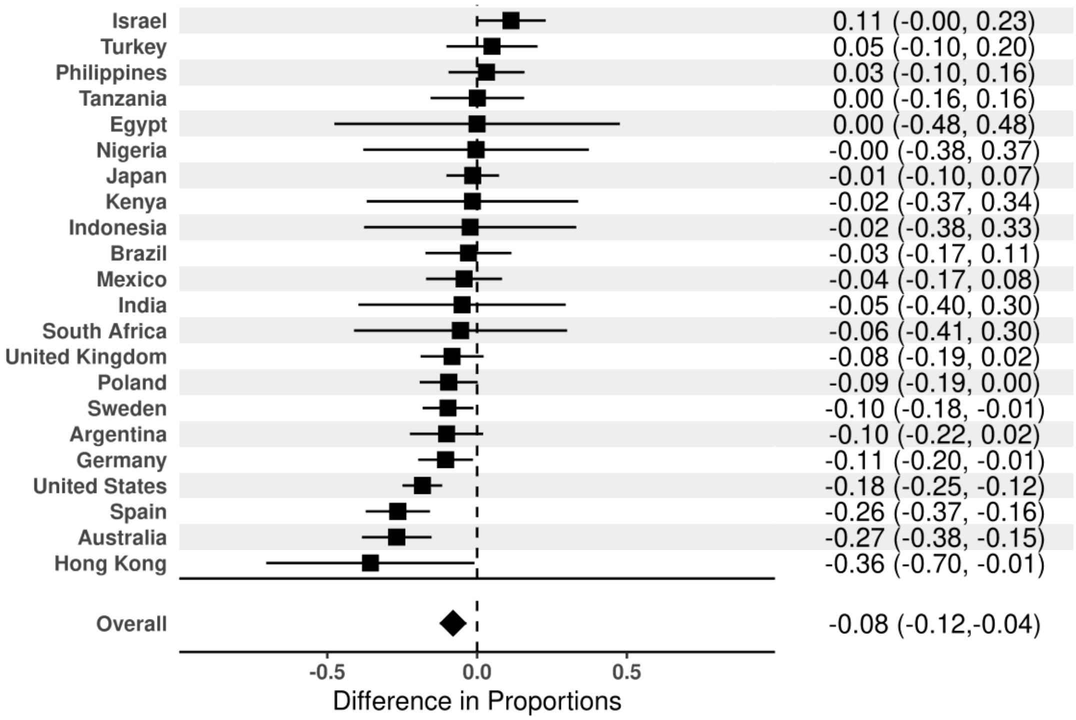

τ=0.073; Q(df=21)=53.71, p<.001;Q-profile 95% CI [0.045, 0.134]; I^2=56.10;

Figure S53. Forest plot for `Age group`-`(Ref: 40-49) 50-59`

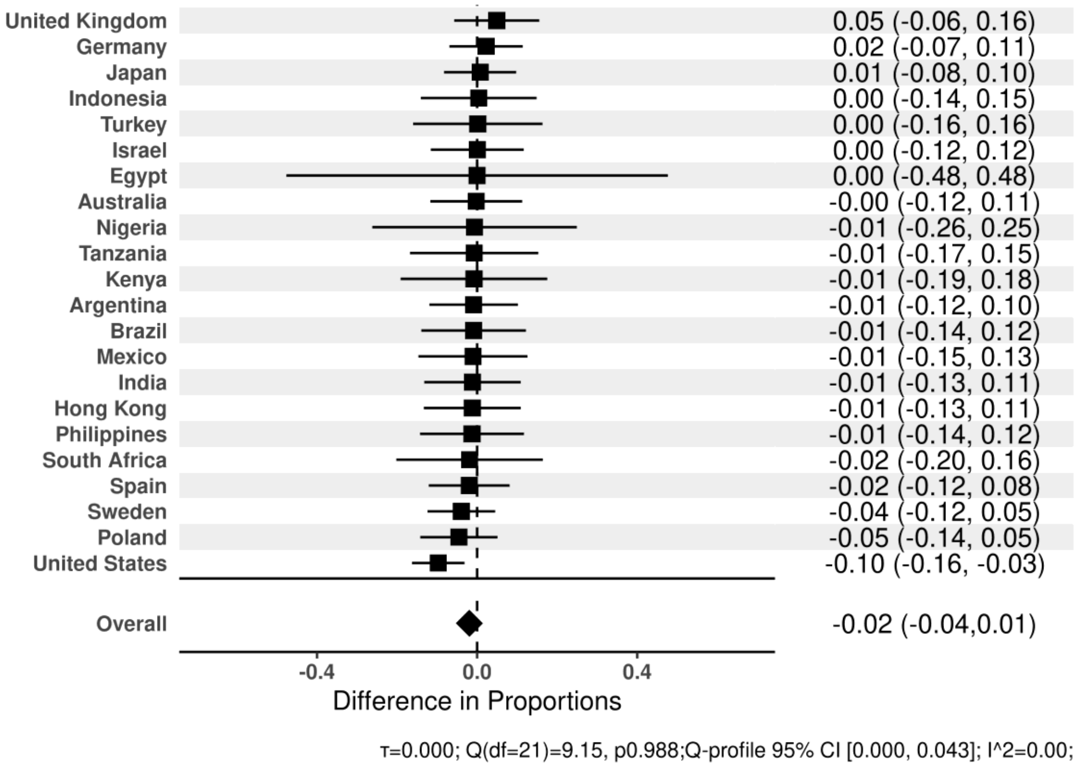

Figure S54. Forest plot for `Age group`-`(Ref: 40-49) 60-69`

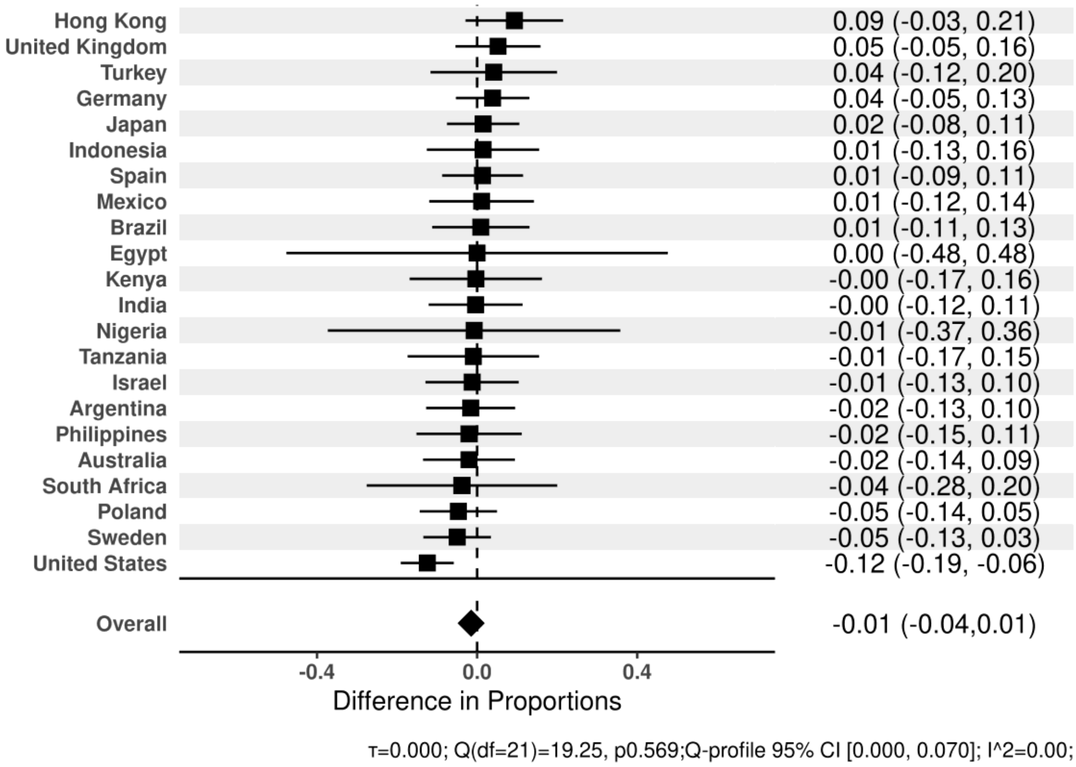

Figure S55. Forest plot for `Age group`-`(Ref: 40-49) 70-79`

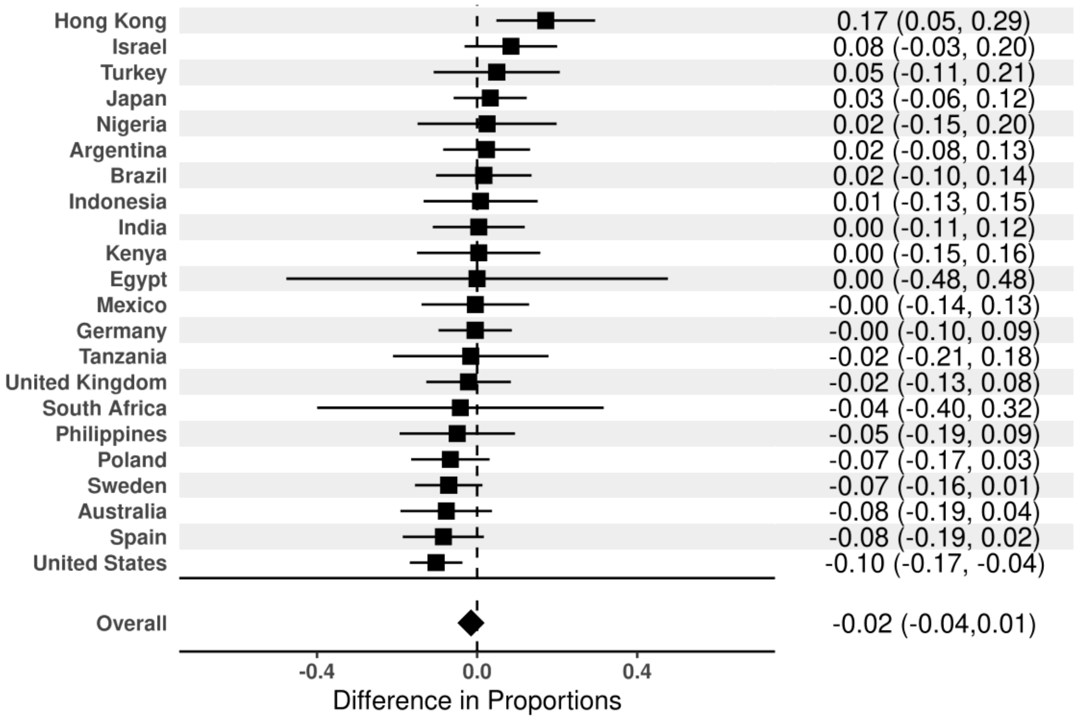

τ=0.027; Q(df=21)=27.23, p0.163;Q-profile 95% CI [0.000, 0.078]; I^2=16.77;

Figure S56. Forest plot for `Age group`-`(Ref: 40-49) 80 or older`

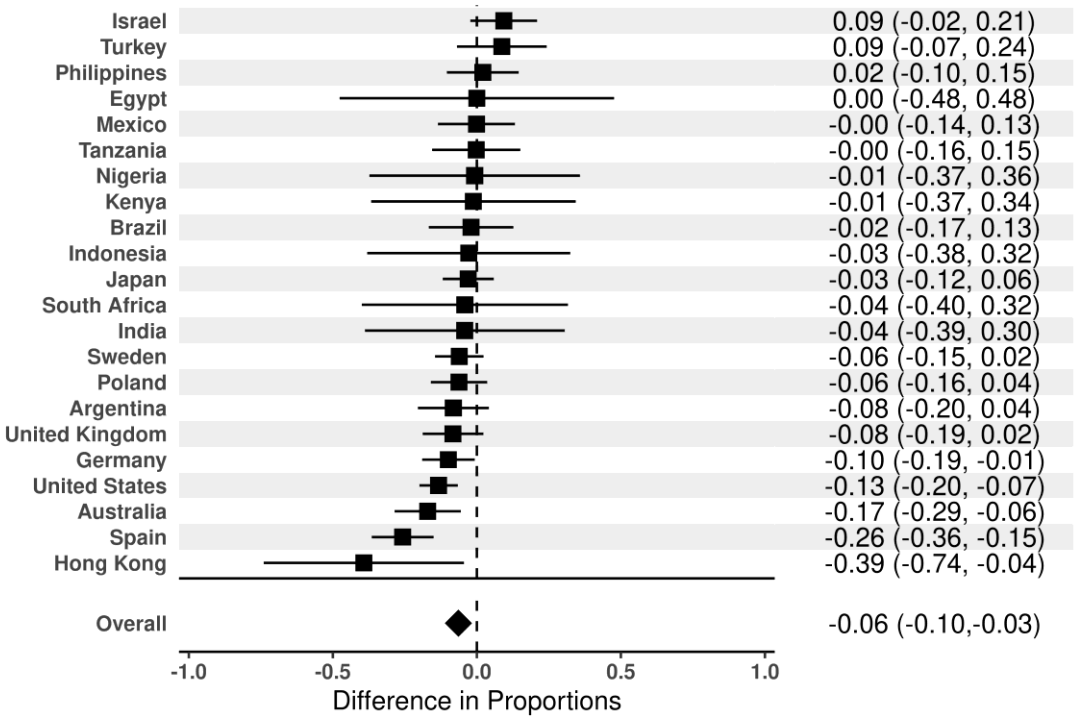

τ=0.057; Q(df=21)=38.88, p0.010;Q-profile 95% CI [0.022, 0.111]; I^2=43.31;

Figure S57. Forest plot for `Age group`-`(Ref: 50-59) 60-69`

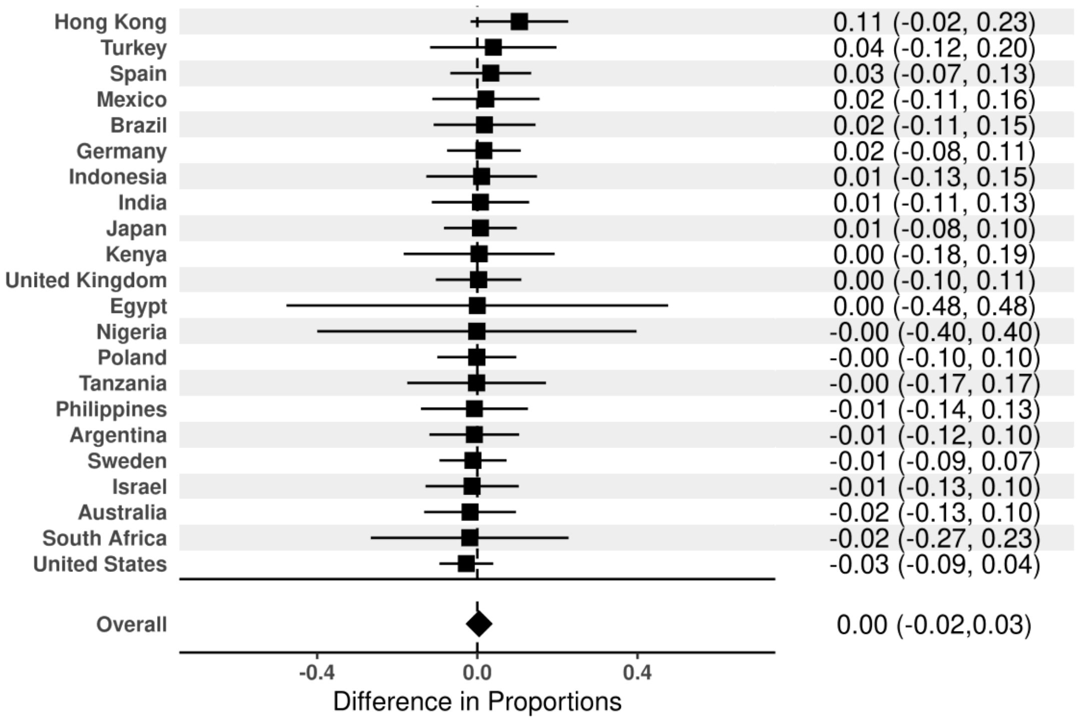

τ=0.000; Q(df=21)=4.66, p1.000;Q-profile 95% CI [0.000, 0.031]; I^2=0.00;

Figure S58. Forest plot for `Age group`-`(Ref: 50-59) 70-79`

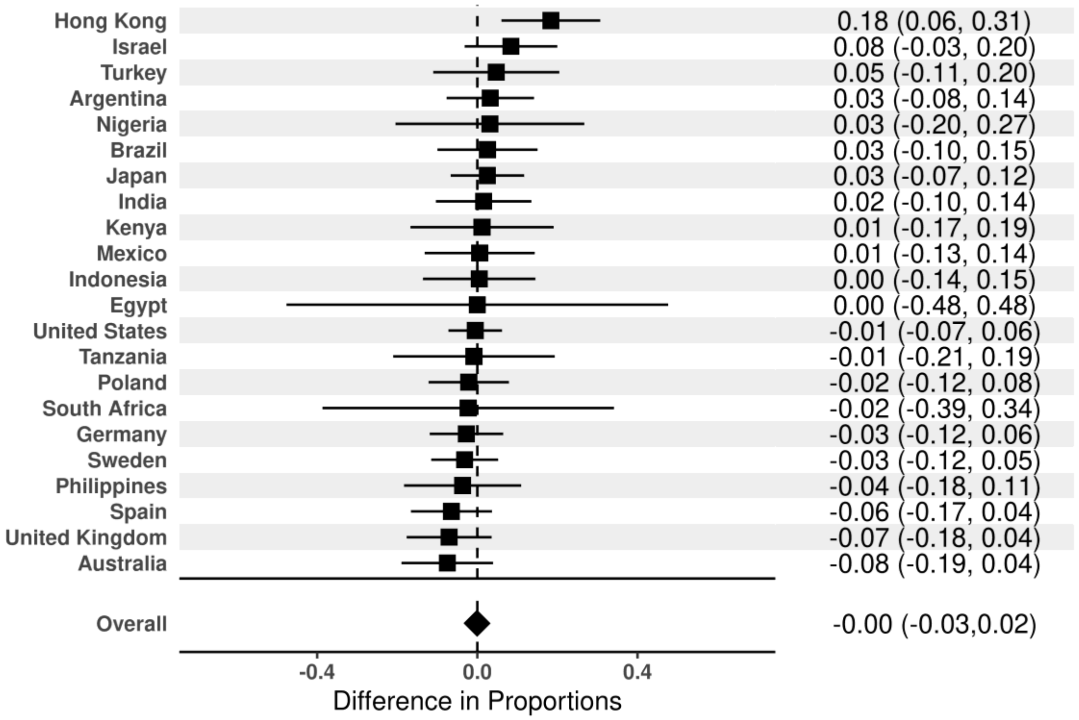

τ=0.000; Q(df=21)=18.10, p0.643;Q-profile 95% CI [0.000, 0.053]; I^2=0.00;

Figure S59. Forest plot for `Age group`-`(Ref: 50-59) 80 or older`

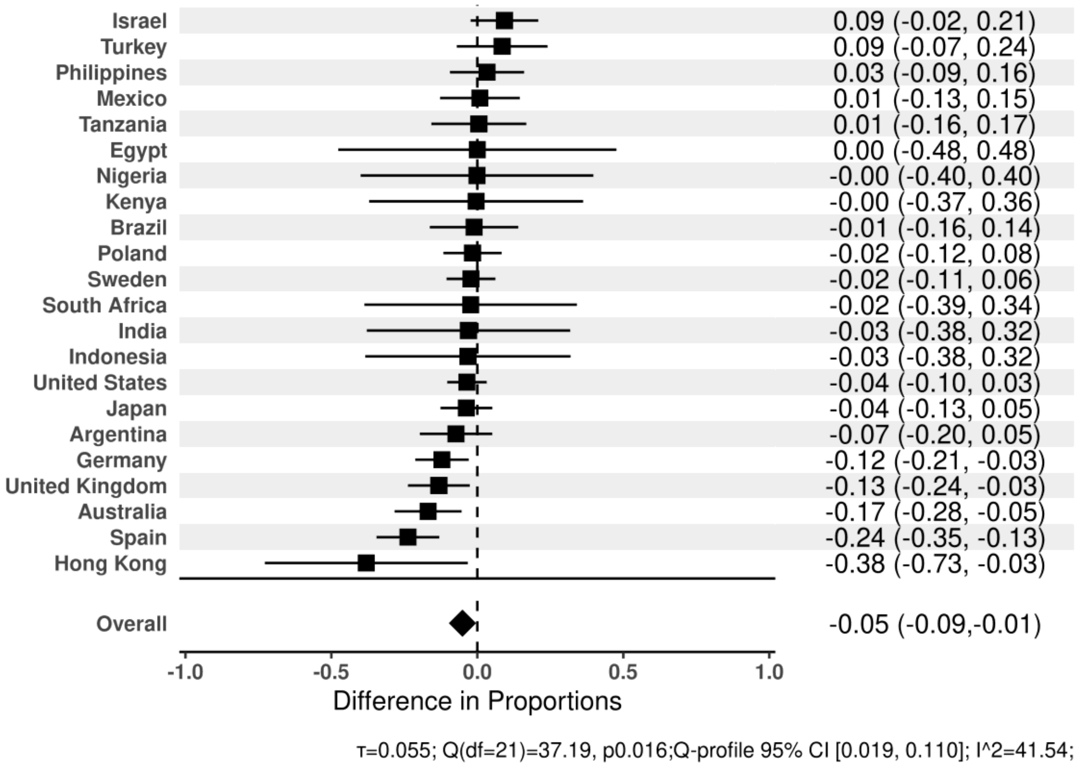

Figure S60. Forest plot for `Age group`-`(Ref: 60-69) 70-79`

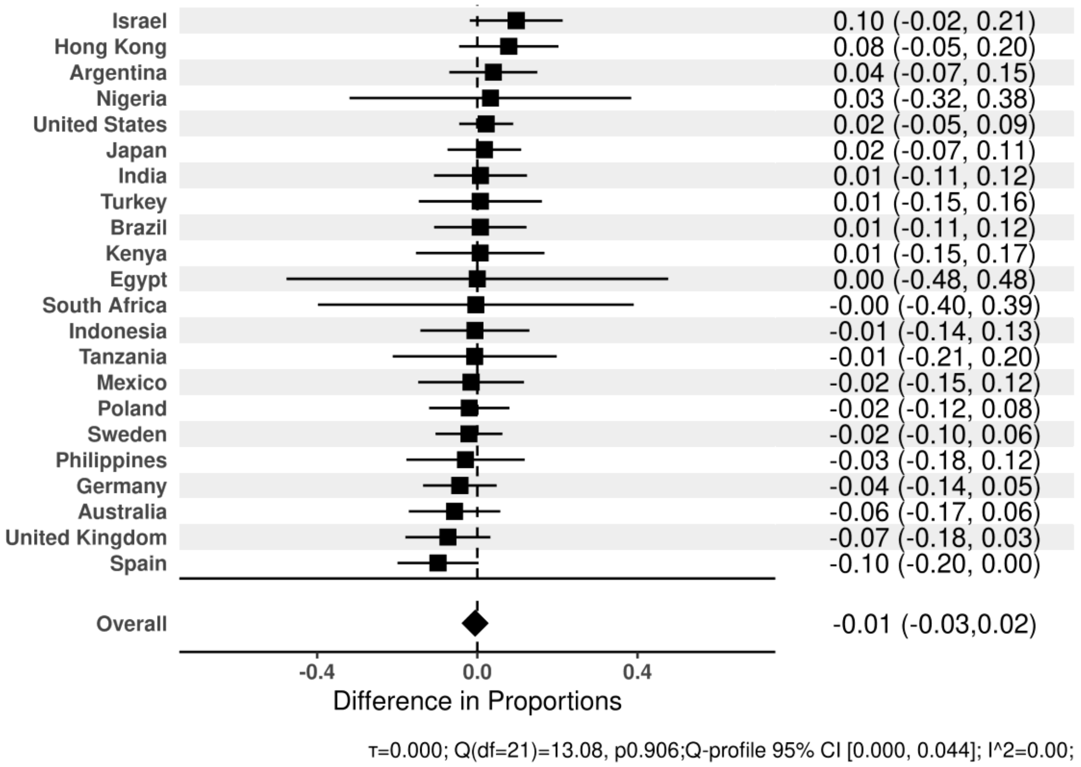

Figure S61. Forest plot for `Age group`-`(Ref: 60-69) 80 or older`

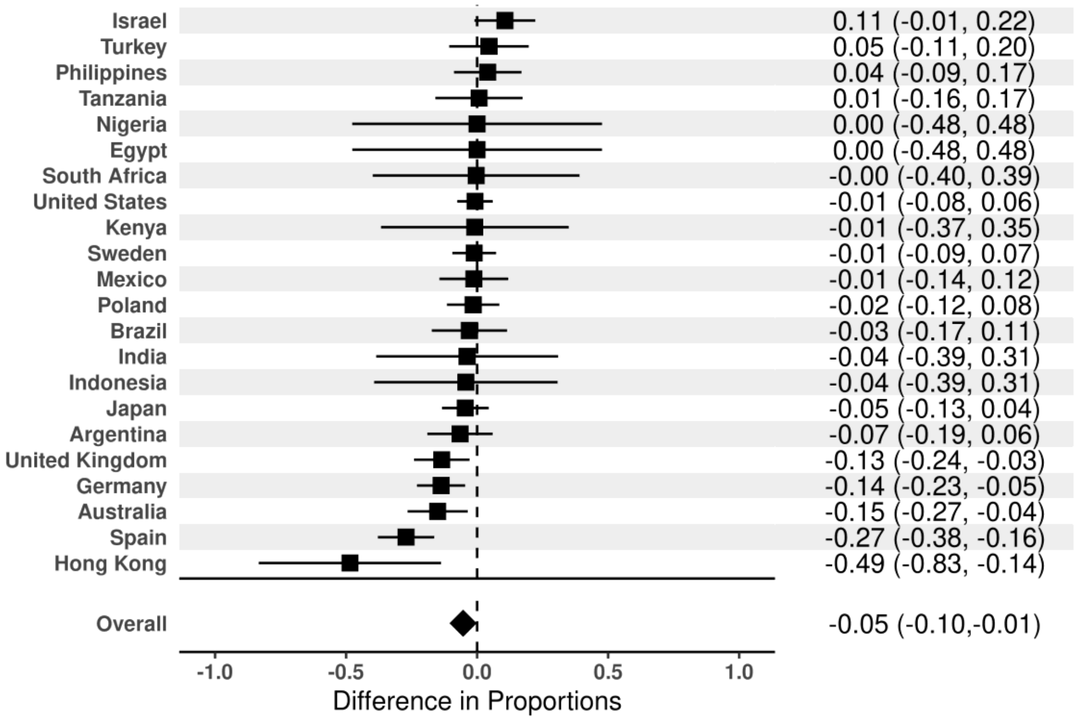

τ=0.069; Q(df=21)=45.47, p0.002;Q-profile 95% CI [0.034, 0.122]; I^2=52.47;

Figure S62. Forest plot for `Age group`-`(Ref: 70-79) 80 or older`

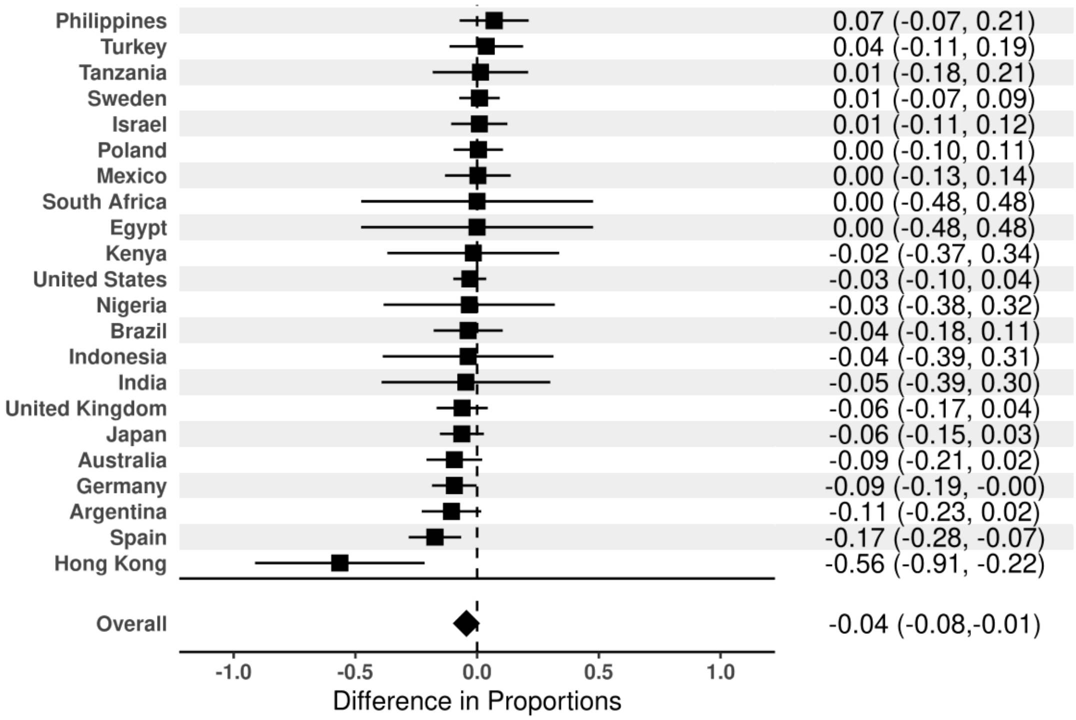

τ=0.031; Q(df=21)=25.17, p0.240;Q-profile 95% CI [0.000, 0.071]; I^2=18.62;

Figure S63. Forest plot for `Gender`-`(Ref: Male) Female`

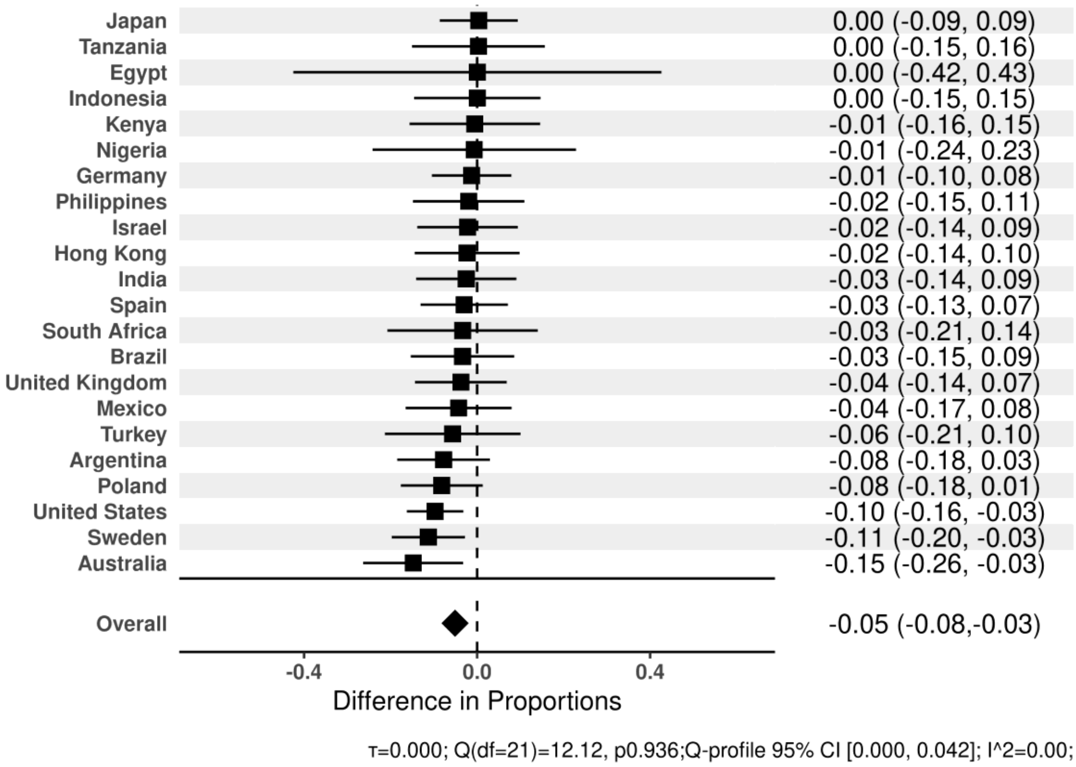

Figure S64. Forest plot for `Gender`-`(Ref: Male) Other`

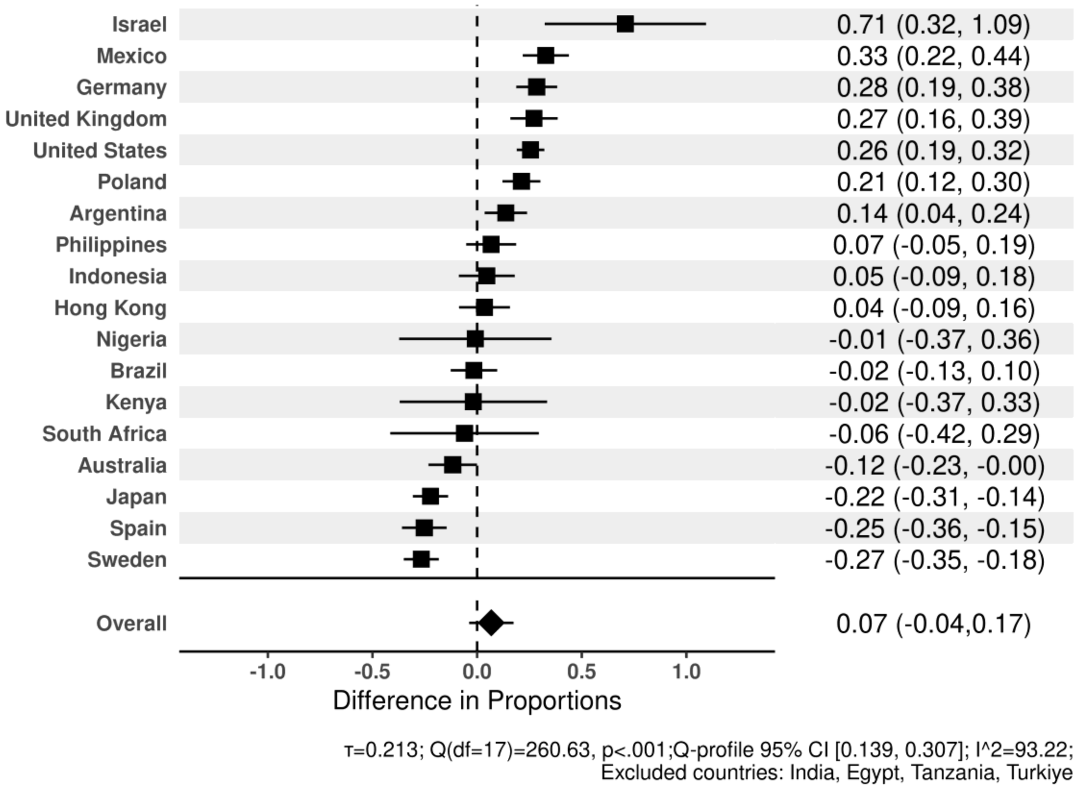

Figure S65. Forest plot for `Gender`-`(Ref: Female) Other`

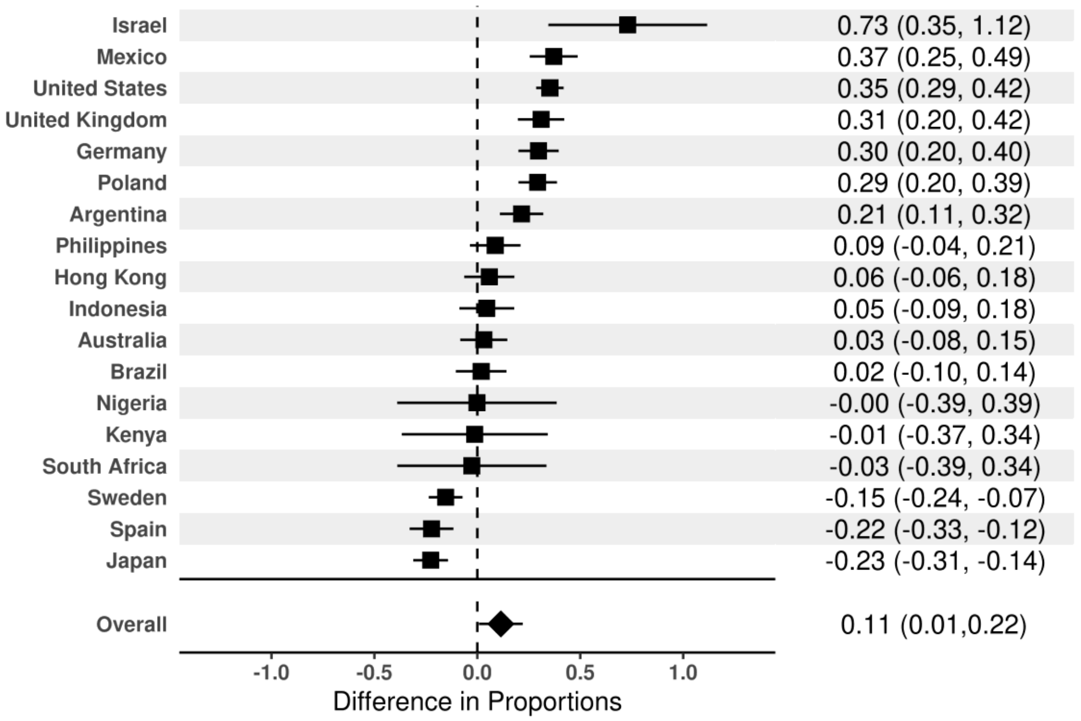

τ=0.212; Q(df=17)=269.95, p<.001;Q-profile 95% CI [0.140, 0.307]; I^2=93.02;
Excluded countries: India, Egypt, Tanzania, Turkiye

Figure S66. Forest plot for `Marital status`-`(Ref: Married) Separated`

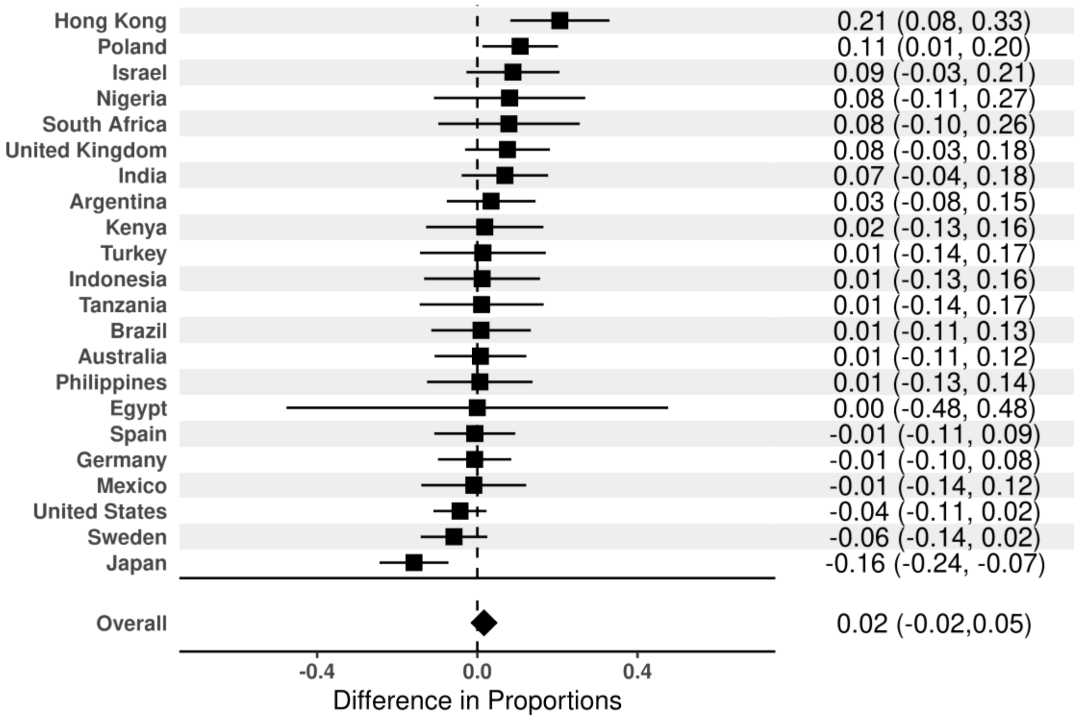

τ=0.047; Q(df=21)=39.45, p0.009;Q-profile 95% CI [0.024, 0.093]; I^2=39.10;

Figure S67. Forest plot for `Marital status`-`(Ref: Married) Divorced`

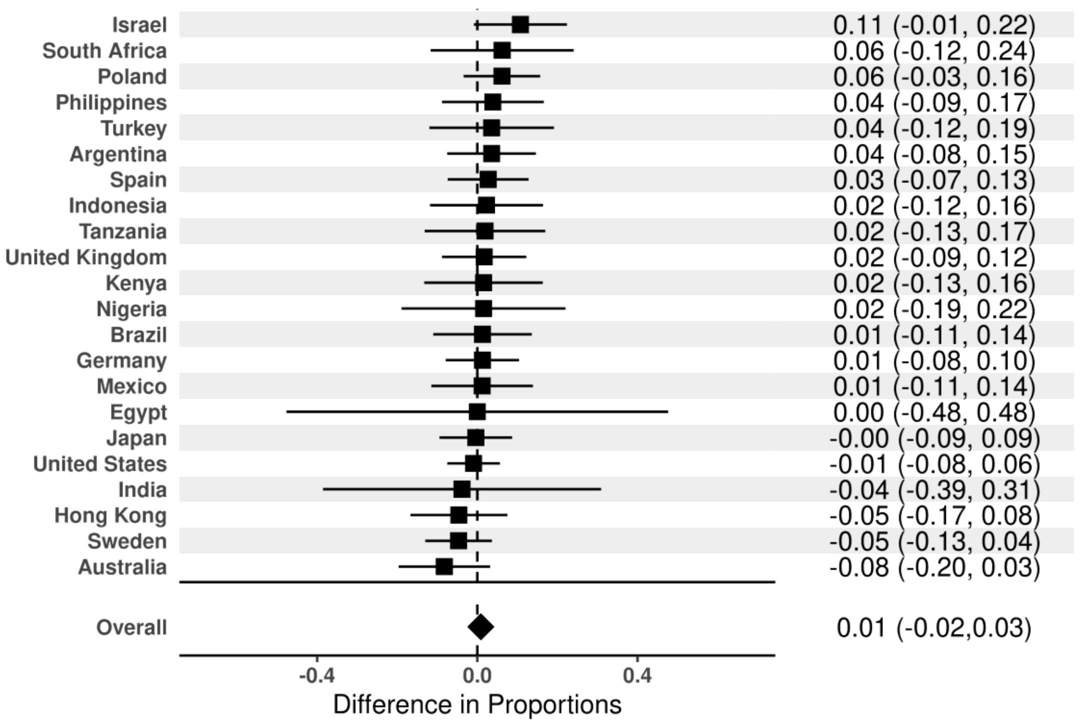

τ=0.000; Q(df=21)=10.43, p0.973;Q-profile 95% CI [0.000, 0.038]; I^2=0.00;

Figure S68. Forest plot for `Marital status`-`(Ref: Married) Widowed`

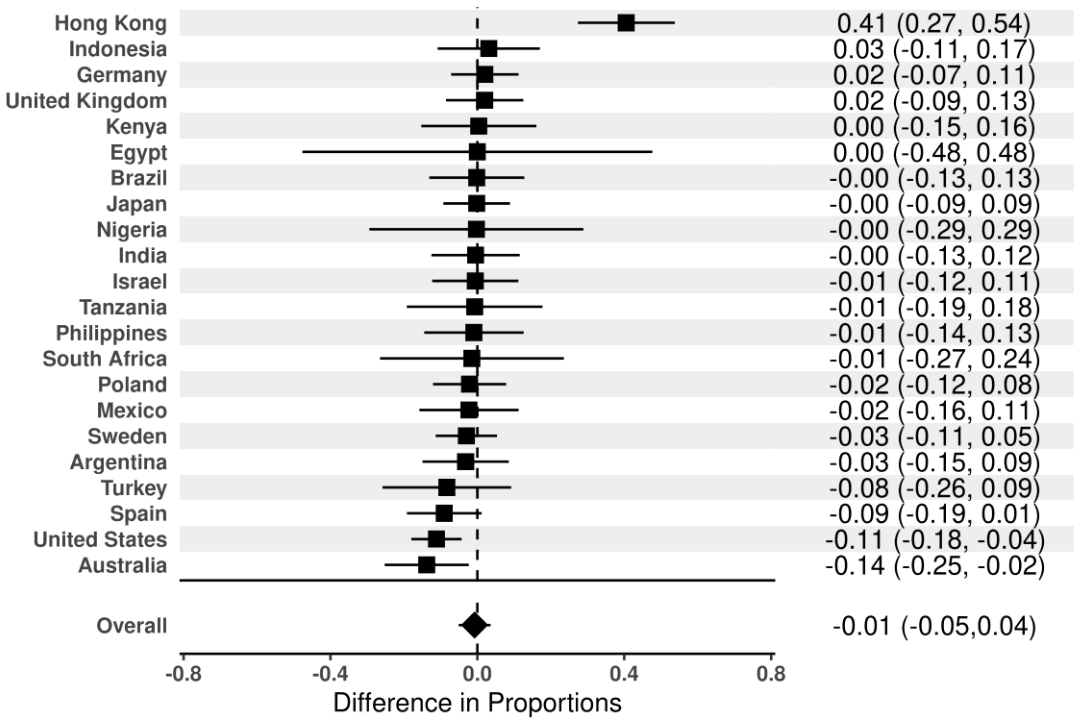

τ=0.078; Q(df=21)=55.61, p<.001;Q-profile 95% CI [0.044, 0.128]; I^2=62.41;

Figure S69. Forest plot for `Marital status`-`(Ref: Married) Single, never married`

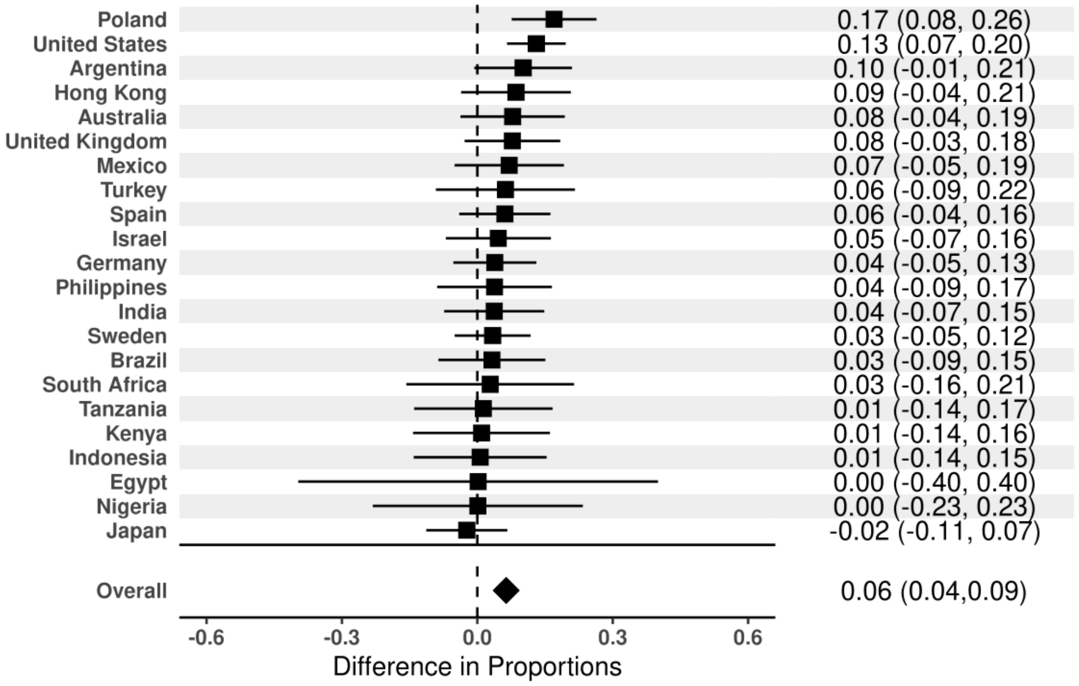

τ=0.000; Q(df=21)=16.84, p0.721;Q-profile 95% CI [0.000, 0.056]; I^2=0.00;

Figure S70. Forest plot for `Marital status`-`(Ref: Married) Domestic partner`

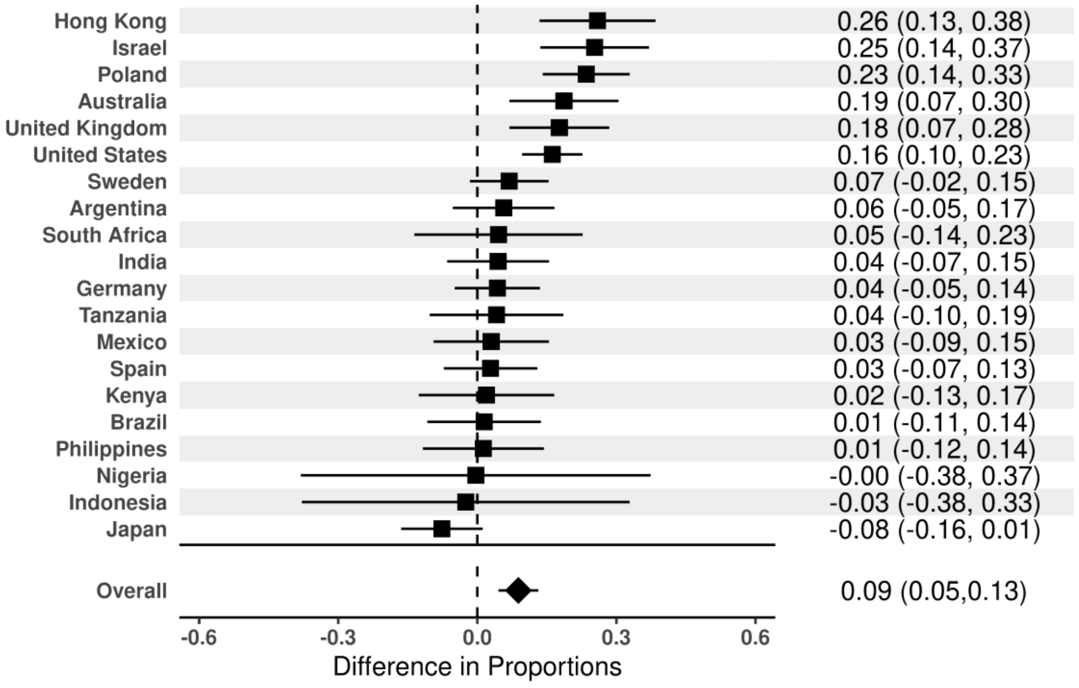

τ=0.074; Q(df=19)=56.55, p<.001;Q-profile 95% CI [0.045, 0.124]; I^2=62.90;
Excluded countries: Egypt, Turkiye

Figure S71. Forest plot for `Marital status`-`(Ref: Separated) Divorced`

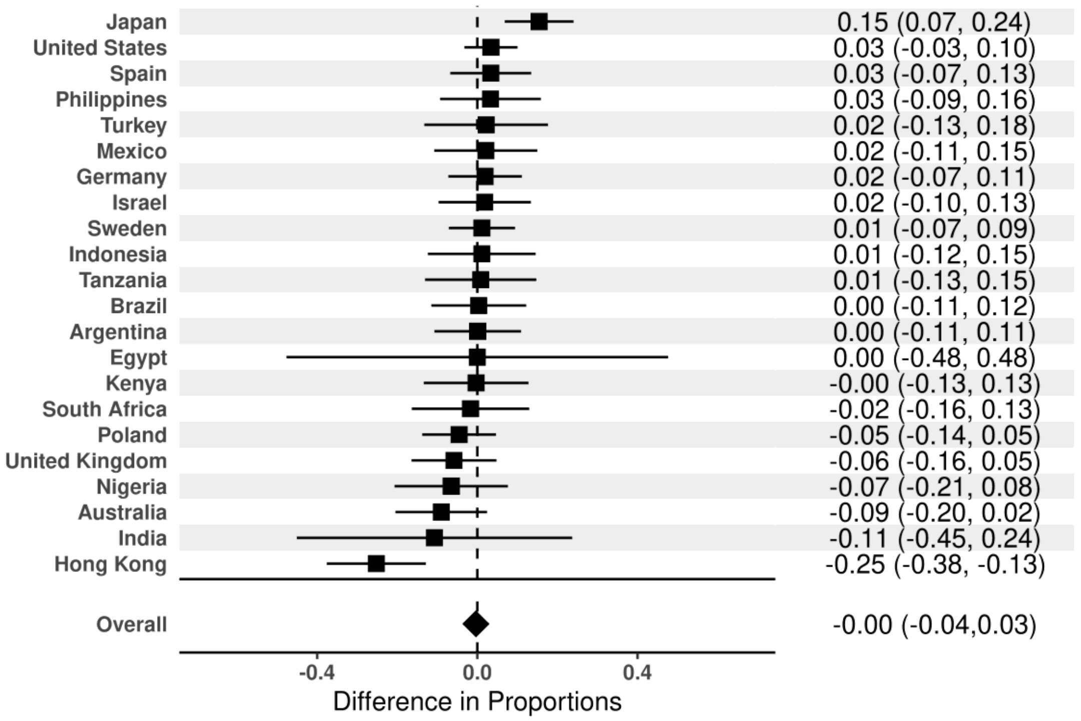

τ=0.046; Q(df=21)=36.34, p0.020;Q-profile 95% CI [0.013, 0.089]; I^2=39.00;

Figure S72. Forest plot for `Marital status`-`(Ref: Separated) Widowed`

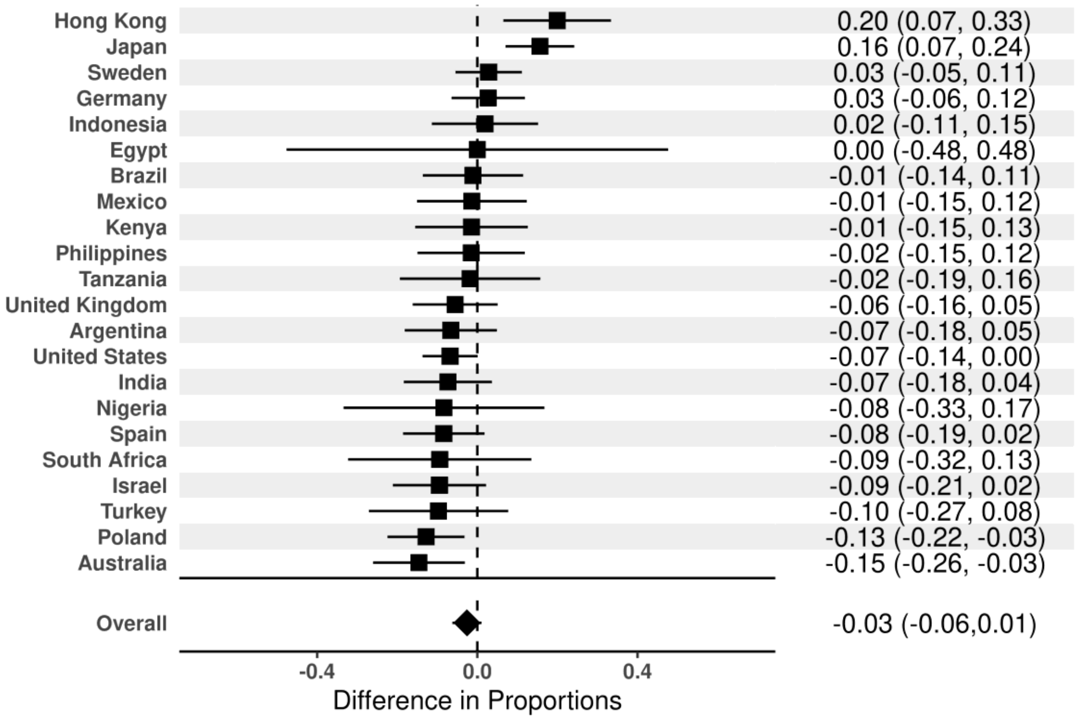

τ=0.059; Q(df=21)=46.81, p<.001;Q-profile 95% CI [0.033, 0.106]; I^2=49.38;

Figure S73. Forest plot for `Marital status`-`(Ref: Separated) Single, never married`

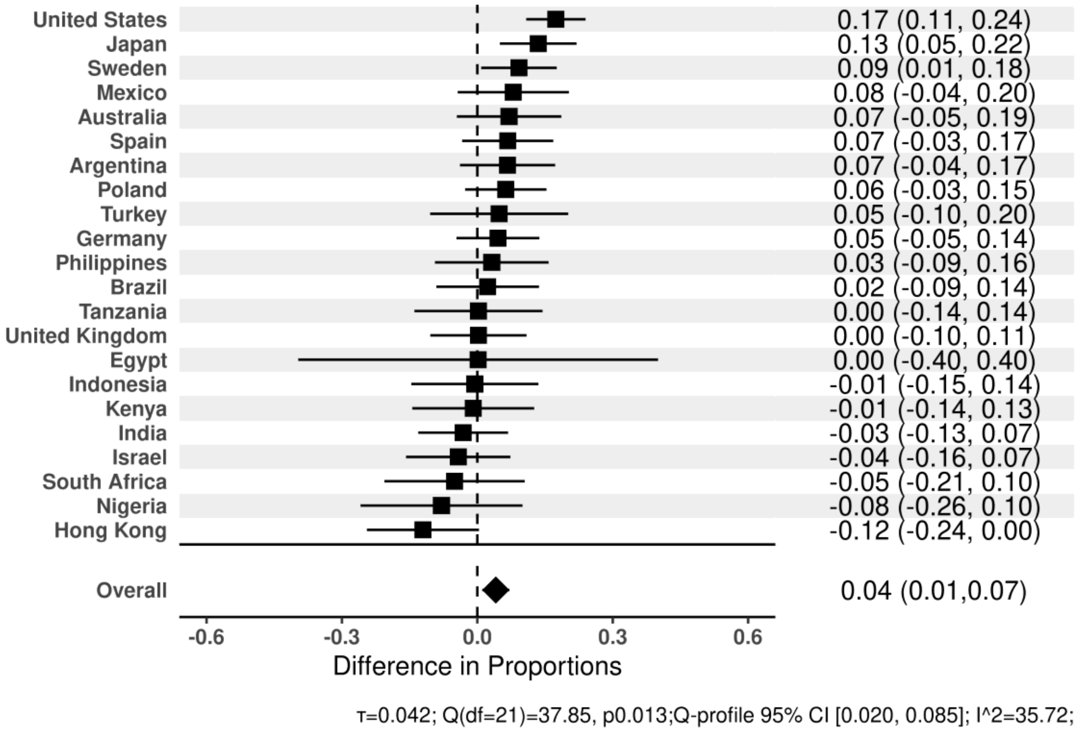

Figure S74. Forest plot for `Marital status`-`(Ref: Separated) Domestic partner`

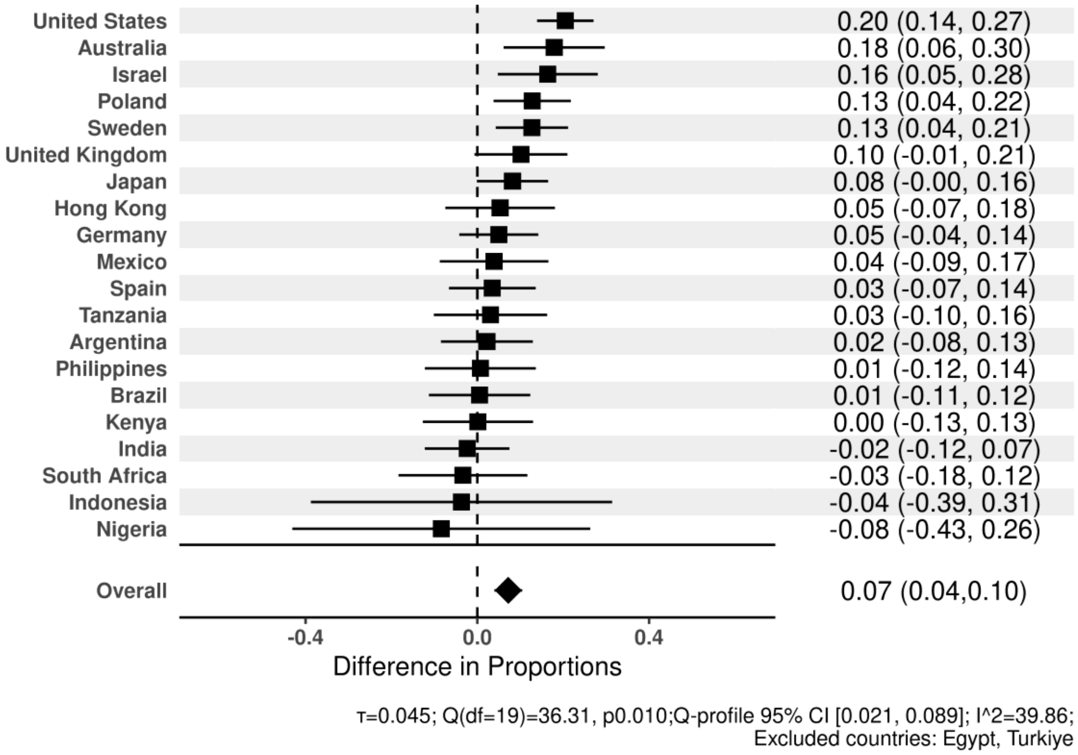

Figure S75. Forest plot for `Marital status`-`(Ref: Divorced) Widowed`

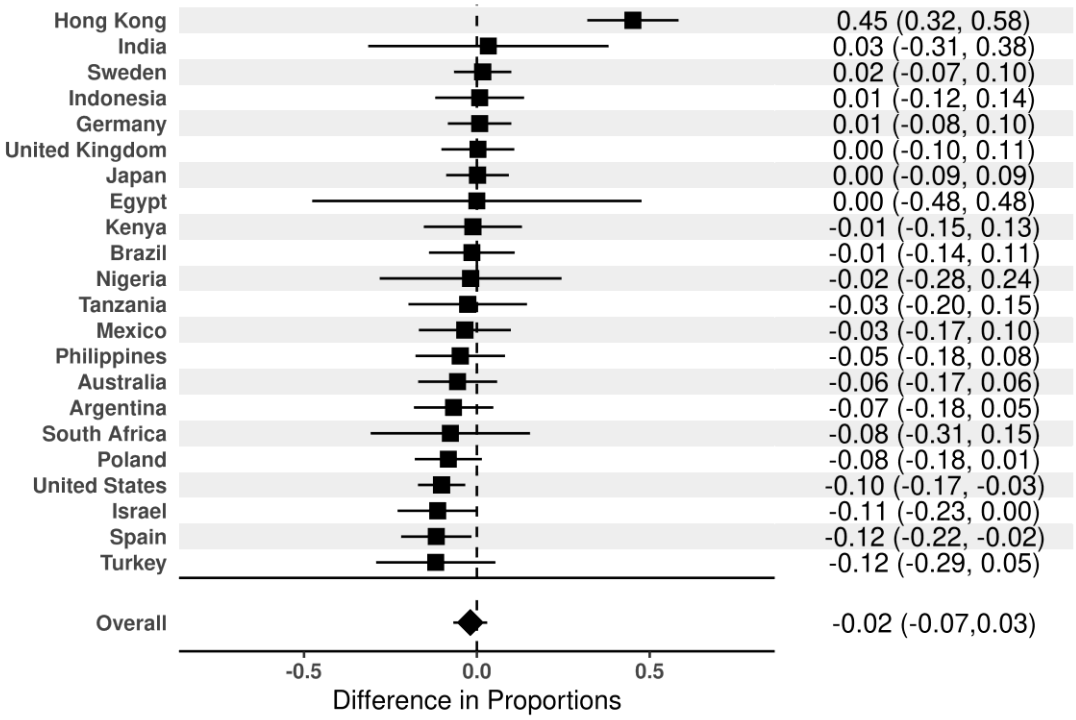

τ=0.093; Q(df=21)=66.45, p<.001;Q-profile 95% CI [0.057, 0.147]; I^2=70.02;

Figure S76. Forest plot for `Marital status`-`(Ref: Divorced) Single, never married`

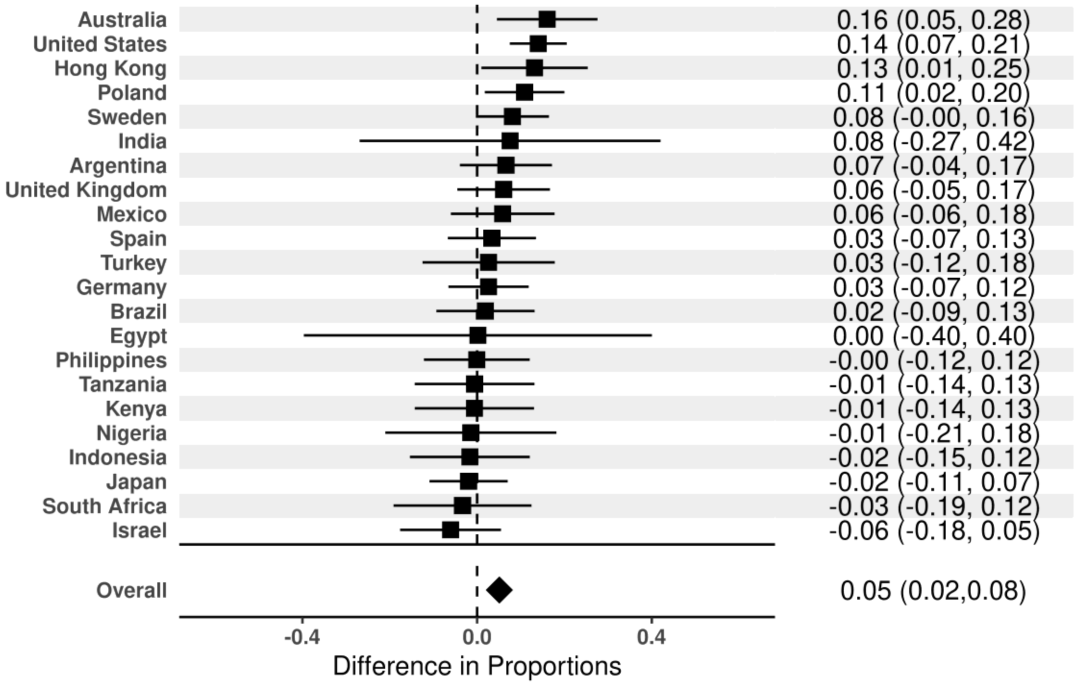

τ=0.022; Q(df=21)=25.75, p0.216;Q-profile 95% CI [0.000, 0.072]; I^2=13.09;

Figure S77. Forest plot for `Marital status`-`(Ref: Divorced) Domestic partner`

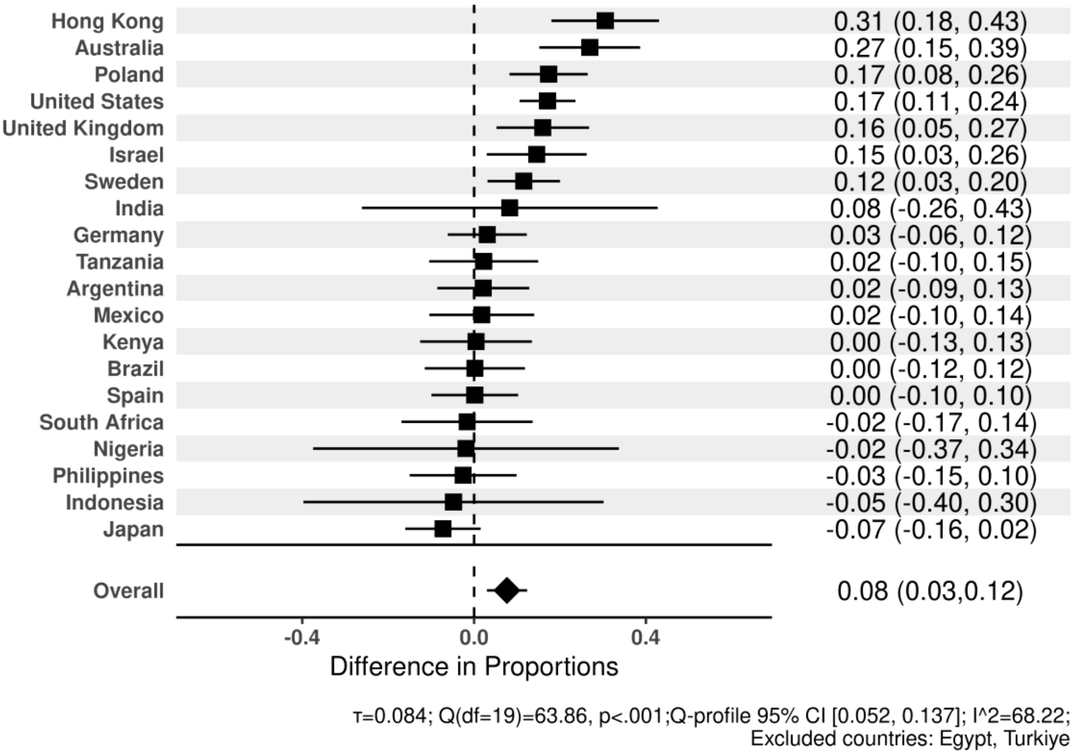

Figure S78. Forest plot for `Marital status`-`(Ref: Widowed) Single, never married`

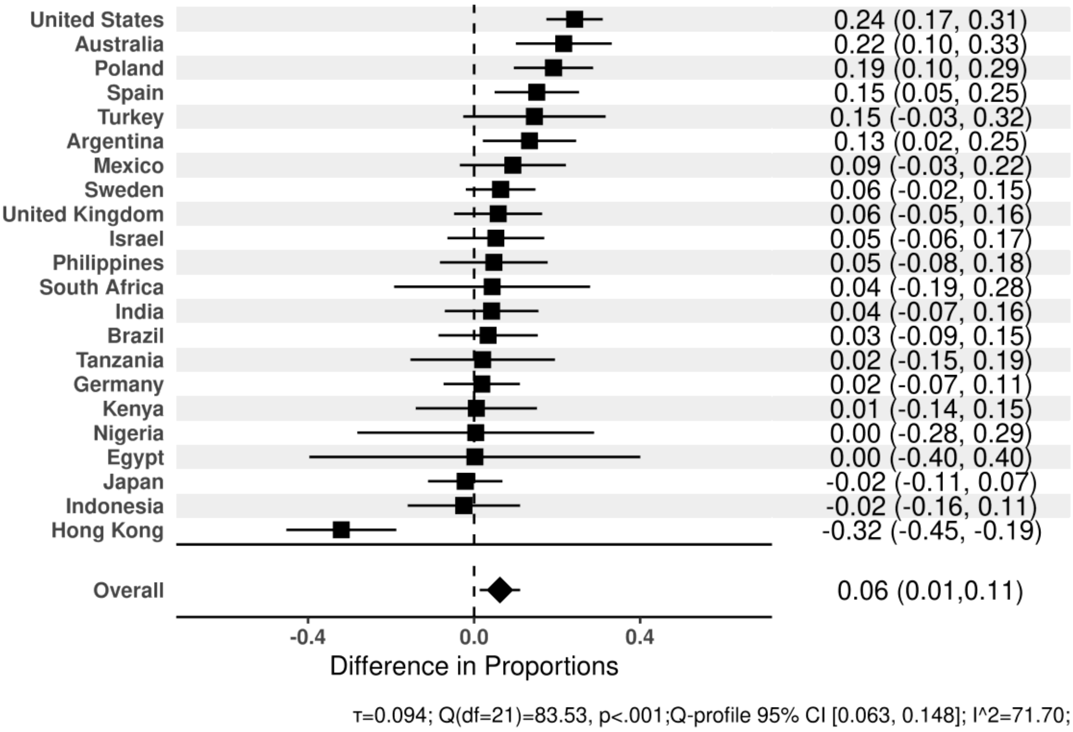

Figure S79. Forest plot for `Marital status`-`(Ref: Widowed) Domestic partner`

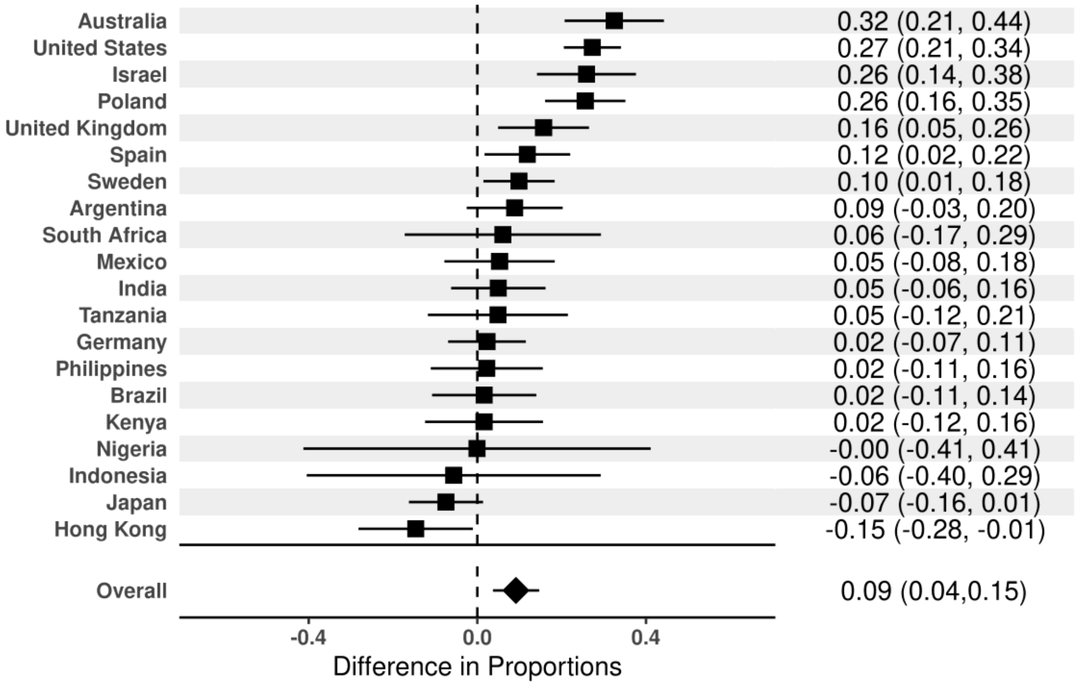

τ=0.104; Q(df=19)=94.44, p<.001;Q-profile 95% CI [0.071, 0.163]; I^2=76.09;
Excluded countries: Egypt, Turkiye

Figure S80. Forest plot for `Marital status`-`(Ref: Single, never married) Domestic partner`

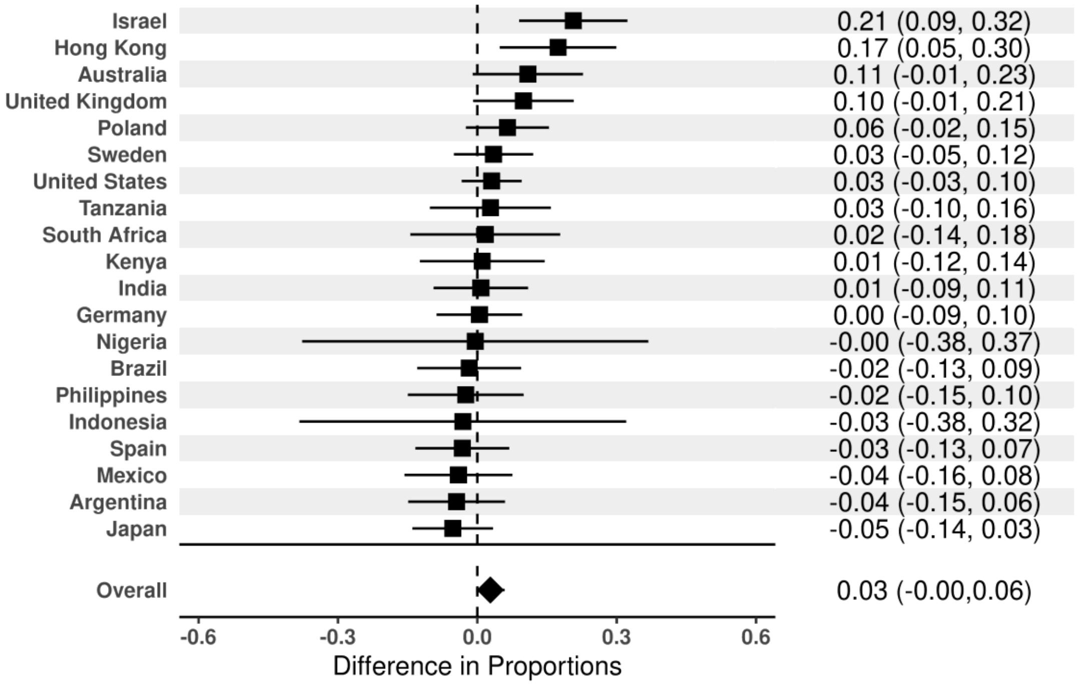

τ=0.040; Q(df=19)=28.14, p0.081;Q-profile 95% CI [0.000, 0.080]; I^2=33.81;
Excluded countries: Egypt, Turkiye

Figure S81. Forest plot for `Employment status`-`(Ref: Employed for an employer) Self-employed`

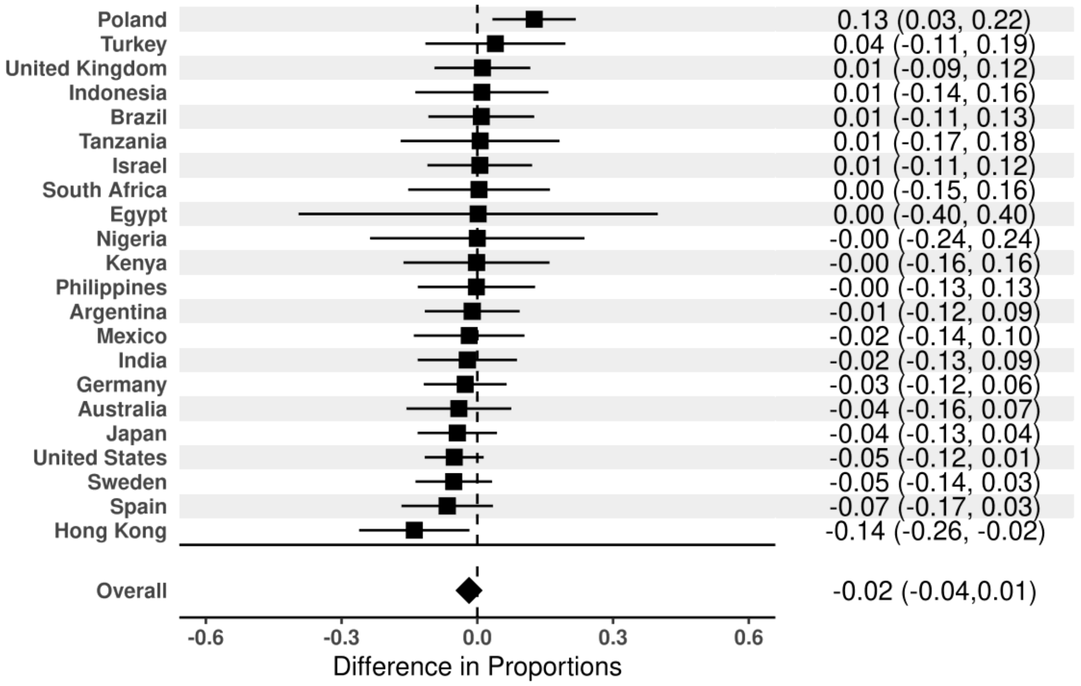

τ=0.000; Q(df=21)=17.79, p0.662;Q-profile 95% CI [0.000, 0.055]; I^2=0.00;

Figure S82. Forest plot for `Employment status`-`(Ref: Employed for an employer) Retired`

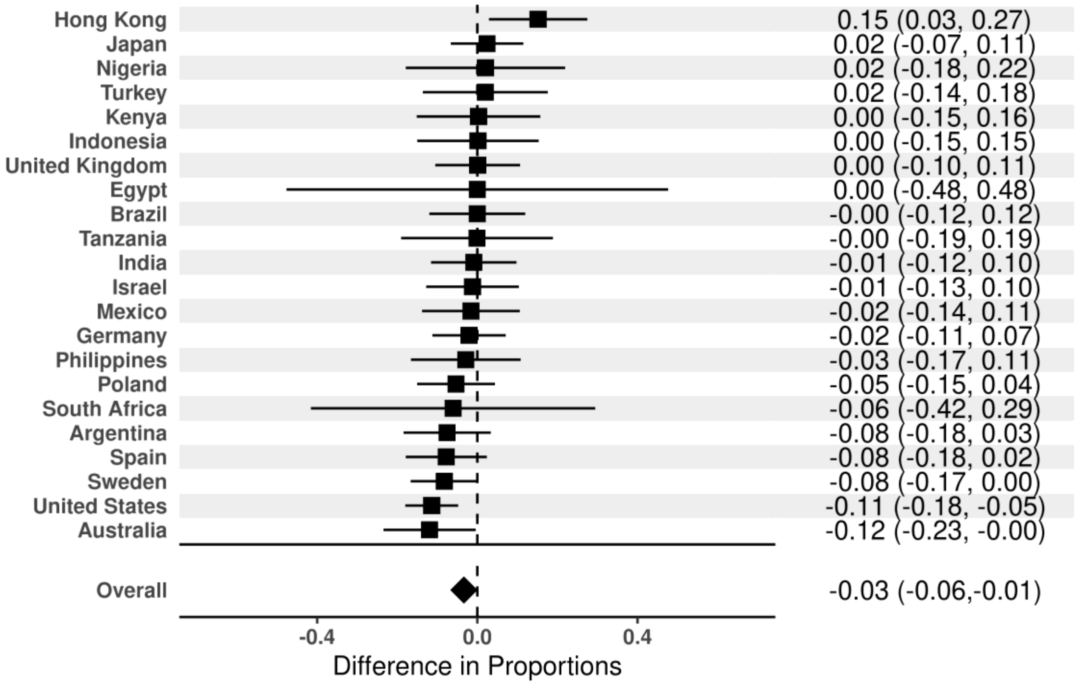

τ=0.017; Q(df=21)=23.38, p0.324;Q-profile 95% CI [0.000, 0.070]; I^2=7.51;

Figure S83. Forest plot for `Employment status`-`(Ref: Employed for an employer) Student`

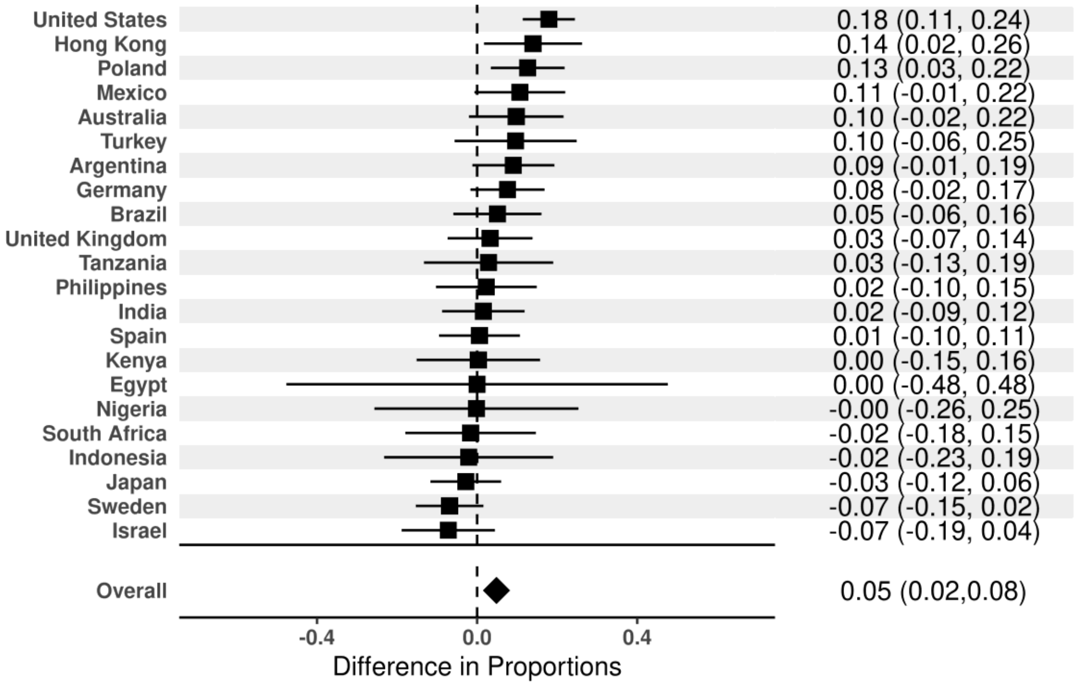

τ=0.043; Q(df=21)=40.52, p0.006;Q-profile 95% CI [0.025, 0.091]; I^2=35.28;

Figure S84. Forest plot for `Employment status`-`(Ref: Employed for an employer) Homemaker`

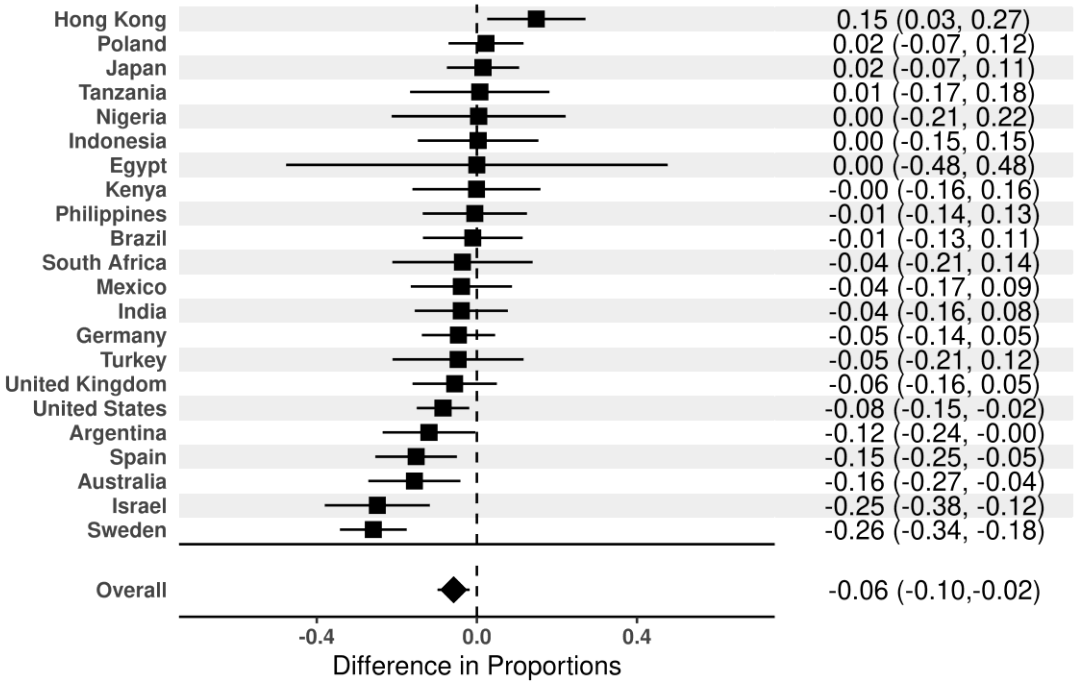

τ=0.071; Q(df=21)=57.47, p<.001;Q-profile 95% CI [0.044, 0.119]; I^2=58.67;

Figure S85. Forest plot for `Employment status`-`(Ref: Employed for an employer) Unemployed and looking for a job`

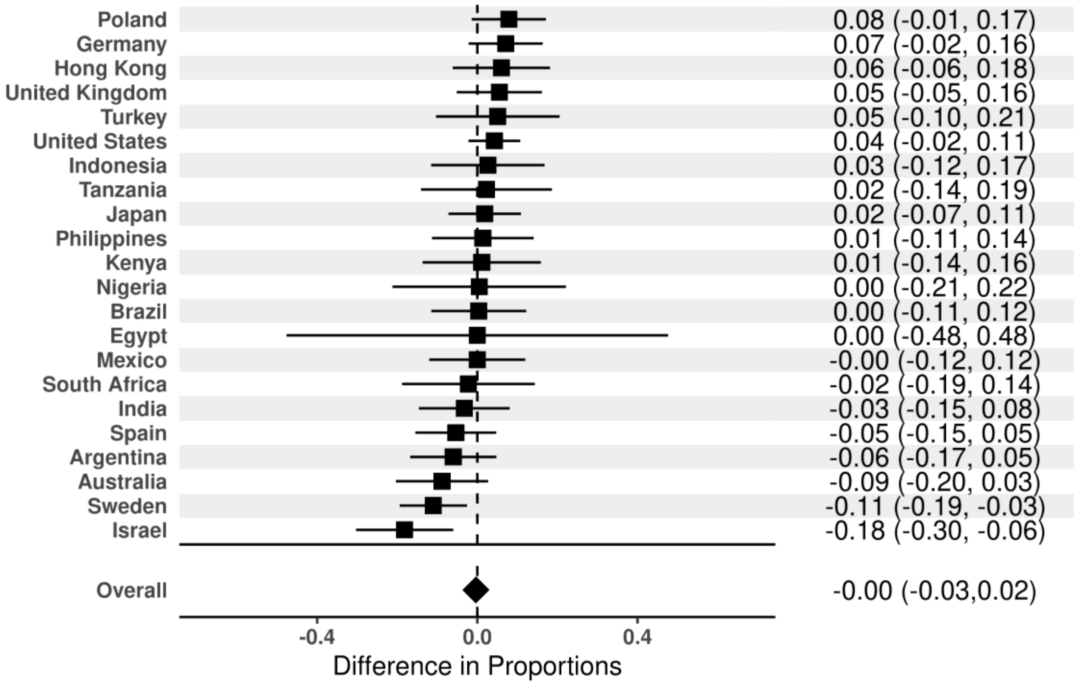

τ=0.033; Q(df=21)=29.56, p0.101;Q-profile 95% CI [0.000, 0.078]; I^2=23.99;

Figure S86. Forest plot for `Employment status`-`(Ref: Employed for an employer) None of these/other`

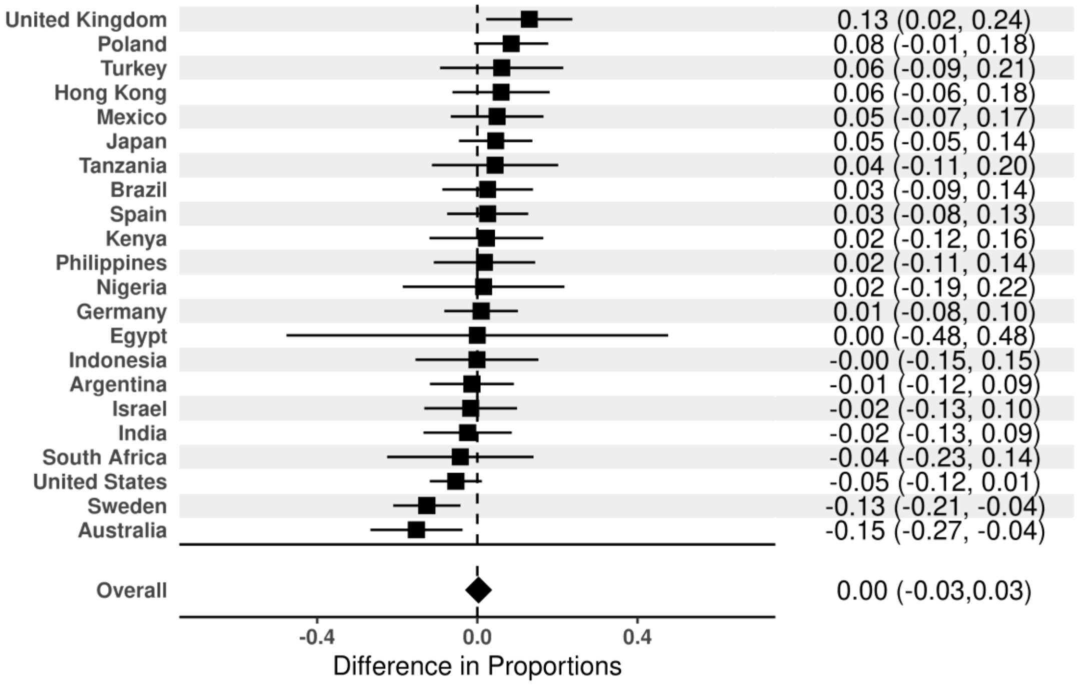

τ=0.035; Q(df=21)=31.49, p0.066;Q-profile 95% CI [0.000, 0.081]; I^2=26.47;

Figure S87. Forest plot for `Employment status`-`(Ref: Self-employed) Retired`

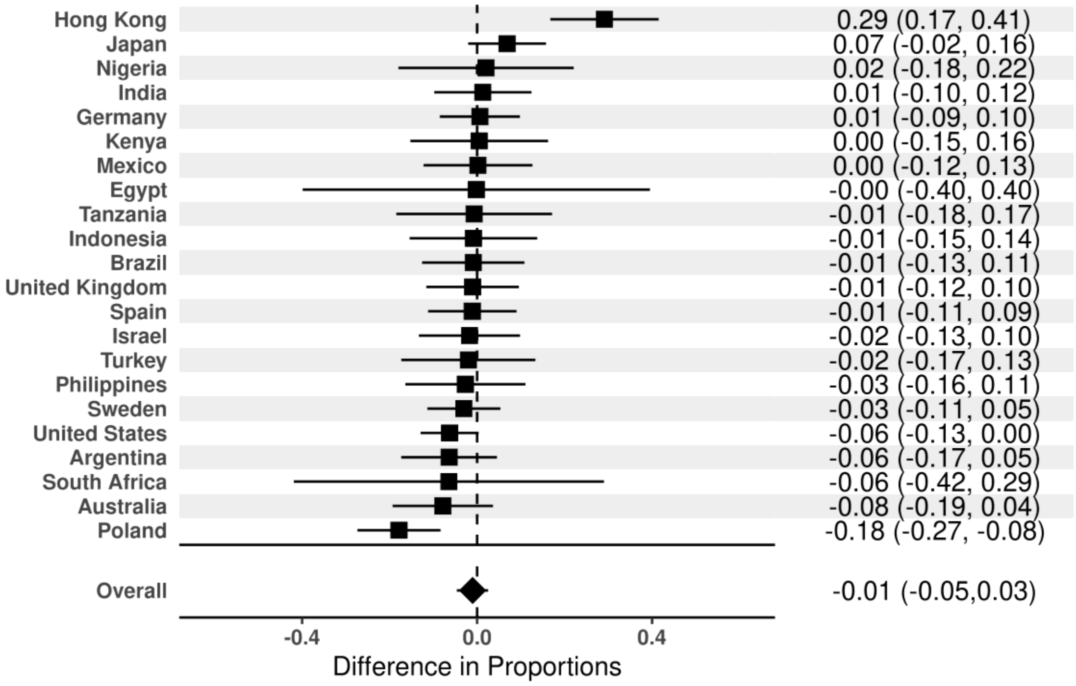

τ=0.057; Q(df=21)=43.25, p0.003;Q-profile 95% CI [0.027, 0.104]; I^2=48.49;

Figure S88. Forest plot for `Employment status`-`(Ref: Self-employed) Student`

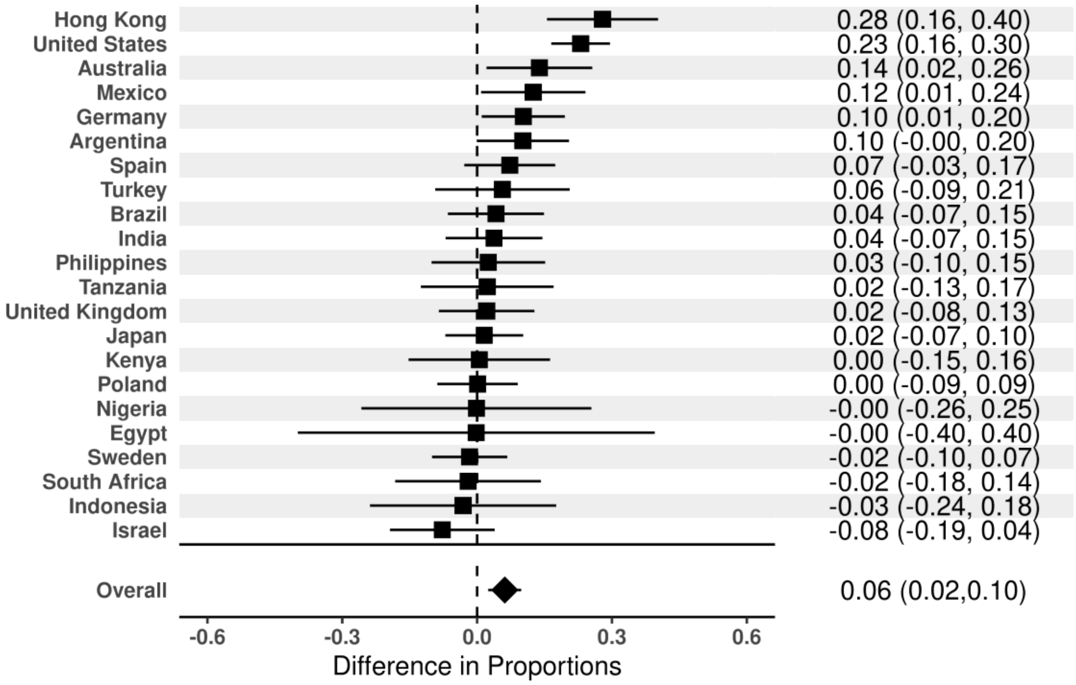

τ=0.061; Q(df=21)=56.66, p<.001;Q-profile 95% CI [0.039, 0.108]; I^2=53.41;

Figure S89. Forest plot for `Employment status`-`(Ref: Self-employed) Homemaker`

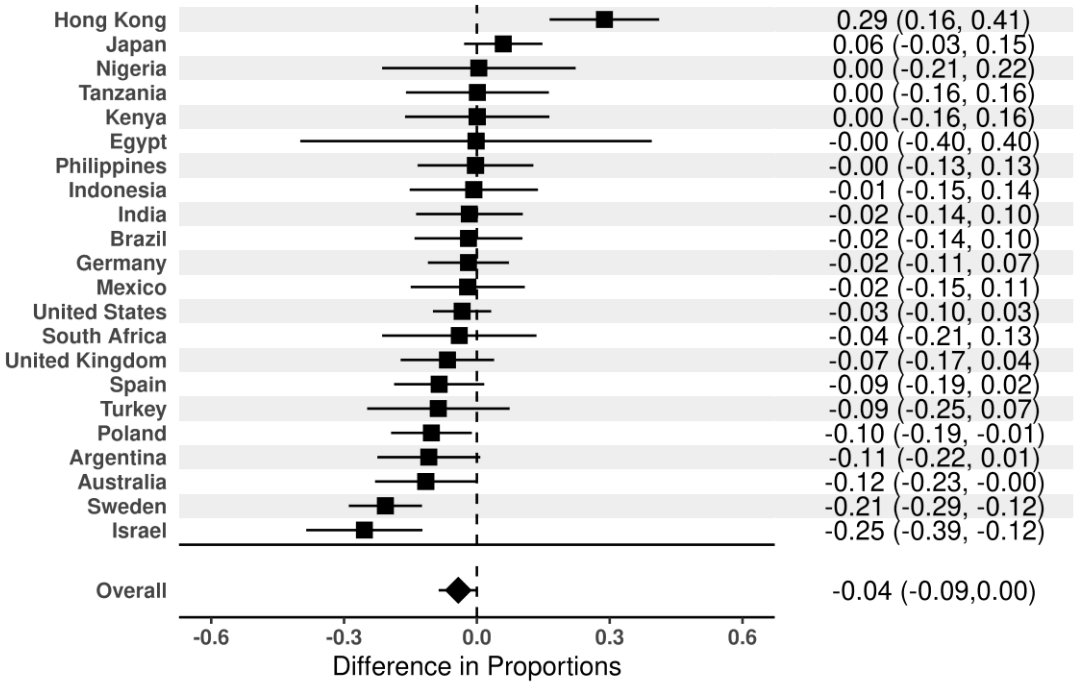

τ=0.083; Q(df=21)=65.25, p<.001;Q-profile 95% CI [0.052, 0.132]; I^2=66.34;

Figure S90. Forest plot for `Employment status`-`(Ref: Self-employed) Unemployed and looking for a job`

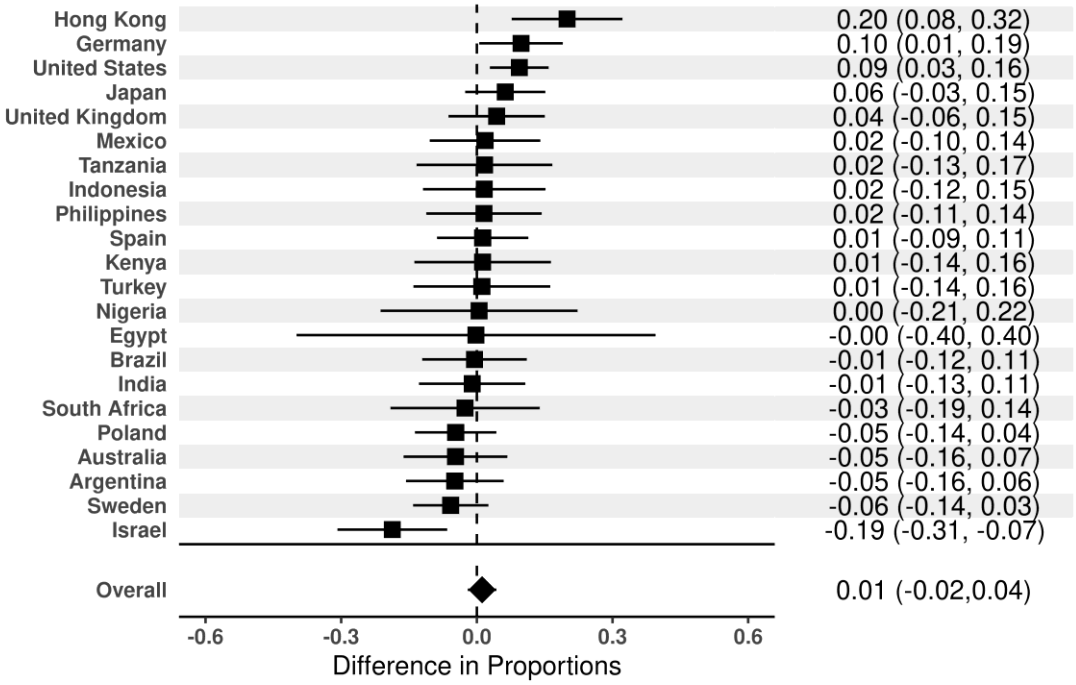

τ=0.046; Q(df=21)=37.24, p0.016;Q-profile 95% CI [0.017, 0.089]; I^2=38.52;

Figure S91. Forest plot for `Employment status`-`(Ref: Self-employed) None of these/other`

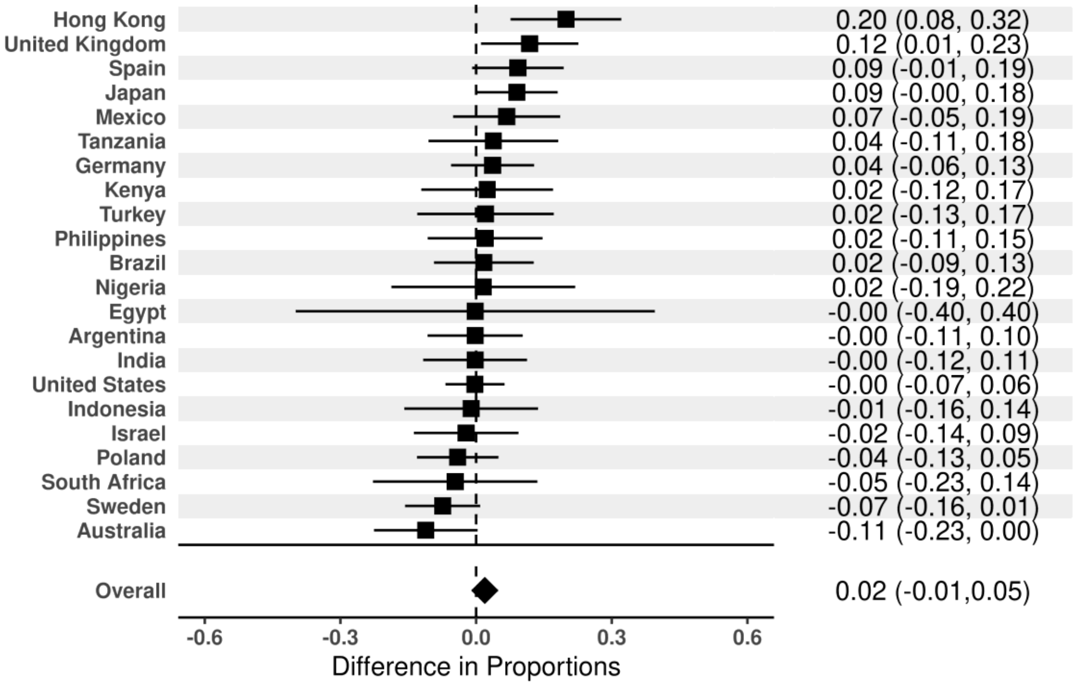

τ=0.035; Q(df=21)=30.12, p0.090;Q-profile 95% CI [0.000, 0.078]; I^2=27.20;

Figure S92. Forest plot for `Employment status`-`(Ref: Retired) Student`

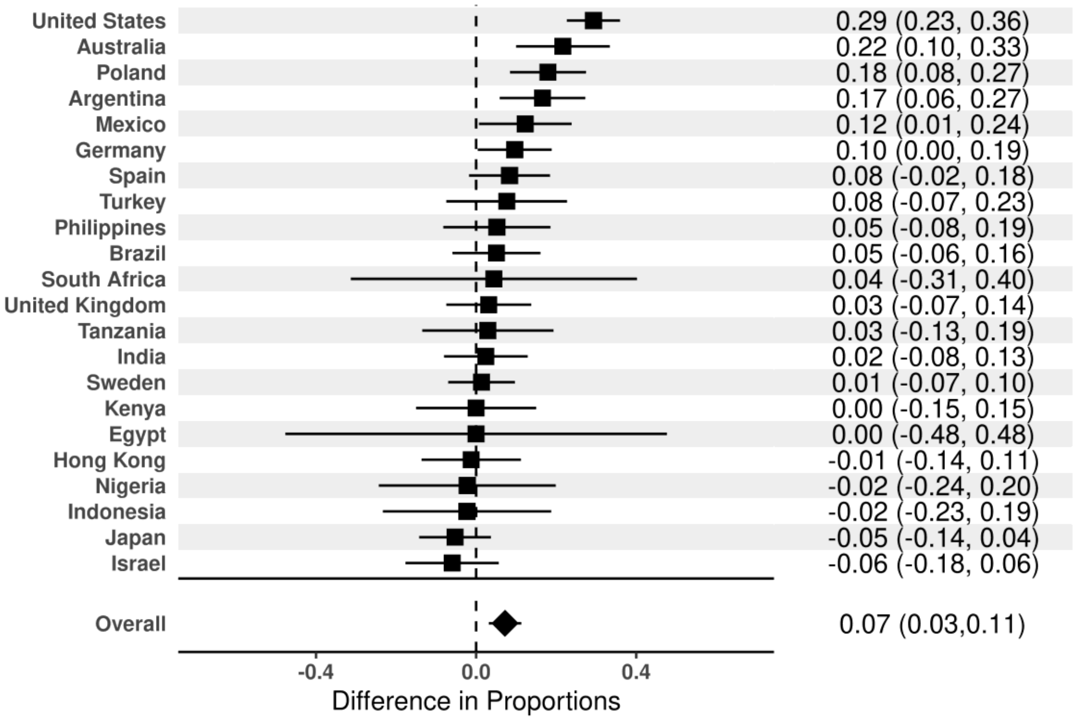

τ=0.071; Q(df=21)=75.89, p<.001;Q-profile 95% CI [0.051, 0.125]; I^2=59.71;

Figure S93. Forest plot for `Employment status`-`(Ref: Retired) Homemaker`

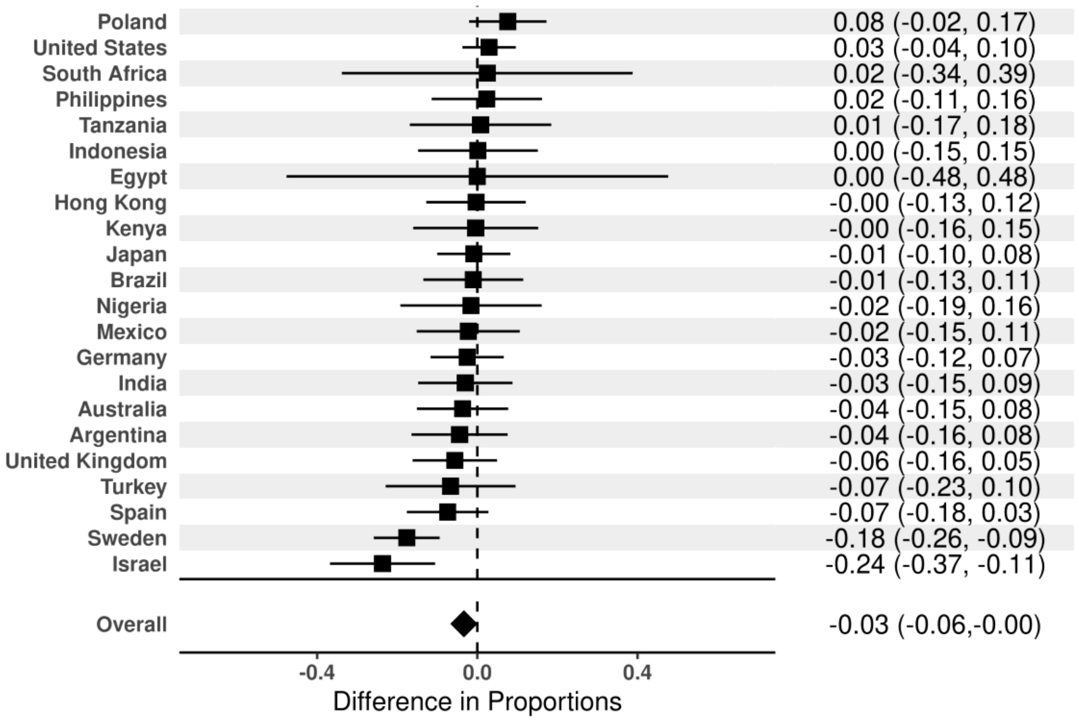

τ=0.036; Q(df=21)=32.21, p0.056;Q-profile 95% CI [0.011, 0.086]; I^2=26.08;

Figure S94. Forest plot for `Employment status`-`(Ref: Retired) Unemployed and looking for a job`

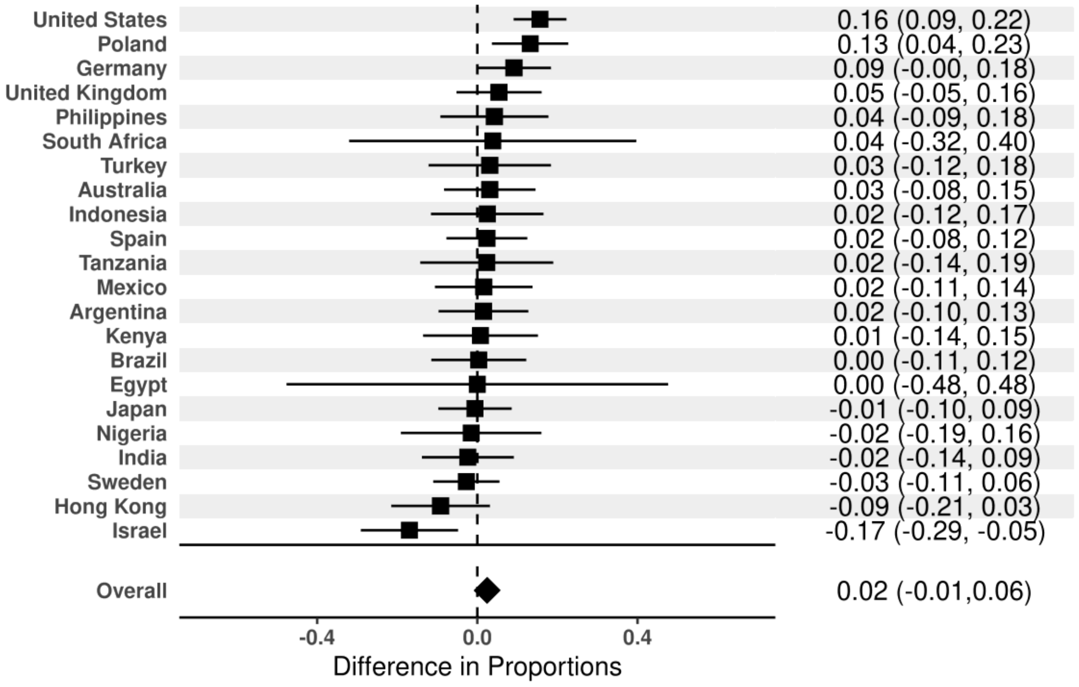

τ=0.042; Q(df=21)=38.52, p0.011;Q-profile 95% CI [0.022, 0.092]; I^2=33.69;

Figure S95. Forest plot for `Employment status`-`(Ref: Retired) None of these/other`

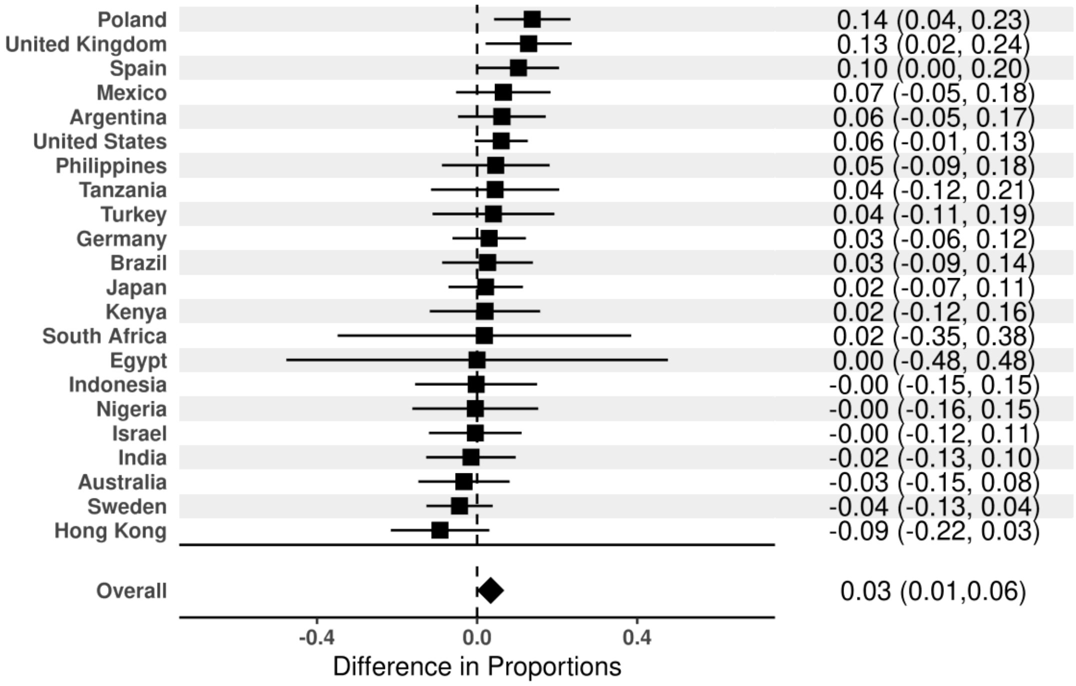

τ=0.000; Q(df=21)=21.10, p0.453;Q-profile 95% CI [0.000, 0.064]; I^2=0.00;

Figure S96. Forest plot for `Employment status`-`(Ref: Student) Homemaker`

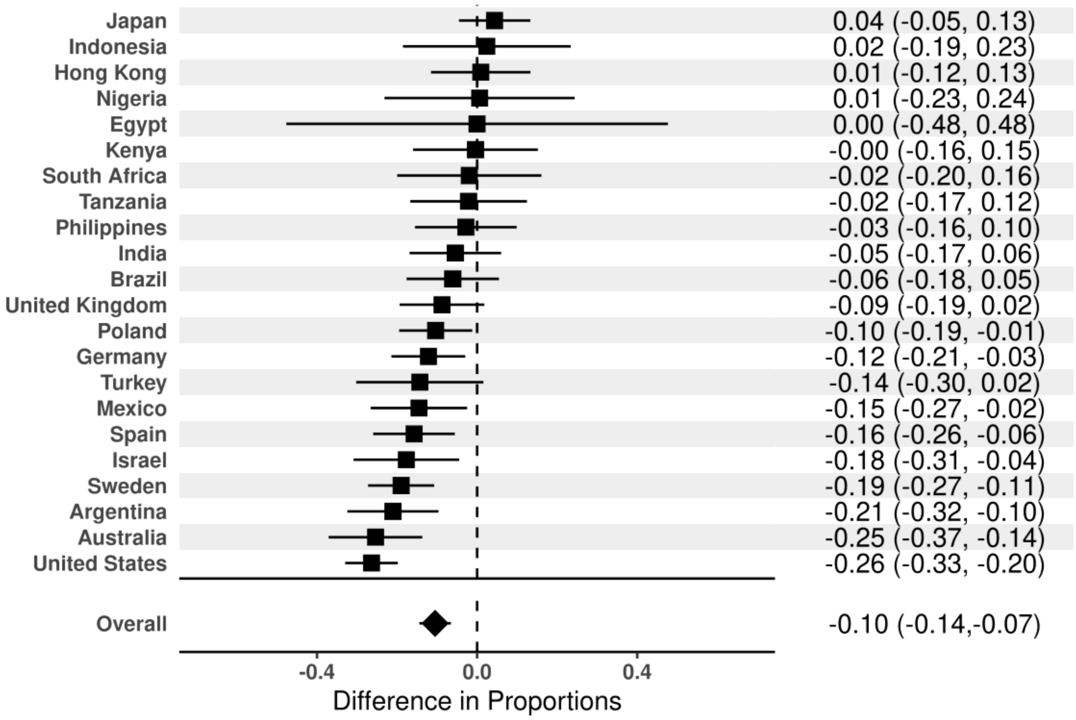

τ=0.068; Q(df=21)=60.14, p<.001;Q-profile 95% CI [0.043, 0.116]; I^2=57.34;

Figure S97. Forest plot for `Employment status`-`(Ref: Student) Unemployed and looking for a job`

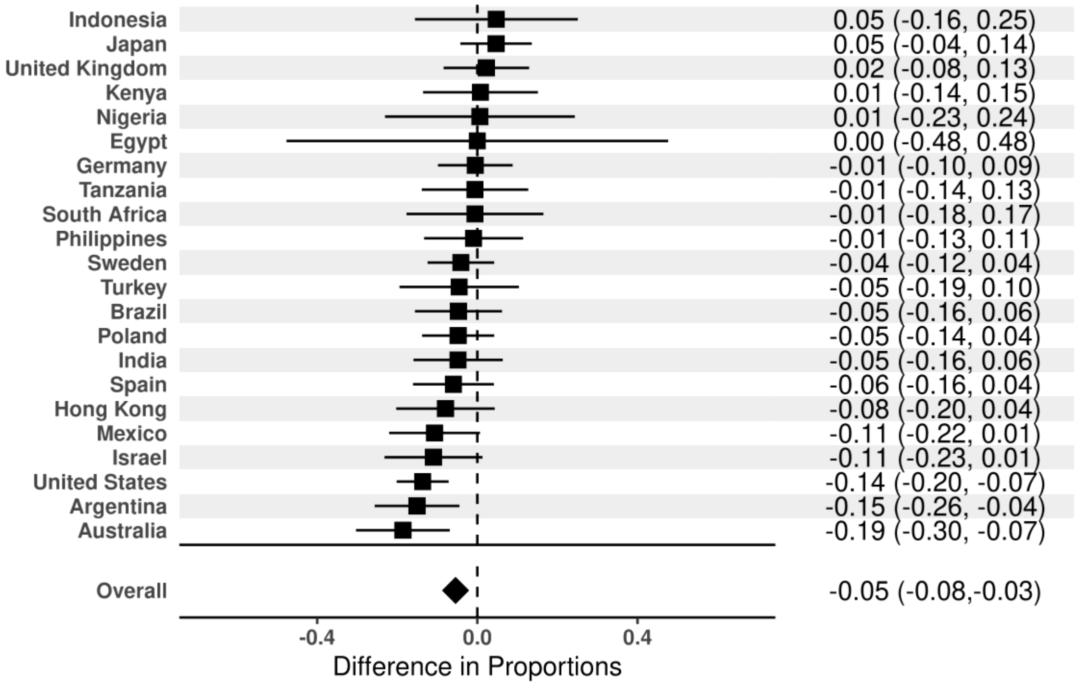

τ=0.026; Q(df=21)=27.42, p0.157;Q-profile 95% CI [0.000, 0.074]; I^2=17.40;

Figure S98. Forest plot for `Employment status`-`(Ref: Student) None of these/other`

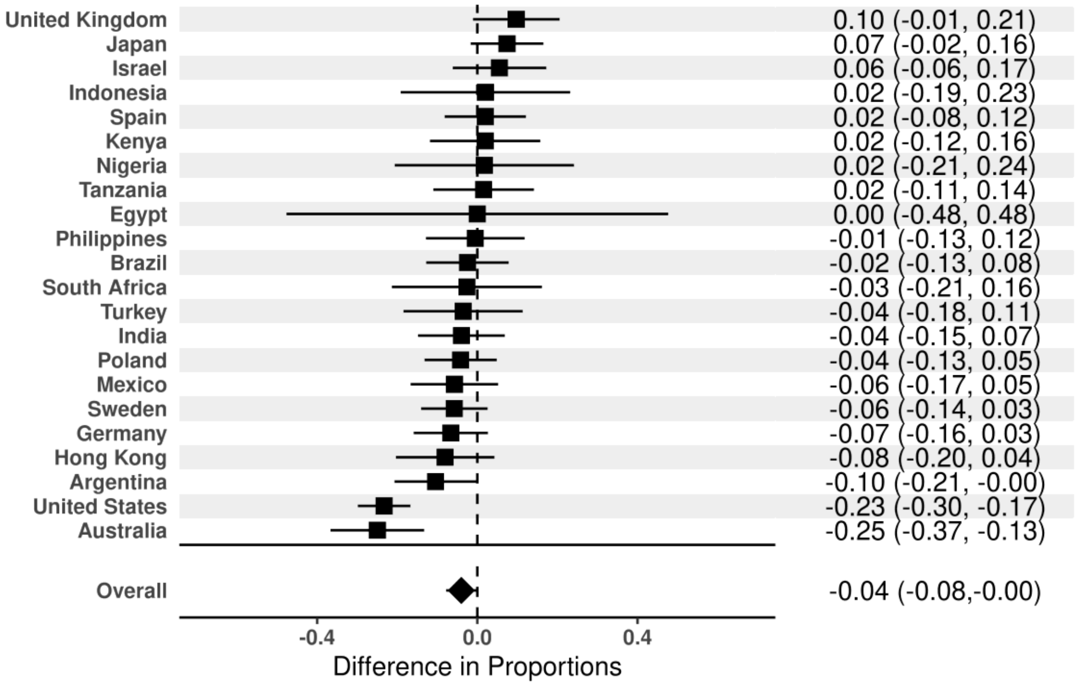

τ=0.066; Q(df=21)=65.61, p<.001;Q-profile 95% CI [0.045, 0.114]; I^2=57.20;

Figure S99. Forest plot for `Employment status`-`(Ref: Homemaker) Unemployed and looking for a job`

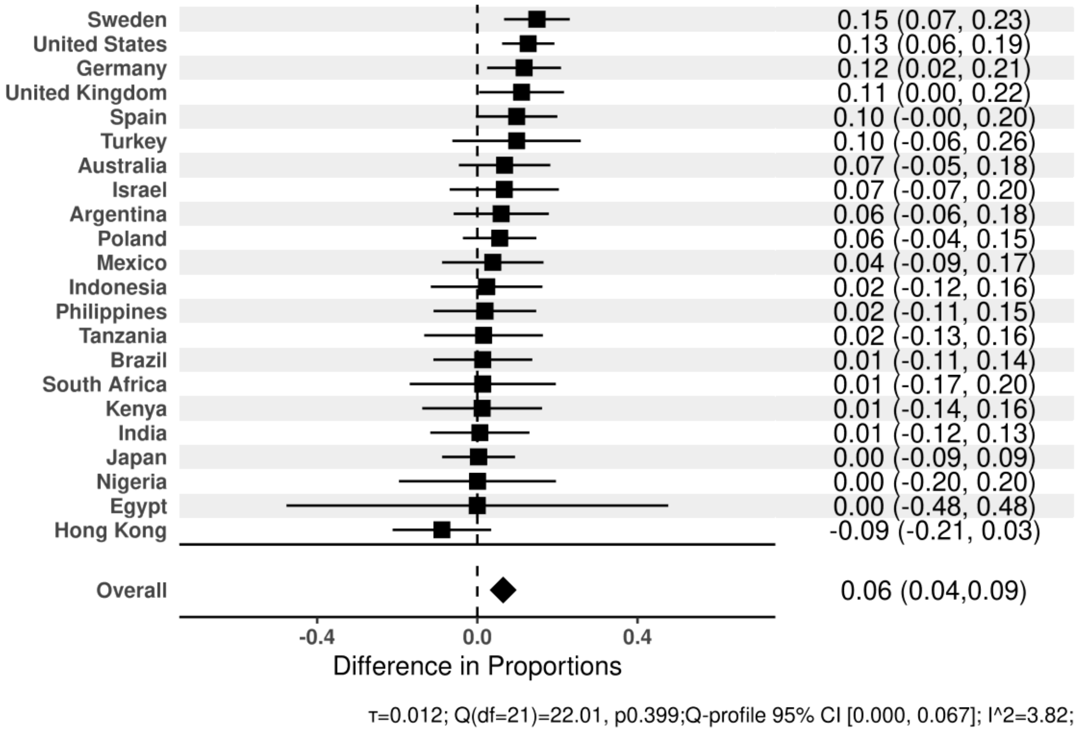

Figure S100. Forest plot for `Employment status`-`(Ref: Homemaker) None of these/other`

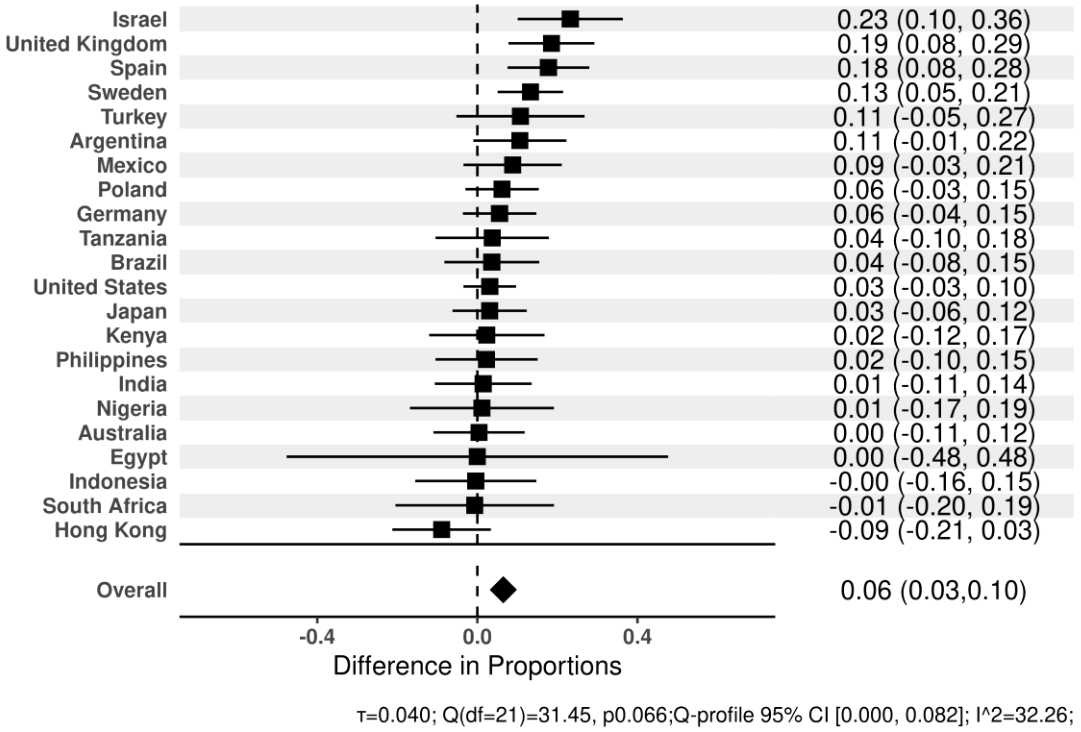

Figure S101. Forest plot for `Employment status`-`(Ref: Unemployed and looking for a job) None of these/other`

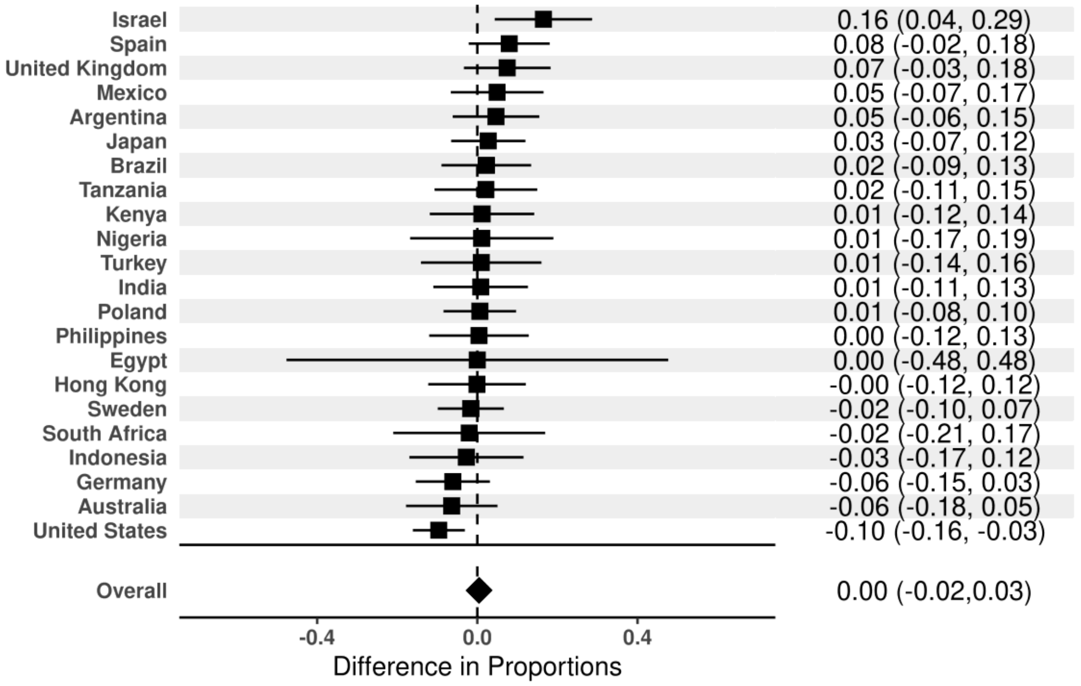

τ=0.019; Q(df=21)=25.01, p0.247;Q-profile 95% CI [0.000, 0.071]; I^2=10.35;

Figure S102. Forest plot for `Religious service attendance`-`(Ref: >1/week) 1/week`

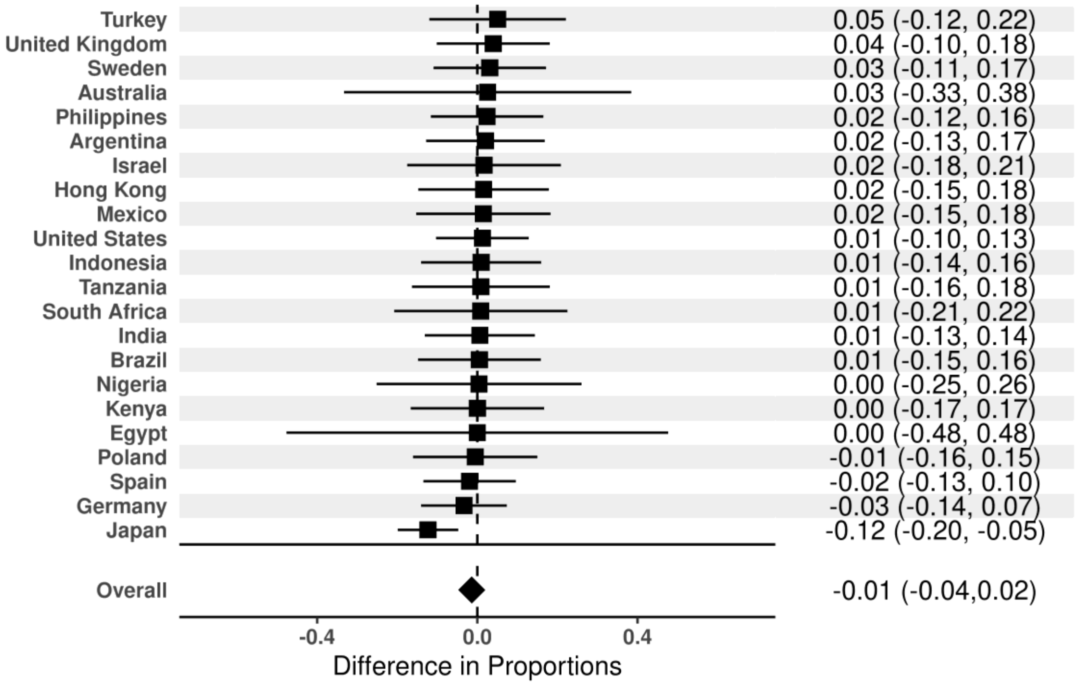

τ=0.000; Q(df=21)=11.16, p0.960;Q-profile 95% CI [0.000, 0.064]; I^2=0.00;

Figure S103. Forest plot for `Religious service attendance`-`(Ref: >1/week) 1-3/month`

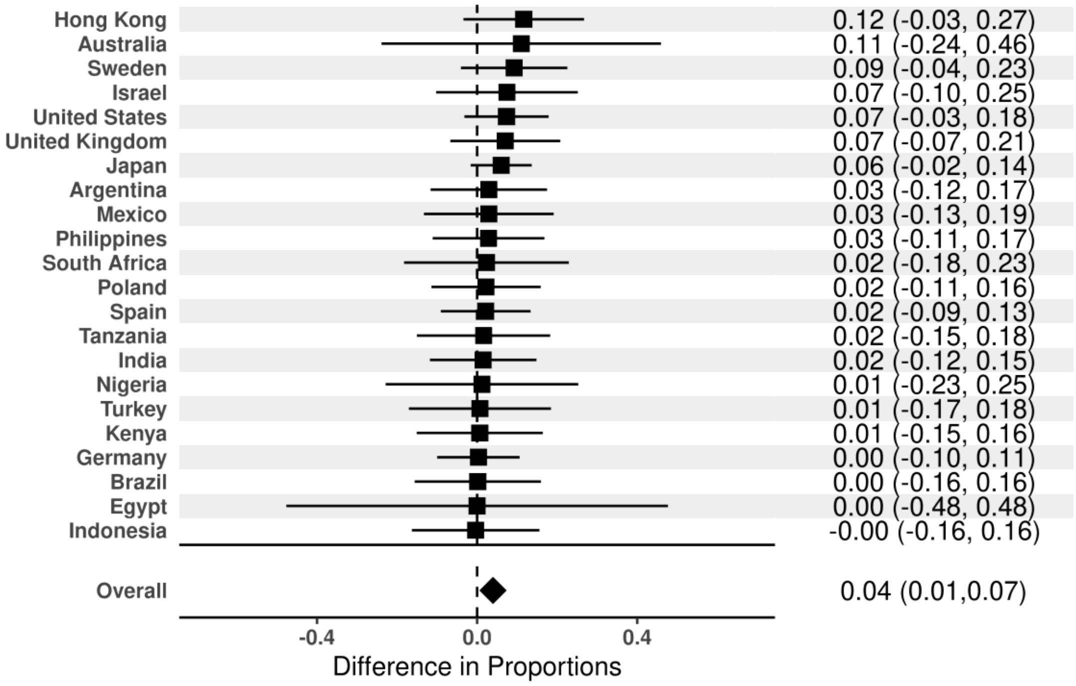

τ=0.000; Q(df=21)=4.60, p1.000;Q-profile 95% CI [0.000, 0.037]; I^2=0.00;

Figure S104. Forest plot for `Religious service attendance`-`(Ref: >1/week) A few times a year`

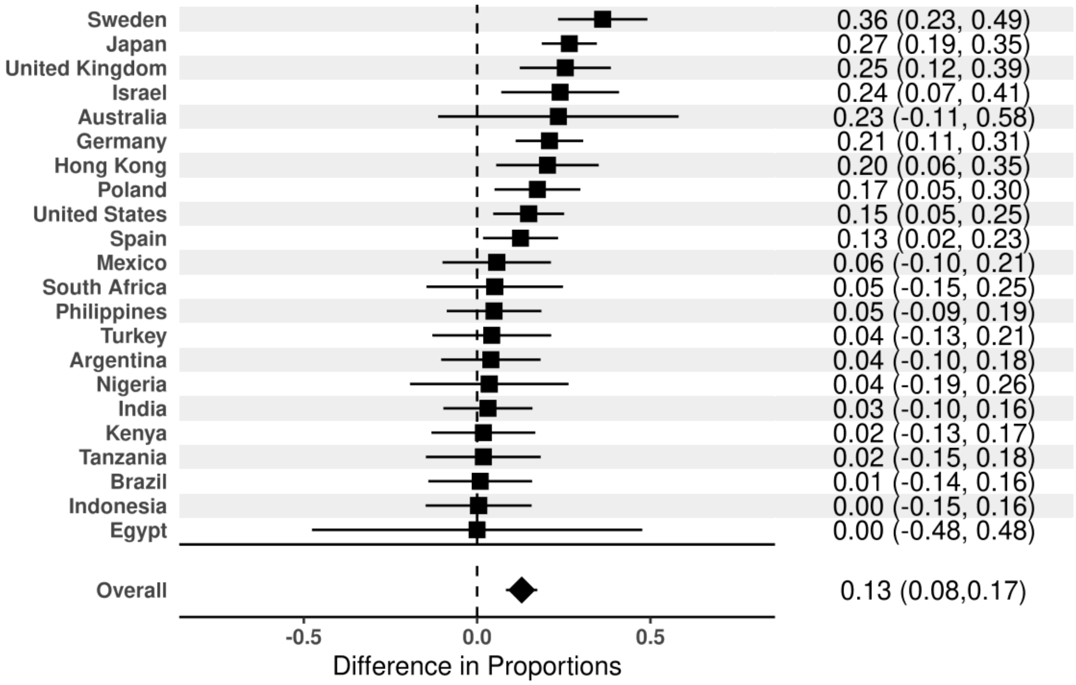

τ=0.076; Q(df=21)=49.65, p<.001;Q-profile 95% CI [0.042, 0.131]; I^2=53.91;

Figure S105. Forest plot for `Religious service attendance`-`(Ref: >1/week) Never`

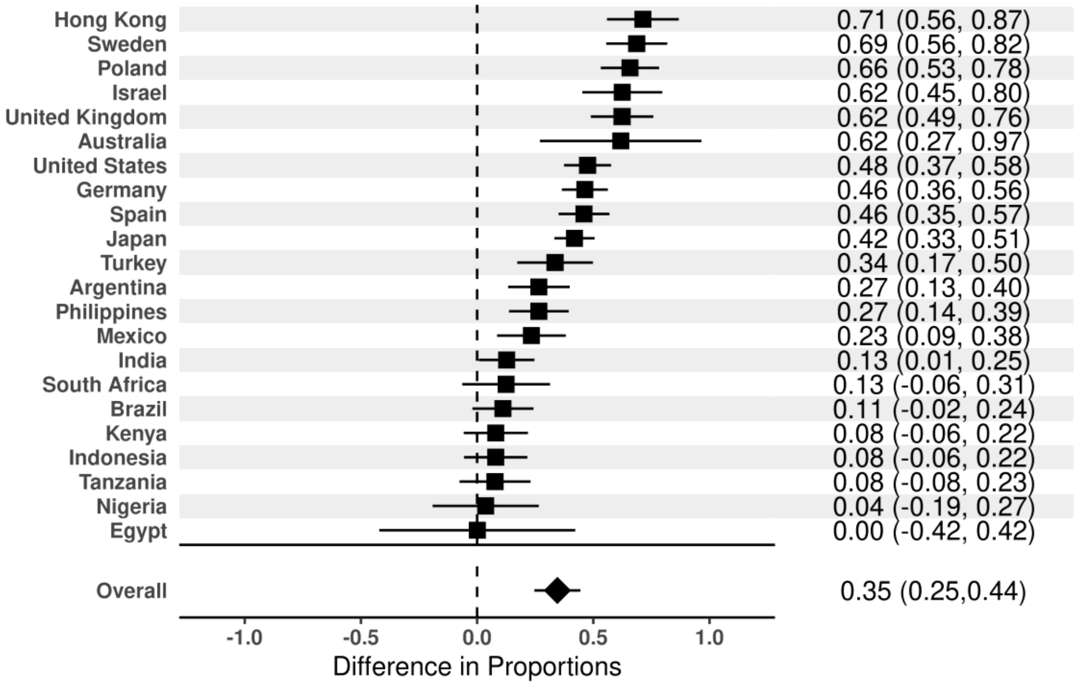

τ=0.222; Q(df=21)=201.98, p<.001;Q-profile 95% CI [0.154, 0.310]; I^2=91.10;

Figure S106. Forest plot for `Religious service attendance`-`(Ref: 1/week) 1-3/month`

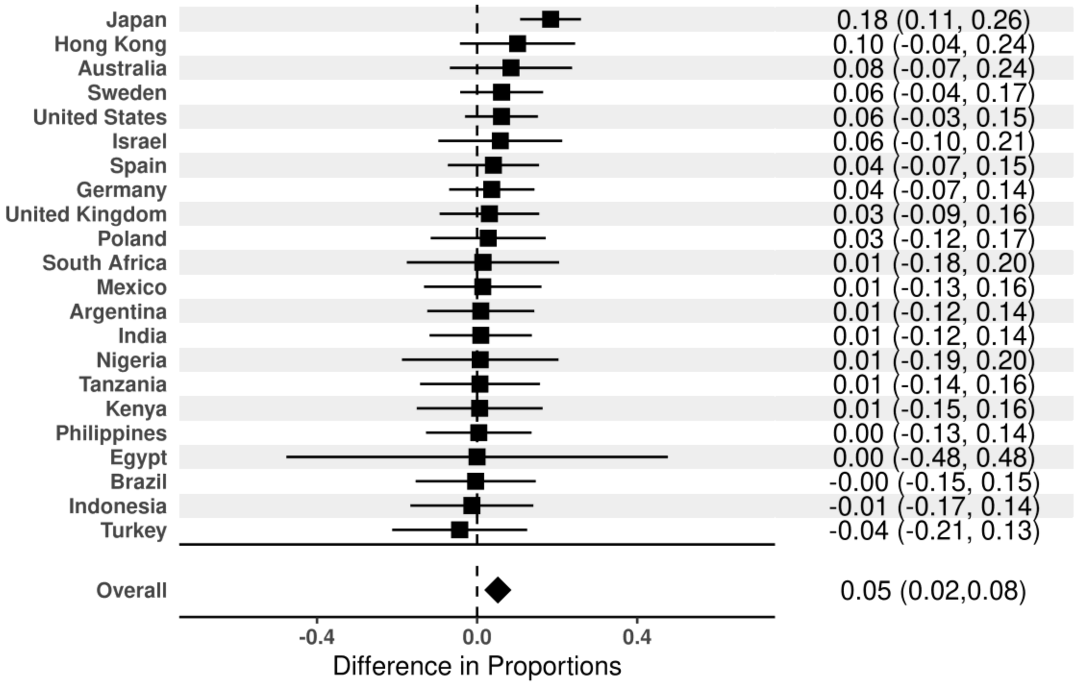

τ=0.000; Q(df=21)=17.62, p0.673;Q-profile 95% CI [0.000, 0.072]; I^2=0.00;

Figure S107. Forest plot for `Religious service attendance`-`(Ref: 1/week) A few times a year`

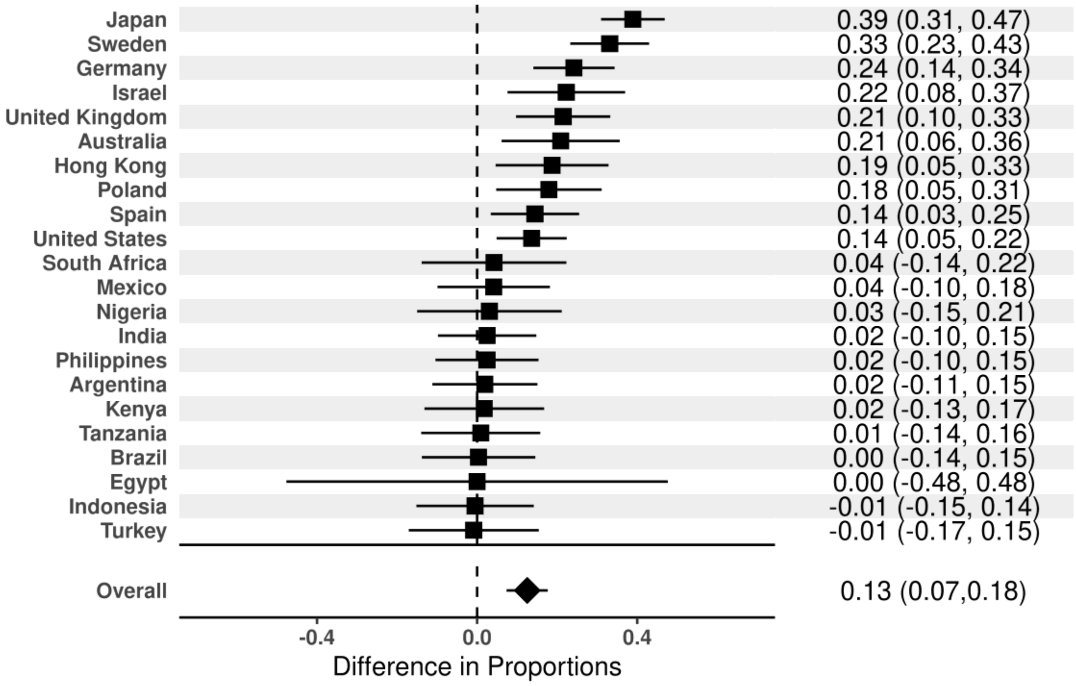

τ=0.100; Q(df=21)=89.70, p<.001;Q-profile 95% CI [0.069, 0.156]; I^2=70.30;

Figure S108. Forest plot for `Religious service attendance`-`(Ref: 1/week) Never`

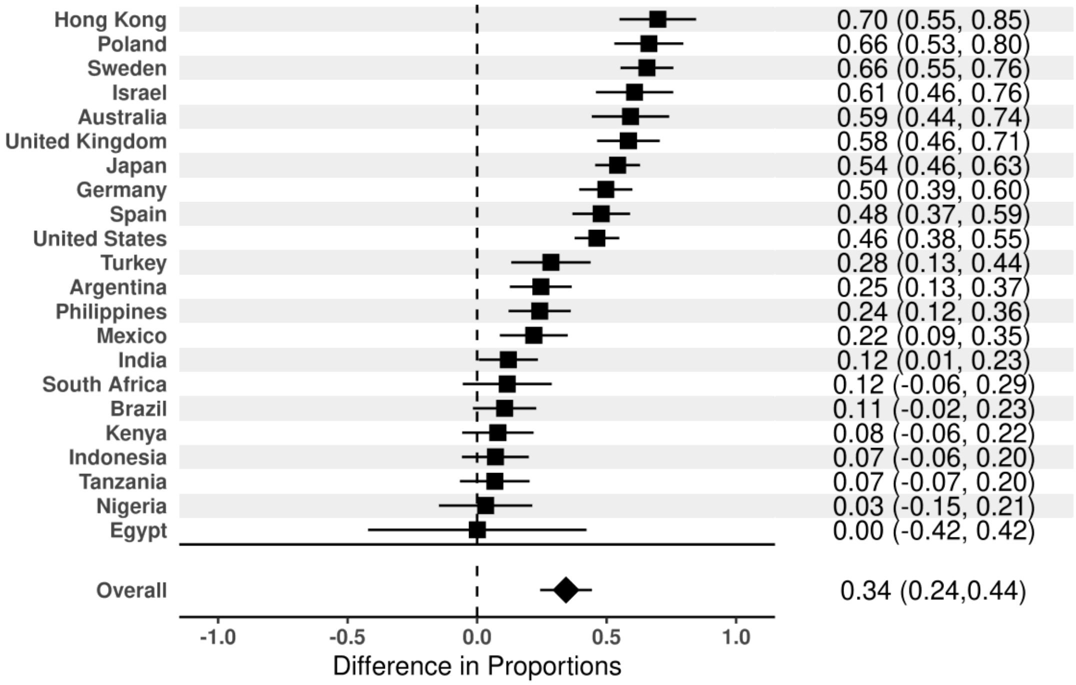

τ=0.228; Q(df=21)=259.59, p<.001;Q-profile 95% CI [0.161, 0.316]; I^2=92.73;

Figure S109. Forest plot for `Religious service attendance`-`(Ref: 1-3/month) A few times a year`

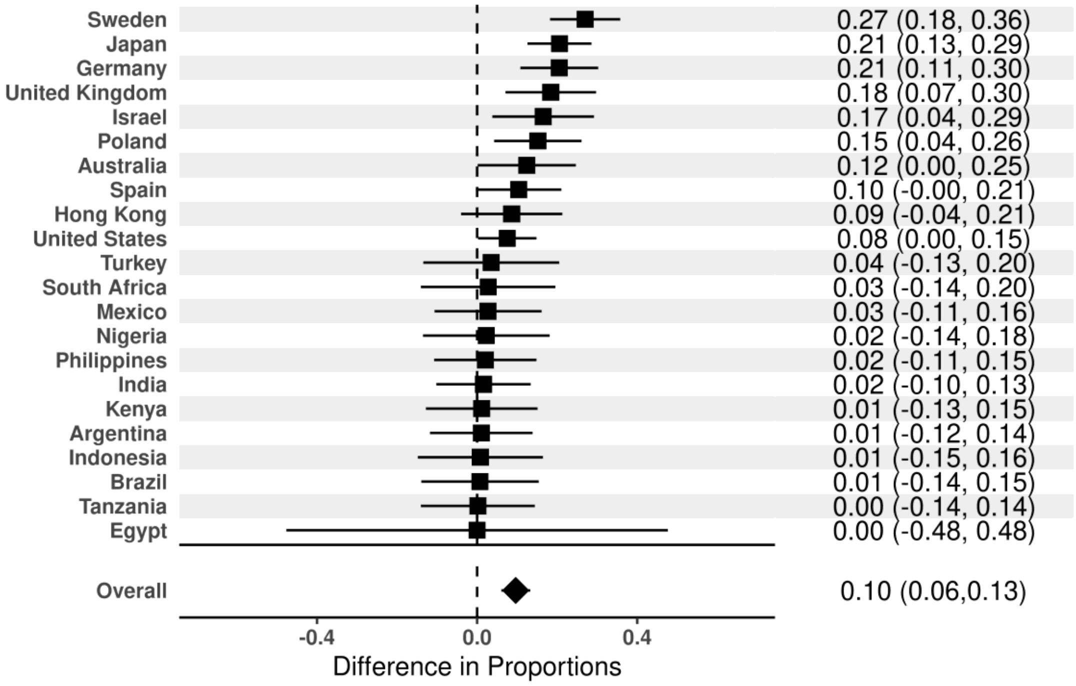

τ=0.058; Q(df=21)=44.80, p0.002;Q-profile 95% CI [0.031, 0.104]; I^2=47.40;

Figure S110. Forest plot for `Religious service attendance`-`(Ref: 1-3/month) Never`

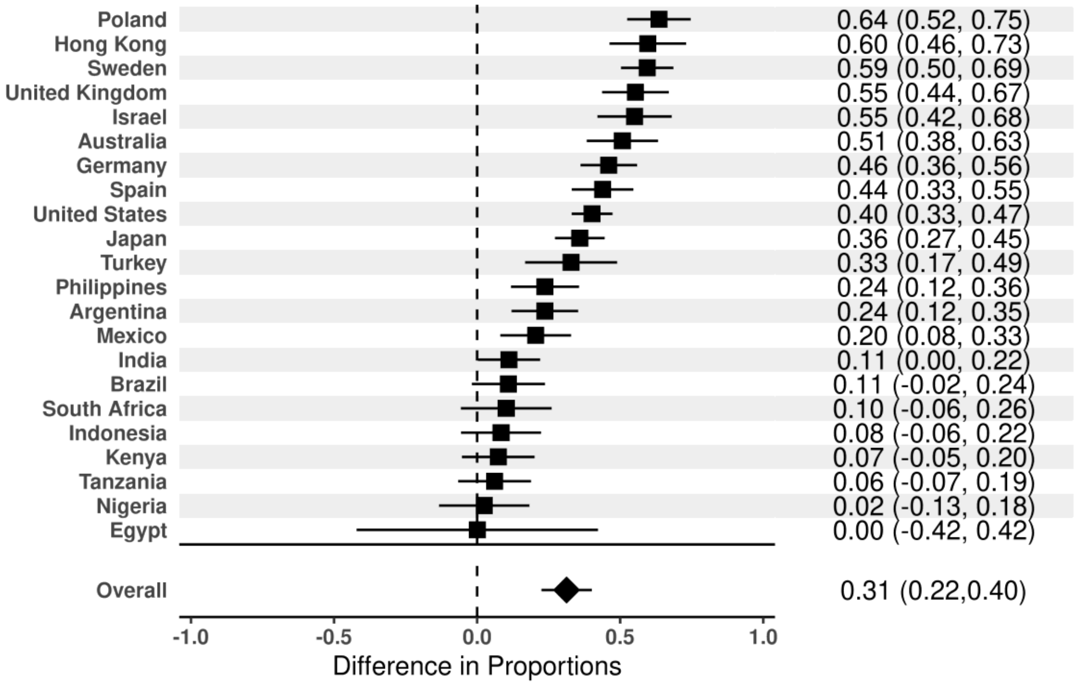

τ=0.199; Q(df=21)=227.01, p<.001;Q-profile 95% CI [0.141, 0.277]; I^2=91.74;

Figure S111. Forest plot for `Religious service attendance`-`(Ref: A few times a year) Never`

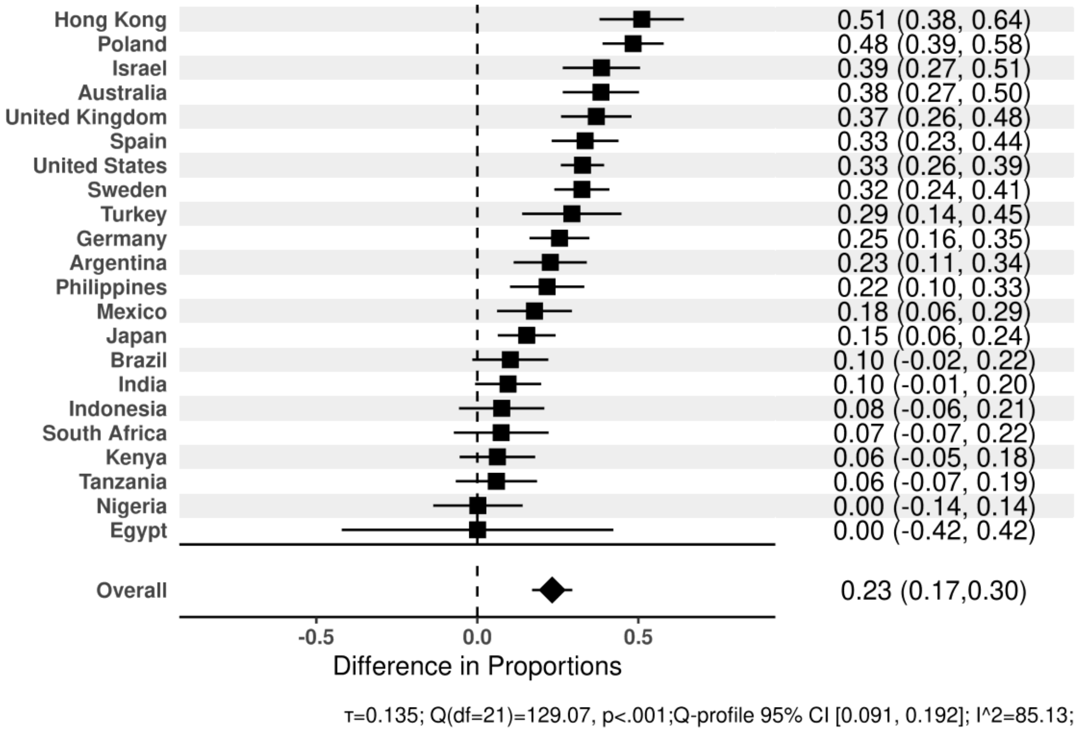

Figure S112. Forest plot for `Education`-`(Ref: Up to 8 years) 9-15 years`

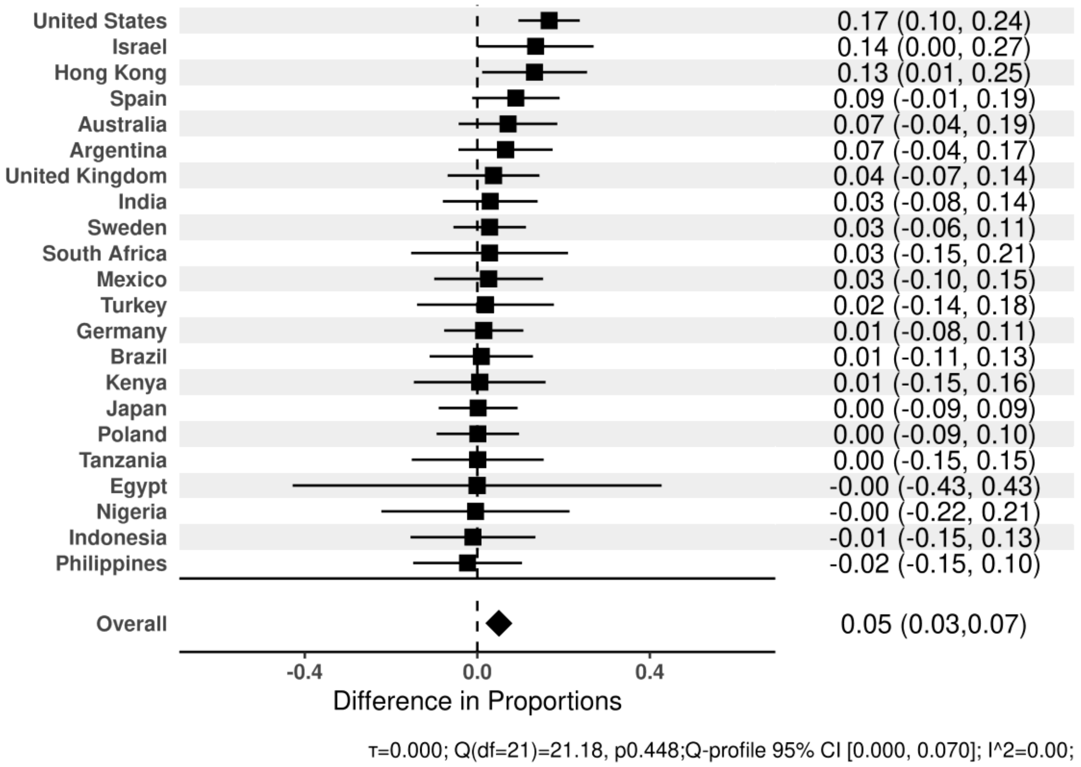

Figure S113. Forest plot for `Education`-`(Ref: Up to 8 years) 16+ years`

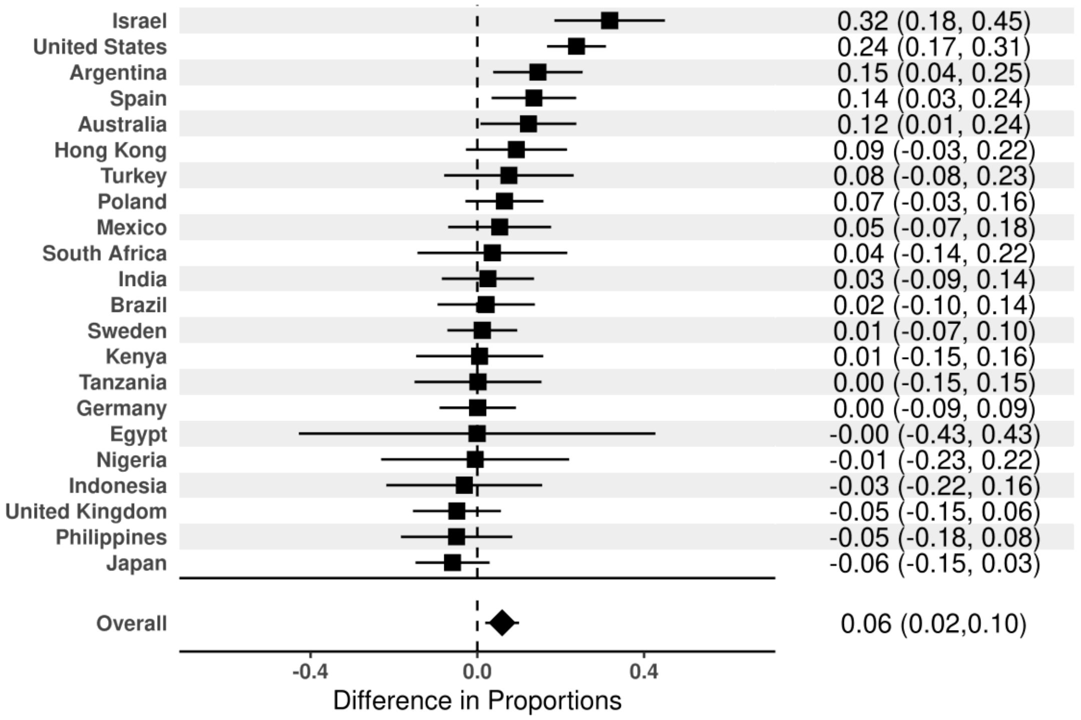

τ=0.072; Q(df=21)=64.23, p<.001;Q-profile 95% CI [0.047, 0.121]; I^2=60.05;

Figure S114. Forest plot for `Education`-`(Ref: 9-15 years) 16+ years`

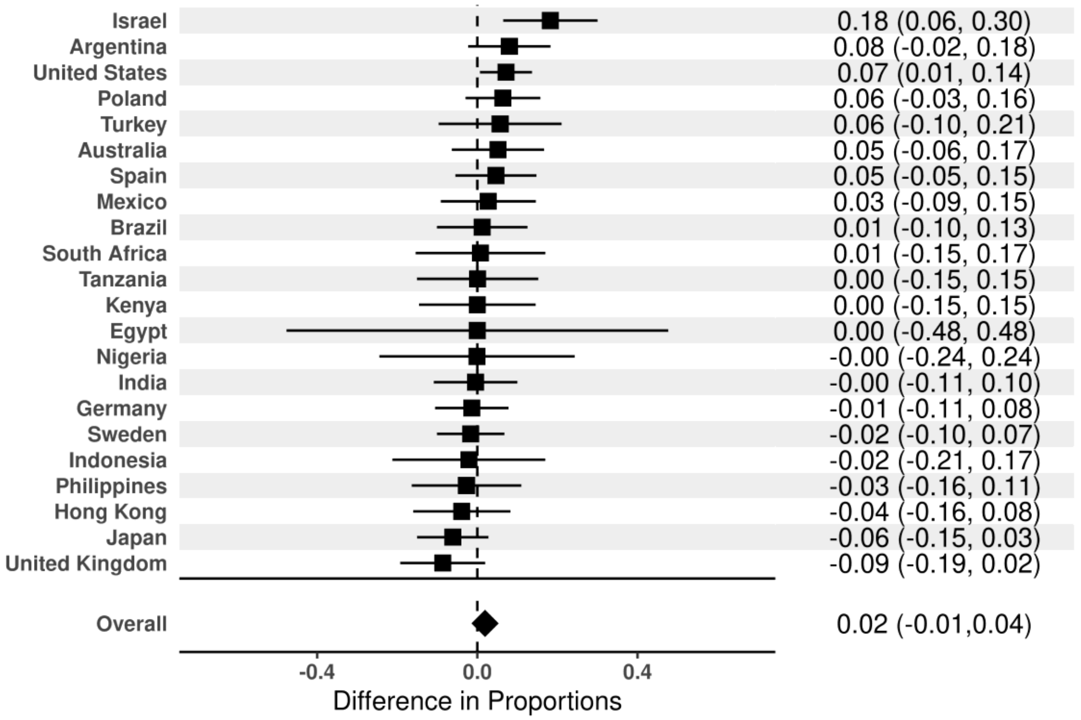

τ=0.015; Q(df=21)=22.96, p0.346;Q-profile 95% CI [0.000, 0.067]; I^2=6.19;

Figure S115. Forest plot for `Immigration status`-`(Ref: Born in this country) Born in another country`

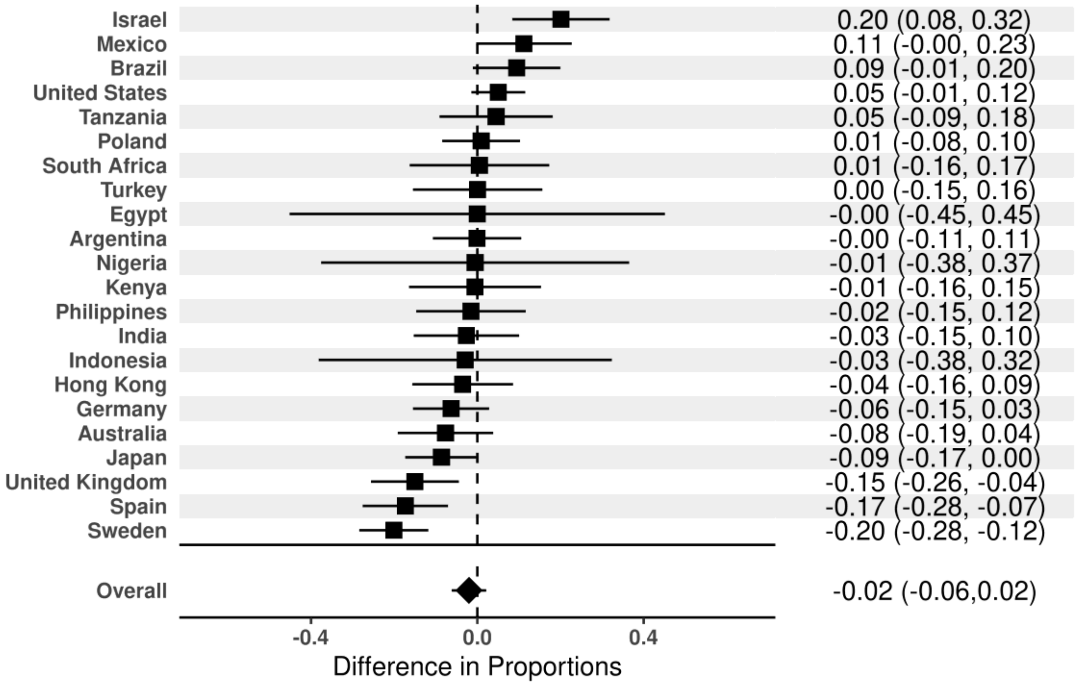

τ=0.074; Q(df=21)=66.07, p<.001;Q-profile 95% CI [0.050, 0.127]; I^2=61.58;
